# Maize protein phosphatase gene family: identification and molecular characterization

**DOI:** 10.1186/1471-2164-15-773

**Published:** 2014-09-09

**Authors:** Kaifa Wei, Si Pan

**Affiliations:** School of Biological Sciences and Biotechnology, Minnan Normal University, Zhangzhou, 363000 China

## Abstract

**Background:**

Protein phosphatases (PPs) play critical roles in various cellular processes through the reversible protein phosphorylation that dictates many signal transduction pathways among organisms. Recently, PPs in Arabidopsis and rice have been identified, while the whole complement of PPs in maize is yet to be reported.

**Results:**

In this study, we have identified 159 PP-encoding genes in the maize genome. Phylogenetic analyses categorized the ZmPP gene family into 3 classes (PP2C, PTP, and PP2A) with considerable conservation among classes. Similar intron/exon structural patterns were observed in the same classes. Moreover, detailed gene structures and duplicative events were then researched. The expression profiles of *ZmPPs* under different developmental stages and abiotic stresses (including salt, drought, and cold) were analyzed using microarray and RNA-seq data. A total of 152 members were detected in 18 different tissues representing distinct stages of maize plant developments. Under salt stress, one gene was significantly up-expressed in seed root (SR) and one gene was down-expressed in primary root (PR) and crown root (CR), respectively. As for drought stress condition, 13 genes were found to be differentially expressed in leaf, out of which 10 were up-regulated and 3 exhibited down-regulation. Additionally, 13 up-regulated and 3 down-regulated genes were found in cold-tolerant line ETH-DH7. Furthermore, real-time PCR was used to confirm the expression patterns of *ZmPPs*.

**Conclusions:**

Our results provide new insights into the phylogenetic relationships and characteristic functions of maize PPs and will be useful in studies aimed at revealing the global regulatory network in maize abiotic stress responses, thereby contributing to the maize molecular breeding with enhanced quality traits.

**Electronic supplementary material:**

The online version of this article (doi:10.1186/1471-2164-15-773) contains supplementary material, which is available to authorized users.

## Background

The reversible protein phosphorylation is a fundamental mechanism that modulates many cellular functions including regulating developmental events and perceiving environmental stimuli [1]. During phosphorylation, protein kinases (PKs) mainly phosphorylate serine (Ser), threonine (Thr) and tyrosine (Tyr) residues, while protein phosphatases (PPs) can reverse this process by removing the phosphate group. Based on substrate specificities, PPs can be categorized into two groups: Ser/Thr and Tyr phosphatases. Recently, 1241 maize PK-encoding genes have been identified, with the data suggesting that maize PKs were implicated in diverse biological processes, such as developmental control and drought stress [2, 3].The development of a flowering plant both at the cellular and organismal level is a highly complex phenomenon. In spite of the apparent autonomy, roots, leaves, and other structures perceive external signals and use different signaling mechanisms to respond. Like functions of mammalian PP genes [4], plant PPs are expected to be the key components in signal transduction networks at distinct stages of plant development and in response to multiple abiotic signals. Specific inhibitor (okadaic acid and calyculin A) of Ser/Thr phosphatases PP1 and 2A-type protein phosphatase (PP2A) arrest root hair growth in a tiny range, and severely affect the shape of cells within the elongation zone and inhibit root growth. In addition, PP2A was associated with controlling microtubules length [5]. As reported in Arabidopsis, PLL4 and PLL5 (POL-like gene) regulate leaf development, but having no detectable functions within the meristem [6]. Flowering time is a major adaptive trait and an important selection criterion in plant breeding. Kim et al. [7] showed that a self-regulatory phytochrome kinase-phosphatase coupling is a key signaling component in the photoperiodic control of flowering in Arabidopsis.

Plants as sessile organisms are constantly challenged by a wide range of abiotic stresses such as salinity, drought, and cold. However, stresses are not necessarily a problem for plants because they have used several strategies to avoid or reduce the possible damage. Drought limits plant growth and result in a drastic decline in the photosynthetic yield due to osmotic stress-imposed constraint. In order to cope with drought stress, plants utilize various mechanisms, known as drought escape, drought avoidance and drought tolerance [8]. Previous study showed that Group A PP2Cs were negative regulators in ABA signaling pathway and acted as key regulators of desiccation tolerance in land plants [9]. Salinity is one of the most severe environmental factors that greatly impacts plant development and restricts crop production. Furthermore, salt stress may cause water deficit, ion toxicity, nutrient imbalance, and oxidative stress. Interestingly, salinity tolerance is more likely to be a complex multigenic trait involving responses to cellular osmotic and ionic stresses. In Arabidopsis, over-expression of *AtPP2CG1* increased salt tolerance, whereas its loss of function impaired salt tolerance [10]. Additionally, cold stress is another key environmental factor that limits the geographic distribution of plants. Cold acclimation has been described as the process in which plants adjust their metabolism during cold treatment. In spite of the fact that maize is generally sensitive to low temperatures [11], the extent of cold sensitivity is different within the maize germplasm. The cold signal is initially perceived by plasma membrane with the help of specific Ca^2+^ channel proteins, membrane histidine kinases and some unknown sensors, which then activate the sophisticated cold-responsive signaling pathways in concert with plant hormone signaling, the circadian clock, and the developmental transition to flowering. Two PP2Cs, ABI1 and AtPP2CA, which were reported as negative regulators in ABA signaling, exhibited differences in their tissue-specific expressions as well as in temporal induction in response to chilling [12]. To improve the crop yields under abiotic stress conditions, it is crucial to understand the fundamental molecular mechanisms behind stress tolerance in plants.

One hundred twelve and one hundred thirty-two candidate PP genes were identified in Arabidopsis and rice, respectively [13, 14]. In Arabidopsis, Group A PP2Cs, ABI1 and ABI2, are negative regulators of ABA signaling, whereas Group B are characterized to regulate mitogen-activated protein kinase (MAPK) signaling cascades. POL-type phosphatases, members of Group C PP2C, are involved in flower development. Independently, another PP2C member, kinase-associated protein phosphatase (KAPP) is a singleton that regulates receptor-like kinases [15]. PTPs are critical partners for tyrosine-specific kinases in regulating the tyrosine phosphorylation status of many proteins. Tyrosine phosphorylation plays a role in MAPK signaling cascades. In particular, the level of tyrosine phosphorylation is determined by the balanced activity of protein tyrosine kinases (PTKs) and PTPs. Moreover, previous study showed that tyrosine phosphorylation is involved in phytohormone responses [16]. Compared with plants, other genomes which lack tyrosine kinases also have very few PTPs. Hence, identification of PTPs provides a stepping stone to better understanding of the functional significance of tyrosine phosphorylation in higher plants.

Although recent advances in higher plants, only a small portion of the members in this family have been found to be components of signaling pathway in maize. This include, ZmPP2Ca (ZmPP8), which is involved in the signal transduction in regulating response to drought stress [17]; ZmPP2C2 (ZmPP159), which plays a positive role in tobacco cold resistance [18]; ZmPP2C (ZmPP76), which is implicated in Arabidopsis stress signal transduction [19]; ZMPP2 (ZmPP138), which was selected as a candidate for the catalytic subunit of phospho-pyruvate dehydrogenase phosphatase (PDP) [20]; ZmRIP1 (ZmPP1), which acts as a chloroplast-to-nucleus signaling messenger, and is confirmed to function in maize redox signaling [21]; ZmPP1 (ZmPP65), whose function and regulation might be very similar to that of mammalian PPs [22].

In the post-genome era, the whole-genome sequencing, together with the global transcriptome profiling, such as microarray and RNA-seq, offer the opportunity to identify diverse gene families and to unravel the functions of genes involved in processes such as developmental regulation, disease resistance and abiotic stress. Maize is not only one of the most important food crops of the world, but also a model plant for study of the genetics in monocotyledons. In this study, we identified the full complement of PP genes in maize genome for evolutionary and functional analyses, which would help crop breeders to develop improved varieties.

## Methods

### Identification and characterization of ZmPPs

The maize protein sequences were downloaded from the Maize Genome Sequence Project (http://ftp.maizesequence.org/release-5b/filtered-set/). To uncover all the members of PPs in maize, the predicted phosphatase sequences from *Arabidopsis thaliana* and *Oryza sativa* were used as query sequences to search against maize protein database using HMMER 3.0 software [23]. Firstly, we used hmmbuild tool from HMMER to build the PP hidden Markov model (HMM) profile from the alignments of the known PP sequences. Secondly, hmmsearch program was separately applied to a search of all the protein sequences in maize protein database with the Pfam PP profiles (PF00481: PP2C, PF01451: LMWPc, PF00782: DSPc, PF00102: Y phosphatase) and PP HMM profile, and the E-value cut-off was set to 1. Subsequently, each protein sequence was subjected to SMART (http://smart.embl-heidelberg.de/) and Pfam (http://pfam.sanger.ac.uk/) databases to ensure the presence of the catalytic domain. Proteins without a phosphatase catalytic domain were removed from the dataset. After eliminating the incomplete sequences manually, 159 sequences remained were finally identified as ZmPPs and renamed based on their loci on chromosomes. The subcellular localization of ZmPPs was predicted by WoLF PSORT (http://wolfpsort.org/). In addition, the molecular weights (MWs) and isoelectric points (pIs) of ZmPPs were predicted by ExPASy Server tool (http://web.expasy.org/compute_pi/).

### Phylogenetic analysis and classification of the ZmPP gene family

Catalytic domains of ZmPPs were used for multiple alignments with the aid of MEGA5 software (http://www.megasoftware.net/) by employing ClustalW as the algorithm. Phylogenetic trees were constructed by neighbor-joining (NJ) algorithm of MEGA5. Bootstrap values from 100 replicates are indicated at each node. Our PP classes were defined based on the the classification criterion suggested by Singh et al. [13, 14].

### Gene structure and intron/exon configuration

To investigate the intron/exon structures of PP genes from maize and rice, we collected useful information from genome annotations of maize and rice from the Maize Genome Sequence Project and TIGR database, respectively. Both DNA sequences and the corresponding coding sequences were loaded into the Gene Structure Display Server (http://gsds.cbi.pku.edu.cn/). For a better visualization and comparison, the 5' untranslated region (UTR) sequences were removed beforehand.

### Chromosomal localization, gene duplication and synteny analysis

To determine the location of ZmPP genes on 10 chromosomes, we extracted data concerning gene positions from the Maize Genome Sequence Project. MapDraw 2.2 was used to visualize the locations of ZmPP genes on maize chromosomes with physical distances in million bp (Mb). We adopted a group-specific color strategy to mark each gene for better visualization and recognition. Tandemly duplicated genes were defined as closely related genes on a single chromosome, with no more than ten intervening genes [2]. In contrast, segmentally duplicated genes were detected by CoGe SynMap program (http://genomevolution.org/CoGe/SynMap.pl). In order to determine the syntenic regions between the rice and maize genomes, the positions of syntenic regions from these two genomes were collected from the results calculated by Synteny Mapping and Analysis Program (SyMAP 4.0) [24]. The segmental duplications and syntenic regions were finally visualized using Circos 0.62 (http://circos.ca).

The number of nonsynonymous substitutions per nonsynonymous site (Ka) and the number of synonymous substitution per synonymous site (Ks) of duplicated genes were calculated by DnaSP 5.0 [25]. The ratio of non-synonymous to synonymous nucleotide substitutions (Ka/Ks) between paralogs was analyzed to explore the mechanism of gene divergence after duplication. The dates of the duplication events were calculated by the equation *T* = Ks/2*λ* × 10^-6^ Mya, the *λ* = 6.5 × 10^-9^ [26].

### Expression analyses by microarray

To analyze the expression patterns of *ZmPPs*, the transcriptome data of genome-wide gene expression atlas of maize inbred line B73 were downloaded from the database PLEXdb (http://www.plexdb.org/) with the accession number ZM37. For drought treatments, microarray data were obtained from NCBI Gene Expression Omnibus (GEO) with accession number GSE16567. For each treatment, signal intensity values were normalized and biological replicate samples were averaged to generate log2 expression values for each gene. Log2-transformed values were loaded into R (15.2) and Bioconductor for expression analysis (http://www.bioconductor.org/). Limma package was applied to data processing, and heatmaps representing log2-transformed values were generated with the gplots package [27]. A hierarchical clustering algorithm was applied to determine similar pattern in expression profiles. Microarray data of cold stress were extracted from the ArrayExpress database under the experiment accession number E-MTAB-1315. For drought, differentially expressed genes were selected under a very stringent cutoff, with 0.05 *P*-value and fold change value of 2, whereas the response must be at least 2.8-fold in cold stress.

### Differential gene expression profiles based on RNA-seq

To generate the expression profiles of *ZmPPs* among different organs and development stages, the RNA-seq data were downloaded from the NCBI Short Read Archive with accession number SRP010680 (http://www.ncbi.nlm.nih.gov/sra/). This RNA-seq data analyzed 18 selected tissues representing five organs from maize inbred line B73. Normalized gene expression values were estimated by fragments per kilobase pair of exon model per million fragments mapped (FPKM). Finally, log2-transformed FPKM values from 18 tissues were used to draw heatmaps. To avoid taking the log of a number less than 1, all such FPKM values were replaced by 1. Similar to microarray analysis, both limma and gplots packages were used to generate the heatmaps.

RNA-seq data of three types of roots (CR, PR, and SR) under salt stress were fetched from GEO database at NCBI under the corresponding accession number GSE53995. Normalized signal intensity values were log-transformed for further analysis. Moreover, 2-fold change was used to test the differentially expressed genes. The up- or down-regulated genes in any tissues were calculated from the average of log-transformed normalized signal values.

### Plant materials and stress treatments

Plants of maize were grown in experimental plots with a 1:1:1 mix of peat:vermiculite:perlite. A growth room with controlled environmental conditions (15 h light/25°C, 9 h dark/20°C, relative humidity of 55%) was used for growing plants. The seedlings were watered daily for about 2 weeks. For drought treatment, 14-day-old seedlings of maize inbred line Ye478 and Han21 were exposed to drought stress for 3 or 4 days, while controls were well watered throughout this period. At the end of treatment period, the volumetric water content of the soil (measured at the depth of approximately 5 cm from the top of soil level) was approximately 12.5% for the drought-stressed plants, compared with approximately 30% for the control plants. For salt treatment, maize inbred line B73 was selected. Seedlings were watered with 200 mM NaCl for 24 h, after which all stress-treated and control seedlings were harvested, separated into shoots and roots and stored at -80°C. Each treatment was replicated three times with 5 to 7 seedlings. For cold treatment, kernels of maize inbred line Huangzao4 and Mol17 were germinated for 3 d in darkness at 25°C. Seedlings were transferred to pots containing Knop's solution and were grown in a growth chamber (photoperiod: 14/10 h day/night, light irradiance: 250 μmol quanta m^-2^ s^-1^, temperature: 24/25°C and relative humidity 60/80%). When the third leaf was fully developed, half of the plants were transferred to 8/6°C (day/night) without changing the other conditions; the other half were grown in the same conditions as before (control plants).

Seeds of *Arabidopsis thaliana* were sterilized with 2% bleach for 20 min, plated on Murashige and Skoog medium (MS; Sigma-Aldrich), chilled at 4°C for 3 d, and transferred to a growth room at 19 to 23°C with a 16 h/8 h light/dark photoperiod.

### RNA extraction and real-time PCR

To validate the RNA-seq data for selected genes under abiotic stresses, real-time PCR was carried out. The leaves and roots of the maize seedlings were gathered, immediately frozen in liquid nitrogen and then stored at a temperature of -80°C. Subsequently, total RNA was isolated from selected tissues using Trizol reagent, and the use of DNase I treatment to remove any genomic DNA contamination from RNA samples. About 2 μg of total RNA was reverse transcribed using a Takara RNA Kit to generate the single-stranded cDNA. Real-time PCR was performed on iCycler iQ5 Multicolor real-time PCR detection system (Bio-Rad) by using the Power SYBR Green PCR Master Mix (APPlied Biosystems). Two biological replicates of each sample were used and three technical replicates were performed for each biological replicate. The thermal cycling conditions are as follows: 50°C for 2 min, 95°C for 10 min, 40 cycles of 95°C for 15 s, and 60°C for 1 min. The relative quantification method 2^-ΔCt^ was used to evaluate quantitative variation between replicates. For drought and cold stresses, maize ubiquilin-1 (UBQ1) gene was used as internal control, while *ACTIN7* was an internal control for salt stress.

For *fptp1* mutant analysis, total RNA was extracted from rosette leaves of 40 day-old plants. *ACTIN8* was used as an internal control. The real-time PCR experiment was repeated for three independent biological replicates. Finally, the ABi7500 real-time PCR system with the RealMasterMix (SYBR Green I) (Takara) was used for performing real-time PCR. Primer pairs used in this study were listed in Additional file 1. The *fptp1* mutant was obtained from Institute National de la Recherche Agronomique (INRA).

### Over-expression of *FPTP1*in Arabidopsis and analysis of ABA synthetase gene expression

To generate Arabidopsis *FPTP1* over-expression transgenic plants, the coding sequence of *FPTP1* were amplified and inserted into the modified pCAMBIA1300-Super vector under control of 35S promoter. These vectors were transformed into *Agrobacterium* strain GV3101 and subsequently introduced into the Arabidopsis wile-type plants.

With the aim of identifying the contribution of PTPs in ABA accumulation, RNA was extracted using an RNeasy Plant Minikit (QIAGEN), and cDNA was synthesized using M-MLV Reverse Transcriptase (M1701) (Promega). 1 μg RNA was mixed thoroughly with 1 μg Oligo (dT)_15_, and treated at 70°C for 5 min. Subsequently, the following were added: 5 μL M-MLV buffer, 1.25 μL dNTP Mix, 1 μL M-MLV, and 0.6 μL RNase inhibitor. The resulting 25 μL reaction was incubated for 1 h at 37°C. PCR primers were showed in Additional file 1. PCR cycles included 94°C 5 min, (94°C 45 s, 58°C 45 s, 72°C 1 min) 35 cycles, 72°C 7 min.

### Subcellular location of ZmPP1 and estrogen treatment of *ZmPP1*

For subcellular localization of *ZmPP1*, its cDNA sequence was cloned into pEZS-NL transient expression vector to generate pEZS-NL-ZmPP1. Particle bombardment-mediated transient expression of ZmPP1-GFP fusion protein in onion epidermal cells were investigated by laser scanning confocal microscopy. As for GUS staining, the cDNA of *ZmPP1* was cloned into pSuper-1391 vector and then introduced into the GV3101. Floral dip method can efficiently generate the Arabidopsis transgenic plants. Then the transgenic plants were screened on solid plates containing 50 mg/mL hygromycin.

We have developed an estrogen receptor-based chemical-inducible system for *ZmPP1* transgenic Arabidopsis. The amplified *ZmPP1* cDNA sequence was cloned into pER8, of which the pER8 vector was provided by N.-H.Chua and B. Ulker [28]. Transformations of Arabidopsis were performed by the floral dip method using GV3101. Over-expression of *ZmPP1* in the transgenic lines was induced by spraying the seedling with 5 or 10 μM β-estradiol.

### Promoter activity and computational prediction of miRNA protein-coding target transcripts

To find out putative *cis*-acting elements in the promoters of putative ZmPP genes, the 1,000 bp upstream promoter regions of all *ZmPPs* were used to search for known stress-responsive *cis*-elements by PlantCARE database (http://bioinformatics.psb.ugent.be/webtools/plantcare/html/). Target-align [29], a miRNA target prediction tool, was used to predict putative miRNA target genes. For the maize protein-coding transcripts, the predicted cDNA of the longest consensus maize transcript were used. A total of 321 mature miRNA sequences downloaded from miRBase database (release 20) [30] were reverse complemented and matched against the indexed maize transcript database. With the default setting, a score cut-off of ≥ 75 and mismatch ≤ 4 nucleotides were applied in the prediction.

## Results and discussion

### Identification of PPs in maize genome

Previously, 112 and 132 PP genes were identified from Arabidopsis and rice, respectively [13, 14]. To explore the occurrence and size of the PP family in maize, HMMER program was used to search against the maize proteomics database. Further, the presence of catalytic domain was confirmed by SMART and Pfam databases. From maize genome, we identified a total of 159 putative PP-encoding genes following the removal of those sequences with an incomplete catalytic domain, suggesting that the size of maize PP family was larger when compared with that in Arabidopsis and rice. For simplicity, the 159 ZmPP genes were renamed from *ZmPP1* to *ZmPP159* according to their exact positions on maize chromosomes 1–10 and from top to bottom. The identified PP genes in maize encode proteins ranging from 131 to 1264 amino acids (aa). With the WoLF PSORT analysis, most PPs were predicted to locate in nucleus and chloroplast as well as other organelles. ExPaSy analysis suggested that the ZmPP proteins had large variations in isoelectric point (pI) values (ranging from 4.46 to 9.8) and molecular weights (ranging from 14,245.15 to 90,157.14 Da). All of the related information on ZmPPs is listed in the Additional file 2.

### Phylogenetic analysis and classification of the ZmPPs

**Figure 1:**
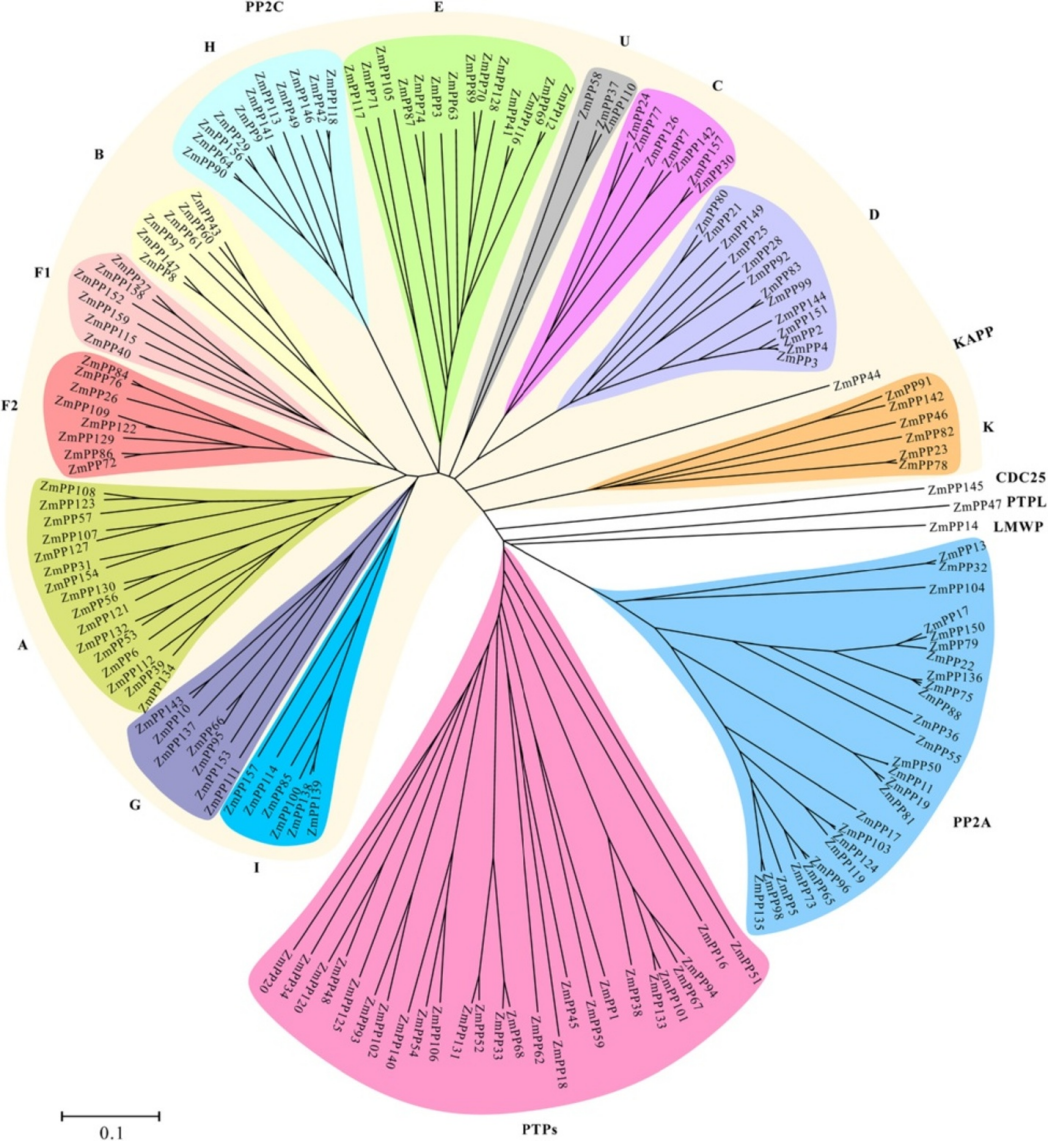
**Phylogenetic relationships of ZmPPs.** An un-rooted NJ tree is made based on the catalytic domain sequences of ZmPPs. The whole PP gene family is divided into different classes, PP2C, PP2A, PTPs, PTPL, CDC25, and LMWP. PP2C class is further subdivided into different classes (A-K). Scale bar represents 0.1 amino acid substitutions per site.

The catalytic domains of PPs are diverse even if the catalytic cores of all eukaryotic PKs share extensive similarities in both primary and three-dimensional structures. With the aim of analyzing the phylogenetic relationships of 159 ZmPPs among maize, phylogenetic tree was carried out based on the catalytic domains by NJ algorithm (Figure 1). Based on the sequence similarities of the ZmPP gene sequences and the classification of PP genes from Arabidopsis and rice, the phylogenetic tree divided the maize PP family proteins into three major classes, namely, PP2C, PTP and PPP (PP2A). PP2C was evolutionarily related to the major class of Ser/Thr PPs with 104 genes, whereas PTP and PP2A comprised of 29 and 26 members, respectively. Interestingly, all the members of PP2C class formed a single major group and this group could be further classified into 11 subgroups except some ambiguous branches. Each subgroup represents a subfamily of PP2C and is designated from A-K according to Xue et al. [31] and Singh et al. [14]. Group J PP2C did not include any rice and maize PPs but contained only members from Arabidopsis, suggesting that they might have been lost both in rice and maize after being originated from common ancestor.

In terms of the previous studies [1, 31], plant lacks typical tyrosine kinase, and most plant PPs (67%) have Ser/Thr specificity. It seems that plants prefer to use Ser/Thr as substrates for PKs and PPs, in sharp contrast with the case in human [32]. Three genes belonging to LMWP (ZmPP14), PTPL (ZmPP47) and CDC25 (ZmPP145) classes were positioned separately. PP2As, which were ubiquitous enzymes in eukaryotes, formed another single major class. Phylogenetic tree obtained from Arabidopsis, rice and maize showed very similar topologies and subfamily organization with individual maize tree (Additional file 3). It is noteworthy that the number of maize PP genes was generally overrepresented than that of Arabidopsis and rice in almost all classes.

### Gene structure analysis and intron/exon arrangement

As a evolutionary relic, it is noteworthy that intron/exon arrangement carries the imprint of the evolution of a gene family, and may provide insight into their evolutionary mechanisms underlying the origin of gene families [33]. With the purpose of understanding the structure diversity and evolution of ZmPP gene family, we analyzed the intron/exon structure, intron position and phase. A detail illustration of the intron/exon structure was shown in Additional file 4. It was found that the great majority of introns were positioned in the coding sequences of ZmPP genes. Notably, in PP2C class, the intron/exon structures were different between subclasses and correlated with the architecture of protein sequence and phylogenetic analysis. However, it was notable that a similar gene structure was found in each group, while 4 members of the 104 ZmPP2C genes (*ZmPP39*, -*112*, -*91*, and -*142*) had no introns. This indicated that the intron patterns, which correlate well with the phylogentic clades, strongly support their close evolutionary relationships among the ZmPP2C genes within the same subclass. Among those having introns, the number of introns within the open reading frame (ORF) ranged from 1 to 12, showing a great difference in the ZmPP2C class. A great degree of variation in the number of introns exists in groups E, G and K, while the number of introns in the rest groups changes within a small range, mostly from 1 to 3. In rice, similar results of gene structure analysis were given in Additional file 5, indicating that similar intron loss or gain events occurred during expansion and structural evolution of 2C-type protein phosphatase may exist in monocotyledon.

In PTPs, introns are not equally distributed across family. Additionally, we found that nearly all members (expect *ZmPP73*) have introns, while the number of introns varies widely within PP2A group. We further analyzed the exon/intron configuration of the paralogous pairs in PP gene family to obtain traceable intron gain or loss information (Additional file 5). Despite the structural conservation of intron/exon arrangement found in some paralogous pairs, others exhibited extensive variation. What's more, growing evidence has showed a functional link between the structural diversity of gene members and the evolution of multiple gene families, while intron loss or gain can be an important step in generating structural diversity and complexity [34].

### Chromosomal location and gene duplication of ZmPP genes

**Figure 2:**
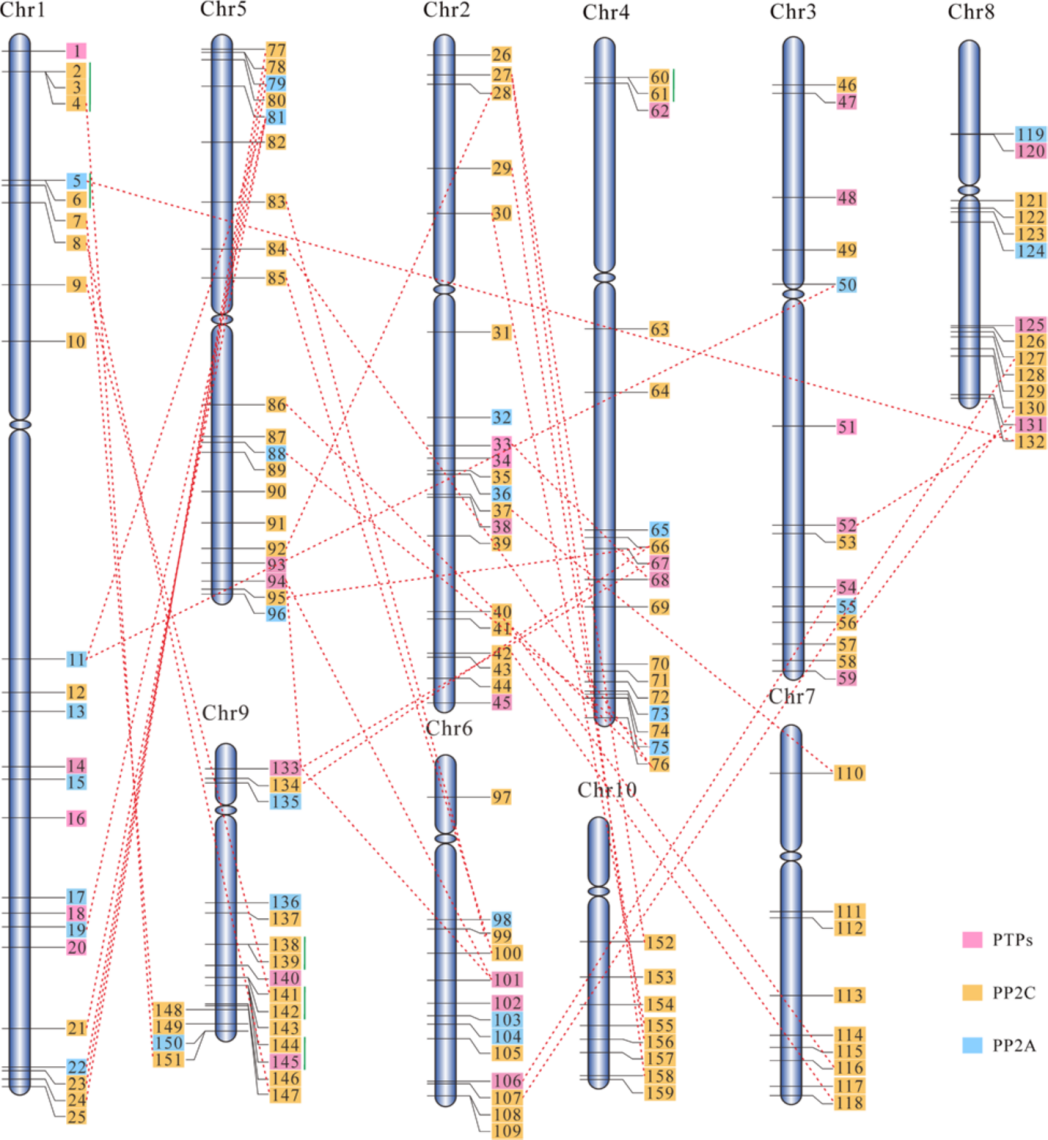
**Chromosomal localization of ZmPP genes on 10 chromosomes of maize.** Respective chromosome numbers are written at the top. Genes belonging to three classes have been marked by different colors (PTPs means protein tyrosine phosphatases). Dashed lines join the genes, lying on duplicated segments of the genome. Tandemly duplicated genes are joined with vertical lines.

To determine the genomic distribution of the ZmPP genes and study their evolution in the context of whole genome duplication, we mapped each gene on maize chromosomes based on their corresponding coordinates. As shown in Figure 2, *ZmPPs* are almost unevenly distributed across the ten chromosomes. All members of class PP2C are distributed across 10 maize chromosomes. In contrast, no PTPs and PP2As were found to be located on chromosome 7 and 10, respectively (Figure 2).

Gene duplication is prominent in eukaryotic evolution, because duplicated genes provide the raw materials for evolving a new gene, which in turn facilitate the generation of novel gene functions. Compared with most other eukaryotes, plants have substantially higher gene duplication rates in the adaptive response to environmental stimuli [35]. Previous study revealed that maize genome underwent a whole-genome duplication event (WGD) that resulted in the presence of two sub-genomes and ~30% of genes retained copies in both sub-genomes [36]. The duplication mechanism including transposition, segmental duplication, replicative transposition, or even WGD underlying gene family expansion are similar between eukaryotes. To clarify the probable relationship between ZmPP genes and potential gene duplication within the genome, we analyzed the occurrence of tandem duplication and large-scale segmental duplication during the evolution. In all, 15 (~9.4%) ZmPP genes were found to be tandem repeats with a maximum number of ten intervening spacer genes (Table 1). These 15 ZmPP genes were represented in 9 distinct tandem duplicated gene clusters, with one cluster containing 3 tandem genes and the rest of clusters possessing 2 tandem genes. It is notable that genes in one duplicated cluster may come from different group. For instance, *ZmPP144* was grouped into PP2C, but *ZmPP145* was a member of PTPs. Hence, it is speculated that tandem duplications have an indispensable role in the evolution of maize PP gene family. Additionally, previous study showed that most plants were diploidized polyploids and retained numerous duplicated chromosomal blocks in their genomes, segmental duplication occurred most frequently in plants [37]. To evaluate the contribution of large segmental duplications in the expansion of ZmPP-encoding gene family, CoGe SynMap program was used to detect segmental duplicated genes. We noted that 38 segmental duplication events in maize were observed, which were originated from a polyploidy event occurred around 15 to 20 million years ago (Table 1). Interestingly, all genes in segmental duplicated pairs were from the same classes. Thus, we speculated that segmental duplications exclusively contributed to the expansion of PP gene family in the maize genome. Taken together, our analyses suggested that large-scale segmental duplication appear to have prominently contributed to the current complexity of the maize PP family. It is consistent with the duplicative mechanism of PP gene family in rice [14].

**Table 1:** **The Ka/Ks ratios and estimated absolute dates for the duplication events between the duplicated ZmPP genes**

Duplicated pair	Ka/Ks	Date (Mya)	Duplicate type	Purifying selection	Group
ZmPP53/ZmPP132	0.2091	13.02307692	Segmental	Yes	PP2C/PP2C
ZmPP56/ZmPP130	0.35128	13.86153846	Segmental	Yes	PP2C/PP2C
ZmPP107/ZmPP127	0.26416	18.2	Segmental	Yes	PP2C/PP2C
ZmPP134/ZmPP66	0.10751	86.57692308	Segmental	Yes	PP2C/PP2C
ZmPP8/ZmPP147	0.13034	20.53846154	Segmental	Yes	PP2C/PP2C
ZmPP7/ZmPP148	0.39908	10.06153846	Segmental	Yes	PP2C/PP2C
ZmPP24/ZmPP77	1.29469	5.507692308	Segmental	No	PP2C/PP2C
ZmPP30/ZmPP155	0.15067	12.1	Segmental	Yes	PP2C/PP2C
ZmPP4/ZmPP151	0.10791	16.53846154	Segmental	Yes	PP2C/PP2C
ZmPP21/ZmPP80	0.14157	21.40769231	Segmental	Yes	PP2C/PP2C
ZmPP28/ZmPP92	0.16562	67.48461538	Segmental	Yes	PP2C/PP2C
ZmPP83/ZmPP99	0.08266	14.23846154	Segmental	Yes	PP2C/PP2C
ZmPP41/ZmPP116	0.11765	16.73846154	Segmental	Yes	PP2C/PP2C
ZmPP27/ZmPP152	1.60306	16.60769231	Segmental	No	PP2C/PP2C
ZmPP152/ZmPP158	2.32622	11.17692308	Segmental	No	PP2C/PP2C
ZmPP158/ZmPP27	0.21536	12.32307692	Segmental	Yes	PP2C/PP2C
ZmPP72/ZmPP86	0.11398	16.06153846	Segmental	Yes	PP2C/PP2C
ZmPP76/ZmPP84	0.09925	15.34615385	Segmental	Yes	PP2C/PP2C
ZmPP122/ZmPP129	0.19247	65.58461538	Segmental	Yes	PP2C/PP2C
ZmPP95/ZmPP66	0.07717	30.7	Segmental	Yes	PP2C/PP2C
ZmPP9/ZmPP141	0.18169	11.17692308	Segmental	Yes	PP2C/PP2C
ZmPP29/ZmPP156	0.16686	13.23076923	Segmental	Yes	PP2C/PP2C
ZmPP42/ZmPP118	0.10881	14.13846154	Segmental	Yes	PP2C/PP2C
ZmPP85/ZmPP100	1.28064	17.76153846	Segmental	No	PP2C/PP2C
ZmPP23/ZmPP78	0.17848	23.23076923	Segmental	Yes	PP2C/PP2C
ZmPP37/ZmPP110	0.25391	15.23846154	Segmental	Yes	PP2C/PP2C
ZmPP33/ZmPP68	0.18219	16.76153846	Segmental	Yes	DSP/DSP
ZmPP52/ZmPP131	0.12905	14.00769231	Segmental	Yes	DSP/DSP
ZmPP54/ZmPP106	0.24256	70.78461538	Segmental	Yes	DSP/DSP
ZmPP67/ZmPP133	0.33671	44.04615385	Segmental	Yes	DSP/DSP
ZmPP94/ZmPP133	2.1275	11.94615385	Segmental	No	DSP/DSP
ZmPP94/ZmPP101	1.8272	13.8	Segmental	No	DSP/DSP
ZmPP101/ZmPP133	0.13011	13.36153846	Segmental	Yes	DSP/DSP
ZmPP11/ZmPP50	0.05879	15.17692308	Segmental	Yes	PP2A/PP2A
ZmPP11/ZmPP81	0.01588	105.6230769	Segmental	Yes	PP2A/PP2A
ZmPP19/ZmPP81	0.05683	14.75384615	Segmental	Yes	PP2A/PP2A
ZmPP22/ZmPP79	0.00792	13.59230769	Segmental	Yes	PP2A/PP2A
ZmPP75/ZmPP88	0.05525	9.884615385	Segmental	Yes	PP2A/PP2A
ZmPP2/ZmPP3	0.10884	8.269230769	Tandem	Yes	PP2C/PP2C
ZmPP2/ZmPP4	0.12221	5.853846154	Tandem	Yes	PP2C/PP2C
ZmPP3/ZmPP4	0.1199	6.030769231	Tandem	Yes	PP2C/PP2C
ZmPP5/ZmPP6	0.96739	84.93076923	Tandem	Yes	PP2A/PP2C
ZmPP60/ZmPP61	0.36275	41.66923077	Tandem	Yes	PP2C/PP2C
ZmPP119/ZmPP120	0.77549	49.03076923	Tandem	Yes	PP2A/DSP
ZmPP138/ZmPP139	0.43648	2.361538462	Tandem	Yes	PP2C/PP2C
ZmPP141/ZmPP142	0.57337	85.86153846	Tandem	Yes	PP2C/PP2C
ZmPP144/ZmPP145	0.68466	50.03076923	Tandem	Yes	PP2C/DSP

*Ka/Ks < 1 means purifying selection.

To illuminate the divergence after gene duplication, the ratios of Ka/Ks were estimated for all 48 duplicated pairs. Usually, Ka/Ks > 1 means positive selection; Ka/Ks = 1 means neutral evolution; while Ka/Ks < 1 means purifying selection [38, 39]. Ka/Ks ratios of most duplicated pairs, regardless of whether they are orthologs and paralogs, were less than 1, subjecting to purifying selection. Nevertheless, there are 5 segmentally duplicated pairs suffered positive selection with Ka/Ks ratios greater than 1. Among these duplicated pairs, the average Ka/Ks value of segmental duplication was very similar to those in tandem duplication (~0.4), indicating that most duplicated ZmPP genes are under strong purifying selection pressure. The duplication events for the segmental duplicated genes were estimated to occur approximately 24 Mya, while that in tandem duplicated pairs were estimated to occur approximately 37 Mya, indicating that the segmental duplication events might occur before the emergence of tandem duplication events. Therefore, the segmental and tandem duplication events played essential roles in evolutionary expansion of PP family in maize.

### Expression profiles of maize PP genes and their potential functions in different tissues and developmental stages

**Figure 3:**
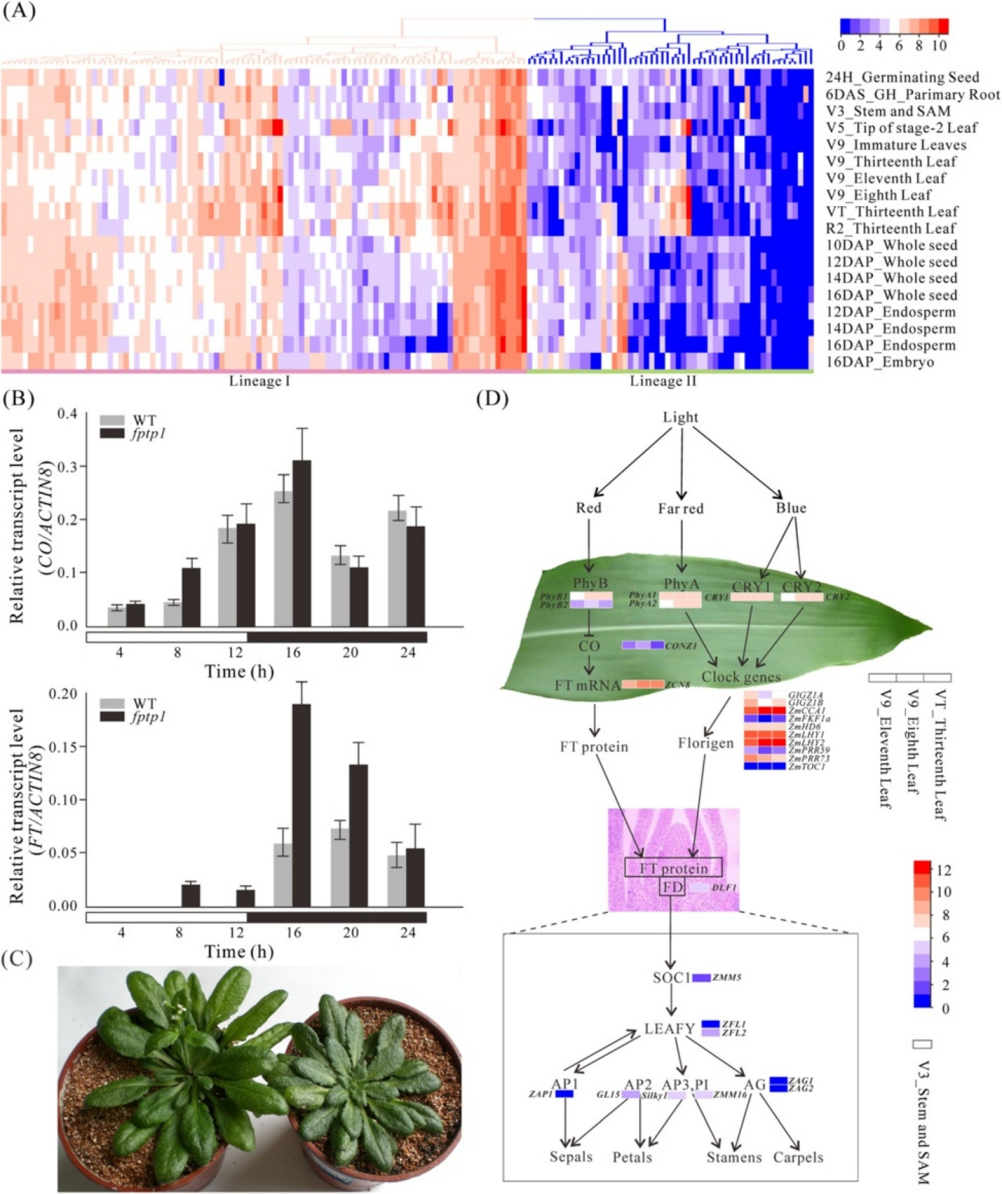
**RNA-seq analysis of** ***ZmPPs*** **in 18 selected tissues and a genetic regulatory network for flowering time control in maize. A** Expression profiles of 152 detected *ZmPPs* in 18 tissues from different developmental stages. **B** Real-time PCR analysis of *CO* and *FT* in *fptp1* mutant. **C** Plant images of *fptp1* transgenic plants. Left, wild type plant; right, *FPTP1* overexpression transgenic line. **D** Overview of flowering time pathway in maize.

Recent advances in functional analyses of PPs have revealed the importance of some PP genes in the life cycles of both Arabidopsis and rice [14, 40], however, their roles in maize remained unclear. Aim for achieving gene expression patterns of *ZmPPs* in diverse growth phases, we downloaded the genome-wide expression data of maize inbred B73 from PLEXdb and analyzed the expression profiles of *ZmPPs* across the 60 different developmental stages of 11 organs. Following whole-chip data processing, the log2 values of 154 PP genes were showed as heatmap (Additional file 6). From Additional file 6, we found that *ZmPPs* were implicated in a wide range of plant development processes, including root development, leaf development gradient and flower induction. In addition to conducting microarray analysis, RNA-seq data with 18 tissues representing distinct stages of maize plant development were selected for analysis. A total of 152 *ZmPPs* were reliably detected, while the remaining 7 members were undetected (Figure 3A, Additional file 7). We also calculated the CV value (CV = S/X_mean_, where S represents the standard deviation and X_mean_ indicates the mean expression of a gene across all the tissues) of each gene to elucidate the expression profiles of differentially expressed genes [41]. Based on their expression levels and CV values, these detected PP genes were categorized into two lineages. Lineage I showed higher expression levels, but lower CV values compared with lineage II. As shown in Additional file 7, genes with higher CV values displayed higher fluctuation in their expression levels.

Recently, a systematic survey of morphology and histology of young seedling root in maize hybrids and their parental inbred lines has been performed [42]. Before this, most of our knowledge about the complexity of auxin signaling in regard to root development was based on the molecular and physiological analyses in Arabidopsis, which laid the groundwork for the discovery of parallel pathways using maize as a model for monocot plants. To better understand the role of auxin in maize root development, we searched for some key factors in the signaling pathways (Additional file 8). In comparison with Arabidopsis and rice, less development-regulated genes and their functions in maize have been characterized in detail. In our study, identification of key genes involved in root development signaling pathway in maize, such as auxin transporters (PIN) and auxin response factors (ARF) were performed using HMM-based searches. All genes involved in this developmental signaling pathway were showed in Additional file 9. It has been reported that PP2As are required for asymmetric auxin distribution in seedling roots of Arabidopsis [43]. During male and female inflorescence differentiation and kernel development, the transcripts of ZmPIN gene were showed to have overlapping expression domain in the root apex [44]. Moreover, the inhibition of PP2A leads to a preferential apical PIN targeting [43]. In our microarray data, *ZmPP65* was relatively high expressed in root, indicating that *ZmPP65* might play an important role in auxin-induced root development. Interestingly, another two genes (*ZmPP67* and *ZmPP94*), which belong to PP2C class, exhibited high expression levels in root. Thus, we hypothesize that except for PP2A, other classes of PP genes may participate in root development as well, such as PP2C. Moreover, expression patterns of other PP genes involved in maize root developmental pathway were analyzed as well (Figure 3A, Additional file 9). Most of them were highly expressed in root, which were in accordance with the results that other PP genes except PP2As play roles in root development as well.

It has been reported that maize leaves are characterized by clear proximal/distal domains as seen in the morphological differences of the sheath and blade. In our research, it was noteworthy that two genes (*ZmPP145* and *ZmPP58*), belonging to lineage I, were found to display high expression levels at V5_Tip of stage-2 Leaf, while *ZmPP58* was highly expressed at V9_Eighth leaf. It is speculated that *ZmPP145* and *ZmPP58* may play critical roles in early leaf developmental stages (V5 and V9). To provide a broader insight into the maize leaf development, four representative zones of the leaf blade were selected for deeper transcriptomic analysis (Additional file 10) [45]. Of 159 ZmPP genes, a total of 88 genes were expressed at all four sampled sections of the maize leaf. Most of them were up-regulated in all four regions of maize leaf. Especially, *ZmPP47* showed increased transcript abundance in the basal (base, 1 cm above the leaf three ligule) and transitional zones (-1 cm, 1 cm below the leaf two ligule), while *ZmPP58* and *ZmPP133* were highly expressed in the maturing (+4 cm, 4 cm above the leaf two ligule) and mature zones (tip, +1 cm below the leaf three tip), which might participate in the transcriptional regulation of carbon fixation pathways. Arabidopsis *PLL4* (a member of PP2C class) was reported to play a part in the regulation of leaf development, while its homolog in maize (*ZmPP24*) showed higher expression in the maturing and mature zones as well. Strikingly, 4 genes (*ZmPP48*, -*113*, -*120* and -*146*) were obviously down-regulated in the maturing and mature zones with their functions in leaf development remain unknown. Overall, genes with specific expression patterns in this study might play critical roles in leaf development.

Morphogenesis is referred to as a process in developmental biology in which organisms develop their characteristic shapes. Furthermore, flowering and architecture development are two key processes in plant morphogenesis, which are of particular significances in crop. Modulation of the life cycle by photoperiodic signaling is a fundamental mechanism for plant to cope with the seasonal changes. As reported by Dong et al. [46], both environmental and endogenous factors control the shoot apical meristem (SAM) transition from the vegetative to the reproductive stage. In Figure 3A, *ZmPP67* was expressed at a relative high level in both root and SAM, suggesting that *ZmPP67* might participate in floral transition. To validate the exact role of *ZmPP67* in maize flowering time pathway, the function of Arabidopsis FLOWERING ASSOCIATED PTPase1 (FPTP1, Genebank accession number: FJ605097), which is homologous to *ZmPP67*, was analyzed by mutant analysis. *CO*, a circadian-regulated gene, and *FLOWERING LOCUS T* (*FT*), belonging to the BBX and PEBP family, respectively, contribute to the photoperiod flowering control in plant [47]. As showed in Figure 3B, mutation in *FPTP1* significantly promoted *FT* expression but had no obvious effect on *CO* expression. Furthermore, over-expression of *FPTP1* caused delayed flowering (Figure 3C). Thus, we suspected that ZmPP67 may function in the upstream of FT.

It was reported that many of the flowering time pathways in Arabidopsis and rice are conserved in maize [47]. Through a genetic and molecular model for flower development in Arabidopsis, a possible flowering time pathway in maize was proposed (Figure 3D). Light is the most important environmental cue implicated in the regulation of flowering time in plants. In maize, a reduced light response is associated with the development of early flowering inbred lines [48]. Phytochromes and cryptochromes are the primary red/far-red and blue light photoreceptors, respectively, with three pairs in our study, PhyB1/2, PhyA1/2, and CRY1/2. In our expression profiles of genes involved in flower induction (Figure 3D), *PhyA*, *PhyB* and *CRY1/2* were expressed slightly higher levels in leaves compared with other tissues. Particularly, expressions of *PhyB1* and *PhyB2* are highly similar to that of PhyA1 and PhyA2 genes. Previous study showed that *PhyB* regulates CO protein, but not mRNA level [49]. *CO* coordinates light and clock input in leaves to trigger the expression of florigen gene *FT*. Consequently, we suspected that the effect of *CO* on *FT* expression must result from a post-transcriptional regulation. DLF1, homologous to Arabidopsis FD, form a complex with ZCN8 (maize FT protein) to activate downstream floral organ identity genes, such as *ZMM5*, the homolog of Arabidopsis *SOC1* in maize. Then, *AP1/3*, *PI* and *AG* were the downstream of *LEAFY* in Arabidopsis, and the homologs in maize were identified in this study as well as their expression patterns were analyzed. It was showed that *DLF1*, *ZMM5*, *ZFL2* (*LEAFY*), *GL15* (*AP2*), *Silky1* (*AP3*), and *ZMM16* (*PI*) were relatively highly expressed in SAM compared with other tissues, while other genes in the downstream of LEAFY, including *ZAP1* (AP1) and *ZAG1/2* (AG) had lower expression. All genes involved in the flowering time signaling pathway were showed in Additional file 11.

In addition to RNA-seq analysis in 18 representative tissues during maize development, another RNA-seq study of 12 maize reproductive tissues that represent male, female and developing seed was carried out. Overall, ~96% (153) of *ZmPPs* were detected in reproductive transcriptome (Additional file 12). It was noteworthy that *ZmPP37*, -*76*, -*81*, -*84*, -*90*, and -*110* were significantly highly expressed in pollen, notably, *ZmPP76* and *ZmPP84* were specially expressed in anther. Additionally, *ZmPP15*, -*63* and -*97* had relatively high expression values in both anther and pollen. Hence, genes with expression restricted to specific tissues within this study are strong candidates for further analysis to unravel the mechanisms of vegetative and reproductive growth.

### Expression profiles of *ZmPPs*under abiotic stresses

#### Expression analysis of ZmPP genes under salt stress

**Figure 4:**
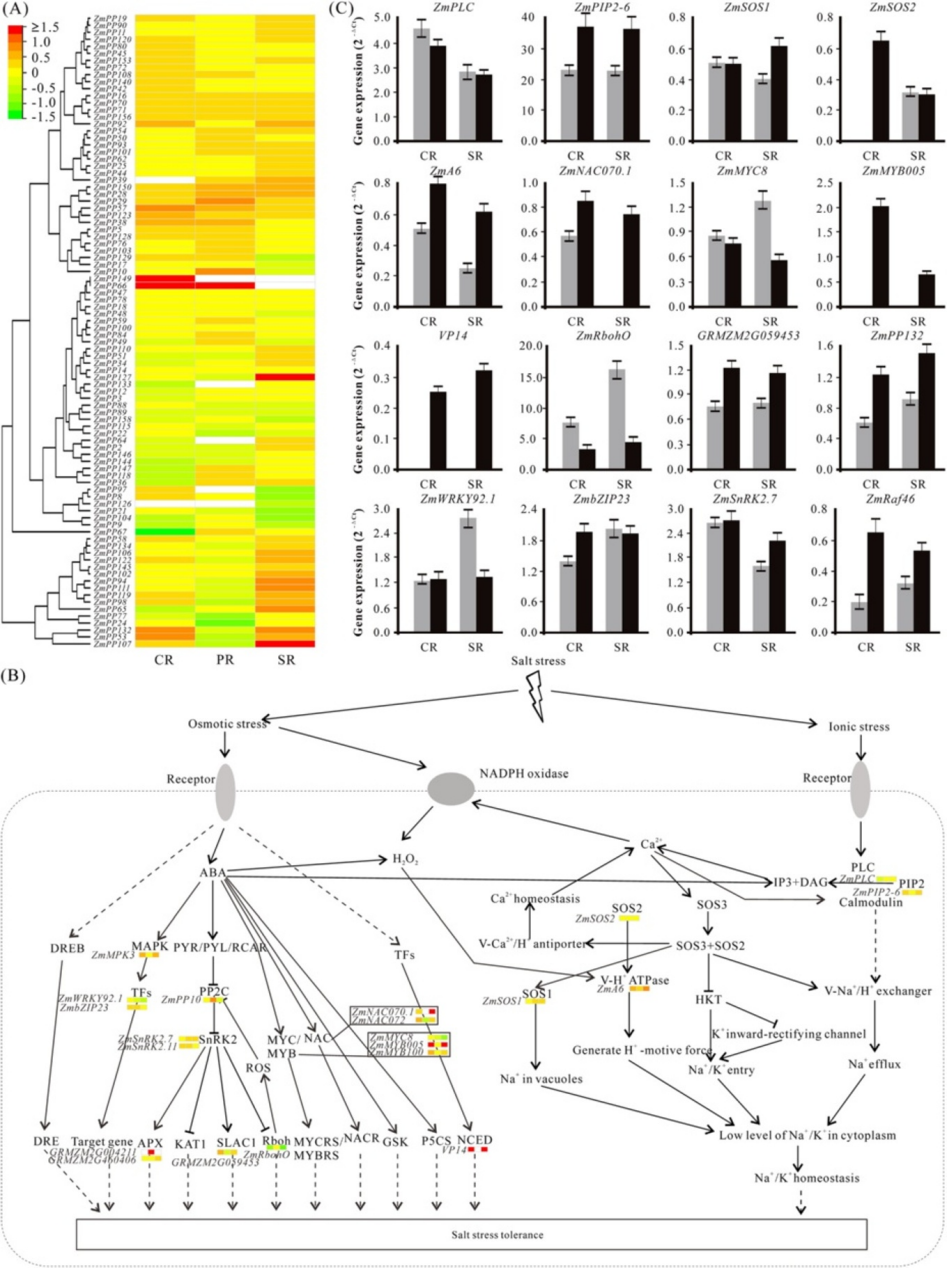
**Differential gene expression of ZmPP genes and overall signaling pathway under salt stress. A** Expression profiles of *ZmPPs* under salt stress in crown root (CR), primary root (PR), and seminal root (SR). Log2 signal intensities were used to create the heatmap. White mean only expressed in control or have no expression value both in control and salt stress treatment. **B** Overview of salt-responsive systems in plants. **C** Real-time PCR results of salt-induced pathway components. Grey, control; black, salt treatment.

Plants encounter a wide range of environmental insults, which pose serious threat to crop production. Among all the abiotic stresses, salinity represents the major constraint for agricultural productivity. Maize is a salt-sensitive crop plant and responds to salt stress via complex changes at both the transcriptional and post-transcriptional levels. Plant roots are highly sensitive organ and induce primary response to salt stress. To better understand the different responding mechanisms to salt stress in maize root, examination of three types of roots (CR, PR, and SR) under salt treatment was performed. As shown in the Figure 4A, three specific genes were significantly and differentially expressed under salt stress, namely, *ZmPP149*, *ZmPP66*, and *ZmPP127*. Surprisingly, all of them are the members of PP2C class, and *ZmPP66* is a member of Group G PP2C. It has been demonstrated that *AtPP2CG1* (*Arabidopsis thaliana* protein phosphatase 2C G Group 1) can positively regulate salt-tolerance of Arabidopsis in ABA-dependent manner [10]. Intriguingly, *ZmPP10*, which is homologues to *AtPP2CG1*, showed significantly up-regulated expression in PR. In addition to these three specially expressed genes (*ZmPP149*, *ZmPP66*, and *ZmPP127*), *ZmPP107*, a member of Group A PP2C, was significantly up-regulated in CR and SR, indicating that Group A PP2C may play a part in salt stress except for its role in drought stress condition.

To clearly understand the roles of ZmPPs in salt stress-induced signaling pathway, salt stress signaling and mechanisms of maize salt tolerance were analyzed. Salt stress involves both osmotic stress and ionic stress that limit the efficiency of crops and especially quantity and quality of their metabolic (secondary plant products) products. Osmotic stress can rapidly increase ABA biosynthesis, and regulate ABA-dependent stress response pathway [50]. The expression patterns of key genes involved in ABA biosynthesis showed that *VP14* (*ZmNCED1*) and *ZmAO2* (the homolog of Arabidopsis *AAO3*) were significantly up-regulated in CR and SR (Figure 4B). Several salt stress-inducible genes were identified in this study and showed in Additional file 13. To detect whether all of these genes were implicated in salt signal transduction pathways, RNA-seq analysis of three types of roots of maize under salt treatment was performed (Additional file 14). As the partner of PPs, MAPK was reported to function significantly in salt signaling in yeast [51]. To investigate the role of MAPK cascades in maize salt stress, the expression patterns of MAPK pathway components in the three types of roots were analyzed. As shown in Additional file 15, we found that *ZmRaf46* was expressed at relatively high level in SR and CR compared with PR, indicating that MAPK cascades might play crucial roles in salt stress response in maize. Apart from MAPK, SNF1-related protein kinases (SnRKs) which acted as the downstream elements of PPs, function in salt stress tolerance as well [52]. Among the 14 maize *SnRK2s*, *ZmSnRK2.7* and *ZmSnRK2.11* seem to play roles in salt stress tolerance with relative up-regulations (Additional file 14). Transcription factors (TFs) including MYB/MYC, NAC, WRKY, and zinc-finger protein have also been identified as salt stress-responsive factors in plants [50]. *ZmWRKY33* (named *ZmWRKY92.1* in our study), was reported to be significantly up-regulated in salt stress [53, 54]. In contrast, the expression level of *ZmWRKY92.1* has no obvious change in CR under salt stress, but is slightly down-regulated in PR and SR, indicating that *ZmWRKY92.1* might act as a negative regulator of salt stress response. On the other hand, bZIP TFs function as ABA-dependent transcription factors and may be candidate genes enhancing crops stress tolerance [33, 55]. With the result of RNA-seq analysis, *ZmbZIP23* expression was up-regulated by salt treatment in maize CR and PR, suggesting that *ZmbZIP23* might play a partial role in response to salt stress. MYB TFs ,which are characterized by highly conserved MYB DNA-binding domain, are involved in the regulation of salt stress response through ABA-independent pathway [50]. In this study, both *ZmMYB005* and *ZmMYB100* showed increased expressions in salinity, suggesting that they may act as master switch in the salt stress tolerance. NAC-type TFs also regulate some salt-responsive genes through ABA-dependent signaling pathway. In our analysis, high salinity stress induces several *ZmNACs* as well. It is notable that *ZmNAC070.1* and *ZmNAC072* showed significantly up-regulated expression in CR and SR. Additionally, plant exposure to high levels of NaCl also creates ionic stress in the form of cellular accumulation of Cl^-^ and, in particular, Na^+^ ions. Previous study showed that the vacuolar Na^+^/H^+^ antiporter gene, *ZmNHX* displayed an up-regulation in root when exposed to high NaCl concentration [50]. What's more, salt stress induce either influx or efflux of nutrient ions such as Ca^2+^, K^+^, and NO^3-^. The salt overly sensitive (SOS) signaling pathway that comprises SOS1, SOS2, and SOS3 is a well known signaling pathway for resistance to salt stress [50]. SOS2, a Ser/Thr PK, forms a complex with SOS3, an EF-hand Ca^2+^-binding protein, to activate SOS1, which plays a central role in sodium extrusion and controlling long distance Na^+^ transport. Moreover, SOS2 also works to activate and interact with vacuolar N^+^/H^+^ and H^+^/Ca^2+^ antiporters and V-ATPase, which will contribute to sequestration of excess Na^+^ ions, further resulting in Na^+^ ion homeostasis.

To confirm the expression patterns of these predicted genes under salt stress, 16 genes were selected for real-time PCR analysis. As shown in Figure 4C, most of the selected genes were up-regulated in salt stress, while few were down-regulated. Among these 16 genes, *ZmMYB005*, *VP14*, and *ZmRaf46* were specially expressed under salt stress in both CR and SR, and *ZmNAC070.1* displayed up-regulation in CR and had specific expression in SR. Moreover, *ZmSOS2* was expressed specially in CR but has no obvious fluctuation in SR. Taken together, real-time PCR-based expression profiling for these selected genes confirmed the outcome of RNA-seq analysis. Combined with RNA-seq, real-time PCR, and co-expression analysis (data not shown), ZmRaf46/49/17-ZmMKK3-ZmMPK3 might represent a potential MAPK cascade involved in salinity stress.

#### Differential expression of ZmPP genes under drought stress

**Figure 5:**
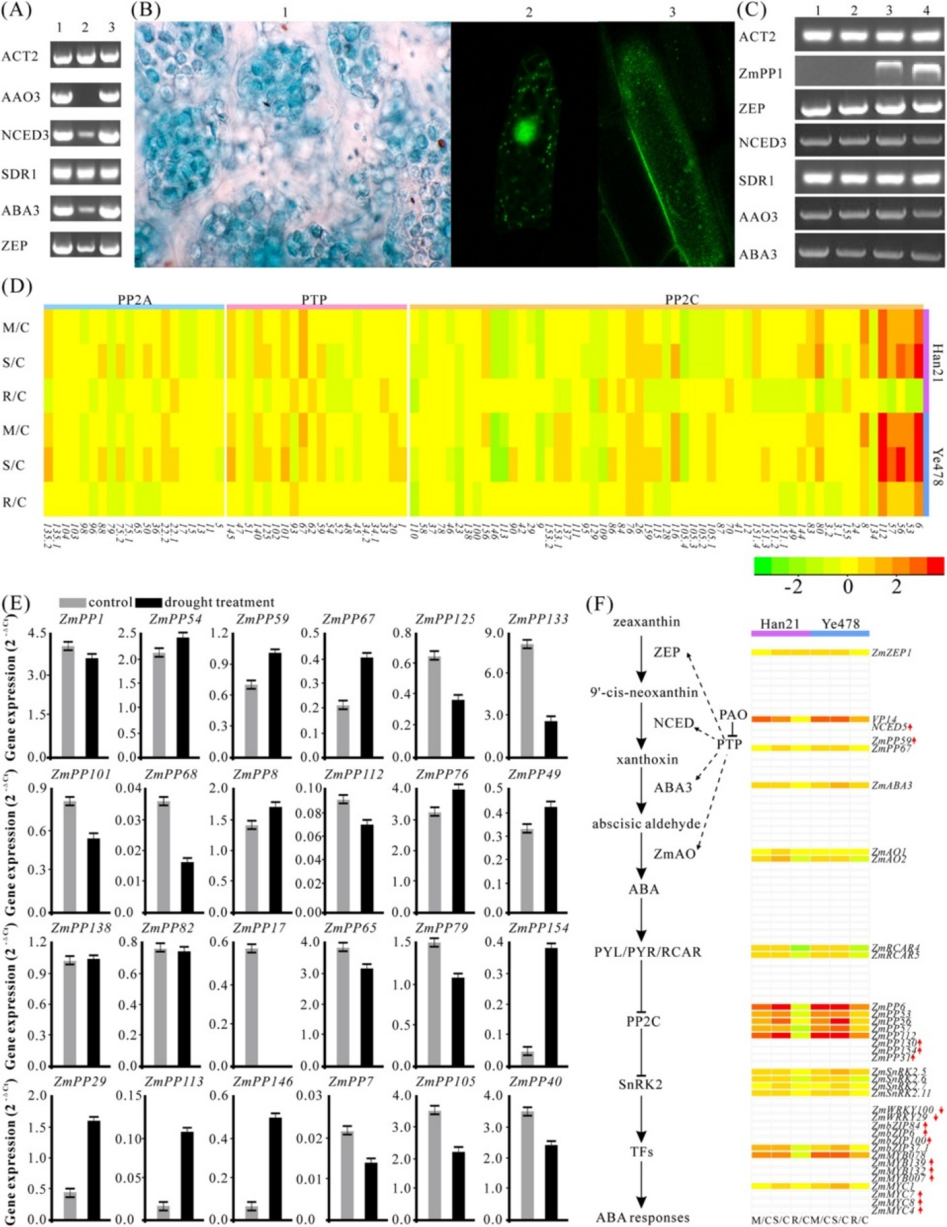
**Differential gene expression and real-time PCR analysis of ZmPP genes. A** Effect of PAO on the key enzymes of ABA biosynthesis under water stress condition in Arabidopsis. 1, control; 2, PAO-treated; 3, negative control. **B** 1, stable expression of ZmPP1-GUS fusion protein in Arabidopsis chloroplast; 2, transient expression of ZmPP1-GFP fusion protein in onion epidermal cell; 3, transient expression of GFP protein only. **C** Analysis of gene expression in Arabidopsis transformed with estradiol-inducible *ZmPP1*. 1, wild-ethanol treated; 2, wild-estradiol treated; 3, *ZmPP1* over-expression1 (*ZmPP1OE1*)-estradiol treated; *ZmPP1OE2*-estradiol treated. **D** Expression profiles of ZmPP genes under moderate drought stress (M/C), severe drought stress (S/C), and re-watering (R/C) as compared to control seedlings in Han21 and Ye478, respectively. **E** Real-time PCR analysis of 24 differently expressed PP genes under drought stress. **F** ABA biosynthesis and ABA-dependent pathway of response to drought in plants. The red arrows represent expression levels only detected by RNA-seq under drought stress.

It is well known that drought is a major environmental factor determining plant productivity and distribution. The previous researches suggested that some PP genes not only played crucial roles in stress-induced ABA accumulation but also function in redox signaling in maize [19, 21]. PTP is a group of PP with 29 members in maize. To gain an insight into the functions of PTPs in ABA accumulation and stress responses, we examined the effect of PAO (a PTPase-specific inhibitor) [21] on the expression of the key enzymes involved in ABA biosynthesis, such as AAO3, NCED3, SDR1, ABA3, and ZEP in Arabidopsis. ZmPP1 is sensitive to the PAO and 50 μM PAO was use to inactivated the enzyme. We found that PAO can arrest the expression of genes encoding the key enzymes (*AAO3*, *NCED3*, and *ABA3*) in the ABA biosynthesis pathway, indicating that PTPs may have the potential roles in ABA biosynthesis (Figure 5A). Li et al. has showed that *ZmRIP1* (named *ZmPP1* in this study), which is a member of PTPs, functioned in redox signaling in maize [21]. To better understand the cellular function of ZmPP1, we further examined its subcellular location (Figure 5B, Additional file 16). When GFP-ZmPP1 was introduced into onion epidermal cells, we found that ZmPP1 had multiple subcellular locations, whereas GFP alone was localized to the nucleus and cytoplasm (Figure 5B). Moreover, histochemical staining revealed that *GUS* expression occurred mainly in the chloroplasts. However, stable expression of *ZmPP1* in Arabidopsis based on estrogen-receptor-based inducible system showed that *ZmPP1* is not a mediator of stress-induced ABA accumulation (Figure 5C).

To test whether the *ZmPTPs* play roles in drought stress, microarray data of different drought treatments were used to generate a heatmap. This genomic study of maize gene expression in response to water deficit include two different inbred lines, namely, drought-tolerant line Han21 and drought-sensitive line Ye478 (Figure 5D). Besides DNA microarray analysis, RNA-seq analysis was performed to investigate the functions of *ZmPTPs* in different drought sensitive organs, namely, leaf and cob, under drought and well-watered conditions [56]. Imposing the two-fold change requirement with a *P* value <0.05, the public microarray data showed that expression of *ZmPP67*, a member of PTP subfamily, was up-regulated in Han21. Further observation from RNA-seq data showed that *ZmPP67* was significantly up-regulated in both leaf and cob, indicative of its important role in response to water deficit conditions. To confirm the expression patterns of *ZmPTPs* under water deficit condition, real-time PCR analysis was carried out using 8 differently expressed genes. As shown in Figure 5E, *ZmPP67* was obviously up-regulated in water deficient, which is consistent with our previous assumption that *ZmPP67* might have an essential part during drought stress. Moreover, from RNA-seq and real-time PCR data, it was interesting to find that the other two PTP genes, *ZmPP54* and *ZmPP59*, whose expression levels were also up-regulated. Of these 8 differentially expressed genes, 4 genes (*ZmPP125*, -*133*, -*101* and -*68*) were down-regulated in water deficit condition. But the functions of these 4 genes in drought stress are still unknown.

Previous studies have reported that 6 Arabidopsis Group A PP2Cs interacted with ABA receptors and functioned as key negative regulators in ABA signaling [9, 57]. Thus, in addition to PTPs, other PP genes may be involved in drought response. In RNA-seq data (Additional file 17), 10 genes (*ZmPP6*, -*21*, -*29*, -*31*, -*113*, -*124*, -*127*, -*130*, -*146*, and -*154*) were significantly up-regulated in leaf with the criterion that fold change > 2. Of these 10 genes, all of them (except *ZmPP124*) were belong to PP2C class. After integrating the RNA-seq and real-time PCR data (Figure 5E, Additional file 17), it is notable that several putative PP orthologs among Arabidopsis, rice and maize showed strikingly consistent expression patterns, which lend further supporting to the existence of functional conservation among these species. For instance, *ZmPP2C* (named *ZmPP76*), which was reported to be essential for plant stress signal transduction, showing up-regulated expression pattern in drought stress [19]. In our study, *ZmPP76* was slightly up-regulated, whose expression pattern was in good agreement with the previous report [19]. Interestingly, another stress-induced gene *ZmPP2Ca*, named *ZmPP8* in this study, was up-regulated by drought. In contrast, previous study has revealed that down-regulation of the *ZmPP2Ca* transcript was observed in drought tolerant lines, whereas transcript levels were up-regulated in sensitive lines under drought stress [17]. In addition, *ZmPP112*, the homolog of rice protein phosphatase *OsPP2C1*, was up-regulated more significantly in the drought sensitive line Ye478 than that in the drought tolerant line Han21. Previous study showed that *OsPP2C1* was highly induced in response to abiotic stresses, even up-regulated at low temperature and drought conditions [58]. On the contrary, RNA-seq data and real-time PCR analyses of *ZmPP112* exhibited decreased expression level in drought. Recently, Américo et al. reported that two members of Group A PP2C (ABI1 and PP2CA) can interact with the SnRK1 catalytic subunit, causing its dephosphorylation and inactivation, while the inhibition of PP2C allows ABA to promote SnRK1 activation [59]. *ABI2*, the homolog of *ABI1* in Arabidopsis, has been reported to involve in ABA signal transduction with up-regulated expression pattern [60]. Similarly, *ZmPP49*, the homolog of Arabidopsis *ABI2* in maize, was up-regulated, indicating that ZmPP49 might interact with ABA receptors to suppress the activity of SnRKs.

Apart from the above putative genes up-regulated by ABA, another two genes (*ZmPP138* and *ZmPP82*) were expressed at nearly equivalent levels. As previously reported, the expression level of *ZmPP138* (published *ZMPP2*) was relatively high in root and etiolated shoots [20]. Also, *OsBIPP2C1*, the homolog of *ZmPP82* in rice, was up-regulated upon drought treatments [61]. In an attempt to examine the effects of drought stress on the expression of the PP2A genes, comprehensive analysis on microarray, RNA-seq and real-time PCR was done (Figure 5D-E, Additional file 17). Especially, *ZmPP17* and *ZmPP79*, two homologs of rice PP2As (*OsPP2A-4* and *OsPP2A-2*), showed special expression patterns. *OsPP2A-4* is expressed at comparably high levels in stems, roots and flowers, while an appreciably lower level of expression is observed in leaves. However, *ZmPP17* was not expressed during drought in leaf [62]. Additionally, the expression level of *OsPP2A-2* was significantly down-regulated in leaves and stems in response to drought stress, which is consistent with the expression pattern of *ZmPP79* in our study [62]. Another PP2A gene, *ZmPP65*, showed down-regulated expression in leaf under drought stress. In addition to those genes that have been reported or had homologs in other species, some maize PP genes were also found to be up-regulated under water deficit, such as *ZmPP29*, -*113*, -*146* and -*154*. Typically, *ZmPP154* is a member of Group A PP2C in maize. In addition, *ZmPP7*, *ZmPP105* and *ZmPP40* were especially down-regulated under water deficit condition.

A putative schematic of the ABA biosynthesis and ABA cellular response during drought stress was proposed based on the described web-like networks of ABA biosynthesis and ABA signaling under water shortage condition (Figure 5F). Compared with Arabidopsis, maize has less characterized drought-induced genes (Additional files 18 and 19). Based on our investigation data and the previous research results [63, 64], we found that ABA biosynthetase genes were up-regulated in the drought-tolerant line Han21, although their protein levels have not been examined in most cases.

#### Expression profiling of ZmPP genes under cold stress

Cold responses have been observed in many plants, like Arabidopsis and rice, which alter mRNA levels of genes belonging to multiple independent pathways to response to low temperature [65, 66]. To search for the molecular mechanism of high intraspecific diversity in cold tolerance of maize, transcriptomic analysis was studied in two inbred lines contrasting in chilling tolerance (Additional file 20). With the criterion that the response must be at least 2.8-fold (|log2[(expression level in the cold):(expression level in control conditions)]| ≥ 1.5) [11], 13 ZmPP genes (*ZmPP6*, -*24*, -*29*, -*66*, -*77*, -*82*, -*92*, -*112*, -*116*, -*127*, -*149*, -*154*, and -*155*) were found to be significantly up-regulated, while three genes (*ZmPP69*, -*87*, and -*101*) displayed obvious down-regulation. AtPP2CA, an Arabidopsis group A PP2C, was reported as a negative regulator in ABA responses during cold acclimation [12]. *ZmPP6*, the homolog of *AtPP2CA* in maize, which was up-regulated in cold-tolerant line ETH-DH7. In addition, *ZmPP2C2*, named *ZmPP159* in this study, occurred to be a positive regulator of cold resistance in plant [18], whose expression was slightly up-regulated. It is conceivable that maize PP2C activity could be modulated via cold stress-induced response. To investigate the role of MAPK cascades in cold tolerance, we performed microarray analysis of components of MAPK pathway (Additional file 21). In cold tolerant line, three *MAPKs* (*ZmMPK6*, -*8* and -*12*), two *MAPKKs* (*ZmMKK2* and *ZmMKK4*) and four *MP3Ks* (*ZmMAPKKK1*, *ZmMAPKKK17*, *ZmZIK9* and *ZmRaf43*) were significantly up-regulated. To explore the gene expression patterns in our maize inbred lines under cold stress, the obviously up-regulated *ZmPPs* and the components of MAPK cascades were selected for real-time PCR analysis. As shown in Additional file 22, all the selected genes were up-regulated under cold treatment. We proposed that MAP3K1/17/ZIK9/Raf43-MKK2/4-MAPK6/8/12 might represent putative MAPKK-MAPK or MAPKKK-MAPKK-MAPK cascades based on microarray, real-time PCR and co-expression analyses (data not shown). The predicted MAPKKK-MAPKK-MAPK phosphorylation regulatory network constitutes a valuable resource to understand the function and specificity of MAPK signaling systems in maize. It is worth mentioning that ZmMP3K1- and ZmMPK12-overexpression in tobacco showed higher seed germination rate under low temperature treatment (12°C) (data not shown).

### Promoter analysis and miRNA targets of *ZmPPs*

In genetics, a promoter is a region of DNA that initiates transcription of a particular gene and confers developmental and/or environmental regulation of gene expression. Discovery of *cis*-regulatory elements in the promoter regions is essential to understand the spatial and temporal expression pattern of ZmPP genes. To further expound the transcription regulation machineries of the 159 ZmPP genes in maize, a comprehensive promoter analysis was performed by PlantCARE database [67]. The occurrence of *cis*-elements in ZmPP genes are showed in Additional file 23. The statistical analysis showed that 12 *cis*-elements were enriched in maize PP genes, which were involved in regulation of gene expression under stress conditions. Nearly all genes (except *ZmPP13, -71, -125*) contained part of these 12 *cis*-elements, and the elements were variably present in the promoter regions of most ZmPP genes. Interestingly, we found that the majority of the ZmPP genes contained ABRE, G-Box and MBS, which are the TF binding sites for bZIP, GBF (G-Box-binding factor) and MYB, respectively. The data might indicate *cis*-elements in this study may play major roles in regulating the expression of PP genes in response to different stresses in maize.

MicroRNAs (miRNAs) play important roles in plant post-transcriptional gene regulation by either cleaving mRNA transcripts or repressing protein translation [68]. Putative miRNAs targeting the ZmPP genes were identified using Target-align program. It showed that about 35 ZmPP genes were targeted by maize miRNAs (Additional file 24). In addition to regulating growth and development, most miRNAs in plants play important roles in the regulation of various cellular processes underlying plant adaptation to environmental stresses. Among these target genes, 6 and 8 members belong to Group A PP2C and PP2A, respectively, indicating that miRNAs in the present study would assist in understanding the post-transcriptional control of gene regulation during physiological and stress-induced cellular responses.

## Conclusions

Conclusively, our results present a comprehensive account of PP-encoding genes and provide new insights into the phylogenetic relationships and characteristic functions of maize PPs. In addition, analyses of expression profiles based on microarray and RNA-seq method unraveled their probable functions during different developmental stages and abiotic stresses and will be useful in studies aimed at revealing the global regulatory network in maize development and abiotic stress responses, thereby contributing to the maize molecular breeding with enhanced quality traits.

## Electronic supplementary material

Additional file 1: Figure S1: Phylogenetic analysis of maize, rice, and Arabidopsis PP genes. An un-rooted NJ tree is made based on the catalytic domain sequences of maize, rice, and Arabidopsis PPs. PPs from Arabidopsis, rice, and maize belong to the same class falling in the same clades. Scale bar represents 0.1 amino acid substitutions per site. (PDF 1 MB)

Additional file 2: Figure S2: The map of intron/exon arrangement of ZmPP genes. (PDF 451 KB)

Additional file 3: Figure S3: Circos diagram of protein phosphatase gene pairs between maize and rice genomes. *Outer two circles* Distribution of each of the PP genes and scaled chromosomes for each species in million bp (Mb) units, respectively. *Histograms below each chromosome* Number of introns of PK genes: *green* < 5 introns, *red* ≥ 10 introns. *Boxes* Syntenic regions. Colors are assigned to the syntenic regions according to the colors of the corresponding chromosome. *Innermost colored lines* interconnect putative orthologous PP gene pairs between rice and maize. (PDF 497 KB)

Additional file 4: Figure S4: Heatmap showing the clustering of *ZmPPs* according to their expression profiles of 60 detected transcripts at different stages/organs of maize. Red, white and green indicate high, medium and low levels of gene expression, respectively. E2enzyme was used as a internal control. (PDF 508 KB)

Additional file 5: Figure S5: A putative schematic of root development signaling pathway in maize. The little colored blocks besides the gene expression level of pathway components under different developmental stages. (PDF 392 KB)

Additional file 6: Figure S6: RNA-seq differentially expressed analysis of ZmPP genes along maize leaf developmental gradients, namely, base, -1 cm, +4 cm, and tip. E2enzyme was used as a internal control. (PDF 388 KB)

Additional file 7: Figure S7: Expression patterns of ZmPP genes in 12 diverse maize reproductive tissues. DAP, days after pollination; Pre-em, preemergence; Post-em, postemergence. E2enzyme was used as a internal control. (PDF 417 KB)

Additional file 8: Figure S8: Expression profiles of signaling components under salt stress in CR, PR, and SR, respectively. Log2 based fold changes were used to create the heatmap. Expression values highlight with white mean only expressed in control or have no expression value both in control and salt stress treatment. (PDF 409 KB)

Additional file 9: Figure S9: The vector map of pEZS-NL-*ZmPP1*. (PDF 17 KB)

Additional file 10: Figure S10: Real-time PCR analysis of representative *ZmPPs* and MAPK-cascade genes under cold treatments. The maize ACTIN7 gene was used as endogenous control to normalize data. E2 enzyme was used as a internal control. (PDF 396 KB)

Additional file 11: Table S1: List of primers used in this study. (PDF 40 KB)

Additional file 12: Table S2: The identified maize protein phosphatases and their related information. (PDF 277 KB)

Additional file 13: Table S3: List of 152 detected ZmPP genes and their expression patterns at 18 selected tissues. (PDF 344 KB)

Additional file 14: Table S4: List of putative root development signaling components in maize. (PDF 116 KB)

Additional file 15: Table S5: List of flowering time signaling components in maize. (PDF 16 KB)

Additional file 16: Table S6: List of putative salt stress signaling components in maize. (PDF 341 KB)

Additional file 17: Table S7: List of expression values of MAPK cascade-related genes and key genes of ABA biosynthesis in maize under salt stress. (PDF 74 KB)

Additional file 18: Table S8: List of FPKM values of *ZmPPs* in maize reproductive (cob) and vegetative tissue (leaf) under both drought and well-watered conditions. MCC and MCD stand for maize ovary, well watered and drought, respectively; MLC and MLD stand for maize basal leaf meristem, well watered and drought, respectively. Numbers 1 and 2 indicate the two biological replicates. The extent of differential expression is measured in terms of fold change and (-) indicates failure to calculate or undetected values. Values in red and blue indicate the fold increase and decrease in expression in the drought-stressed tissue, respectively. (PDF 220 KB)

Additional file 19: Table S9: List of FPKM values of components involved in ABA biosynthesis and ABA-dependent pathway of response to drought in vegetative tissue (leaf) under both drought and well-watered conditions. MLC and MLD stand for maize basal leaf meristem, well watered and drought, respectively. Numbers 1 and 2 indicate the two biological replicates. The extent of differential expression is measured in terms of fold change and (-) indicates failure to calculate or undetected values. Values in red and blue indicate the fold increase and decrease in expression in the drought-stressed tissue, respectively. (PDF 265 KB)

Additional file 20: Table S10: List of putative components involved in ABA biosynthesis and ABA-dependent pathway of response to drought in maize. (PDF 270 KB)

Additional file 21: Table S11: List of expression values of ZmPP genes under cold stress. ETH-DH7 log(c), ETH-DH7 log(k), ETH-DL3 log(c), ETH-DL3 log(k) means intensity (AU) of fluorescence of labeled aaRNA from cold-treatment (c) or control (k) hybridizing to the probe, shown as log2 in the respective maize line. DH7 log(c/k), DL3 log(c/k) means log2 of ratio of expression in cold-treated vs. control of the probe in the respective maize line, respectively. Values in red and blue indicate the fold increase and decrease in expression in the drought-stressed tissue, respectively. (PDF 121 KB)

Additional file 22: Table S12: List of expression values of MAPK cascades genes in cold stress. ETH-DH7 log(c), ETH-DH7 log(k), ETH-DL3 log(c), ETH-DL3 log(k) means intensity (AU) of fluorescence of labeled aaRNA from cold-treatment (c) or control (k) hybridizing to the probe, shown as log2 in the respective maize line. ETH-DH7 log(c/k), ETH-DL3 log(c/k) means log2 of ratio of expression in cold-treated vs. control of the probe in the respective maize line, respectively. (PDF 84 KB)

Additional file 23: Table S13: List of promoters in ZmPP genes. (PDF 49 KB)

Additional file 24: Table S14: List of predicted miRNA-regulated ZmPP genes. (PDF 144 KB)
