# Supplementary figures and images for: Maize protein phosphatase gene family: identification and molecular characterization
Source: BMC Genomics. 2014 Sep 9;15(1):773. doi: 10.1186/1471-2164-15-773 (PMC4169795; doi:10.1186/1471-2164-15-773)

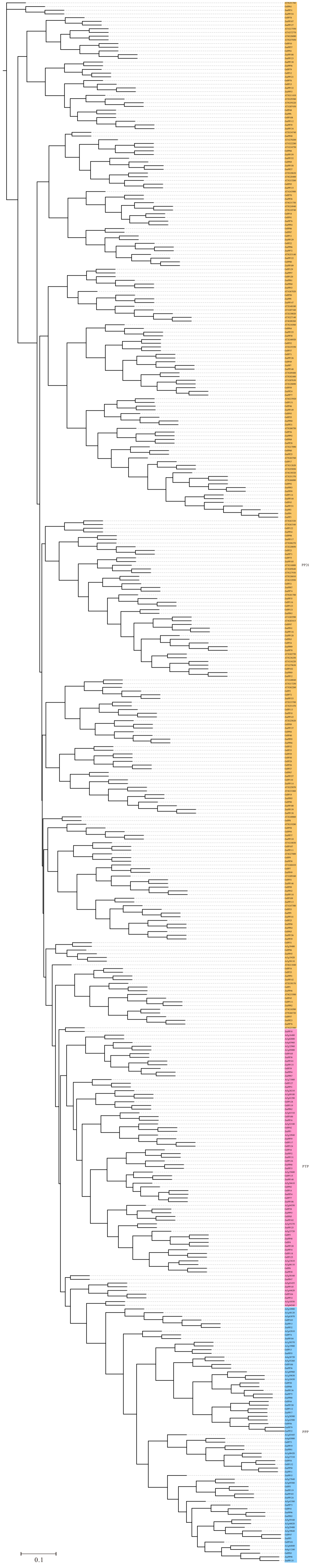

Supplement: Supplementary file 1 — Additional file 1: Figure S1: Phylogenetic analysis of maize, rice, and Arabidopsis PP genes. An un-rooted NJ tree is made based on the catalytic domain sequences of maize, rice, and Arabidopsis PPs. PPs from Arabidopsis, rice, and maize belong to the same class falling in the same clades. Scale bar represents 0.1 amino acid substitutions per site. (PDF 1 MB) [file 12864_2014_6458_MOESM1_ESM.pdf]

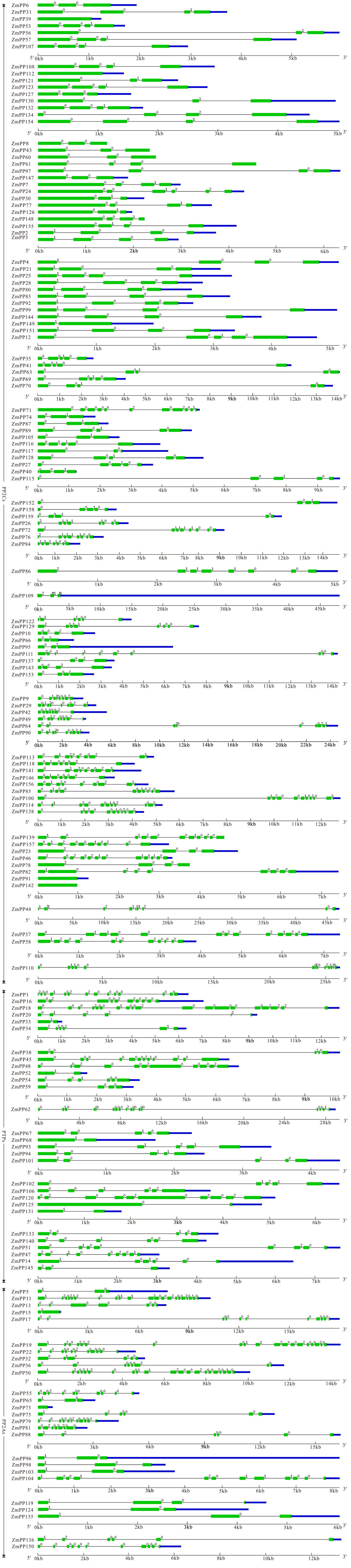

Supplement: Supplementary file 2 — Additional file 2: Figure S2: The map of intron/exon arrangement of ZmPP genes. (PDF 451 KB) [file 12864_2014_6458_MOESM2_ESM.pdf]

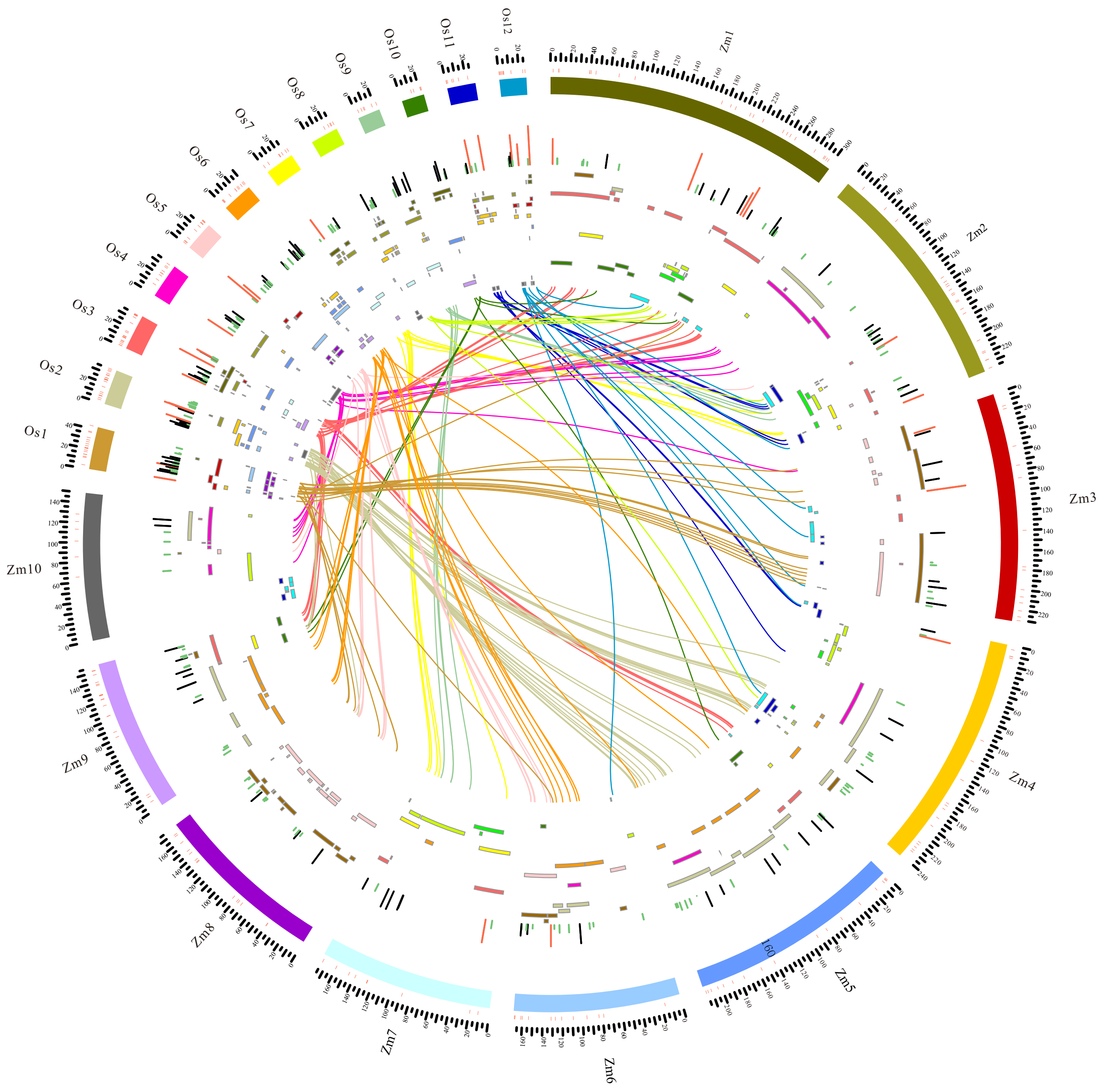

Supplement: Supplementary file 3 — Additional file 3: Figure S3: Circos diagram of protein phosphatase gene pairs between maize and rice genomes. Outer two circles Distribution of each of the PP genes and scaled chromosomes for each species in million bp (Mb) units, respectively. Histograms below each chromosome Number of introns of PK genes: green < 5 introns, red ≥ 10 introns. Boxes Syntenic regions. Colors are assigned to the syntenic regions according to the colors of the corresponding chromosome. Innermost colored lines interconnect putative orthologous PP gene pairs between rice and maize. (PDF 497 KB) [file 12864_2014_6458_MOESM3_ESM.pdf]

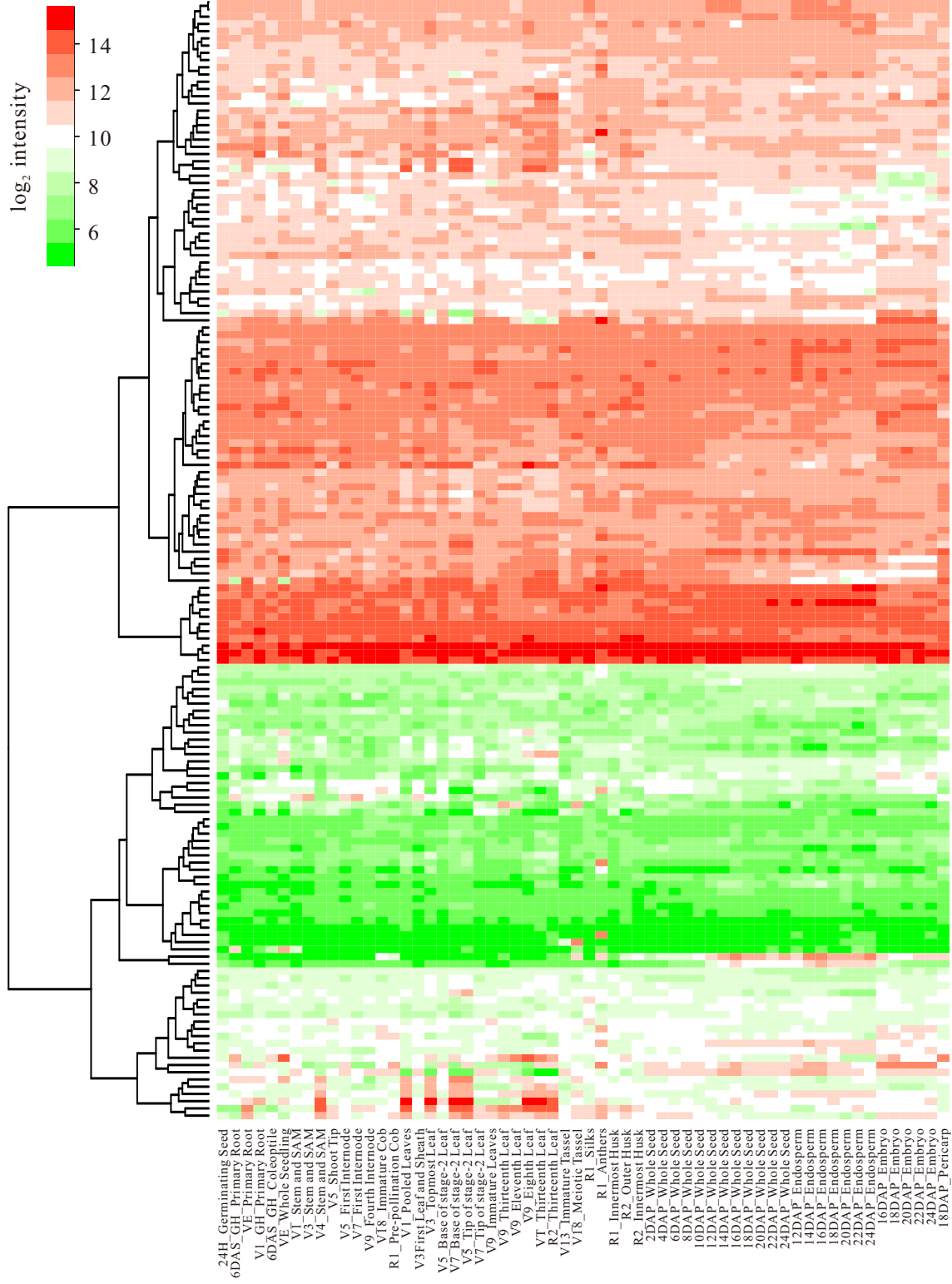

Supplement: Supplementary file 4 — Additional file 4: Figure S4: Heatmap showing the clustering of ZmPPs according to their expression profiles of 60 detected transcripts at different stages/organs of maize. Red, white and green indicate high, medium and low levels of gene expression, respectively. E2enzyme was used as a internal control. (PDF 508 KB) [file 12864_2014_6458_MOESM4_ESM.pdf]

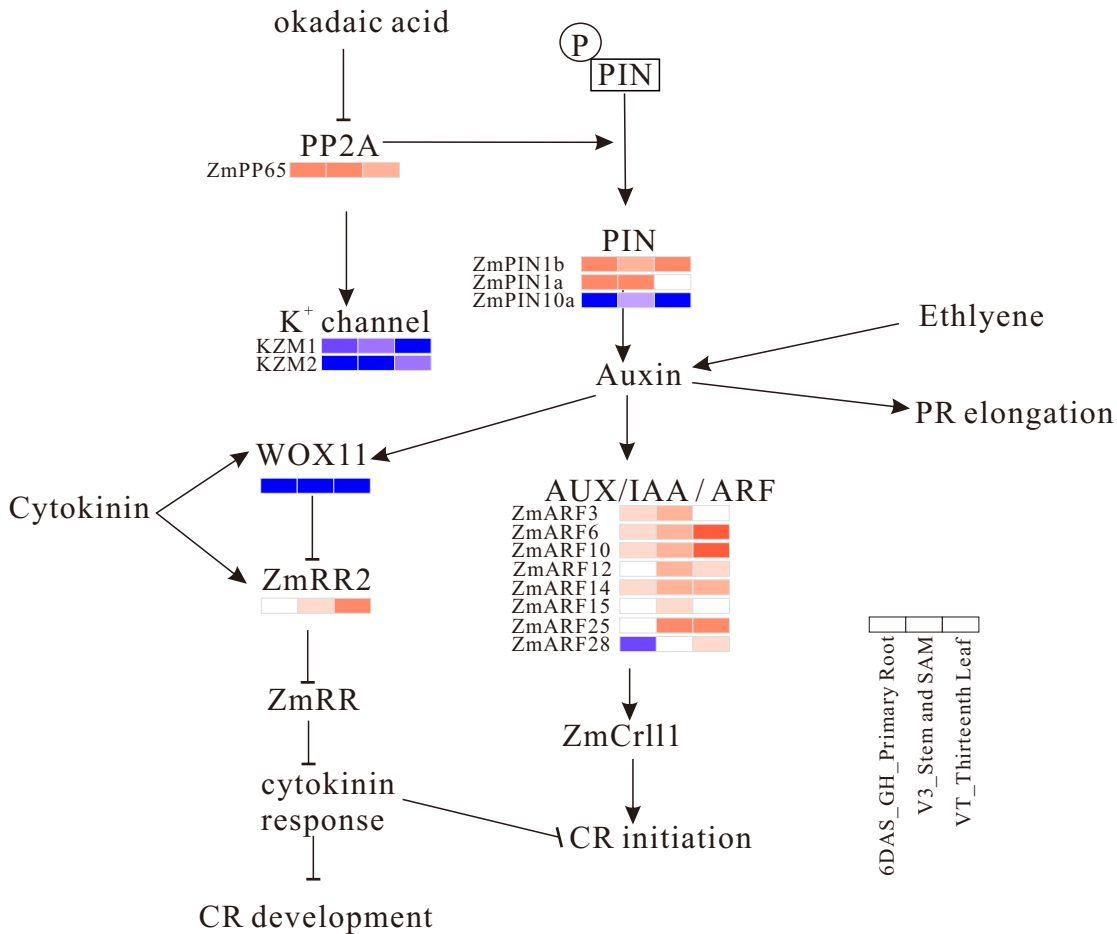

Supplement: Supplementary file 5 — Additional file 5: Figure S5: A putative schematic of root development signaling pathway in maize. The little colored blocks besides the gene expression level of pathway components under different developmental stages. (PDF 392 KB) [file 12864_2014_6458_MOESM5_ESM.pdf]

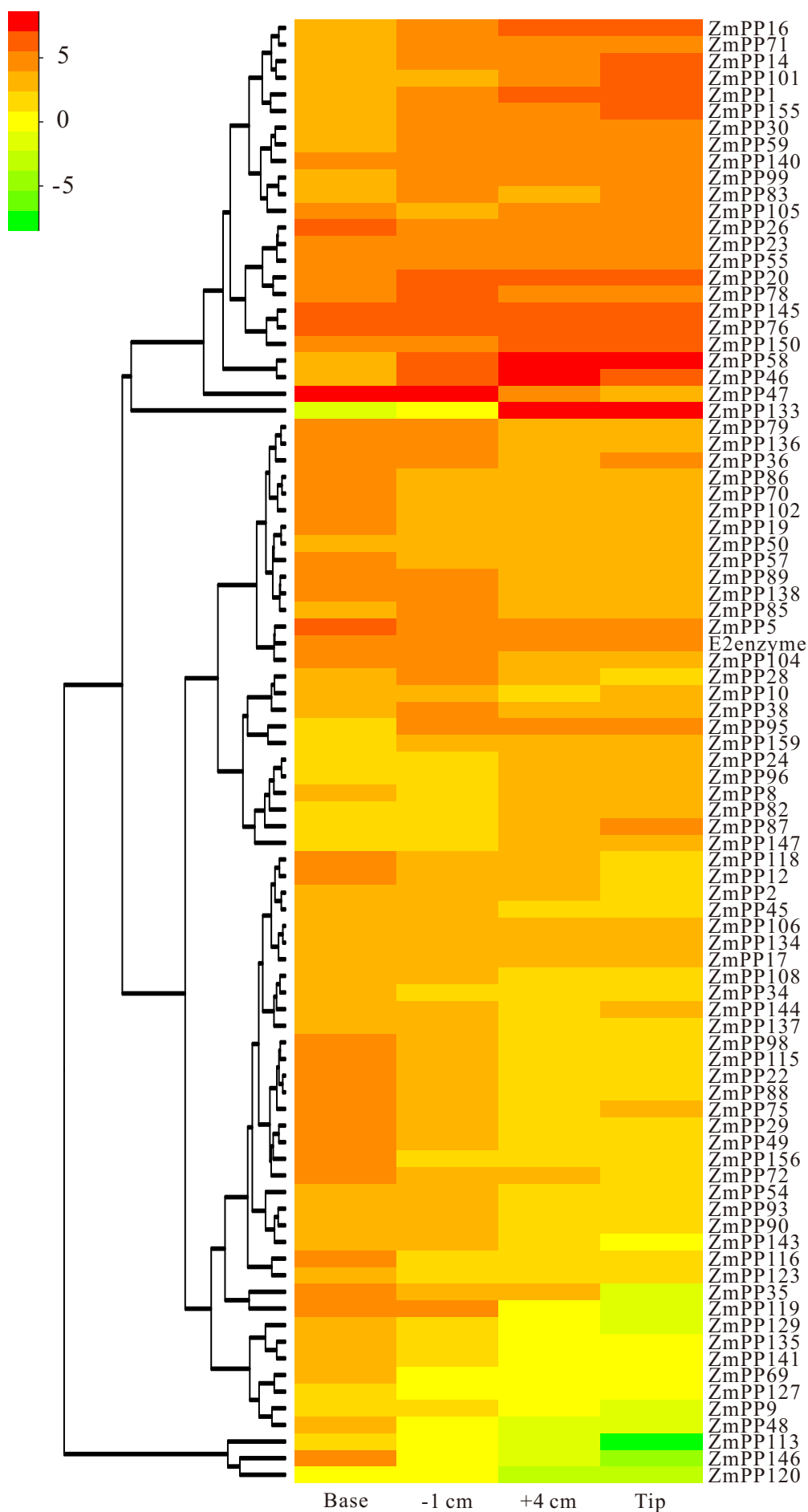

Supplement: Supplementary file 6 — Additional file 6: Figure S6: RNA-seq differentially expressed analysis of ZmPP genes along maize leaf developmental gradients, namely, base, -1 cm, +4 cm, and tip. E2enzyme was used as a internal control. (PDF 388 KB) [file 12864_2014_6458_MOESM6_ESM.pdf]

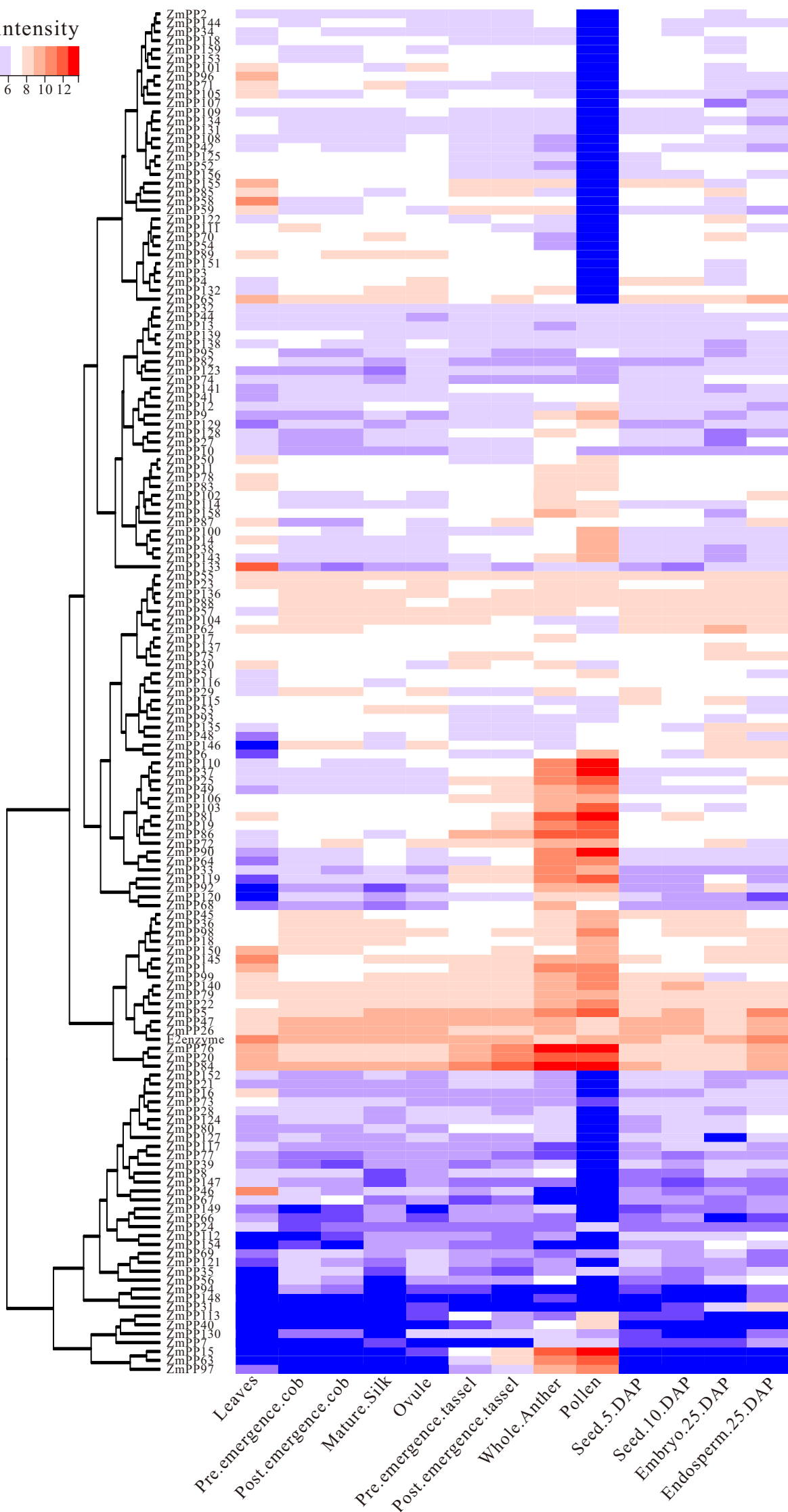

Supplement: Supplementary file 7 — Additional file 7: Figure S7: Expression patterns of ZmPP genes in 12 diverse maize reproductive tissues. DAP, days after pollination; Pre-em, preemergence; Post-em, postemergence. E2enzyme was used as a internal control. (PDF 417 KB) [file 12864_2014_6458_MOESM7_ESM.pdf]

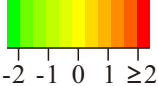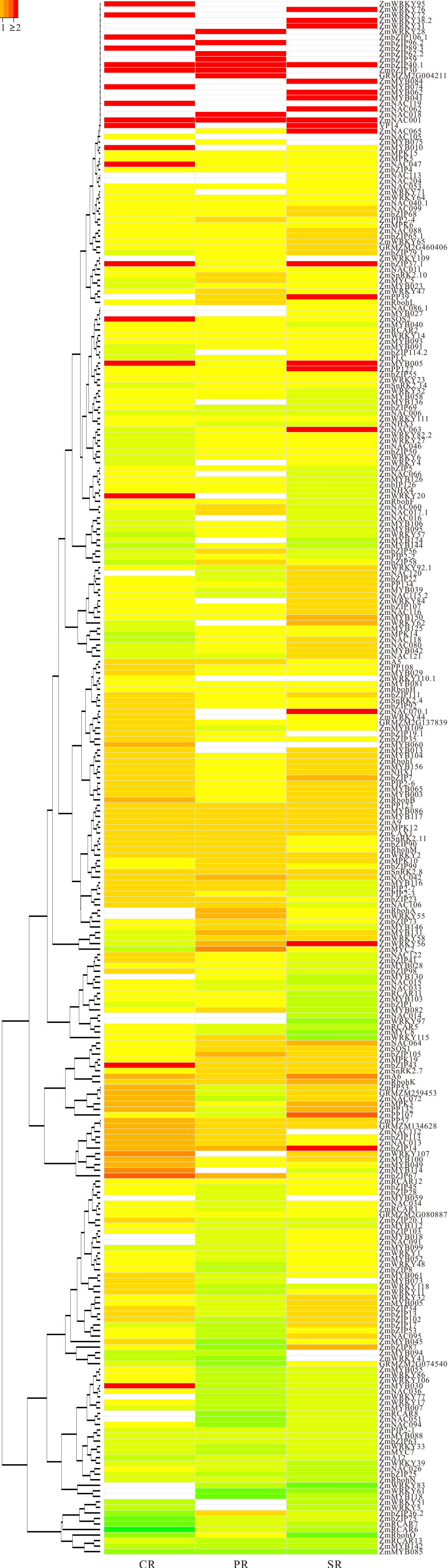

Supplement: Supplementary file 8 — Additional file 8: Figure S8: Expression profiles of signaling components under salt stress in CR, PR, and SR, respectively. Log2 based fold changes were used to create the heatmap. Expression values highlight with white mean only expressed in control or have no expression value both in control and salt stress treatment. (PDF 409 KB) [file 12864_2014_6458_MOESM8_ESM.pdf]

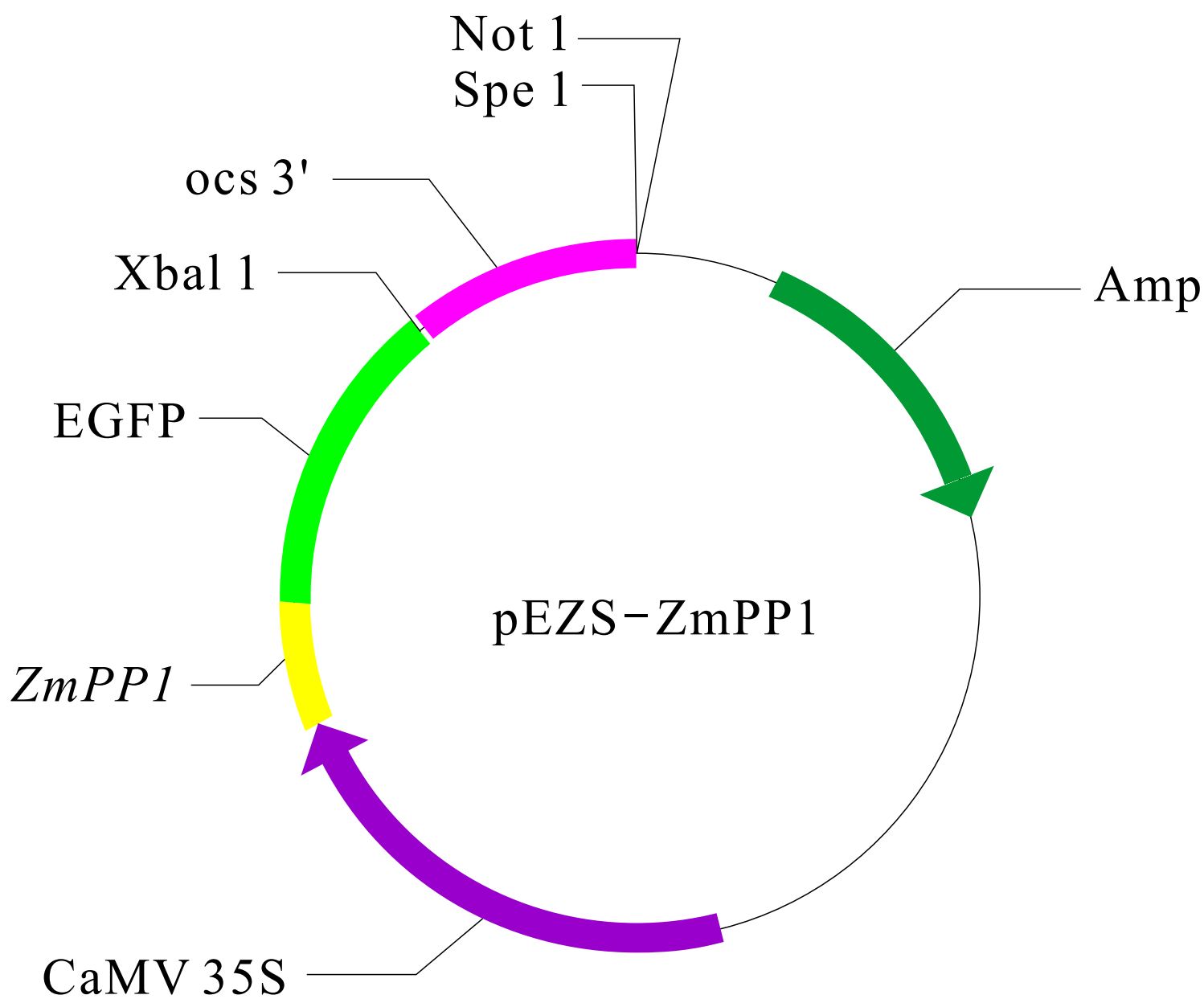

Supplement: Supplementary file 9 — Additional file 9: Figure S9: The vector map of pEZS-NL-ZmPP1. (PDF 17 KB) [file 12864_2014_6458_MOESM9_ESM.pdf]

control cold treatment

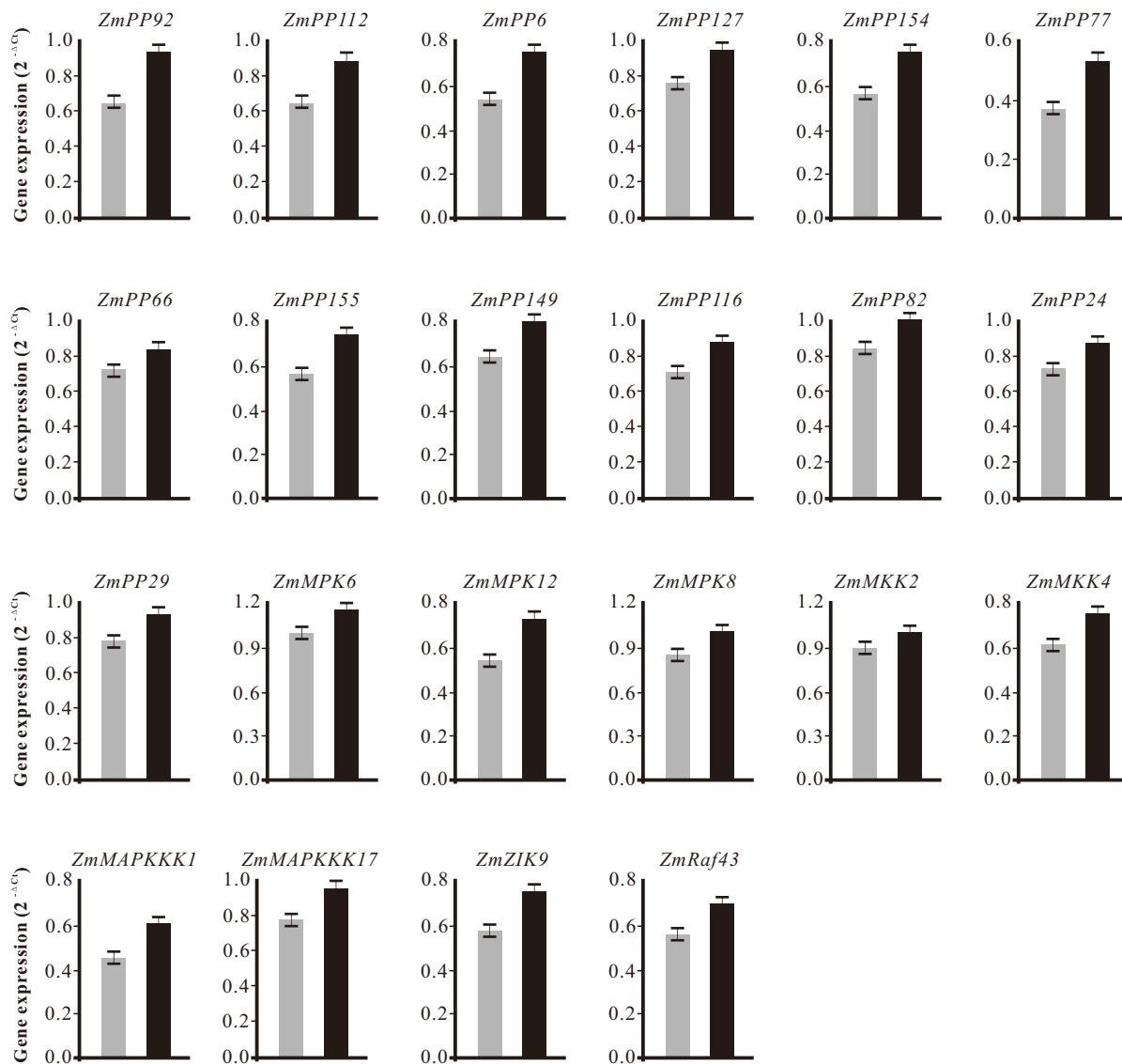

Supplement: Supplementary file 10 — Additional file 10: Figure S10: Real-time PCR analysis of representative ZmPPs and MAPK-cascade genes under cold treatments. The maize ACTIN7 gene was used as endogenous control to normalize data. E2 enzyme was used as a internal control. (PDF 396 KB) [file 12864_2014_6458_MOESM10_ESM.pdf]
