# Supplementary material for: Maize protein phosphatase gene family: identification and molecular characterization
Source: BMC Genomics. 2014 Sep 9;15(1):773. doi: 10.1186/1471-2164-15-773 (PMC4169795; doi:10.1186/1471-2164-15-773)
Supplement: Supplementary file 11 — Additional file 11: Table S1: List of primers used in this study. (PDF 40 KB) [file 12864_2014_6458_MOESM11_ESM.pdf]

Table S1 List of primers used in this study.

real-time PCR	Forward	Reverse
CO	5'-AGAACAACAGGGCACGAC-3'	5'-GTAGAATTGGAACTCGCTGATG-3'
FT	5'-CCCTGCTACAACTGGAACAACCT-3'	5'-CATCACCGTTCGTTACTCGTATCA-3'
UBQ1	5'-TCCAGGAAATGACAGGCCAT-3'	5'-GAGCACATGGGCAAGATCAG-3'
ACTIN7	5'-TCTGGCCTCTCTCAGTACCT-3'	5'-CTTCATGTGGACGATGCCTG-3'
ACTIN8	5'-TCAGCACTTTCCAGCAGATG-3'	5'-CTGTGGACAATGCCTGGAC-3'
ZmPLC	5'-TGAGTTGCCAGACCAAGGAA-3'	5'-CAAAGAAAGCCGCCTGACTT-3'
ZmPIP2-5	5'-GCTCGTCTACACCGTCTTCT-3'	5'-CACCCAGAAGATCCAGTGGT-3'
ZmSOS1	5'-GGGTTCTGGTTGCTGAAAGG-3'	5'-TGGCTTCCACCTGATACCTG-3'
ZmSOS2	5'-ATGAAGGTGCTCGACAAGGA-3'	5'-TCCTGGCTTCATTCTCACGT-3'
ZmA6	5'-CACCAACATGTACCCCTCCT-3'	5'-CTTCCTCTCCTGCAGCTTCT-3'
ZmNAC070.1	5'-GCTCAACTACTACCTCCGCA-3'	5'-CGTTGTAGATGGCCTTGTCG-3'
ZmMYC8	5'-GGTAGAGATCGACGCCAAGA-3'	5'-CTGCTGGATCATGAGGTCCT-3'
ZmMYB005	5'-GACAGTACCTCCAGAGCCTG-3'	5'-CTGTCCAACATCGCCATCAC-3'
VP14	5'-ACTTCTACTTCAGGCCCGAC-3'	5'-TCTTCTCCTTGTCCAGCACC-3'
ZmRbohO	5'-TTCCCATCCTTCTCTACGCC-3'	5'-GTAGATGTACTGCCCGCTCT-3'
ZmPP53	5'-CACAACTTCTTCGGCGTCTT-3'	5'-AGAAGCTCCTCTCCATCACG-3'
ZmPP132	5'-CGAGATGGAGGACACGGTC-3'	5'-CAATCTGCCTTCCTGTGCTC-3'
ZmWRKY92.1	5'-GACGACGTCCTTACCTTCCT-3'	5'-GCCCTCTCCACCTTCTTCTT-3'
ZmbZIP23	5'-GGAGTGGAGAAAGTGGTGGA-3'	5'-TCTGCCTGTTTCCTCTCCAG-3'
ZmSnRK2.7	5'-GAGAATGTCCAGCGCGAAAT-3'	5'-GGAAGAAGTAGCGAGCCTCA-3'
ZmRaf46	5'-TACTACCTCCAGCTGTCCCT-3'	5'-GGATGTTGTAGAACCCGTGC-3'
ZmPP1	5'-AACGAAACCGAATGCTGACG-3'	5'-CATCCGCGTACTGTTCCAAG-3'
ZmPP54	5'-GGCTGTACGAACACAACAGG-3'	5'-ACACCGCATCTTTCCTGGTA-3'
ZmPP59	5'-GGGTTTATGTCCACTGCACC-3'	5'-AACTTCCTCTCCCAGTCTGC-3'
ZmPP67	5'-CTTGACGTGAGAAACCAGCC-3'	5'-TCGAACAGCTCCATGAACCT-3'
ZmPP125	5'-CGGATTCAAGTGTCAGCTCG-3'	5'-GTGTGAAGCCTCCTCTCCTT-3'
ZmPP133	5'-CCCAAGAAACCAACCTCTGC-3'	5'-TCGAACAGCTCCATGAACCT-3'
ZmPP101	5'-TCGAGAAGAACGGGATCAGG-3'	5'-TTCGGTGCTTGCCTCTCTTA-3'
ZmPP68	5'-CTAGCCACCGGTATTGTTGC-3'	5'-TCAAGAGAAACGGCCATCCT-3'
ZmPP8	5'-GGGAAGGAGAAGGGTGGAAA-3'	5'-TTCGATCTCTCCGCCGTTTA-3'
ZmPP112	5'-GAGGAGCACAGCGTCATTG-3'	5'-ACACGTACGGCTTCAGGTAT-3'
ZmPP76	5'-AATCAAGGAGGAGGTGGTCG-3'	5'-GTCGTCGCCTGATTTCTCAC-3'
ZmPP49	5'-GCATTATCCTCAGAAGCCGC-3'	5'-AGACTTCAGCTTGCTCGTCT-3'
ZmPP138	5'-GGATGAAATGATGCGGGGTC-3'	5'-ACACATGCTGTACTCCCACA-3'
ZmPP82	5'-CAGTTGATGTGAGTGGTGGC-3'	5'-AAAGCCGAACTCAGCTCTCT-3'
ZmPP17	5'-GGCAGTGCAAATGTCTGGAA-3'	5'-CCCAGCCACATCTATCGTCT-3'
ZmPP65	5'-TGCATGGTGGTCTATCTCCG-3'	5'-CACGATCATTGATGGCCCAG-3'
ZmPP79	5'-ACTGCATTGGTCGAGTCAGA-3'	5'-AGGAGAAATACCCCAGCCAC-3'
ZmPP154	5'-CTCCGACCACAAGCCAAATC-3'	5'-ATGTCGTTGCTGATCACGTC-3'
ZmPP29	5'-TCCCCTTAGATGTTGGCCAG-3'	5'-AAAGCATCCCAAACACCGTC-3'
ZmPP113	5'-TTTTGCACCCTCCAGTTGTG-3'	5'-GCACATGTGACCAAAGACGT-3'
ZmPP146	5'-CGGCTAGAGGAGAATGCAGA-3'	5'-GGTACAATGAACTCGCCGAC-3'
ZmPP7	5'-GAACAGCAATGTGGAGTGGG-3'	5'-AAGCCTTTGAGCTCGTTGTG-3'
ZmPP105	5'-AGGTGACGCAGAAGAGGATC-3'	5'-ATATGTACCGCCTCGTCGTT-3'
ZmPP40	5'-GTCTCGTGTTCTTCGTGTCC-3'	5'-AACCCGTACGTCACCTTCC-3'
ZmPP92	5'-GGCGGGAATTTGCTCTTGAT-3'	5'-GATCGTGGATTGCTGTGGAC-3'
ZmPP6	5'-GAGAAGAGCTACGCGAGGAT-3'	5'-TTGTGGTCAGCTGAGAGAGG-3'
ZmPP127	5'-GAGCCCTTAGAGCTGTCGAT-3'	5'-GGGGAAAAGGTCATTCTGCC-3'
ZmPP77	5'-ATGTTCATCGCAACAACCCC-3'	5'-CAGATCCTGCCCTCCAAAGA-3'
ZmPP66	5'-CAACTTCGTCCCGTTCATCC-3'	5'-GTTATGGCCATCGAACACCC-3'
ZmPP155	5'-GACTCGTTCTCCACCTCACA-3'	5'-CTATCCTCTCCAGCAGCTCC-3'
ZmPP149	5'-CTTCATCATCTTCGCGTCGG-3'	5'-TCTCGATGGTCCGGATCTTG-3'
ZmPP116	5'-CTTGCATCAGATGGGGTGTG-3'	5'-CTTCGTCGAGGCTCATGTTG-3'
ZmPP82	5'-CAGTTGATGTGAGTGGTGGC-3'	5'-AAAGCCGAACTCAGCTCTCT-3'
ZmPP24	5'-CGAACAAAGCAGGGATGGAC-3'	5'-GATGCGCTTTTGGTTTGGTG-3'

ZmPP29	5'-TCCCCTTAGATGTTGGCCAG-3'	5'-AAAGCATCCCAAACACCGTC-3'
ZmMPK6	5'-TCAAGGTGATGTTGCAAGGC-3'	5'-TCTCACTCTTGCAGGTCTGG-3'
ZmMPK12	5'-CCAAGGTTGAGAGAGAGCCA-3'	5'-ATCACAGGTCCGTTTCCACT-3'
ZmMPK8	5'-CAACGCAAACTGTGACCTGA-3'	5'-AATATGCACCCCACAGACCA-3'
ZmMKK2	5'-GCGCGTTATACACCGAGATC-3'	5'-GATTCTTTCTGGCGCCATGT-3'
ZmMKK4	5'-CCTCAGCATCCTCGAGTTCT-3'	5'-TCACCGGGTTCTTCTGTAGG-3'
ZmMAPKKK1	5'-ATCATGGCGACGGGTTAGAA-3'	5'-GCCTACCTTTGCTAACGTCG-3'
ZmMAPKKK17	5'-CCTAGTTGATGCAAGCGGAC-3'	5'-TGTGCAGCCAAGACTCCATA-3'
ZmZIK9	5'-CTCAACTGCAGCAACGTCTT-3'	5'-GCCGTACGAGTAGATGTCCA-3'
ZmRaf43	5'-TGATAGCACCCAGGAAAGCA-3'	5'-ATCCGAGCCATACCACTGAG-3'
**PCR primer**	**Forward**	**Reverse**
FPTP1	5'-GCGGATCCAATGGGCAACGGTATGG-3'	5'-ATCCATGGGCTTCATCTCTGAATTGGGA-3'
ZEP	5'-ATTTGACGGTTGGTGCGACA-3'	5'-CTCTCATTCTTCCTCGTCGATTT-3'
NCED3	5'-CAACGGAGCTAACCCACTTCA-3'	5'-ACCCTATCACGACGACTTCATCT-3'
SDR1	5'-AGGGATAGGTGAGAGCATTGTTC-3'	5'-CGCTACATCATCAACCGTCAGT-3'
AAO3	5'-GCTTCCTGGCATTTGGTTCTTAT-3'	5'-TCTTTCGCTCAGCTTCTTCC-3'
ABA3	5'-ACCAGTGACCTTATAGCGGATGC-3'	5'-CGTTGGGCCTGATTTATGTGAA-3'
ACT2	5'-CTTCCCTCAGCACATTCCAG-3'	5'-CCCAGCTTTTTAAGCCTTTG-3'
ZmPP1	5'-CTCGAGATGAACTGCCTCCAGAACCTGCTCAAG-3'	5'-GGTACCTTGCGCTCGGCGGCATCCGCGTAC-3'
