# Supplementary material for: Maize protein phosphatase gene family: identification and molecular characterization
Source: BMC Genomics. 2014 Sep 9;15(1):773. doi: 10.1186/1471-2164-15-773 (PMC4169795; doi:10.1186/1471-2164-15-773)
Supplement: Supplementary file 12 — Additional file 12: Table S2: The identified maize protein phosphatases and their related information. (PDF 277 KB) [file 12864_2014_6458_MOESM12_ESM.pdf]

Table S2. The identified maize protein phosphatases and their related information.

[1]Isoelectric Point of ZmPPs.

[2]Molecular Weight of ZmPPs.

[3]Predicted subcellular localization of ZmPPs. "nuclear", "cytosol","chloroplast", "mitochondrion", "cytoskeleton", "Vacuolar membrane", "plastid", and "endoplasmic reticulum" are represented as "nucl", "cyto", "chlo", "mito", "cysk", "vacu", "plas" and "E.R.", respectively.

Name	Gene ID	Protein ID	Group	Chr.	W/C	Locus	No. introns in ORF	Full length	Position of catalytic domain	pI[1]	Mw[2]	At Ortholog	Rice Ortholog	Subcellular location[3]
**PP2C**														
ZmPP6	GRMZM2G010855	GRMZM2G010855_P01	A	1	W	39139491..39141597	2	396	33-329	6.06	42032.99	AT1G07430.1[1]	Os03g16170.1	nucl
ZmPP31	GRMZM2G019819	GRMZM2G019819_P01	A	2	C	103045720..103049593	3	452	127-449	5.63	48351.51	AT2G29380.1	Os05g38290.2	nucl
ZmPP39	GRMZM2G082487	GRMZM2G082487_P01	A	2	W	175540392..175541806	0	350	81-346	6.34	36961.56	AT1G07430.1	Os09g15670.1	nucl
ZmPP53	GRMZM2G059453	GRMZM2G059453_P01	A	3	W	180041545..180043505	3	408	105-405	5.74	43279.76	AT1G07430.1	Os01g62760.1	nucl
ZmPP56	GRMZM2G122228	GRMZM2G122228_P01	A	3	W	212832371..212838370	2	410	121-397	5.81	43434.87	AT2G29380.1	Os01g46760.1	nucl
ZmPP57	GRMZM2G134628	GRMZM2G134628_P01	A	3	C	220844236..220850001	3	484	160-475	4.74	50696.61	AT1G17550.1	Os01g40094.1	cyto
ZmPP107	GRMZM2G177386	GRMZM2G177386_P02	A	6	W	161548061..161551449	3	370	54-361	5.26	39716.06	AT1G72770.3	Os05g46040.1	cyto
ZmPP108	GRMZM2G102255	GRMZM2G102255_P01	A	6	C	167404554..167407544	3	459	148-450	5.42	49072.28	AT1G17550.1	Os05g51510.1	chlo
ZmPP112	GRMZM2G308615	GRMZM2G308615_P01	A	7	W	89034033..89035626	0	358	81-354	6.38	37599.2	AT2G29380.1	Os09g15670.1[2]	nucl
ZmPP121	GRMZM5G818101	GRMZM5G818101_P02	A	8	W	72623215..72625583	2	375	87-365	7.02	39966.71	AT2G29380.1	Os05g49730.1	nucl
ZmPP123	GRMZM2G383807	GRMZM2G383807_P01	A	8	C	77983607..77986678	3	423	112-414	5.02	44895.58	AT1G17550.1	Os05g51510.1	chlo
ZmPP127	GRMZM2G001243	GRMZM2G001243_P01	A	8	C	136017188..136018955	2	282	24-245	5.36	30202.34	AT1G72770.2	Os05g46040.2	cyto
ZmPP130	GRMZM2G300125	GRMZM2G300125_P01	A	8	C	147912922..147918179	2	394	109-379	6.19	41708.93	AT2G29380.1	Os01g46760.1	cyto
ZmPP132	GRMZM2G166297	GRMZM2G166297_P01	A	8	W	168479978..168481963	3	413	113-410	6.34	43820.41	AT1G07430.1	Os01g62760.1	nucl
ZmPP134	GRMZM2G149132	GRMZM2G149132_P01	A	9	C	14014949..14019990	3	354	64-320	5.05	39029.13	AT2G25620.1	Os06g08140.1	cyto
ZmPP154	GRMZM2G159811	GRMZM2G159811_P01	A	10	C	101360842..101366143	3	464	143-461	5.39	48826.87	AT2G29380.1	Os03g16170.1	nucl
ZmPP8[3]	AC208201.3_FG002	AC208201.3_FGP002	B	1	C	45366147..45367585	2	388	141-383	5.97	40789.15	AT1G07160.1	Os03g18150.1	chlo
ZmPP43	GRMZM2G342197	GRMZM2G342197_P01	B	2	C	219328554..219330884	2	429	174-425	6.96	44565.81	AT1G07160.1	Os11g13820.1	chlo
ZmPP60	GRMZM2G407605	GRMZM2G407605_P01	B	4	C	10795891..10798358	2	436	184-429	6.27	45449.71	AT2G30020.1	Os11g13820.1	chlo
ZmPP61	GRMZM2G407623	GRMZM2G407623_P01	B	4	C	10803889..10808446	3	465	178-461	6.28	48770.04	AT2G40180.1	Os12g09640.1	chlo
ZmPP97	GRMZM2G000603	GRMZM2G000603_P01	B	6	C	16792554..16799019	3	431	156-428	7.12	45113.36	AT2G30020.1	Os12g09640.1	chlo
ZmPP147	GRMZM2G465287	GRMZM2G465287_P01	B	9	W	137595003..137596967	2	421	173-416	7.04	44750.78	AT2G30020.1	Os03g18150.1	chlo
ZmPP7	GRMZM2G112925	GRMZM2G112925_P01	C	1	C	40511639..40514803	3	665	239-585	6.43	72070.41	AT1G07630.1	Os03g16760.1	nucl
ZmPP24	GRMZM2G093776	GRMZM2G093776_P01	C	1	W	293454216..293458523	5	669	227-607	6.24	72213.6	AT2G28890.1	Os03g60650.1	chlo
ZmPP30	GRMZM2G016749	GRMZM2G016749_P01	C	2	C	60629665..60634125	2	521	172-509	4.86	57469.74	AT3G16560.1	Os04g33080.3	chlo
ZmPP77	GRMZM2G144109	GRMZM2G144109_P01	C	5	C	2315004..2318785	3	632	236-624	5.41	68202.56	AT1G07630.1	Os03g60650.1[4]	chlo
ZmPP126	GRMZM2G141859	GRMZM2G141859_P01	C	8	W	133946985..133949080	2	550	181-536	5.93	59087.08	AT2G28890.1	Os05g02110.1	chlo
ZmPP148	GRMZM2G103247	GRMZM2G103247_P01	C	9	W	139705351..139707757	3	614	234-606	5.86	66121.83	AT2G28890.1	Os03g16760.1	cyto
ZmPP155	GRMZM2G443509	GRMZM2G443509_P01	C	10	W	113162222..113169068	3	616	172-509	5.25	68088.38	AT3G16560.1	Os04g33080.3	chlo
ZmPP2	AC155624.2_FG006	AC155624.2_FGP006	D	1	C	8264824..8269232	3	357	57-357	7.25	39941.37	AT3G51370.1	Os03g04430.1	nucl
ZmPP3	GRMZM5G833774	GRMZM5G833774_P01	D	1	W	8271537..8275361	3	399	57-357	8.49	44151.12	AT3G51370.1	Os03g04430.1	nucl
ZmPP4	GRMZM5G891266	GRMZM5G891266_P01	D	1	C	8283445..8289244	3	399	57-362	8.69	44060.99	AT3G51370.1	Os03g04430.1	nucl
ZmPP21	GRMZM2G050512	GRMZM2G050512_P01	D	1	W	279119397..279122777	3	376	44-338	9.39	41018.53	AT4G33920.1	Os03g55320.1	chlo
ZmPP25	GRMZM2G156543	GRMZM2G156543_P01	D	1	W	295487761..295492645	3	379	64-357	6.36	41043.52	AT3G17090.1	Os03g61690.1	cyto
ZmPP28	GRMZM2G479665	GRMZM2G479665_P01	D	2	C	14613507..14616809	3	392	64-355	6.18	43680.29	AT5G06750.3	Os04g49490.2	cyto

ZmPP80	GRMZM2G377904	GRMZM2G377904_P01	D	5	C	6436446..6439210	3	367	45-337	9.44	40083.56	AT4G33920.1	Os03g55320.1	chlo
ZmPP83	GRMZM2G069970	GRMZM2G069970_P01	D	5	W	61837656..61842314	3	394	56-356	8.94	43795.1	AT3G51370.1	Os06g50380.2	mito
ZmPP92	GRMZM2G057907	GRMZM2G057907_P01	D	5	W	197686662..197689690	3	387	61-352	8.87	43102	AT5G06750.3	Os02g46080.2	mito
ZmPP99	GRMZM2G151254	GRMZM2G151254_P01	D	6	C	83605222..83611396	3	390	56-356	9.03	43143.32	AT3G51370.1	Os06g50380.2	chlo
ZmPP144	GRMZM2G108309	GRMZM2G108309_P01	D	9	C	126336491..126340954	3	398	57-357	8.51	44107.02	AT4G38520.2	Os10g39780.1	chlo
ZmPP149	GRMZM2G130943	GRMZM2G130943_P02	D	9	W	147545345..147547482	1	391	53-348	8.03	42142.89	AT4G33920.1	Os03g10950.1	cyto
ZmPP151	GRMZM2G010017	GRMZM2G010017_P01	D	9	C	151815439..151819668	3	399	57-357	8.69	44145.05	AT4G38520.2	Os03g04430.1	chlo
ZmPP12	GRMZM2G170299	GRMZM2G170299_P01	E	1	W	184118323..184123276	4	515	49-381	5.27	55043.58	AT1G16220.1	Os08g39100.1	nucl
ZmPP35	GRMZM2G153675	GRMZM2G153675_P01	E	2	W	152500883..152503674	4	473	71-381	5.17	51015	AT1G79630.2	Os11g01790.1	cyto
ZmPP41	GRMZM2G021610	GRMZM2G021610_P01	E	2	W	205787043..205799282	4	432	67-330	5.54	46245.04	AT4G03415.2	Os07g37890.1	chlo
ZmPP63	GRMZM2G120246	GRMZM2G120246_P01	E	4	C	99604469..99618606	4	458	99-381	6.24	49964.5	AT3G02750.2	Os11g22404.1	nucl
ZmPP69	GRMZM2G071196	GRMZM2G071196_P01	E	4	W	198325575..198329863	4	523	47-384	5.12	56366.12	AT1G16220.1	Os08g39100.1	chlo
ZmPP70	GRMZM2G067910	GRMZM2G067910_P01	E	4	C	218666642..218680650	4	500	68-453	8.04	53199.1	AT3G02750.2	Os02g27220.1	chlo
ZmPP71	GRMZM2G053722	GRMZM2G053722_P01	E	4	W	221295031..221301313	10	813	115-396	5.03	90157.14	AT2G20050.1	Os02g17970.2	nucl
ZmPP74	GRMZM2G040452	GRMZM2G040452_P03	E	4	W	229537254..229540046	2	385	76-365	6.32	40996.28	AT5G26010.1	Os02g13100.1	chlo
ZmPP87	GRMZM2G119623	GRMZM2G119623_P01	E	5	W	153945630..153948183	2	377	70-358	5.25	40599.54	AT5G36250.1	Os02g13100.1	chlo
ZmPP89	GRMZM2G102560	GRMZM2G102560_P02	E	5	W	159957620..159962813	4	526	149-455	6.65	55797.08	AT3G02750.2	Os02g27220.1	chlo
ZmPP105	GRMZM2G069713	GRMZM2G069713_P01	E	6	C	138870557..138873663	2	380	50-336	9.09	41413.8	AT3G05640.2	Os05g29030.1	chlo
ZmPP116	GRMZM2G107565	GRMZM2G107565_P01	E	7	W	158156507..158160970	4	431	77-335	5.72	46356.2	AT1G03590.1	Os07g37890.1	chlo
ZmPP117	GRMZM2G161544	GRMZM2G161544_P01	E	7	W	170365708..170370184	10	352	67-332	5	37237.76	AT3G06270.1	Os07g45170.1	cyto
ZmPP128	GRMZM2G136765	GRMZM2G136765_P01	E	8	C	138556689..138562724	4	473	133-436	8.9	50377.87	AT1G16220.1	Os02g27220.1	chlo
ZmPP27	GRMZM2G111232	GRMZM2G111232_P01	F1	2	C	11050697..11054729	4	315	75-314	8.72	34151.75	AT4G28400.1	Os04g52000.1	chlo
ZmPP40	GRMZM2G311187	GRMZM2G311187_P01	F1	2	W	203085227..203086469	3	325	73-325	6.28	35363.59	AT3G15260.2	Os07g32380.1	mito
ZmPP115	GRMZM2G010298	GRMZM2G010298_P01	F1	7	W	151508928..151519024	4	290	49-389	6.27	31734.98	AT3G15260.2	Os07g32380.1	cyto
ZmPP152	GRMZM2G074489	GRMZM2G074489_P02	F1	10	W	65474354..65488822	3	336	85-312	5.94	36524.49	AT4G28400.1	Os04g52000.1	chlo
ZmPP158	GRMZM2G155991	GRMZM2G155991_P01	F1	10	W	141619078..141623273	4	318	78-317	8.73	34552.13	AT4G28400.1	Os04g52000.1	chlo
ZmPP159[5]	GRMZM2G180471	GRMZM2G180471_P01	F1	10	W	143662996..143675455	4	284	46-283	5.83	31193.5	AT1G34750.1	Os04g37904.1	cyto
ZmPP26	GRMZM2G056572	GRMZM2G056572_P02	F2	2	C	3969379..3973803	7	284	39-375	4.7	30659.07	AT4G31750.1	Os04g56450.1	cyto
ZmPP72	GRMZM2G073788	GRMZM2G073788_P01	F2	4	C	225064123..225073527	7	357	115-352	5.7	38543.2	AT4G31750.1	Os02g05630.1	chlo
ZmPP76[6]	GRMZM2G166035	GRMZM2G166035_P01	F2	4	W	237508371..237511930	7	290	33-270	4.76	31207.52	AT4G31750.1	Os02g05630.2	cysk
ZmPP84	GRMZM5G829894	GRMZM5G829894_P01	F2	5	C	80387639..80389787	6	255	1-235	4.7	27749.93	AT5G53140.1	Os02g15594.1	cyto
ZmPP86	GRMZM2G442404	GRMZM2G442404_P01	F2	5	W	141575880..141581134	7	365	123-360	5.79	39295.97	AT5G53140.1	Os02g15594.1	chlo
ZmPP109	GRMZM2G412937	GRMZM2G412937_P01	F2	6	C	168009624..168067650	6	499	245-482	4.77	54300.21	AT5G53140.1	Os05g50970.1	cyto
ZmPP122	GRMZM2G158818	GRMZM2G158818_P01	F2	8	W	76319202..76324406	5	208	36-192	5.02	22719.37	AT5G53140.1	Os05g50970.1	cyto
ZmPP129	GRMZM2G040642	GRMZM2G040642_P01	F2	8	C	144367294..144375321	7	367	115-352	6.38	39586.46	AT5G53140.1	Os05g50970.1	chlo
ZmPP10	GRMZM2G057768	GRMZM2G057768_P02	G	1	W	84671696..84674989	3	375	89-341	6.07	40359.67	AT2G33700.1	Os10g39540.1	nucl
ZmPP66	GRMZM2G108147	GRMZM2G108147_P02	G	4	W	173563703..173565614	2	245	24-245	5.18	26691.93	AT2G25620.1	Os02g55560.1	cyto
ZmPP95	GRMZM2G180430	GRMZM2G180430_P01	G	5	C	214092148..214098979	2	357	66-322	5.28	39691.75	AT2G25620.1	Os02g55560.1	chlo
ZmPP111	GRMZM2G126832	GRMZM2G126832_P02	G	7	W	86047473..86062099	7	360	94-359	6.57	39023.96	AT1G18030.1	Os09g14540.1	nucl
ZmPP137	GRMZM2G158734	GRMZM2G158734_P01	G	9	W	87131326..87135073	3	360	70-325	5.09	39093.22	AT2G25620.1	Os06g39600.1	cyto
ZmPP143	GRMZM2G009163	GRMZM2G009163_P01	G	9	C	125898278..125902397	3	372	86-338	6.4	40291.82	AT2G33700.1	Os02g17970.2	cyto
ZmPP153	GRMZM2G060798	GRMZM2G060798_P01	G	10	C	85581746..85584681	3	405	98-360	4.82	44303.09	AT3G62260.2	Os05g04360.1	chlo
ZmPP9	GRMZM2G077960	GRMZM2G077960_P01	H	1	C	68554980..68559182	7	438	40-304	5.43	47270.86	AT1G47380.1	Os03g27780.2	chlo
ZmPP29	GRMZM2G015610	GRMZM2G015610_P01	H	2	C	44386603..44391746	8	444	48-303	5.8	47357.53	AT1G47380.1	Os04g37660.1	mito
ZmPP42	GRMZM5G836628	GRMZM5G836628_P02	H	2	C	217972747..217978479	6	429	42-298	8.15	46098.7	AT1G09160.1	Os07g49040.1	chlo

ZmPP49	GRMZM2G173734	GRMZM2G173734_P01	H	3	W	75132287..75136332	7	433	93-355	7.61	46025.41	AT1G68410.2[7]	Os01g32964.1	chlo
ZmPP64	GRMZM2G081359	GRMZM2G081359_P03	H	4	W	122320874..122345693	8	449	55-310	6.07	48214.45	AT1G47380.1	Os02g35910.1	vacu
ZmPP90	GRMZM2G052699	GRMZM2G052699_P01	H	5	W	175461538..175465986	8	446	55-315	5.79	47985.08	AT1G47380.1	Os02g35910.1	chlo
ZmPP113	GRMZM2G135444	GRMZM2G135444_P01	H	7	W	126349583..126354736	8	431	38-295	6.06	47157.9	AT1G47380.1	Os09g28560.1	chlo
ZmPP118	GRMZM2G113016	GRMZM2G113016_P01	H	7	C	174530527..174534871	6	429	42-298	7.51	46024.57	AT1G09160.1	Os07g49040.1	chlo
ZmPP141	GRMZM2G150213	GRMZM2G150213_P01	H	9	C	121712179..121717046	8	446	40-310	5.38	47991.53	AT1G47380.1	Os03g27780.1	chlo
ZmPP146	GRMZM2G006429	GRMZM2G006429_P01	H	9	C	136552200..136555676	6	430	42-298	6.53	45911.18	AT1G09160.1	Os03g18970.1	nucl
ZmPP156	GRMZM2G044382	GRMZM2G044382_P01	H	10	W	120823846..120828918	8	443	47-302	5.8	47533.75	AT1G47380.1	Os04g37660.1	chlo
ZmPP85	GRMZM2G360455	GRMZM2G360455_P02	I	5	C	91807061..91814398	9	359	33-330	5.22	39222.09	AT4G31860.1	Os02g08364.1	nucl
ZmPP100	GRMZM2G019812	GRMZM2G019812_P01	I	6	W	95635541..95648328	10	427	88-393	4.72	47144.22	AT4G31860.1	Os06g44210.1	cyto
ZmPP114	GRMZM2G006416	GRMZM2G006416_P02	I	7	C	145764674..145770422	10	363	33-329	4.96	39537.45	AT4G31860.1	Os09g38550.1	chlo
ZmPP138[8]	GRMZM2G047376	GRMZM2G047376_P01	I	9	W	104264956..104269979	10	366	33-330	4.7	40146.8	AT4G31860.1	Os06g44210.1	cyto
ZmPP139	GRMZM2G104076	GRMZM2G104076_P02	I	9	W	104360920..104365697	10	428	107-403	5.1	46781.87	AT4G31860.1	Os06g44210.1	chlo
ZmPP157	GRMZM2G109496	GRMZM2G109496_P05	I	10	C	128323406..128328677	9	365	31-319	6	39849.96	AT4G31860.1	Os04g42260.1	cyto
ZmPP23	GRMZM2G134227	GRMZM2G134227_P01	K	1	W	291254272..291259644	3	466	234-461	8.74	49660.16	AT4G16580.1	Os03g59470.1	chlo
ZmPP46	GRMZM2G003096	GRMZM2G003096_P01	K	3	C	14335790..14339102	11	356	83-318	5.25	38093.09	AT2G30170.1	Os01g07090.1	chlo
ZmPP78	AC210013.4_FG011	AC210013.4_FGP011	K	5	C	3393450..3397191	3	468	239-466	8.4	49521.62	AT4G16580.1	Os03g59470.1	chlo
ZmPP82	GRMZM2G071087	GRMZM2G071087_P01	K	5	C	38472076..38479696	8	565	330-557	4.46	58875.9	AT4G33500.1	Os10g22460.1[9]	cyto
ZmPP91	GRMZM2G159904	GRMZM2G159904_P01	K	5	W	187808501..187809804	0	320	83-318	7.7	34329.01	AT4G16580.1	Os02g42270.1	cyto
ZmPP142	AC217887.3_FG001	AC217887.3_FGP001	K	9	C	122054370..122055332	0	322	82-320	6.01	34141.59	AT4G16580.1	Os02g42270.1	cyto
ZmPP44	GRMZM2G053713	GRMZM2G053713_P01	KAPP	2	C	226963057..227123377	9	741	12-316	6.26	84632.43	AT3G63340.1	Os11g37540.1	cyto
ZmPP37	GRMZM2G150608	GRMZM2G150608_P01	Un	2	W	160781682..160789474	12	581	315-571	6.2	63708.75	AT5G19280.1	Os07g11010.1	chlo
ZmPP58	GRMZM2G372297	GRMZM2G372297_P01	Un	3	C	226956068..226960039	10	383	63-342	5.43	41052.65	AT4G27800.1	Os01g37130.1	chlo
ZmPP110	GRMZM2G042627	GRMZM2G042627_P01	Un	7	C	19320389..19347469	12	583	313-571	7.03	63731.92	AT5G19280.1	Os07g11010.1	vacu
**PTP**														
ZmPP1[10]	GRMZM2G052546	GRMZM2G052546_P03	DSP/ZmRIP1	1	W	2538102..2544683	13	373	111-227	5.77	41850.63	AT3G52180.1	Os03g01750.1	chlo
ZmPP16	GRMZM2G127374	GRMZM2G127374_P01	DSP	1	W	219567806..219574992	9	596	302-423	8.89	66120.27	AT3G01510.1	Os08g29160.1	chlo
ZmPP18	GRMZM2G146819	GRMZM2G146819_P01	DSP	1	W	246654816..246667719	13	1264	97-250,514-670	6.26	142388.08	AT3G62010.1	Os03g38970.1	nucl
ZmPP20	GRMZM2G003640	GRMZM2G003640_P01	DSP	1	W	256296778..256306481	5	197	46-172	7.63	21197.67	AT3G23610.2	Os01g29469.1	nucl
ZmPP33	GRMZM2G042897	GRMZM2G042897_P02	DSP	2	W	143672210..143674292	1	238	122-229	9.62	26580.32	AT5G56610.1	Os12g05660.3	chlo
ZmPP34	GRMZM2G173035	GRMZM2G173035_P01	DSP	2	C	148054055..148060611	5	354	91-184	5.65	39044.59	AT4G18593.1	Os11g04180.1	cyto
ZmPP38	GRMZM2G086418	GRMZM2G086418_P01	DSP	2	W	161951772..161962083	4	235	76-228	5.51	26431.25	AT1G05000.1	Os09g05020.1	cyto
ZmPP45	GRMZM2G132468	GRMZM2G132468_P01	DSP	2	W	235478100..235485711	11	652	277-306	5.72	70410.49	AT3G19420.1	Os12g21890.1	nucl
ZmPP48	GRMZM2G139892	GRMZM2G139892_P02	DSP	3	C	55801368..55808447	10	928	707-838	5.96	103067.26	AT5G23720.1	Os01g20940.1	nucl
ZmPP52	GRMZM2G060027	GRMZM2G060027_P02	DSP	3	W	176810128..176817038	1	256	123-232	8.46	28293.99	AT5G56610.1	Os01g64010.1	plas
ZmPP54	GRMZM2G051288	GRMZM2G051288_P01	DSP	3	W	199661246..199664915	5	340	92-209	5.34	37618.69	AT2G35680.1	Os01g53710.1	cyto
ZmPP59	GRMZM2G000660	GRMZM2G000660_P01	DSP	3	C	230742740..230746075	3	280	127-227	9.15	31420.87	AT3G10940.1	Os12g02120.2	chlo
ZmPP62	GRMZM2G030463	GRMZM2G030463_P01	DSP	4	C	12580010..12609706	16	683	133-262	6.24	80034.03	AT3G09100.2	Os11g11070.1	nucl
ZmPP67	GRMZM2G126765	GRMZM2G126765_P01	DSP	4	C	177642843..177645152	4	263	92-255	8.7	29310.63	AT1G05000.1	Os02g53160.1	chlo
ZmPP68	GRMZM2G031094	GRMZM2G031094_P02	DSP	4	C	188486869..188489292	1	238	122-229	9.67	26652.47	AT5G56610.1	Os12g05660.3	plas
ZmPP93	GRMZM2G045404	GRMZM2G045404_P01	DSP	5	C	203651899..203655593	4	269	57-179	7.08	30699.34	AT2G04550.1	Os02g48840.1	nucl
ZmPP94	GRMZM2G084086	GRMZM2G084086_P01	DSP	5	W	210752879..210755426	4	220	52-214	6.3	24358.91	AT1G05000.1	Os06g10650.1	cyto
ZmPP101	GRMZM2G134389	GRMZM2G134389_P01	DSP	6	C	109281527..109286136	4	213	55-207	8.4	24477.55	AT1G05000.1	Os06g10650.1	cyto
ZmPP102	GRMZM2G174170	GRMZM2G174170_P02	DSP	6	W	120851110..120857801	4	275	56-180	6.32	30627.39	AT2G04550.1	Os06g20340.1	nucl

ZmPP106	GRMZM2G031435	GRMZM2G031435_P01	DSP	6	C	160119167..160123160	5	371	115-208	5.98	41585.3	AT2G35680.1	Os05g44910.1	cyto
ZmPP120	GRMZM2G061684	GRMZM2G061684_P01	DSP	8	W	40383566..40388878	9	866	647-780	5.92	96721.05	AT5G23720.1	Os01g24470.1	nucl
ZmPP125	GRMZM2G005350	GRMZM2G005350_P01	DSP	8	C	133181699..133186955	1	766	147-277	7.9	83316.91	AT3G55270.1	Os05g02500.1	nucl
ZmPP131	GRMZM2G168087	GRMZM2G168087_P01	DSP	8	C	166800538..166804990	1	256	123-232	8.98	28273.96	AT5G56610.1	Os01g64010.1	plas
ZmPP133	GRMZM2G070315	GRMZM2G070315_P01	DSP	9	W	8778312..8783014	4	216	58-210	8.84	24645.75	AT1G05000.1	Os06g10650.1	cyto
ZmPP140	GRMZM2G119079	GRMZM2G119079_P01	DSP	9	C	115320787..115325491	5	347	123-222	6.1	38789.52	AT2G35680.1	Os10g41240.1	cyto
ZmPP51	GRMZM2G010775	GRMZM2G010775_P01	PTP	3	C	140409749..140417545	7	351	109-340	7.26	39754.83	AT1G71860.3[11]	Os12g07590.1	mito
ZmPP47	GRMZM2G151087	GRMZM2G151087_P02	PTPL	3	W	17429943..17433359	8	221	55-218	9.8	25205.72	AT5G10480.1	Os04g20280.1	chlo
ZmPP14	GRMZM2G319357	GRMZM2G319357_P01	LMWP	1	W	205241339..205247763	5	336	171-323	9.02	36055.96	AT3G44620.1	Os08g44320.1	nucl
ZmPP145	GRMZM2G011520	GRMZM2G011520_P01	CDC25	9	W	126671626..126675008	2	131	10-112	7.65	14245.15	AT5G03455.1	Os10g39860.2	mito
**PP2A**														
ZmPP5	GRMZM2G160237	GRMZM2G160237_P01		1	C	39043378..39051301	2	322	38-313	5.1	36260.52	AT2G39840.1	Os03g16110.1	cyto
ZmPP11	GRMZM2G028700	GRMZM2G028700_P01		1	W	174634733..174645383	20	996	660-950	5.68	105657.03	AT2G27210.1	Os12g42310.1	cysk
ZmPP13	GRMZM2G004582	GRMZM2G004582_P01		1	C	189474106..189481738	6	623	259-582	9.36	68793.08	AT5G63870.1	Os08g40200.1	chlo
ZmPP15	GRMZM2G470452	GRMZM2G470452_P01		1	W	208732967..208734529	1	406	25-290	8.63	45634.58	AT1G64040.1	Os08g35440.1	cyto
ZmPP17	GRMZM2G382077	GRMZM2G382077_P01		1	W	242151150..242169299	10	315	26-301	5.04	35811.51	AT3G58500.1	Os10g27050.1[12]	cyto
ZmPP19	GRMZM2G070323	GRMZM2G070323_P01		1	C	250428573..250443357	17	924	666-924	5.74	97595.07	AT2G27210.1	Os03g44500.1	cyto
ZmPP22	GRMZM2G062394	GRMZM2G062394_P01		1	C	290103711..290108581	10	307	18-293	5.16	35205.81	AT3G58500.1	Os03g59060.1	cyto
ZmPP32	GRMZM2G039359	GRMZM2G039359_P04		2	C	133469918..133475008	5	413	81-373	5.91	45336.93	AT5G63870.1	Os08g40200.1	E.R.
ZmPP36	GRMZM2G180691	GRMZM2G180691_P01		2	W	153956036..153967953	5	307	16-292	5.33	34951.91	AT5G55260.1	Os09g11230.1	cyto
ZmPP50	GRMZM2G009593	GRMZM2G009593_P01		3	W	87798819..87808953	20	998	663-952	5.65	105693.14	AT2G27210.1	Os12g42310.1	cysk
ZmPP55	GRMZM2G055905	GRMZM2G055905_P01		3	W	206851721..206857487	9	303	13-289	5.08	34740.45	AT3G19980.1	Os01g49690.1	cyto
ZmPP65[13]	GRMZM2G112240	GRMZM2G112240_P01		4	C	170914968..170917894	2	316	24-299	5.27	35763.02	AT2G39840.1	Os02g57450.1	cyto
ZmPP73	GRMZM2G338631	GRMZM2G338631_P01		4	C	228371898..228374204	0	177	1-177	5.81	20324.53	AT5G59160.3	Os02g57450.1	cyto
ZmPP75	GRMZM2G038195	GRMZM2G038195_P01		4	C	230556470..230569433	3	306	17-292	4.89	35126.74	AT1G10430.1	Os06g37660.1	cyto
ZmPP79	GRMZM2G108355	GRMZM2G108355_P01		5	W	3660187..3664632	10	307	18-393	5.16	35189.81	AT3G58500.1	Os03g59060.1[12]	nucl
ZmPP81	GRMZM2G148539	GRMZM2G148539_P02		5	C	16615450..16618165	7	421	62-351	5.43	47221.05	AT2G27210.1	Os03g44500.1	cyto
ZmPP88	GRMZM2G133464	GRMZM2G133464_P01		5	C	156260125..156276652	5	306	17-292	4.83	35053.65	AT1G10430.1	Os02g12580.1	cyto
ZmPP96	GRMZM2G390076	GRMZM2G390076_P02		5	W	216074554..216083395	2	317	25-300	5.26	35814.03	AT2G39840.1	Os02g57450.1	cyto
ZmPP98	GRMZM2G041822	GRMZM2G041822_P01		6	W	78831860..78838998	2	324	24-299	5.41	36745.2	AT1G64040.1	Os06g06880.1	cyto
ZmPP103	GRMZM5G899390	GRMZM5G899390_P01		6	C	127199973..127203814	2	322	32-307	5.29	36296.83	AT5G27840.1	Os01g24750.1	cyto
ZmPP104	GRMZM2G126435	GRMZM2G126435_P01		6	W	131454814..131463174	11	483	196-466	5.72	54679.29	AT2G42810.1	Os05g11550.1	nucl
ZmPP119	GRMZM2G120202	GRMZM2G120202_P01		8	C	40152776..40157471	2	325	32-307	5.13	36581.01	AT5G27840.1	Os01g24750.1	cyto
ZmPP124	GRMZM2G137286	GRMZM2G137286_P02		8	C	83126356..83129282	2	335	32-307	5.59	37858.59	AT5G27840.1	Os01g24750.1	cyto
ZmPP135	GRMZM2G016930	GRMZM2G016930_P01		9	C	16491882..16497920	2	404	104-379	8.75	45362.73	AT4G11240.1	Os06g06880.1	chlo
ZmPP136	GRMZM2G119546	GRMZM2G119546_P01		9	C	81640174..81653751	5	306	17-292	4.83	35157.82	AT1G10430.1	Os06g37660.1	cyto
ZmPP150	GRMZM2G420926	GRMZM2G420926_P01		9	C	151473561..151480045	10	313	24-299	5.02	35624.24	AT3G58500.1	Os03g07150.1[12]	cyto

[1] Kuhn, J.M., Boisson-Dernier, A., Dizon, M.B., Maktabi, M.H., and Schroeder, J.I. (2006). The protein phosphatase AtPP2CA negatively regulates abscisic acid signal transduction in Arabidopsis, and effects of abh1 on AtPP2CA mRNA. Plant physiology 140, 127-139.

[2] Jiang, Y., Dong, J., Chen, R., Gao, X., and Xu, Z. (2011). Isolation of a novel PP2C gene from rice and its response to abiotic stresses. African Journal of Biotechnology 10, 7143-7154

[3] Fu, H., Feng, L., Li, N., Liang, H., and Wan, C. (2009). Differential Expression of Serine/Threonine Protein Phosphatase Type-2C Under Drought Stress in Maize. Plant Mol Biol Rep 27, 29-37

[4] Park, C.J., Peng, Y., Chen, X., Dardick, C., Ruan, D., Bart, R., Canlas, P.E., and Ronald, P.C. (2008). Rice XB15, a protein phosphatase 2C, negatively regulates cell death and XA21-mediated innate immunity. PLoS biology 6, e231.

[5] Hu, X., Liu, L., Xiao, B., Li, D., Xing, X., and Kong, X. (2010). Enhanced tolerance to low temperature in tobacco by over-expression of a new maize protein phosphatase 2C, ZmPP2C2. Journal of plant physiology 167, 1307-1315.

[6] Liu, L., Hu, X., Song, J., Zong, X., and Li, D. (2009). Over-expression of a Zea mays L. protein phosphatase 2C gene (ZmPP2C) in Arabidopsis thaliana decreases tolerance to salt and drought. Journal of plant physiology 166, 531-542.

[7] Rodriguez, P.L. (1998). Protein phosphatase 2C (PP2C) function in higher plants. Plant molecular biology 38, 919-927.

[8] Broz, A.K., Thelen, J.J., Muszynski, M.G., Miernyk, J.A., and Randall, D.D. (2001). ZMPP2, a novel type-2C protein phosphatase from maize. Journal of experimental botany 52, 1739-1740.

[9] Hu, X., Song, F., Zheng, Z., Molecular characterization and expression analysis of a rice protein phosphatase 2C gene, OsBIPP2C1, and overexpression in transgenic tobacco conferred enhanced disease resistance and abiotic tolerance. Physiologia Plantarum 127, 225-236

[10] Li, B., Zhao, Y., Liang, L., Ren, H., Xing, Y., Chen, L., Sun, M., Wang, Y., Han, Y., Jia, H., Huang, C., Wu, Z., and Jia, W. (2012). Purification and characterization of ZmRIP1, a novel reductant-inhibited protein tyrosine phosphatase from maize. Plant physiology 159, 671-681.

[11] Gupta, R., and Luan, S. (2003). Redox control of protein tyrosine phosphatases and mitogen-activated protein kinases in plants. Plant physiology 132, 1149-1152.

[12] Yu, R.M., Wong, M.M., Jack, R.W., and Kong, R.Y. (2005). Structure, evolution and expression of a second subfamily of protein phosphatase 2A catalytic subunit genes in the rice plant (Oryza sativa L.). Planta 222, 757-768.

[13] Smith, R.D., and Walker, J.C. (1991). Isolation and expression of a maize type 1 protein phosphatase. Plant physiology 97, 677-683.
