# Supplementary material for: Maize protein phosphatase gene family: identification and molecular characterization
Source: BMC Genomics. 2014 Sep 9;15(1):773. doi: 10.1186/1471-2164-15-773 (PMC4169795; doi:10.1186/1471-2164-15-773)
Supplement: Supplementary file 13 — Additional file 13: Table S3: List of 152 detected ZmPP genes and their expression patterns at 18 selected tissues. (PDF 344 KB) [file 12864_2014_6458_MOESM13_ESM.pdf]

Table S3. List of 152 detected ZmPP genes and their expression change at 18 selected tissues.

Name	24H_Germinating.Seed	6DAS_GH_Primary.Root	V3_Stem.and.SAM	V5_Tip.of.stage.2.Leaf	V9_Immature.Leaves	V9_Thirteenth.Leaf	V9_Eleventh.Leaf	V9_Eighth.Leaf	VT_Thirteenth.Leaf	R2_Thirteenth.Leaf	10DAP_Whole.seed	12DAP_Whole.seed	14DAP_Whole.seed	16DAP_Whole.seed	12DAP_Endopsperm	14DAP_Endopsperm	16DAP_Endosperm	16DAP_Embryo	S	Xmean	CV
ZmPP19	86.73	96.93	80.92	155.17	100.46	87.72	77.76	125.73	185.27	174.25	117.77	104.71	134.46	148.4	130.96	155.7	141.94	106.01	32.56633	122.8272	0.265139
ZmPP11	133.24	58.8	67.12	130.48	87.99	79.64	71.15	119.04	134.95	123.5	94.3	124.51	123.79	126.6	149.12	152.54	92.81	96.85	28.63831	109.2461	0.262145
ZmPP50	83.74	70.53	59.4	65.69	72.53	70.66	63.16	123.65	115.51	105.53	83.32	101.11	124.05	147.68	140.07	173.68	145.75	96.3	34.2432	102.3533	0.334559
ZmPP23	78.78	49.75	86.66	189.52	163.67	101.15	111.28	127.04	114.8	98.24	92.07	98.28	97.51	98.88	152.89	163.72	219.46	78.5	43.521	117.9	0.369135
ZmPP103	120.65	52.49	44.1	103.13	89.52	93.72	97.44	116.15	114.81	139.26	75.4	93.09	103.76	106.97	106.68	127.65	133.98	40.76	28.79286	97.75333	0.294546
ZmPP70	183.46	120.24	139.47	105	56.56	51.1	63.6	105.92	119.73	131.53	57.35	75.22	84.32	100.29	108.76	104.97	127.76	190.12	39.68912	106.9667	0.371042
ZmPP122	93.5	109.27	88.72	75.5	109.62	92.15	64.59	51.36	60.25	91.33	95.37	79.98	83.46	73.44	92.78	61.61	85.18	190.37	30.04134	88.80444	0.338286
ZmPP17	70.68	97.16	114.34	81.1	78.04	64.76	46.52	62.83	77.66	74.16	104.08	123.01	143.79	123.3	189.57	194.79	164.74	164.69	45.53477	109.7344	0.414954
ZmPP22	105.97	92.63	115.45	61.08	104.93	72.12	40.28	61.83	72.86	75.06	103.79	111.25	101.98	102.68	142.06	134.5	137.85	91.88	28.02364	96.01111	0.291879
ZmPP88	106.93	99.19	80.16	44.96	69.41	95.48	68.29	80.93	64.67	79.4	111.07	121.26	132.77	118.24	191.79	170.47	100.31	152.78	38.49881	104.895	0.367022
ZmPP55	117.1	163.56	126.32	133.32	76.78	68.3	58.5	48.35	101.41	108.25	94.35	98.06	190.17	173.84	329.06	266.66	152.33	103.63	71.98124	133.8883	0.537621
ZmPP102	292.66	155.15	84.21	83.54	77.63	56.09	53.02	65.46	52.8	76.76	102.75	138.84	182.03	144.6	167.03	190.63	215.4	80.27	66.93998	123.2706	0.543033
ZmPP29	120.53	269.24	192.24	34.44	160.24	60.87	62.25	63.77	150.52	92.93	225.51	169.79	130.4	132.78	105.09	92.63	98.35	109.66	60.79436	126.18	0.481807
ZmPP25	77.32	54.13	60.89	124.71	56.8	58.24	56.25	75.8	103.98	64.74	102	148.85	170.96	183.05	251.27	239.7	215.82	85.6	67.04556	118.3394	0.566553
ZmPP106	47.87	96.13	57.95	74.81	65.42	78.2	75.62	59.74	77.76	80.18	123.1	124.54	169.89	152.38	152.64	206.02	133.43	58.24	45.68551	101.8844	0.448405
ZmPP110	51.97	42.52	49.12	69.24	44.18	81.43	70.5	55.91	38.5	45.12	81.15	97.11	132.97	155.34	134.48	201.77	139.19	70.97	46.96158	86.74833	0.541354
ZmPP98	76.72	76.68	95.03	33.81	85.19	48.04	46.74	58.47	49.8	43.11	132.33	151.95	222.82	182.97	241.58	280.86	282.06	172.76	84.987	126.7178	0.670679
ZmPP18	18.37	47.75	46.42	76.9	57.95	43.26	34.98	68	113.66	127.33	72.47	68.55	142.88	208.62	124.57	207.43	200.61	35.54	61.91452	94.18278	0.657387
ZmPP93	135.43	63.16	50.11	16.94	74.64	54.36	57.89	35.37	17.22	17.35	90.66	91.39	93.99	106.64	73.88	121.05	142.91	52.85	38.85932	71.99111	0.539779
ZmPP49	65.22	75.61	57.09	36.29	45.73	46.56	32.25	27.74	16.31	23.95	73.82	88.45	114.63	112.64	150.96	164.83	110.83	52.17	43.61522	71.94889	0.606197
ZmPP36	33.92	35.71	37.29	38.35	78.27	60.91	73.64	48.68	62.46	34.97	113.93	105.6	91.85	71	118.05	96.43	30.08	18.42	31.35327	63.86444	0.490935
ZmPP108	104.08	51.3	50.86	48.2	54.26	57.24	67.53	59.24	35.6	23.2	113.55	69.53	64.04	60.08	26.2	37.29	27.04	40.74	24.16731	54.99889	0.439415
ZmPP109	68.27	61.73	37.15	82.04	65.04	55.12	28.91	31.86	18.64	34.39	33.5	31.26	46.16	32.12	52.96	43.44	33.9	72.83	17.87047	46.07333	0.38787
ZmPP72	84.95	156.84	137.51	40.26	116.19	68.03	48.36	32.48	34.17	40.15	86.07	63.26	46.52	42.47	69.72	38.06	32.38	227.75	52.97834	75.84278	0.698528
ZmPP2	96.09	58.61	62.55	29.46	50.28	39.55	38.39	50.82	48.92	47.35	78.04	58.68	71.52	66.38	34.51	54.61	68.87	38.77	16.94713	55.18889	0.307075
ZmPP4	54.73	54.5	40.08	15.25	35.33	28.29	44.2	39.33	70.65	52.03	62.42	60.92	62.61	90.72	47.06	48.54	118.18	32.02	23.64784	53.15889	0.444852
ZmPP151	42.26	23.46	34.52	36.3	53.43	21.77	45.35	75.27	49.1	40.51	92.99	51.84	49.94	50.37	37.77	79.58	75.39	35.53	19.52438	49.74333	0.392502
ZmPP34	67.52	74.63	54.15	45.5	43.09	38.61	43.56	66.42	58.23	44.21	62.19	75.31	82.35	83.45	93.47	118.69	97.76	54.43	22.07527	66.865	0.330147
ZmPP52	86.02	35.54	54.07	44.08	23.9	26.58	24.34	47.96	64.47	68.14	73.96	63.89	82.94	79.97	69.16	68.67	66.27	107.72	23.12912	60.42667	0.382763
ZmPP51	52.28	34.73	40.73	32.44	36.55	42.31	36.28	77.74	48.34	54.47	45.67	48.52	39.22	35.68	43.57	31.53	27.7	57.84	11.9054	43.64444	0.272782
ZmPP139	40.26	27.95	35.82	20.94	55.43	25.63	40.68	39.12	41.62	48.76	47.17	36.18	37.65	39.65	41.29	40.07	29.74	24.45	8.897354	37.35611	0.238177
ZmPP44	19.65	31.48	19.39	32.72	28.48	33.06	26.26	34.58	73.9	82.14	41.87	40.13	49.64	74.91	43.43	58.45	58.85	35.1	18.954	43.55778	0.435146
ZmPP73	26.46	48.61	29.14	46.27	11.46	27.9	35.48	66.78	72.4	49.87	34.11	42.83	52.06	43.63	47.09	49.15	35.61	19.04	15.40924	40.99389	0.375891
ZmPP100	114.47	101.34	56.33	124.67	86.32	84.17	82.51	102.42	108.93	119.81	95.88	70.54	79.86	66.98	55.66	39.54	44.79	82.55	25.25959	84.265	0.299764
ZmPP144	123.14	68.86	55.47	66.03	59.83	61.24	57.76	80.77	64.79	85.04	63.5	49.81	57.04	59.5	36.47	57.84	61.65	52.92	18.1104	64.53667	0.280622
ZmPP118	46.86	121.81	80.73	79.14	109.83	94.25	97.67	55.47	74.3	46.54	98.66	69.16	69.23	67.07	45.07	47.09	44.7	51.53	24.26862	72.17278	0.336257
ZmPP3	123.85	95.99	104.14	78.05	94.1	110.81	96.29	93.43	104.13	96.83	144.2	104.86	100.7	94.74	72.62	69.84	75.37	35.26	23.22791	94.17833	0.246637
ZmPP99	34.81	33.42	43.42	144.55	59.78	160.48	140.63	171.7	199.57	226.62	123.63	85.98	65.84	100.22	31.71	63.51	59.27	31.91	62.33942	98.725	0.631445
ZmPP80	77.71	140.55	21.43	24.29	48.08	54.87	60.13	127.5	179.26	162.22	46.61	58.41	55.57	63.08	48.79	47.99	73.79	46.31	46.12137	74.255	0.621121
ZmPP21	75.69	163.88	19.66	27.28	57.76	33.63	34.15	92.61	417.64	206.29	41.72	34.33	42.05	45.29	44.78	34.15	56.44	29.78	97.29588	80.95167	1.201901
ZmPP158	178.95	84.6	50.6	49.34	22.28	49.2	63.26	178.15	119.29	163.78	107.07	69.75	97.11	101.26	54.89	62.14	61.74	8.25	49.71474	84.53667	0.588085

ZmPP6	1	98.92	9.3	55.95	26.67	93.52	90.96	134.77	234.03	156.6	34.8	27.67	83.61	108.86	46.78	66.46	65.89	180.34	61.72755	84.22944	0.73285
ZmPP78	77.56	85.94	90.82	409.53	136.55	209.05	228.66	229.23	176.52	153.46	101.87	99.93	121.37	109.81	113.57	165.19	110.8	130.77	79.57147	152.8128	0.520712
ZmPP153	131.92	97.38	72.33	206.8	84.29	112.84	98.91	158.68	154.45	232.7	93.99	74.36	90.59	115.9	73.6	95.47	115.4	112.82	44.72542	117.9128	0.379309
ZmPP89	64.26	263.61	123.59	147.73	151.43	134.48	118.88	89	222.52	210.84	49.76	86.86	118.24	113.63	150.83	157.15	134.82	103.06	54.21656	135.5939	0.399845
ZmPP54	37.67	78.43	96.41	67.84	74.84	206.44	204.39	75.82	54.08	51.27	118.71	106.13	111.87	124.28	105.68	101.73	119.66	99.89	45.24209	101.9522	0.443758
ZmPP115	250.89	344.61	178.36	135.41	158.08	108.32	111.13	157.21	126.66	170.25	89.07	53.28	67.48	59.79	42.77	65.4	75.16	167.12	76.53084	131.1661	0.583465
ZmPP156	78.37	104.91	134.46	142.66	68.27	50.09	170.65	194.37	133.09	110.5	73.26	52.13	48.14	34.29	43.86	28.94	22.56	79.63	50.75437	87.23222	0.581831
ZmPP87	274.9	134.86	77.16	214.74	98.48	145.96	157.3	214.48	244.05	195.42	28.29	34.44	45.01	50.64	33.42	61.58	227.81	4.41	87.18927	124.6083	0.699707
ZmPP150	43.53	84.17	68.76	537.66	55.03	63.27	53.17	391.59	628.05	375.22	61.49	99.89	154.89	137.31	164.95	195.88	137.23	89.46	176.5535	185.6417	0.951045
ZmPP101	69.1	121.23	106.65	582.91	31.27	54.49	73.21	124.9	260.53	171.79	114.35	106.21	100.77	87.79	85.79	67.37	77.4	46.84	125.0333	126.8111	0.985981
ZmPP145	100.86	430.88	229.15	1452.46	87.76	279.48	418.84	582.75	256.24	361.99	166.82	104.78	109.7	104.61	126.07	87.01	60.47	240.7	325.3778	288.9206	1.126184
ZmPP58	122.36	24.46	32.47	1899.49	74.05	136.88	213.28	1271.24	987.26	805.4	73.92	70.76	86.51	94.97	43.24	98.25	127.83	32.06	534.5665	344.135	1.553363
ZmPP10	47.99	25.64	23.44	74.4	24.56	20.89	17.24	39.67	55.22	47.74	17.63	21.12	15.55	24.01	27.94	17.47	16.3	19.11	16.48505	29.77333	0.553685
ZmPP131	19.17	21.4	17.85	21.75	25.06	15.8	17.02	22.47	33.74	35	18.99	19.85	26.72	25.42	33.02	32.01	16.02	29.55	6.41658	23.93556	0.268077
ZmPP143	30.32	10.16	19.8	24.01	16.98	50.37	58.6	30.39	21.22	19.25	11.03	18.08	24.33	34.68	18.84	30.05	23.89	27.1	12.30947	26.06111	0.472331
ZmPP42	16.81	36.33	37.08	25.97	40.91	30.38	49.93	46.19	37.25	32.09	32.77	24.21	15.58	16.96	5.97	6.79	12.2	33.36	13.04704	27.82111	0.468962
ZmPP116	12.36	18.85	27.98	25.23	11.71	13.95	17.7	27.09	58.56	88.58	33.1	21.14	27.88	55.89	21.5	35.61	73.65	28.17	21.75225	33.275	0.653712
ZmPP86	35.15	84.05	66.25	63.88	24.62	23.76	23.57	9.24	65.19	16.83	53.18	34.77	28.01	35.02	42.59	53.06	13.09	91.25	24.13621	42.41722	0.569019
ZmPP137	44.57	20.8	28.38	35.03	18.21	15.2	19.73	18.94	28.76	36.62	48.12	52.94	24.75	23.16	48.44	33.5	16.1	86.87	17.92774	33.34	0.537725
ZmPP123	63.72	53.15	47.41	24.21	45.2	47.83	39.8	30.05	18.73	12.09	53.86	30.82	36.61	44.17	26.2	27.6	20.57	89.75	18.71904	39.54278	0.473387
ZmPP90	47.38	33.88	23.53	16.95	39.99	35.54	26.51	14.62	21.1	11.84	40.81	41.91	32.3	32.77	40.87	29.46	14.77	22.18	10.82061	29.245	0.369999
ZmPP159	75.77	31.77	35.87	165.75	41.98	61.81	74.69	83.79	53.52	170.76	32.34	41.65	47.23	27.8	19.08	15.57	20.13	27.8	45.14869	57.07278	0.791072
ZmPP30	57.62	35.93	26.16	265.78	17.58	35.29	28.23	66.12	104.09	69.26	38.79	28.76	52.97	45.65	26.74	35.91	39.87	22.06	56.56333	55.37833	1.021398
ZmPP14	7	27.98	38.37	251.77	32.48	40.88	38.32	139.01	112.86	56.77	41	35.3	32.71	33.27	19.21	16.79	18.78	35.08	59.11932	54.31	1.088553
ZmPP16	36.37	11.11	15.32	194.19	21.87	42.26	55.82	101.67	105.98	58.97	11.03	32.16	40.08	52.33	58.09	64.07	59.46	20.86	44.21424	54.53556	0.810741
ZmPP125	12.4	22.69	19.02	12.51	18.86	20.24	15.52	21.85	11.74	15.06	17.52	19.67	15.39	17.67	10.92	11.33	12.41	13.47	3.792395	16.015	0.236803
ZmPP138	26.31	16.74	17.22	33.8	23.57	12.75	8.58	14.47	41.93	28.06	15.08	11.31	11.81	4.94	10.28	12.6	10.6	7.62	9.8612	17.09278	0.576922
ZmPP85	14.41	40.75	21.52	7.39	8.86	13.49	14.3	38.81	38.2	17.33	4.18	21.69	3.94	25.63	22.38	16.65	20.95	16.58	11.05393	19.28111	0.573304
ZmPP82	61.84	74.98	28.2	89.98	10.73	22.46	13.67	47.84	88.85	59.78	7.93	22.7	24.59	25.75	39.28	17.39	38.7	5.94	26.99687	37.81167	0.713983
ZmPP114	75.76	44.79	21.2	27.82	9.38	11.96	12.38	42.34	29.85	47.7	23.2	27.71	22.25	5.25	11.98	4.66	43.49	20.46	18.31071	26.78778	0.683547
ZmPP38	50.32	15.59	16.82	24.29	13.18	61.82	74.21	62.24	85.74	39.08	34.95	29.9	17.63	24.9	37.93	19.89	11.09	7.18	23.18014	34.82	0.665713
ZmPP128	17.92	16.89	18.28	40.04	67.26	58.47	77.96	56.55	100.36	42.21	40.82	23.87	18.76	19.83	7.53	5.79	14.51	0	27.71057	34.83611	0.795455
ZmPP155	6.59	7.76	11.01	34.28	10.1	20.51	35.25	89.86	180.88	261.1	40.2	19.72	19.8	28.6	7.88	8.79	5.43	11.13	68.67721	44.38278	1.547384
ZmPP1	9.25	19.32	9.46	211.37	7.31	32.74	39.79	53.17	112.52	102.59	5.83	15.78	9.49	10.31	8.61	11.09	4.33	9.66	54.01575	37.36778	1.445517
ZmPP83	71.68	31.36	24.31	105.5	42.17	53.22	78.98	117.54	181.27	106.38	75.35	42.28	53.77	56.59	22.98	30.38	5.99	8.93	44.40495	61.59333	0.720938
ZmPP107	48.2	42.27	31.19	142.04	41.21	58.26	52.45	63.99	159.11	178.67	91.25	64.72	55.99	47.13	14.83	25.94	0	3.33	50.55546	62.25444	0.812078
ZmPP59	30.13	11.58	20.36	464.6	58.85	110	107.85	82.33	197.53	148.3	46.8	29.58	19.49	17.16	8.26	9.58	0	39.82	110.7579	77.90111	1.421776
ZmPP28	56.73	71.16	34.63	68.1	67.71	90.63	86.86	61.63	75.75	59.35	43.11	34.38	30.86	29.01	19.89	11.55	5.88	22.41	25.90218	48.31333	0.536129
ZmPP12	31.37	45.8	37.47	46.55	80.72	52.42	46.43	48.85	67.07	45.84	60.33	44.75	29.96	25.42	18.93	14.75	6.03	66.71	19.43151	42.74444	0.454597
ZmPP134	76.46	87.51	80.47	58	93.92	127.86	138.9	112.95	78.94	60.23	58.76	20.75	24.19	27.46	12.51	11.93	0	61.02	40.9411	62.88111	0.651087
ZmPP8	24.85	391.91	52.67	37.84	102.52	148.16	246.17	669.76	229.76	118.12	40.81	53.15	33.14	40.03	59.16	18.81	0	76.63	167.7897	130.1939	1.288768
ZmPP132	346.97	36.85	31.38	79.56	41.34	224.3	195.71	126.77	340.7	241.99	84.43	48.4	51.85	33.31	0	0	0	96.19	112.4239	109.9861	1.022164
ZmPP53	154.01	41.77	24.35	37.36	38.92	134.18	123.95	149.89	268.66	214.99	51.58	20.59	26.04	10.53	0	0	0	87.25	79.80197	76.89278	1.037834
ZmPP71	32.14	2.62	63.79	711.8	68.54	77.8	139.25	350.62	620.52	569.74	136.58	67.69	72.32	63.43	7.49	6.86	2.95	16.21	230.5876	167.2417	1.378769
ZmPP62	93.89	84.47	108.92	164.67	101.8	109.65	123.11	151.57	235.94	218.06	177.87	195.21	253.95	276.46	273.04	308.82	243.79	323.81	78.28694	191.3906	0.409043

ZmPP79	98.65	110.13	97.73	109.73	125.69	103.81	81.15	109.83	132.4	124.21	128.9	159.91	154.8	208.13	233.8	230.28	308.01	178.08	60.29382	149.7356	0.402669
ZmPP104	105.17	153.32	107.8	139.19	100.88	143.41	140.62	65.4	127.27	152.5	233.06	266.91	283.64	267.23	415.18	410.91	281.32	165.61	103.2307	197.7456	0.522038
ZmPP136	221.13	105.9	129.84	135.77	113.92	138.75	132.06	135.75	147.11	126.47	204.34	181.26	156.36	170.18	223.23	212.9	133.61	169.38	37.11035	157.6644	0.235376
ZmPP45	141.36	151.59	140.42	117.04	154.95	143.24	107.71	109.51	97.41	112.8	164.69	149.47	160.11	132.86	167.43	181.89	100.46	154.92	25.2214	138.2144	0.18248
ZmPP140	294.6	223.92	166.07	296.76	164.78	249.5	248.95	331.43	399.94	351.53	292.81	223.44	201.89	139.78	125.06	121.73	150.25	100.07	87.5803	226.8061	0.386146
ZmPP57	207.83	111.27	64.92	278.65	127.14	150.95	133.96	124.52	400.19	344.95	261.22	239.11	328.61	224.49	225.46	246.34	194.02	267.13	89.08158	218.3756	0.407928
ZmPP26	294.8	291.29	209.19	189.75	126.69	79.33	181.86	240.94	243.86	215.49	485.61	459.04	473.04	441.23	423.19	518.92	288.71	144.09	137.9824	294.835	0.467999
E2enzyme	526.29	501.67	439.02	829.2	344.32	340.79	431.32	508.82	541.87	519.76	475.93	522.29	801.41	722.73	721.81	926.18	1034.85	680.41	197.9729	603.815	0.32787
ZmPP76	628.27	358.44	358.85	1317.49	354.8	299.29	284.39	422.72	950.86	1070.13	284.2	345.26	679.04	881.93	527.06	926.9	1323.07	470.39	354.3056	637.9494	0.555382
ZmPP84	436.03	256.04	402.69	1100.71	400.87	381.69	351.96	458.86	805.27	832.08	312.72	387.22	482.17	482.67	375.63	512.14	526.65	379.43	211.9055	493.6017	0.429305
ZmPP65	264.27	479.53	277.41	475.32	188.25	280.73	368.08	642.73	642.85	643.9	381.65	389.09	508.58	429.17	446.89	491.03	327.24	299.39	136.2597	418.6728	0.325456
ZmPP47	755.27	476.46	555.94	242.02	852.98	641.34	545.38	254.39	281.71	240.24	1058.96	820.18	762.06	695.32	609.25	584.71	706.63	400.18	233.7474	582.39	0.401359
ZmPP5	485.79	279.08	282.93	192.63	317.23	314.19	224.83	231.11	369.04	344.45	469.67	866.6	1062.57	1245.66	1348.98	1476.92	1671.46	229.44	500.1769	634.0322	0.788882
ZmPP9	17.51	51.18	21.52	10.94	33.68	14.92	15.29	8.2	7.61	9.24	32.91	28.39	45	59.24	33.93	56.91	67.38	15.26	19.31659	29.395	0.657139
ZmPP135	42.61	36.44	47.14	4.25	9.61	14.31	12.8	5.38	1.89	4.46	38.68	34.67	56.3	90.06	70.23	80.19	63.69	61.35	28.50864	37.44778	0.761291
ZmPP48	7.06	40.7	55.51	1.71	25.53	10.9	8.22	7.79	10.8	12.14	40.56	21.13	15.76	25.7	18.73	17.61	12.52	100.86	23.66088	24.06833	0.983071
ZmPP41	4.84	20.57	26.01	3.56	5.44	7.32	10.51	3.24	3.54	3.66	29.28	21.74	19.8	40.07	22.61	30.26	28.91	22.01	11.70103	16.85389	0.694263
ZmPP119	21.69	29.81	42.14	1	43.18	36.01	18.85	0	0	0	14.77	16.17	18.01	20.57	21.24	14.33	7.23	121.24	27.85896	23.68	1.176476
ZmPP129	14.77	19.3	23.31	13.4	20.32	20.91	15.53	0	0	0	23.64	14.27	16.68	16.49	18.15	14.18	15.18	45.68	10.38766	16.21167	0.640752
ZmPP20	7.69	12.18	22.69	2.86	20.07	9.74	68.7	18.77	116.35	1	28.12	23.94	90.37	6.69	20.62	3.1	109.26	115.73	41.76325	37.66	1.108955
ZmPP64	21.33	11.42	15.13	12.36	12.34	13.11	2.21	15.86	12.95	23.89	26.78	29.57	37.61	38.59	40.68	38.96	43.13	22.72	12.35227	23.25778	0.531103
ZmPP111	27.47	12.11	17.88	8.65	14.13	7.82	1.4	19.14	15.13	26.74	7.56	21.01	27.49	40.68	32.64	41.94	37.29	11.84	12.10153	20.60667	0.587263
ZmPP37	6.65	4	6.72	5.13	4.63	1.03	3.71	15.04	5.72	10.18	12.52	18.16	20.84	29.53	22.56	40.62	29.13	11.47	10.99587	13.75778	0.799247
ZmPP124	9.28	2.55	3.57	3.65	16.2	11.76	12.51	6.13	13.52	11.84	7.88	6.93	15.95	14.11	15.79	14.81	32.79	6	7.02542	11.40389	0.616055
ZmPP32	8.66	11.83	6.12	7.11	5.91	5.35	4.75	8.22	8.8	8.85	10.08	11.38	7.44	12.2	4.14	18.44	27.12	7.04	5.523947	9.635556	0.573288
ZmPP127	3.65	18.66	7.91	9	17.39	18.29	19.08	13.37	13.59	17.04	10.05	13.18	15.17	14.2	13.38	17.29	27.16	0	6.201505	13.80056	0.449366
ZmPP27	30.24	26.71	11.41	6.78	0	22.85	7.08	39.87	20.18	7.05	22.83	27.46	35.28	34.54	21.24	25.67	19.09	0	12.07559	19.90444	0.606678
ZmPP154	18.85	0	0	1	0	3.45	3.37	2.2	98.34	50.54	40.05	63.79	136.67	114.54	109.52	160.02	167.43	64.2	59.62732	57.44278	1.03803
ZmPP112	46.23	1	0	1.38	0	11.02	15.08	11.92	72.07	135.79	23.32	14.23	30.52	27.04	15.58	18.26	16.86	55.31	33.38418	27.53389	1.212476
ZmPP92	27.53	66.98	18.93	101.3	0	0	3.92	0	28.98	20.17	39.98	24.05	54.11	83.14	46.96	75.93	66.77	384.64	87.12356	57.96611	1.503009
ZmPP146	34.67	46.29	130.05	0	109.01	25.36	11.62	1.03	0	0	132.57	176.53	230.93	236.94	296.67	296.1	323.41	187.2	115.8347	124.3544	0.931489
ZmPP31	59.23	0	0	0	0	0	0	1.56	0	0	6.81	84.53	226.51	352.45	187.67	369.19	711.8	83.09	192.5529	115.7133	1.664051
ZmPP33	27.74	18.96	19.71	36.34	8.39	5.94	14.44	31.32	29.81	17.45	15.63	17.08	17.84	13.92	15.46	4.28	0	6.26	9.857497	16.69833	0.590328
ZmPP77	19.24	28.81	22.81	15.76	7.58	7.58	14.93	14.07	17.68	24.24	12.27	11	9.82	12.18	1.75	2.69	0	28.51	8.562715	13.94	0.614255
ZmPP117	11.08	5.37	8.81	16.52	5.73	7.33	6.22	10.81	15.87	18.61	9.61	5.01	6.12	8.54	4.82	2.79	4.72	8.31	4.44529	8.681667	0.512032
ZmPP152	28.24	12.65	8.12	16.69	6.92	16.94	30.89	47.82	28.57	34.23	23.97	23.69	11.47	7.59	1	5.51	0	1	13.38309	16.96111	0.789046
ZmPP147	5.53	70.4	11	12.71	13.58	22.65	50.78	124.61	17.99	9.42	7.44	0	6.11	2.83	1.94	0	0	18.35	31.71553	20.85222	1.520966
ZmPP66	7.72	11.71	2.46	16.01	4.65	21.03	19.79	4.42	23.26	26.19	8.73	0	1.5	0	0	0	0	0	9.175978	8.192778	1.120008
ZmPP149	11.83	31.42	6.6	19.94	0	18.76	38	93.64	49.31	77.6	0	0	6.73	15.1	22.27	7.45	20.8	0	26.64472	23.30278	1.143414
ZmPP39	109.83	9.38	0	11.21	0	15.53	18.43	48.53	108.15	132.07	12.18	0	9.6	23.95	4.59	0	0	62.25	42.8971	31.42778	1.364942
ZmPP24	5.47	48.37	13.48	121.56	29.64	34.27	65.03	204.93	364.07	257.86	7.82	2.61	0	7.15	0	0	0	5.18	105.4607	64.85778	1.62603
ZmPP105	29.36	16.51	9.29	50.66	17.14	31.45	80.86	222.44	78.9	95.09	1.72	1.91	2.45	3.72	0	0	1.87	1	56.00367	35.79833	1.564421
ZmPP46	1	1	7.89	557.11	16.68	97.57	145.82	395	290.13	132.36	4.39	5.18	3.62	3.18	0	0	0	3.62	161.8587	92.475	1.750297
ZmPP133	32.01	13.64	18.95	2070.35	0	0	7.62	2492.37	1535.25	826.17	12.51	3.39	15.1	29.07	10.68	9.91	0	12.76	795.1527	393.8767	2.018786
ZmPP120	0	18.64	12.61	0	18.22	3.35	2.59	0	0	0	11.3	7.03	5.77	7.59	5.33	5.85	0	9.34	6.091419	5.978889	1.018821

ZmPP69	3.53	8.33	6.87	2.43	8.18	2.07	4.44	0	0	1.11	6.65	5.43	1.69	4.6	3.69	2.31	0	19.02	4.517009	4.463889	1.0119
ZmPP68	11.05	12.43	10.87	1.6	14.9	9.61	6.55	2.85	0	0	6.64	4.23	7.18	7.8	9.79	6.47	0	19.58	5.405642	7.308333	0.739655
ZmPP13	13.47	5.05	8.09	6.03	2.9	5.35	1.83	8.96	1	2.03	10.9	1	0	1	1.51	20.99	30.28	4.17	7.93547	6.92	1.146744
ZmPP74	22.52	1.19	1.41	1	6.14	4.41	1	4.43	7.68	2.64	4.58	4.12	4.9	5.68	3.07	6.13	14.15	3.22	5.261377	5.459444	0.96372
ZmPP75	15.94	6.8	5.21	6.94	1.05	7.64	3.24	1.17	2.34	2.41	23.09	14.26	23.13	28.05	13.22	31.71	15.69	1	9.873044	11.27167	0.875917
ZmPP141	15.64	30.9	14.18	1.65	3.48	8.72	7.72	3.95	1.03	3.67	30.75	16.46	20.07	18.21	6.37	11.35	1.03	1	9.59569	10.89889	0.880428
ZmPP7	0	0	2.65	0	0	0	0	0	0	0	9.34	7.97	11.02	24.31	5.09	2.75	0	0	6.335034	3.507222	1.806282
ZmPP121	72.84	0	1	4.7	35.55	11.33	11.98	10.76	2.79	2.31	15.43	10.76	10.66	4.29	6.02	2.05	0	20.53	17.45321	12.38889	1.40878
ZmPP35	20.95	0	59.31	0	67.6	33.15	11.08	0	0	0	8.47	8.14	9.41	12.38	0	0	0	45.66	21.66058	15.34167	1.411879
ZmPP67	0	151.88	157.7	30.81	0	0	6.07	18.07	0	0	23.34	22.02	21.44	21.79	0	0	0	0	48.38997	25.17333	1.922271
ZmPP97	0	0	0	15.04	0	4.95	5.25	7.88	18.54	15.86	0	0	0	0	0	0	0	0	6.335508	3.751111	1.688968
ZmPP95	1.86	2.19	1.22	8.29	1.55	1.73	4.92	9.67	3.99	1.45	1	1.55	1	1.96	1	1	0	1	2.617111	2.521111	1.038078
ZmPP81	5.21	4.35	7.98	5.22	1	1.3	3.6	10.58	10.85	8.73	1	7	1.38	4.9	9.53	4.65	1.46	2.08	3.387656	5.045556	0.671414
ZmPP96	4.41	1	1	1	8.35	3.88	17.38	3.61	6.7	8.91	1	1.24	1	1.64	1	1	12.17	7.33	4.71193	4.59	1.026564
ZmPP130	0	0	0	0	0	0	0	0	0	0	2.03	0	3.6	0	0	2.91	0	0	1.124519	0.474444	2.370181
ZmPP148	0	0	0	0	0	0	0	0	0	0	1.65	0	0	3.89	0	0	0	0	0.974665	0.307778	3.166781
ZmPP40	4.13	0	0	0	0	2.64	0	0	0	0	0	0	0	0	0	0	0	0	1.124074	0.376111	2.988676
ZmPP43	0	0	0	0	0	0	0	2.41	10.75	0	0	0	0	0	0	0	0	0	2.56388	0.731111	3.506827
ZmPP60	0	0	0	0	0	0	0	0	4.16	0	0	0	0	0	0	0	0	0	0.980521	0.231111	4.242641
ZmPP113	0	0	2.49	0	0	52.91	30.72	0	0	0	1.08	0	0	0	0	0	0	0	13.99184	4.844444	2.888224
ZmPP94	0	208.51	0	0	0	0	0	0	0	0	0	0	0	0	0	0	0	0	49.14628	11.58389	4.242641
ZmPP56	0	0	0	0	0	0	0	0	0	0	1.54	1	1	2.76	31.21	2.01	72.61	31.26	18.93834	7.966111	2.377363
