# Supplementary material for: Maize protein phosphatase gene family: identification and molecular characterization
Source: BMC Genomics. 2014 Sep 9;15(1):773. doi: 10.1186/1471-2164-15-773 (PMC4169795; doi:10.1186/1471-2164-15-773)
Supplement: Supplementary file 14 — Additional file 14: Table S4: List of putative root development signaling components in maize. (PDF 116 KB) [file 12864_2014_6458_MOESM14_ESM.pdf]

Table S4. List of putative root development signaling components in maize.

Name	Gene ID	Protein ID	Class
ZmPP11	GRMZM2G028700	GRMZM2G028700_P01	PP2A
ZmPP13	GRMZM2G004582	GRMZM2G004582_P01	PP2A
ZmPP15	GRMZM2G470452	GRMZM2G470452_P01	PP2A
ZmPP17	GRMZM2G382077	GRMZM2G382077_P01	PP2A
ZmPP19	GRMZM2G070323	GRMZM2G070323_P01	PP2A
ZmPP22	GRMZM2G062394	GRMZM2G062394_P01	PP2A
ZmPP32	GRMZM2G039359	GRMZM2G039359_P04	PP2A
ZmPP36	GRMZM2G180691	GRMZM2G180691_P01	PP2A
ZmPP50	GRMZM2G009593	GRMZM2G009593_P01	PP2A
ZmPP55	GRMZM2G055905	GRMZM2G055905_P01	PP2A
ZmPP65	GRMZM2G112240	GRMZM2G112240_P01	PP2A
ZmPP73	GRMZM2G338631	GRMZM2G338631_P01	PP2A
ZmPP75	GRMZM2G038195	GRMZM2G038195_P01	PP2A
ZmPP79	GRMZM2G108355	GRMZM2G108355_P01	PP2A
ZmPP81	GRMZM2G148539	GRMZM2G148539_P02	PP2A
ZmPP88	GRMZM2G133464	GRMZM2G133464_P01	PP2A
ZmPP96	GRMZM2G390076	GRMZM2G390076_P02	PP2A
ZmPP98	GRMZM2G041822	GRMZM2G041822_P01	PP2A
ZmPP103	GRMZM5G899390	GRMZM5G899390_P01	PP2A
ZmPP104	GRMZM2G126435	GRMZM2G126435_P01	PP2A
ZmPP119	GRMZM2G120202	GRMZM2G120202_P01	PP2A
ZmPP124	GRMZM2G137286	GRMZM2G137286_P02	PP2A
ZmPP135	GRMZM2G016930	GRMZM2G016930_P01	PP2A
ZmPP136	GRMZM2G119546	GRMZM2G119546_P01	PP2A
ZmPP150	GRMZM2G420926	GRMZM2G420926_P01	PP2A
ZmPIN1a	GRMZM2G098643	GRMZM2G098643_P01	PIN
ZmPIN1b	GRMZM2G074267	GRMZM2G074267_P01	PIN
ZmPIN1c	GRMZM2G149184	GRMZM2G149184_P01	PIN
ZmPIN1d	GRMZM2G171702	GRMZM2G171702_P01	PIN
ZmPIN5a	GRMZM2G025742	GRMZM2G025742_P01	PIN
ZmPIN5b	GRMZM2G148648	GRMZM2G148648_P01	PIN
ZmPIN5c	GRMZM2G040911	GRMZM2G040911_P01	PIN
ZmPIN8	GRMZM5G839411	GRMZM5G839411_P01	PIN
ZmPIN9	GRMZM5G859099	GRMZM5G859099_P01	PIN
ZmPIN10a	GRMZM2G126260	GRMZM2G126260_P02	PIN
ZmPIN10b	GRMZM2G160496	GRMZM2G160496_P01	PIN
ZmPINY	GRMZM2G050089	GRMZM2G050089_P02	PIN
KZM1	GRMZM2G178356	GRMZM2G178356_P01	K^+ channel
KZM2	GRMZM2G093313	GRMZM2G093313_P02	K^+ channel
ZmWOX11	GRMZM2G170958	GRMZM2G170958_P01	WOX
ZmRR1	GRMZM2G040736	GRMZM2G040736_P01	cytokinin-inducible response regulators

ZmRR2	GRMZM2G392101	GRMZM2G392101_P01	cytokinin-inducible response regulators
ZmARF1	GRMZM2G169820	GRMZM2G169820_P01	ARF
ZmARF2	GRMZM2G153233	GRMZM2G153233_P01	ARF
ZmARF3	GRMZM2G078274	GRMZM2G078274_P01	ARF
ZmARF4	GRMZM2G034840	GRMZM2G034840_P02	ARF
ZmARF5	GRMZM5G808366	GRMZM5G808366_P01	ARF
ZmARF6	GRMZM2G017187	GRMZM2G017187_P02	ARF
ZmARF7	GRMZM2G475263	GRMZM2G475263_P01	ARF
ZmARF9	GRMZM2G073750	GRMZM2G073750_P01	ARF
ZmARF10	GRMZM2G338259	GRMZM2G338259_P02	ARF
ZmARF11	GRMZM2G056120	GRMZM2G056120_P01	ARF
ZmARF12	GRMZM2G437460	GRMZM2G437460_P01	ARF
ZmARF13	GRMZM2G378580	GRMZM2G378580_P01	ARF
ZmARF14	GRMZM2G702026	GRMZM2G702026_P01	ARF
ZmARF15	GRMZM2G081406	GRMZM2G081406_P01	ARF
ZmARF16	GRMZM2G028980	GRMZM2G028980_P01	ARF
ZmARF17	GRMZM2G159399	GRMZM2G159399_P01	ARF
ZmARF18	GRMZM2G035405	GRMZM2G035405_P02	ARF
ZmARF19	AC207656.3_FG002	AC207656.3_FGP002	ARF
ZmARF20	GRMZM2G102845	GRMZM2G102845_P01	ARF
ZmARF21	GRMZM2G390641	GRMZM2G390641_P01	ARF
ZmARF22	GRMZM2G089640	GRMZM2G089640_P01	ARF
ZmARF23	GRMZM2G441325	GRMZM2G441325_P01	ARF
ZmARF24	GRMZM2G030710	GRMZM2G030710_P01	ARF
ZmARF25	GRMZM2G116557	GRMZM2G116557_P03	ARF
ZmARF26	GRMZM5G874163	GRMZM5G874163_P03	ARF
ZmARF27	GRMZM2G160005	GRMZM2G160005_P01	ARF
ZmARF28	GRMZM2G006042	GRMZM2G006042_P02	ARF
ZmARF29	GRMZM2G086949	GRMZM2G086949_P01	ARF
ZmARF30	GRMZM2G475882	GRMZM2G475882_P02	ARF
ZmARF31	GRMZM2G023813	GRMZM2G023813_P01	ARF

**Name**	**Genebank accession nunber**	**class**
ZmCrll1	BG873644	Crown rootless1
