# Supplementary material for: Maize protein phosphatase gene family: identification and molecular characterization
Source: BMC Genomics. 2014 Sep 9;15(1):773. doi: 10.1186/1471-2164-15-773 (PMC4169795; doi:10.1186/1471-2164-15-773)
Supplement: Supplementary file 15 — Additional file 15: Table S5: List of flowering time signaling components in maize. (PDF 16 KB) [file 12864_2014_6458_MOESM15_ESM.pdf]

Table S5. List of flowering time signaling components in maize.

Name	Gene ID	Protein ID	Class
PhyA1	GRMZM2G157727	GRMZM2G157727_P02	PhyA
PhyA2	GRMZM2G181028	GRMZM2G181028_P01	PhyA
PhyB1	GRMZM2G124532	GRMZM2G124532_P03	PhyB
PhyB2	GRMZM2G092174	GRMZM2G092174_P01	PhyB
CRY1	GRMZM2G024739	GRMZM2G024739_P01	CRY
CRY2	GRMZM2G104262	GRMZM2G104262_P01	CRY
ZmCONZ1	GRMZM2G405368	GRMZM2G405368_P01	CO
ZmZCN8	GRMZM2G179264	GRMZM2G179264_P01	FT
GIGZ1A	GRMZM2G107101	GRMZM2G107101_P06	GI
GIGZ1B	GRMZM5G844173	GRMZM5G844173_P01	GI
ZmCCA1	GRMZM2G014902	GRMZM2G014902_P01	clock gene
ZmFKF1b	GRMZM2G106363	GRMZM2G106363_P01	KFK
ZmHD6	GRMZM5G845755	GRMZM5G845755_P02	KFK
ZmLHY1	GRMZM2G474769	GRMZM2G474769_P01	LHY
ZmLHY2	GRMZM2G014902	GRMZM2G014902_P01	LHY
ZmPRR59	GRMZM2G135446	GRMZM2G135446_P02	PPR
ZmPRR73	GRMZM2G095727	GRMZM2G095727_P05	PRR7
ZmTOC1	GRMZM2G020081	GRMZM2G020081_P01	TOC1
ZFL1	GRMZM2G098813	GRMZM2G098813_P01	LFY
ZFL2	GRMZM2G180190	GRMZM2G180190_P01	LFY
ZAP1	GRMZM2G148693	GRMZM2G148693_P01	AP1
GL15	GRMZM2G160730	GRMZM2G160730_P02	AP2
DLF1	GRMZM2G067921	GRMZM2G067921_P01	FD
ZAG1	GRMZM2G052890	GRMZM2G052890_P01	AG
ZAG2	GRMZM2G160687	GRMZM2G160687_P03	AG
silky1	GRMZM2G139073	GRMZM2G139073_P01	AP3
ZMM16	GRMZM2G110153	GRMZM2G110153_P01	PI
ZMM5	GRMZM2G171365	GRMZM2G171365_P04	SOC1/AGL20
