# Supplementary material for: Maize protein phosphatase gene family: identification and molecular characterization
Source: BMC Genomics. 2014 Sep 9;15(1):773. doi: 10.1186/1471-2164-15-773 (PMC4169795; doi:10.1186/1471-2164-15-773)
Supplement: Supplementary file 16 — Additional file 16: Table S6: List of putative salt stress signaling components in maize. (PDF 341 KB) [file 12864_2014_6458_MOESM16_ESM.pdf]

Table S6. List of putative salt stress signaling components in maize.

Name	Gene ID	Protein ID	Class
ZmPLC	GRMZM5G889467	GRMZM5G889467_P01	phospholipase C
ZmPIP2-5	GRMZM2G178693	GRMZM2G178693_P01	diphosphoinositide
ZmPIP2-4	GRMZM2G154628	GRMZM2G154628_P01	diphosphoinositide
ZmPIP2-1	GRMZM2G014914	GRMZM2G014914_P01	diphosphoinositide
ZmPIP2-3	GRMZM2G125023	GRMZM2G125023_P01	diphosphoinositide
ZmPIP2-2	GRMZM2G092125	GRMZM2G092125_P01	diphosphoinositide
ZmPIP2-6	GRMZM2G047368	GRMZM2G047368_P02	diphosphoinositide
ZmPIP2-7	GRMZM2G081192	GRMZM2G081192_P01	diphosphoinositide
ZmHKT1	GRMZM2G047616	GRMZM2G047616_P01	K^+/Na^+symporter
ZmSOS1	GRMZM2G098494	GRMZM2G098494_P01	SOS
ZmSOS2	GRMZM2G137569	GRMZM2G137569_P01	SOS
ZmSOS3	GRMZM2G137751	GRMZM2G137751_P01	SOS
VP14	GRMZM2G014392	GRMZM2G014392_P01	NCED
P5CS	GRMZM2G028535	GRMZM2G028535_P01	P5C synthetase
ZmNAC001	GRMZM2G406204	GRMZM2G406204_P01	NAC
ZmNAC002	GRMZM2G025642	GRMZM2G025642_P01	NAC
ZmNAC003.1	GRMZM2G059428	GRMZM2G059428_P01	NAC
ZmNAC003.2	GRMZM2G059428	GRMZM2G059428_P03	NAC
ZmNAC004	GRMZM2G077045	GRMZM2G077045_P02	NAC
ZmNAC005	GRMZM2G031001	GRMZM2G031001_P01	NAC
ZmNAC006	GRMZM2G011598	GRMZM2G011598_P01	NAC
ZmNAC007	GRMZM2G082709	GRMZM2G082709_P01	NAC
ZmNAC008	GRMZM2G475014	GRMZM2G475014_P01	NAC
ZmNAC009.1	GRMZM2G430522	GRMZM2G430522_P01	NAC
ZmNAC009.2	GRMZM2G430522	GRMZM2G430522_P02	NAC
ZmNAC009.3	GRMZM2G430522	GRMZM2G430522_P03	NAC
ZmNAC010.1	GRMZM2G054252	GRMZM2G054252_P01	NAC
ZmNAC010.2	GRMZM2G054252	GRMZM2G054252_P02	NAC
ZmNAC011	GRMZM2G340305	GRMZM2G340305_P01	NAC
ZmNAC012	GRMZM2G152543	GRMZM2G152543_P01	NAC
ZmNAC013	GRMZM2G031120	GRMZM2G031120_P01	NAC
ZmNAC014	GRMZM2G163251	GRMZM2G163251_P01	NAC
ZmNAC015	GRMZM2G347043	GRMZM2G347043_P01	NAC
ZmNAC016	GRMZM2G156977	GRMZM2G156977_P01	NAC
ZmNAC017.1	GRMZM2G178998	GRMZM2G178998_P01	NAC
ZmNAC017.2	GRMZM2G178998	GRMZM2G178998_P02	NAC
ZmNAC018	AC212859.3_FG008	AC212859.3_FGP008	NAC
ZmNAC019.1	GRMZM2G176677	GRMZM2G176677_P01	NAC
ZmNAC019.2	GRMZM2G176677	GRMZM2G176677_P04	NAC
ZmNAC020	GRMZM2G081930	GRMZM2G081930_P01	NAC

ZmNAC021	AC208663.3_FG002	AC208663.3_FGP002	NAC
ZmNAC022.1	GRMZM2G450445	GRMZM2G450445_P01	NAC
ZmNAC022.2	GRMZM2G450445	GRMZM2G450445_P02	NAC
ZmNAC023.1	GRMZM2G009892	GRMZM2G009892_P01	NAC
ZmNAC023.2	GRMZM2G009892	GRMZM2G009892_P04	NAC
ZmNAC024	GRMZM2G099144	GRMZM2G099144_P01	NAC
ZmNAC025	GRMZM2G316840	GRMZM2G316840_P01	NAC
ZmNAC026	GRMZM2G018436	GRMZM2G018436_P01	NAC
ZmNAC027.1	GRMZM2G162739	GRMZM2G162739_P01	NAC
ZmNAC027.2	GRMZM2G162739	GRMZM2G162739_P02	NAC
ZmNAC028.1	GRMZM2G008374	GRMZM2G008374_P01	NAC
ZmNAC028.2	GRMZM2G008374	GRMZM2G008374_P02	NAC
ZmNAC029	GRMZM2G179049	GRMZM2G179049_P02	NAC
ZmNAC030	GRMZM5G803888	GRMZM5G803888_P01	NAC
ZmNAC031	GRMZM2G166721	GRMZM2G166721_P01	NAC
ZmNAC032	GRMZM2G062650	GRMZM2G062650_P01	NAC
ZmNAC033	GRMZM2G064541	GRMZM2G064541_P01	NAC
ZmNAC034	GRMZM2G114850	GRMZM2G114850_P01	NAC
ZmNAC035	GRMZM2G139700	GRMZM2G139700_P01	NAC
ZmNAC036	AC203535.4_FG002	AC203535.4_FGP002	NAC
ZmNAC037	GRMZM5G813651	GRMZM5G813651_P01	NAC
ZmNAC038.1	GRMZM2G014653	GRMZM2G014653_P01	NAC
ZmNAC038.2	GRMZM2G014653	GRMZM2G014653_P03	NAC
ZmNAC039	GRMZM5G832473	GRMZM5G832473_P01	NAC
ZmNAC040.1	GRMZM2G312201	GRMZM2G312201_P01	NAC
ZmNAC040.2	GRMZM2G312201	GRMZM2G312201_P02	NAC
ZmNAC040.3	GRMZM2G312201	GRMZM2G312201_P04	NAC
ZmNAC041	GRMZM2G122615	GRMZM2G122615_P01	NAC
ZmNAC042	GRMZM2G058518	GRMZM2G058518_P01	NAC
ZmNAC043	GRMZM2G069047	GRMZM2G069047_P01	NAC
ZmNAC044	GRMZM2G123246	GRMZM2G123246_P01	NAC
ZmNAC045	GRMZM2G100583	GRMZM2G100583_P01	NAC
ZmNAC046	GRMZM2G125777	GRMZM2G125777_P01	NAC
ZmNAC047	GRMZM2G048826	GRMZM2G048826_P01	NAC
ZmNAC048	AC198937.4_FG005	AC198937.4_FGP005	NAC
ZmNAC049	GRMZM5G898290	GRMZM5G898290_P01	NAC
ZmNAC050.1	GRMZM2G104078	GRMZM2G104078_P02	NAC
ZmNAC050.2	GRMZM2G104078	GRMZM2G104078_P03	NAC
ZmNAC051	GRMZM2G062009	GRMZM2G062009_P01	NAC
ZmNAC052	GRMZM2G354151	GRMZM2G354151_P01	NAC
ZmNAC053	GRMZM2G113950	GRMZM2G113950_P01	NAC
ZmNAC054	GRMZM2G140901	GRMZM2G140901_P01	NAC
ZmNAC055	GRMZM2G439903	GRMZM2G439903_P01	NAC

ZmNAC056.1	GRMZM2G123667	GRMZM2G123667_P02	NAC
ZmNAC056.2	GRMZM2G123667	GRMZM2G123667_P05	NAC
ZmNAC057.1	GRMZM2G336533	GRMZM2G336533_P01	NAC
ZmNAC057.2	GRMZM2G336533	GRMZM2G336533_P02	NAC
ZmNAC058.1	GRMZM2G018553	GRMZM2G018553_P01	NAC
ZmNAC058.2	GRMZM2G018553	GRMZM2G018553_P02	NAC
ZmNAC059	GRMZM2G112548	GRMZM2G112548_P01	NAC
ZmNAC060	GRMZM2G063522	GRMZM2G063522_P01	NAC
ZmNAC061	GRMZM2G389557	GRMZM2G389557_P01	NAC
ZmNAC062	GRMZM2G155816	GRMZM2G155816_P01	NAC
ZmNAC063	GRMZM2G094067	GRMZM2G094067_P01	NAC
ZmNAC064	GRMZM2G038073	GRMZM2G038073_P01	NAC
ZmNAC065	GRMZM2G315140	GRMZM2G315140_P01	NAC
ZmNAC066	GRMZM2G100593	GRMZM2G100593_P01	NAC
ZmNAC067	GRMZM5G857701	GRMZM5G857701_P01	NAC
ZmNAC068	GRMZM2G052239	GRMZM2G052239_P01	NAC
ZmNAC069	GRMZM2G030325	GRMZM2G030325_P01	NAC
ZmNAC070.1	GRMZM2G092465	GRMZM2G092465_P01	NAC
ZmNAC070.2	GRMZM2G092465	GRMZM2G092465_P03	NAC
ZmNAC071.1	GRMZM2G393433	GRMZM2G393433_P01	NAC
ZmNAC071.2	GRMZM2G393433	GRMZM2G393433_P02	NAC
ZmNAC072	GRMZM2G091490	GRMZM2G091490_P01	NAC
ZmNAC073	GRMZM2G074358	GRMZM2G074358_P01	NAC
ZmNAC074.1	GRMZM2G041746	GRMZM2G041746_P01	NAC
ZmNAC074.2	GRMZM2G041746	GRMZM2G041746_P02	NAC
ZmNAC075.1	GRMZM2G027309	GRMZM2G027309_P01	NAC
ZmNAC075.2	GRMZM2G027309	GRMZM2G027309_P02	NAC
ZmNAC076	GRMZM2G086768	GRMZM2G086768_P01	NAC
ZmNAC077	GRMZM2G379608	GRMZM2G379608_P01	NAC
ZmNAC078	GRMZM2G078954	GRMZM2G078954_P01	NAC
ZmNAC079	GRMZM2G147867	GRMZM2G147867_P01	NAC
ZmNAC080	GRMZM2G180328	GRMZM2G180328_P01	NAC
ZmNAC081	GRMZM2G456568	GRMZM2G456568_P01	NAC
ZmNAC082	GRMZM2G465835	GRMZM2G465835_P01	NAC
ZmNAC083.1	GRMZM2G031200	GRMZM2G031200_P01	NAC
ZmNAC083.2	GRMZM2G031200	GRMZM2G031200_P02	NAC
ZmNAC084	GRMZM2G033014	GRMZM2G033014_P01	NAC
ZmNAC085	GRMZM2G479980	GRMZM2G479980_P01	NAC
ZmNAC086.1	GRMZM2G079632	GRMZM2G079632_P01	NAC
ZmNAC086.2	GRMZM2G079632	GRMZM2G079632_P02	NAC
ZmNAC087	GRMZM2G159094	GRMZM2G159094_P01	NAC
ZmNAC088	GRMZM2G004531	GRMZM2G004531_P01	NAC
ZmNAC089.1	GRMZM2G386163	GRMZM2G386163_P01	NAC

ZmNAC089.2	GRMZM2G386163	GRMZM2G386163_P02	NAC
ZmNAC090.1	GRMZM2G054277	GRMZM2G054277_P01	NAC
ZmNAC090.2	GRMZM2G054277	GRMZM2G054277_P02	NAC
ZmNAC091	AC233865.1_FG003	AC233865.1_FGP003	NAC
ZmNAC092	GRMZM2G179885	GRMZM2G179885_P02	NAC
ZmNAC093	GRMZM2G430849	GRMZM2G430849_P01	NAC
ZmNAC094	GRMZM2G181605	GRMZM2G181605_P01	NAC
ZmNAC095	GRMZM2G167492	GRMZM2G167492_P01	NAC
ZmNAC096	GRMZM2G109627	GRMZM2G109627_P01	NAC
ZmNAC097.1	GRMZM2G154182	GRMZM2G154182_P01	NAC
ZmNAC097.2	GRMZM2G154182	GRMZM2G154182_P02	NAC
ZmNAC097.3	GRMZM2G154182	GRMZM2G154182_P03	NAC
ZmNAC098	GRMZM2G342647	GRMZM2G342647_P01	NAC
ZmNAC099	GRMZM2G112681	GRMZM2G112681_P01	NAC
ZmNAC100	GRMZM2G172264	GRMZM2G172264_P01	NAC
ZmNAC101	GRMZM2G104400	GRMZM2G104400_P01	NAC
ZmNAC102	GRMZM2G134687	GRMZM2G134687_P01	NAC
ZmNAC103	GRMZM2G163843	GRMZM2G163843_P01	NAC
ZmNAC104	GRMZM2G163841	GRMZM2G163841_P01	NAC
ZmNAC105	GRMZM2G134073	GRMZM2G134073_P01	NAC
ZmNAC106	GRMZM2G068973	GRMZM2G068973_P01	NAC
ZmNAC107	GRMZM2G171395	GRMZM2G171395_P01	NAC
ZmNAC108	GRMZM2G041668	GRMZM2G041668_P01	NAC
ZmNAC109	GRMZM2G440219	GRMZM2G440219_P01	NAC
ZmNAC110	GRMZM2G459156	GRMZM2G459156_P01	NAC
ZmNAC111.1	GRMZM2G163914	GRMZM2G163914_P02	NAC
ZmNAC111.2	GRMZM2G163914	GRMZM2G163914_P03	NAC
ZmNAC112	GRMZM2G115721	GRMZM2G115721_P01	NAC
ZmNAC113	GRMZM2G104074	GRMZM2G104074_P01	NAC
ZmNAC114	GRMZM2G042494	GRMZM2G042494_P01	NAC
ZmNAC115.1	GRMZM2G159500	GRMZM2G159500_P01	NAC
ZmNAC115.2	GRMZM2G159500	GRMZM2G159500_P02	NAC
ZmNAC116	GRMZM5G894234	GRMZM5G894234_P01	NAC
ZmNAC117	GRMZM2G126817	GRMZM2G126817_P01	NAC
ZmNAC118	GRMZM2G174070	GRMZM2G174070_P01	NAC
ZmNAC119	GRMZM2G127379	GRMZM2G127379_P01	NAC
ZmNAC120	GRMZM2G083347	GRMZM2G083347_P01	NAC
ZmNAC121	GRMZM2G167018	GRMZM2G167018_P01	NAC
ZmNAC122	GRMZM2G003715	GRMZM2G003715_P01	NAC
ZmNAC123	GRMZM2G435824	GRMZM2G435824_P01	NAC
ZmNAC124	GRMZM2G111770	GRMZM2G111770_P01	NAC
ZmNAC125	GRMZM2G043813	GRMZM2G043813_P01	NAC
ZmMYB001	AC217264.3_FG005	AC217264.3_FGP005	MYB

ZmMYB002	GRMZM2G024468	GRMZM2G024468_P01	MYB
ZmMYB003	GRMZM2G037650	GRMZM2G037650_P01	MYB
ZmMYB004	GRMZM2G046443	GRMZM2G046443_P01	MYB
ZmMYB005	GRMZM2G054111	GRMZM2G054111_P01	MYB
ZmMYB006	GRMZM2G057027	GRMZM2G057027_P02	MYB
ZmMYB007	GRMZM2G070849	GRMZM2G070849_P01	MYB
ZmMYB008	GRMZM2G077147	GRMZM2G077147_P01	MYB
ZmMYB009	GRMZM2G079123	GRMZM2G079123_P01	MYB
ZmMYB010	GRMZM2G084583	GRMZM2G084583_P01	MYB
ZmMYB011	GRMZM2G084799	GRMZM2G084799_P01	MYB
ZmMYB012	GRMZM2G106558	GRMZM2G106558_P02	MYB
ZmMYB013	GRMZM2G110135	GRMZM2G110135_P01	MYB
ZmMYB014	GRMZM2G121570	GRMZM2G121570_P01	MYB
ZmMYB015	GRMZM2G130149	GRMZM2G130149_P01	MYB
ZmMYB016	GRMZM2G131937	GRMZM2G131937_P01	MYB
ZmMYB017	GRMZM2G143046	GRMZM2G143046_P01	MYB
ZmMYB018	GRMZM2G147346	GRMZM2G147346_P01	MYB
ZmMYB019	GRMZM2G147698	GRMZM2G147698_P01	MYB
ZmMYB020	GRMZM2G308034	GRMZM2G308034_P01	MYB
ZmMYB021	GRMZM2G403620	GRMZM2G403620_P01	MYB
ZmMYB022	GRMZM2G428555	GRMZM2G428555_P01	MYB
ZmMYB023	GRMZM5G870592	GRMZM5G870592_P01	MYB
ZmMYB024	AC165178.2_FG004	AC165178.2_FGP004	MYB
ZmMYB025	GRMZM2G032655	GRMZM2G032655_P01	MYB
ZmMYB026	GRMZM2G038722	GRMZM2G038722_P01	MYB
ZmMYB027	GRMZM2G048295	GRMZM2G048295_P01	MYB
ZmMYB028	GRMZM2G050305	GRMZM2G050305_P01	MYB
ZmMYB029	GRMZM2G064630	GRMZM2G064630_P01	MYB
ZmMYB030	GRMZM2G087955	GRMZM2G087955_P01	MYB
ZmMYB031	GRMZM2G090837	GRMZM2G090837_P01	MYB
ZmMYB032	GRMZM2G105137	GRMZM2G105137_P01	MYB
ZmMYB033	GRMZM2G115859	GRMZM2G115859_P01	MYB
ZmMYB034	GRMZM2G123202	GRMZM2G123202_P01	MYB
ZmMYB035	GRMZM2G124715	GRMZM2G124715_P01	MYB
ZmMYB036	GRMZM2G139284	GRMZM2G139284_P01	MYB
ZmMYB037	GRMZM2G166337	GRMZM2G166337_P01	MYB
ZmMYB038	GRMZM2G176327	GRMZM2G176327_P01	MYB
ZmMYB039	GRMZM2G001875	GRMZM2G001875_P01	MYB
ZmMYB040	GRMZM2G017520	GRMZM2G017520_P01	MYB
ZmMYB041	GRMZM2G041415	GRMZM2G041415_P01	MYB
ZmMYB042	GRMZM2G047626	GRMZM2G047626_P01	MYB
ZmMYB043	GRMZM2G051256	GRMZM2G051256_P01	MYB
ZmMYB044	GRMZM2G052377	GRMZM2G052377_P01	MYB

ZmMYB045	GRMZM2G064744	GRMZM2G064744_P01	MYB
ZmMYB046	GRMZM2G083239	GRMZM2G083239_P01	MYB
ZmMYB047	GRMZM2G088783	GRMZM2G088783_P01	MYB
ZmMYB048	GRMZM2G111731	GRMZM2G111731_P01	MYB
ZmMYB049	GRMZM2G139688	GRMZM2G139688_P01	MYB
ZmMYB050	GRMZM2G143328	GRMZM2G143328_P01	MYB
ZmMYB051	GRMZM2G158700	GRMZM2G158700_P01	MYB
ZmMYB052	GRMZM2G160838	GRMZM2G160838_P01	MYB
ZmMYB053	GRMZM2G160840	GRMZM2G160840_P01	MYB
ZmMYB054	GRMZM2G162709	GRMZM2G162709_P01	MYB
ZmMYB055	GRMZM2G167829	GRMZM2G167829_P01	MYB
ZmMYB056	GRMZM2G369799	GRMZM2G369799_P01	MYB
ZmMYB057	GRMZM2G460869	GRMZM2G460869_P01	MYB
ZmMYB058	GRMZM2G470307	GRMZM2G470307_P01	MYB
ZmMYB059	GRMZM5G803355	GRMZM5G803355_P01	MYB
ZmMYB060	GRMZM2G011422	GRMZM2G011422_P01	MYB
ZmMYB061	GRMZM2G015021	GRMZM2G015021_P01	MYB
ZmMYB062	GRMZM2G017268	GRMZM2G017268_P01	MYB
ZmMYB063	GRMZM2G043792	GRMZM2G043792_P01	MYB
ZmMYB064	GRMZM2G055158	GRMZM2G055158_P01	MYB
ZmMYB065	GRMZM2G089244	GRMZM2G089244_P01	MYB
ZmMYB066	GRMZM2G108959	GRMZM2G108959_P01	MYB
ZmMYB067	GRMZM2G111045	GRMZM2G111045_P01	MYB
ZmMYB068	GRMZM2G111117	GRMZM2G111117_P01	MYB
ZmMYB069	GRMZM2G127857	GRMZM2G127857_P01	MYB
ZmMYB070	GRMZM2G131442	GRMZM2G131442_P01	MYB
ZmMYB071	GRMZM2G138427	GRMZM2G138427_P01	MYB
ZmMYB072	GRMZM2G162434	GRMZM2G162434_P01	MYB
ZmMYB073	GRMZM2G419239	GRMZM2G419239_P01	MYB
ZmMYB074	GRMZM2G496770	GRMZM2G496770_P01	MYB
ZmMYB075	GRMZM5G833253	GRMZM5G833253_P01	MYB
ZmMYB076	GRMZM2G048136	GRMZM2G048136_P01	MYB
ZmMYB077	GRMZM2G001223	GRMZM2G001223_P03	MYB
ZmMYB078	GRMZM2G027697	GRMZM2G027697_P01	MYB
ZmMYB079	GRMZM2G040924	GRMZM2G040924_P01	MYB
ZmMYB080	GRMZM2G070523	GRMZM2G070523_P01	MYB
ZmMYB081	GRMZM2G073836	GRMZM2G073836_P01	MYB
ZmMYB082	GRMZM2G088189	GRMZM2G088189_P01	MYB
ZmMYB083	GRMZM2G095904	GRMZM2G095904_P01	MYB
ZmMYB084	GRMZM2G104789	GRMZM2G104789_P01	MYB
ZmMYB085	GRMZM2G145444	GRMZM2G145444_P01	MYB
ZmMYB086	GRMZM2G159547	GRMZM2G159547_P01	MYB
ZmMYB087	GRMZM2G161512	GRMZM2G161512_P01	MYB

ZmMYB088	GRMZM2G170049	GRMZM2G170049_P01	MYB
ZmMYB089	GRMZM2G302549	GRMZM2G302549_P01	MYB
ZmMYB090	GRMZM2G455869	GRMZM2G455869_P01	MYB
ZmMYB091	GRMZM2G013581	GRMZM2G013581_P01	MYB
ZmMYB092	GRMZM2G048910	GRMZM2G048910_P01	MYB
ZmMYB093	GRMZM2G069325	GRMZM2G069325_P02	MYB
ZmMYB094	GRMZM2G077789	GRMZM2G077789_P01	MYB
ZmMYB095	GRMZM2G078820	GRMZM2G078820_P01	MYB
ZmMYB096	GRMZM2G093647	GRMZM2G093647_P01	MYB
ZmMYB097	GRMZM2G093660	GRMZM2G093660_P01	MYB
ZmMYB098	GRMZM2G093789	GRMZM2G093789_P01	MYB
ZmMYB099	GRMZM2G102790	GRMZM2G102790_P01	MYB
ZmMYB100	GRMZM2G175232	GRMZM2G175232_P01	MYB
ZmMYB101	GRMZM2G305856	GRMZM2G305856_P01	MYB
ZmMYB102	GRMZM2G343068	GRMZM2G343068_P01	MYB
ZmMYB103	GRMZM2G423833	GRMZM2G423833_P01	MYB
ZmMYB104	GRMZM2G701063	GRMZM2G701063_P01	MYB
ZmMYB105	AC213884.3_FG002	AC213884.3_FGP002	MYB
ZmMYB106	GRMZM2G000818	GRMZM2G000818_P01	MYB
ZmMYB107	GRMZM2G031323	GRMZM2G031323_P01	MYB
ZmMYB108	GRMZM2G045748	GRMZM2G045748_P01	MYB
ZmMYB109	GRMZM2G050550	GRMZM2G050550_P01	MYB
ZmMYB110	GRMZM2G056407	GRMZM2G056407_P01	MYB
ZmMYB111	GRMZM2G104551	GRMZM2G104551_P01	MYB
ZmMYB112	GRMZM2G117244	GRMZM2G117244_P01	MYB
ZmMYB113	GRMZM2G126566	GRMZM2G126566_P01	MYB
ZmMYB114	GRMZM2G150841	GRMZM2G150841_P01	MYB
ZmMYB115	GRMZM2G169356	GRMZM2G169356_P01	MYB
ZmMYB116	GRMZM2G172327	GRMZM2G172327_P01	MYB
ZmMYB117	GRMZM2G003406	GRMZM2G003406_P01	MYB
ZmMYB118	GRMZM2G006352	GRMZM2G006352_P01	MYB
ZmMYB119	GRMZM2G028054	GRMZM2G028054_P01	MYB
ZmMYB120	GRMZM2G047600	GRMZM2G047600_P01	MYB
ZmMYB121	GRMZM2G051528	GRMZM2G051528_P01	MYB
ZmMYB122	GRMZM2G096358	GRMZM2G096358_P01	MYB
ZmMYB123	GRMZM2G119693	GRMZM2G119693_P01	MYB
ZmMYB124	GRMZM2G151205	GRMZM2G151205_P01	MYB
ZmMYB125	GRMZM2G169316	GRMZM2G169316_P01	MYB
ZmMYB126	GRMZM2G171781	GRMZM2G171781_P01	MYB
ZmMYB127	GRMZM2G312419	GRMZM2G312419_P01	MYB
ZmMYB128	GRMZM2G322490	GRMZM2G322490_P01	MYB
ZmMYB129	GRMZM2G330475	GRMZM2G330475_P01	MYB
ZmMYB130	GRMZM2G395672	GRMZM2G395672_P01	MYB

ZmMYB131	GRMZM2G405094	GRMZM2G405094_P01	MYB
ZmMYB132	GRMZM2G431156	GRMZM2G431156_P01	MYB
ZmMYB133	GRMZM2G004090	GRMZM2G004090_P01	MYB
ZmMYB134	GRMZM2G005066	GRMZM2G005066_P01	MYB
ZmMYB135	GRMZM2G022686	GRMZM2G022686_P01	MYB
ZmMYB136	GRMZM2G044824	GRMZM2G044824_P01	MYB
ZmMYB137	GRMZM2G089686	GRMZM2G089686_P01	MYB
ZmMYB138	GRMZM2G098179	GRMZM2G098179_P01	MYB
ZmMYB139	GRMZM2G134279	GRMZM2G134279_P01	MYB
ZmMYB140	GRMZM2G167088	GRMZM2G167088_P01	MYB
ZmMYB141	GRMZM2G416652	GRMZM2G416652_P01	MYB
ZmMYB142	GRMZM5G803308	GRMZM5G803308_P01	MYB
ZmMYB143	AC197146.3_FG002	AC197146.3_FGP002	MYB
ZmMYB144	AC206901.3_FG005	AC206901.3_FGP005	MYB
ZmMYB145	GRMZM2G001824	GRMZM2G001824_P01	MYB
ZmMYB146	GRMZM2G052606	GRMZM2G052606_P01	MYB
ZmMYB147	GRMZM2G081557	GRMZM2G081557_P01	MYB
ZmMYB148	GRMZM2G097636	GRMZM2G097636_P01	MYB
ZmMYB149	GRMZM2G097638	GRMZM2G097638_P01	MYB
ZmMYB150	GRMZM2G127490	GRMZM2G127490_P01	MYB
ZmMYB151	GRMZM2G150680	GRMZM2G150680_P01	MYB
ZmMYB152	GRMZM2G172487	GRMZM2G172487_P01	MYB
ZmMYB153	GRMZM2G172575	GRMZM2G172575_P01	MYB
ZmMYB154	GRMZM2G173633	GRMZM2G173633_P01	MYB
ZmMYB155	GRMZM2G311059	GRMZM2G311059_P01	MYB
ZmMYB156	GRMZM2G325907	GRMZM2G325907_P01	MYB
ZmMYB157	GRMZM2G425427	GRMZM2G425427_P01	MYB
ZmMYC1	GRMZM2G057413	GRMZM2G057413_P01	MYC
ZmMYC2	AC193786.3_FG005	AC193786.3_FGP005	MYC
ZmMYC3	GRMZM2G089501	GRMZM2G089501_P01	MYC
ZmMYC4	GRMZM2G009478	GRMZM2G009478_P05	MYC
ZmMYC5	GRMZM2G317450	GRMZM2G317450_P03	MYC
ZmMYC6	GRMZM2G114444	GRMZM2G114444_P02	MYC
ZmMYC7	GRMZM2G001930	GRMZM2G001930_P01	MYC
ZmMYC8	GRMZM2G049229	GRMZM2G049229_P01	MYC
ZmDREB1.10	GRMZM2G042756	GRMZM2G042756_P01	DREB
ZmDREB1.2	GRMZM2G069082	GRMZM2G069082_P01	DREB
ZmDREB1.3	GRMZM2G069146	GRMZM2G069146_P01	DREB
ZmDREB1.4	GRMZM2G124011	GRMZM2G124011_P01	DREB
ZmDREB1.5	GRMZM2G069126	GRMZM2G069126_P01	DREB
ZmDREB1.6	GRMZM2G175856	GRMZM2G175856_P01	DREB
ZmDREB1.7	GRMZM2G380377	GRMZM2G380377_P01	DREB
ZmDREB1.8	GRMZM2G097182	GRMZM2G097182_P01	DREB

ZmDREB1.9	GRMZM2G137341	GRMZM2G137341_P01	DREB
ZmDREB1.1/1A	GRMZM2G124037	GRMZM2G124037_P01	DREB
ZmDREB2.2	AC209257.4_FG006	AC209257.4_FGP006	DREB
ZmDREB2.3/ZmABI4	GRMZM2G093595	GRMZM2G093595_P01	DREB
ZmDREB2.4	GRMZM2G419901	GRMZM2G419901_P01	DREB
ZmDREB2.5	GRMZM2G376255	GRMZM2G376255_P01	DREB
ZmDREB2.6	GRMZM2G399098	GRMZM2G399098_P01	DREB
ZmDREB2.7	GRMZM2G028386	GRMZM2G028386_P01	DREB
ZmDREB2.8	GRMZM2G156737	GRMZM2G156737_P01	DREB
ZmDREB2.1/2A	GRMZM2G006745	GRMZM2G006745_P01	DREB
ZmDREB2-19	GRMZM2G323172	GRMZM2G323172_P01	DREB
ZmDREB2-20	GRMZM2G348307	GRMZM2G348307_P01	DREB
GRMZM2G106921	GRMZM2G106921	GRMZM2G106921_P01	SLAC1 anion channel
GRMZM2G331393	GRMZM2G331393	GRMZM2G331393_P01	SLAC1 anion channel
GRMZM2G518198	GRMZM2G518198	GRMZM2G518198_P01	SLAC1 anion channel
GRMZM2G362163	GRMZM2G362163	GRMZM2G362163_P01	SLAC1 anion channel
GRMZM2G074540	GRMZM2G074540	GRMZM2G074540_P01	SLAC1 anion channel
GRMZM2G080887	GRMZM2G080887	GRMZM2G080887_P01	SLAC1 anion channel
GRMZM2G061469	GRMZM2G061469	GRMZM2G061469_P01	SLAC1 anion channel
GRMZM2G134628	GRMZM2G134628	GRMZM2G134628_P01	SLAC1 anion channel
GRMZM2G059453	GRMZM2G059453	GRMZM2G059453_P01	SLAC1 anion channel
GRMZM2G308615	GRMZM2G308615	GRMZM2G308615_P01	SLAC1 anion channel
GRMZM2G122228	GRMZM2G122228	GRMZM2G122228_P01	SLAC1 anion channel
KZM1	GRMZM2G178356	GRMZM2G178356_P01	K+ channel
KZM2	GRMZM2G093313	GRMZM2G093313_P02	K+ channel
ZmRbohA	GRMZM2G089291	GRMZM2G089291_P01	NADPH oxidase
ZmRbohB	GRMZM2G448185	GRMZM2G448185_P01	NADPH oxidase
ZmRbohC	GRMZM2G065144	GRMZM2G065144_P01	NADPH oxidase
ZmRbohD	GRMZM2G358619	GRMZM2G358619_P01	NADPH oxidase
ZmRbohE	GRMZM2G037993	GRMZM2G037993_P01	NADPH oxidase
ZmRbohF	GRMZM2G034896	GRMZM2G034896_P01	NADPH oxidase
ZmRbohG	GRMZM2G401179	GRMZM2G401179_P01	NADPH oxidase
ZmRbohH	GRMZM2G426953	GRMZM2G426953_P01	NADPH oxidase
ZmRbohI	GRMZM2G138152	GRMZM2G138152_P02	NADPH oxidase
ZmRbohJ	GRMZM2G441541	GRMZM2G441541_P02	NADPH oxidase
ZmRbohK	GRMZM2G043435	GRMZM2G043435_P01	NADPH oxidase
ZmRbohL	GRMZM2G147966	GRMZM2G147966_P01	NADPH oxidase
ZmRbohM	GRMZM2G022547	GRMZM2G022547_P02	NADPH oxidase
ZmRbohN	GRMZM2G323731	GRMZM2G323731_P01	NADPH oxidase
ZmRbohO	GRMZM2G300965	GRMZM2G300965_P01	NADPH oxidase
ZmA1	GRMZM2G008122	GRMZM2G008122_P01	V-H^+ATPase
ZmA2	GRMZM2G068259	GRMZM2G068259_P01	V-H^+ATPase
ZmA3	GRMZM2G019404	GRMZM2G019404_P01	V-H^+ATPase

ZmA4	GRMZM2G144821	GRMZM2G144821_P01	V-H^+ATPase
ZmA5	GRMZM2G035520	GRMZM2G035520_P01	V-H^+ATPase
ZmA6	GRMZM2G341058	GRMZM2G341058_P01	V-H^+ATPase
ZmA7	GRMZM2G148374	GRMZM2G148374_P01	V-H^+ATPase
ZmA8	AC209050.3_FG001	AC209050.3_FGP001	V-H^+ATPase
ZmA9	GRMZM2G104325	GRMZM2G104325_P01	V-H^+ATPase
ZmA10	GRMZM2G455557	GRMZM2G455557_P01	V-H^+ATPase
ZmA11	GRMZM2G131309	GRMZM2G131309_P01	V-H^+ATPase
ZmA12	GRMZM2G006894	GRMZM2G006894_P01	V-H^+ATPase
ZmSnRK2.1	GRMZM2G180916	GRMZM2G180916_P04	SnRK2
ZmSnRK2.2	GRMZM2G081915	GRMZM2G081915_P02	SnRK2
ZmSnRK2.3	GRMZM2G138861	GRMZM2G138861_P04	SnRK2
ZmSnRK2.4	GRMZM2G155593	GRMZM2G155593_P01	SnRK2
ZmSnRK2.5	GRMZM2G110922	GRMZM2G110922_P01	SnRK2
ZmSnRK2.6	GRMZM2G130018	GRMZM2G130018_P01	SnRK2
ZmSnRK2.7	GRMZM2G171435	GRMZM2G171435_P01	SnRK2
ZmSnRK2.8	GRMZM2G066867	GRMZM2G066867_P01	SnRK2
ZmSnRK2.9	GRMZM2G000278	GRMZM2G000278_P03	SnRK2
ZmSnRK2.10	GRMZM2G063961	GRMZM2G063961_P01	SnRK2
ZmSnRK2.11	GRMZM2G056732	GRMZM2G056732_P02	SnRK2
ZmSnRK2.12	GRMZM2G334791	GRMZM2G334791_P03	SnRK2
ZmSnRK2.13	GRMZM2G035809	GRMZM2G035809_P01	SnRK2
ZmSnRK2.14	GRMZM2G110908	GRMZM2G110908_P01	SnRK2
GRMZM2G120517	GRMZM2G120517	GRMZM2G120517_P02	APX
GRMZM2G460406	GRMZM2G460406	GRMZM2G460406_P01	APX
GRMZM2G006791	GRMZM2G006791	GRMZM2G006791_P04	APX
GRMZM2G140667	GRMZM2G140667	GRMZM2G140667_P01	APX
GRMZM2G004211	GRMZM2G004211	GRMZM2G004211_P01	APX
GRMZM2G137839	GRMZM2G137839	GRMZM2G137839_P01	APX
GRMZM2G054300	GRMZM2G054300	GRMZM2G054300_P04	APX
GRMZM2G014397	GRMZM2G014397	GRMZM2G014397_P03	APX
ZmbZIP1	GRMZM2G428184	GRMZM2G428184_P01	bZIP
ZmbZIP2	GRMZM2G157177	GRMZM2G157177_P01	bZIP
ZmbZIP3	GRMZM2G038015	GRMZM2G038015_P01	bZIP
ZmbZIP4	GRMZM2G174284	GRMZM2G174284_P01	bZIP
ZmbZIP5	GRMZM2G062391	GRMZM2G062391_P01	bZIP
ZmbZIP6	GRMZM2G353553	GRMZM2G353553_P01	bZIP
ZmbZIP7	GRMZM2G093020	GRMZM2G093020_P02	bZIP
ZmbZIP8	GRMZM2G479885	GRMZM2G479885_P01	bZIP
ZmbZIP9.1	GRMZM2G073427	GRMZM2G073427_P04	bZIP
ZmbZIP9.2	GRMZM2G073427	GRMZM2G073427_P02	bZIP
ZmbZIP9.3	GRMZM2G073427	GRMZM2G073427_P03	bZIP
ZmbZIP9.4	GRMZM2G073427	GRMZM2G073427_P07	bZIP

ZmbZIP10	GRMZM2G370026	GRMZM2G370026_P01	bZIP
ZmbZIP11	AC186606.4_FG003	AC186606.4_FGP003	bZIP
ZmbZIP12	GRMZM2G332294	GRMZM2G332294_P01	bZIP
ZmbZIP13	GRMZM2G175280	GRMZM2G175280_P01	bZIP
ZmbZIP14	GRMZM2G478417	GRMZM2G478417_P01	bZIP
ZmbZIP15	GRMZM2G177046	GRMZM2G177046_P01	bZIP
ZmbZIP16.1	GRMZM2G019446	GRMZM2G019446_P02	bZIP
ZmbZIP16.2	GRMZM2G019446	GRMZM2G019446_P01	bZIP
ZmbZIP17	GRMZM2G016150	GRMZM2G016150_P01	bZIP
ZmbZIP18.1	GRMZM2G131961	GRMZM2G131961_P01	bZIP
ZmbZIP18.2	GRMZM2G131961	GRMZM2G131961_P03	bZIP
ZmbZIP18.3	GRMZM2G131961	GRMZM2G131961_P05	bZIP
ZmbZIP19.1	GRMZM2G112483	GRMZM2G112483_P01	bZIP
ZmbZIP19.2	GRMZM2G112483	GRMZM2G112483_P02	bZIP
ZmbZIP20.1	GRMZM5G821024	GRMZM5G821024_P01	bZIP
ZmbZIP20.2	GRMZM5G821024	GRMZM5G821024_P02	bZIP
ZmbZIP21.1	GRMZM2G171370	GRMZM2G171370_P03	bZIP
ZmbZIP21.2	GRMZM2G171370	GRMZM2G171370_P01	bZIP
ZmbZIP22	AC203957.3_FG004	AC203957.3_FGP004	bZIP
ZmbZIP23	GRMZM2G002075	GRMZM2G002075_P01	bZIP
ZmbZIP24	GRMZM2G137532	GRMZM2G137532_P01	bZIP
ZmbZIP25	GRMZM2G125934	GRMZM2G125934_P01	bZIP
ZmbZIP26	GRMZM2G180847	GRMZM2G180847_P01	bZIP
ZmbZIP27	GRMZM2G037910	GRMZM2G037910_P01	bZIP
ZmbZIP28	GRMZM2G060109	GRMZM2G060109_P01	bZIP
ZmbZIP29	GRMZM2G166566	GRMZM2G166566_P01	bZIP
ZmbZIP30	AC233853.1_FG002	AC233853.1_FGP002	bZIP
ZmbZIP31	GRMZM2G039828	GRMZM2G039828_P01	bZIP
ZmbZIP32	GRMZM2G160136	GRMZM2G160136_P02	bZIP
ZmbZIP33	GRMZM2G074373	GRMZM2G074373_P01	bZIP
ZmbZIP34	GRMZM2G160902	GRMZM2G160902_P01	bZIP
ZmbZIP35	GRMZM2G024851	GRMZM2G024851_P01	bZIP
ZmbZIP36.1	GRMZM2G146020	GRMZM2G146020_P01	bZIP
ZmbZIP36.2	GRMZM2G146020	GRMZM2G146020_P02	bZIP
ZmbZIP37.1	GRMZM5G858197	GRMZM5G858197_P02	bZIP
ZmbZIP37.2	GRMZM5G858197	GRMZM5G858197_P03	bZIP
ZmbZIP38.1	GRMZM2G060216	GRMZM2G060216_P01	bZIP
ZmbZIP38.2	GRMZM2G060216	GRMZM2G060216_P02	bZIP
ZmbZIP39	GRMZM2G159134	GRMZM2G159134_P02	bZIP
ZmbZIP40.1	GRMZM2G019907	GRMZM2G019907_P02	bZIP
ZmbZIP40.2	GRMZM2G019907	GRMZM2G019907_P03	bZIP
ZmbZIP41	GRMZM2G027976	GRMZM2G027976_P01	bZIP
ZmbZIP42	GRMZM5G848942	GRMZM5G848942_P01	bZIP

ZmbZIP43	GRMZM2G117851	GRMZM2G117851_P01	bZIP
ZmbZIP44	GRMZM2G368491	GRMZM2G368491_P01	bZIP
ZmbZIP45	GRMZM2G120167	GRMZM2G120167_P01	bZIP
ZmbZIP46.1	GRMZM2G157722	GRMZM2G157722_P02	bZIP
ZmbZIP46.2	GRMZM2G157722	GRMZM2G157722_P01	bZIP
ZmbZIP47	GRMZM2G118870	GRMZM2G118870_P01	bZIP
ZmbZIP48	GRMZM2G380897	GRMZM2G380897_P01	bZIP
ZmbZIP49	GRMZM2G122846	GRMZM2G122846_P01	bZIP
ZmbZIP50	GRMZM2G030877	GRMZM2G030877_P01	bZIP
ZmbZIP51	GRMZM2G011119	GRMZM2G011119_P01	bZIP
ZmbZIP52	AC190609.3_FG001	AC190609.3_FGP001	bZIP
ZmbZIP53	GRMZM2G149150	GRMZM2G149150_P01	bZIP
ZmbZIP54	GRMZM2G144480	GRMZM2G144480_P01	bZIP
ZmbZIP55	GRMZM2G079365	GRMZM2G079365_P01	bZIP
ZmbZIP56	GRMZM2G052102	GRMZM2G052102_P01	bZIP
ZmbZIP57	GRMZM2G336766	GRMZM2G336766_P01	bZIP
ZmbZIP58	GRMZM2G019106	GRMZM2G019106_P01	bZIP
ZmbZIP59	GRMZM2G361611	GRMZM2G361611_P01	bZIP
ZmbZIP60.1	GRMZM2G007063	GRMZM2G007063_P01	bZIP
ZmbZIP60.2	GRMZM2G007063	GRMZM2G007063_P04	bZIP
ZmbZIP61	GRMZM2G386273	GRMZM2G386273_P01	bZIP
ZmbZIP62.1	GRMZM2G000171	GRMZM2G000171_P02	bZIP
ZmbZIP62.2	GRMZM2G000171	GRMZM2G000171_P01	bZIP
ZmbZIP63	GRMZM2G153144	GRMZM2G153144_P01	bZIP
ZmbZIP64	GRMZM2G444748	GRMZM2G444748_P01	bZIP
ZmbZIP65.1	GRMZM2G098904	GRMZM2G098904_P01	bZIP
ZmbZIP65.2	GRMZM2G098904	GRMZM2G098904_P02	bZIP
ZmbZIP66	GRMZM2G080111	GRMZM2G080111_P01	bZIP
ZmbZIP67	GRMZM2G137046	GRMZM2G137046_P01	bZIP
ZmbZIP68	GRMZM2G029979	GRMZM2G029979_P01	bZIP
ZmbZIP69	GRMZM2G158313	GRMZM2G158313_P01	bZIP
ZmbZIP70	GRMZM2G092609	GRMZM2G092609_P01	bZIP
ZmbZIP71	GRMZM2G111504	GRMZM2G111504_P01	bZIP
ZmbZIP72	GRMZM2G020799	GRMZM2G020799_P01	bZIP
ZmbZIP73	GRMZM2G073892	GRMZM2G073892_P01	bZIP
ZmbZIP74	GRMZM2G448607	GRMZM2G448607_P01	bZIP
ZmbZIP75	GRMZM2G402862	GRMZM2G402862_P01	bZIP
ZmbZIP76	GRMZM2G055413	GRMZM2G055413_P01	bZIP
ZmbZIP77.1	GRMZM2G133331	GRMZM2G133331_P01	bZIP
ZmbZIP77.2	GRMZM2G133331	GRMZM2G133331_P02	bZIP
ZmbZIP77.3	GRMZM2G133331	GRMZM2G133331_P03	bZIP
ZmbZIP78	GRMZM2G396632	GRMZM2G396632_P01	bZIP
ZmbZIP79.1	GRMZM2G045236	GRMZM2G045236_P01	bZIP

ZmbZIP79.2	GRMZM2G045236	GRMZM2G045236_P02	bZIP
ZmbZIP80.1	GRMZM2G000842	GRMZM2G000842_P01	bZIP
ZmbZIP80.2	GRMZM2G000842	GRMZM2G000842_P02	bZIP
ZmbZIP80.3	GRMZM2G000842	GRMZM2G000842_P03	bZIP
ZmbZIP81.1	GRMZM2G129247	GRMZM2G129247_P02	bZIP
ZmbZIP81.2	GRMZM2G129247	GRMZM2G129247_P03	bZIP
ZmbZIP82	GRMZM2G175870	GRMZM2G175870_P01	bZIP
ZmbZIP83.1	GRMZM2G011932	GRMZM2G011932_P01	bZIP
ZmbZIP83.2	GRMZM2G011932	GRMZM2G011932_P04	bZIP
ZmbZIP84	GRMZM2G438652	GRMZM2G438652_P01	bZIP
ZmbZIP85.1	GRMZM2G015534	GRMZM2G015534_P02	bZIP
ZmbZIP85.2	GRMZM2G015534	GRMZM2G015534_P01	bZIP
ZmbZIP85.3	GRMZM2G015534	GRMZM2G015534_P03	bZIP
ZmbZIP86.1	GRMZM2G095078	GRMZM2G095078_P01	bZIP
ZmbZIP86.2	GRMZM2G095078	GRMZM2G095078_P02	bZIP
ZmbZIP87	GRMZM2G092137	GRMZM2G092137_P01	bZIP
ZmbZIP88	GRMZM2G358796	GRMZM2G358796_P01	bZIP
ZmbZIP89.1	GRMZM2G006578	GRMZM2G006578_P01	bZIP
ZmbZIP89.2	GRMZM2G006578	GRMZM2G006578_P02	bZIP
ZmbZIP89.3	GRMZM2G006578	GRMZM2G006578_P03	bZIP
ZmbZIP90	AC200057.4_FG007	AC200057.4_FGP007	bZIP
ZmbZIP91	GRMZM2G043600	GRMZM2G043600_P01	bZIP
ZmbZIP92	GRMZM2G149040	GRMZM2G149040_P01	bZIP
ZmbZIP93.1	GRMZM2G361847	GRMZM2G361847_P04	bZIP
ZmbZIP93.2	GRMZM2G361847	GRMZM2G361847_P01	bZIP
ZmbZIP93.3	GRMZM2G361847	GRMZM2G361847_P02	bZIP
ZmbZIP93.4	GRMZM2G361847	GRMZM2G361847_P07	bZIP
ZmbZIP94	GRMZM2G077124	GRMZM2G077124_P03	bZIP
ZmbZIP95	GRMZM2G067921	GRMZM2G067921_P01	bZIP
ZmbZIP96.1	GRMZM2G030280	GRMZM2G030280_P01	bZIP
ZmbZIP96.2	GRMZM2G030280	GRMZM2G030280_P02	bZIP
ZmbZIP97.1	GRMZM2G171912	GRMZM2G171912_P01	bZIP
ZmbZIP97.2	GRMZM2G171912	GRMZM2G171912_P02	bZIP
ZmbZIP98	GRMZM2G132868	GRMZM2G132868_P01	bZIP
ZmbZIP99	GRMZM2G161009	GRMZM2G161009_P01	bZIP
ZmbZIP100.1	GRMZM2G080731	GRMZM2G080731_P01	bZIP
ZmbZIP100.2	GRMZM2G080731	GRMZM2G080731_P02	bZIP
ZmbZIP100.3	GRMZM2G080731	GRMZM2G080731_P03	bZIP
ZmbZIP101.1	GRMZM2G033230	GRMZM2G033230_P01	bZIP
ZmbZIP101.2	GRMZM2G033230	GRMZM2G033230_P02	bZIP
ZmbZIP102	GRMZM2G438293	GRMZM2G438293_P01	bZIP
ZmbZIP103	AC232238.2_FG004	AC232238.2_FGP004	bZIP
ZmbZIP104	GRMZM2G168079	GRMZM2G168079_P01	bZIP

ZmbZIP105	GRMZM2G125243	GRMZM2G125243_P01	bZIP
ZmbZIP106.1	GRMZM2G170079	GRMZM2G170079_P02	bZIP
ZmbZIP106.2	GRMZM2G170079	GRMZM2G170079_P01	bZIP
ZmbZIP107	GRMZM2G033413	GRMZM2G033413_P01	bZIP
ZmbZIP108	GRMZM2G140355	GRMZM2G140355_P01	bZIP
ZmbZIP109	GRMZM2G407631	GRMZM2G407631_P01	bZIP
ZmbZIP110.1	GRMZM2G025812	GRMZM2G025812_P01	bZIP
ZmbZIP110.2	GRMZM2G025812	GRMZM2G025812_P02	bZIP
ZmbZIP111	GRMZM2G060290	GRMZM2G060290_P01	bZIP
ZmbZIP112.1	GRMZM2G103647	GRMZM2G103647_P03	bZIP
ZmbZIP112.2	GRMZM2G103647	GRMZM2G103647_P01	bZIP
ZmbZIP113	GRMZM2G151295	GRMZM2G151295_P02	bZIP
ZmbZIP114.1	GRMZM2G056099	GRMZM2G056099_P01	bZIP
ZmbZIP114.2	GRMZM2G056099	GRMZM2G056099_P02	bZIP
ZmbZIP115	GRMZM2G066734	GRMZM2G066734_P01	bZIP
ZmbZIP116	GRMZM2G473274	GRMZM2G473274_P01	bZIP
ZmbZIP117	GRMZM2G366264	GRMZM2G366264_P01	bZIP
ZmbZIP118	GRMZM2G365754	GRMZM2G365754_P01	bZIP
ZmbZIP119.1	GRMZM2G136266	GRMZM2G136266_P01	bZIP
ZmbZIP119.2	GRMZM2G136266	GRMZM2G136266_P02	bZIP
ZmbZIP120	GRMZM2G088140	GRMZM2G088140_P02	bZIP
ZmbZIP121	GRMZM2G094352	GRMZM2G094352_P01	bZIP
ZmbZIP122	GRMZM5G884349	GRMZM5G884349_P01	bZIP
ZmbZIP123.1	GRMZM2G425920	GRMZM2G425920_P01	bZIP
ZmbZIP123.2	GRMZM2G425920	GRMZM2G425920_P02	bZIP
ZmbZIP124	GRMZM2G358701	GRMZM2G358701_P01	bZIP
ZmbZIP125.1	GRMZM2G445575	GRMZM2G445575_P01	bZIP
ZmbZIP125.2	GRMZM2G445575	GRMZM2G445575_P02	bZIP
ZmbZIP125.3	GRMZM2G445575	GRMZM2G445575_P03	bZIP
ZmWRKY1	GRMZM2G030272	GRMZM2G030272_P01	WRKY
ZmWRKY2	GRMZM2G425430	GRMZM2G425430_P01	WRKY
ZmWRKY3	GRMZM2G383594	GRMZM2G383594_P01	WRKY
ZmWRKY4	GRMZM2G130374	GRMZM2G130374_P01	WRKY
ZmWRKY5	GRMZM2G324999	GRMZM2G324999_P01	WRKY
ZmWRKY6	GRMZM2G070211	GRMZM2G070211_P01	WRKY
ZmWRKY7	GRMZM2G149219	GRMZM2G149219_P01	WRKY
ZmWRKY8	GRMZM2G018487	GRMZM2G018487_P01	WRKY
ZmWRKY9.1	GRMZM2G143204	GRMZM2G143204_P01	WRKY
ZmWRKY9.2	GRMZM2G143204	GRMZM2G143204_P02	WRKY
ZmWRKY10	GRMZM2G008029	GRMZM2G008029_P01	WRKY
ZmWRKY11	GRMZM2G164082	GRMZM2G164082_P01	WRKY
ZmWRKY12	GRMZM2G083717	GRMZM2G083717_P01	WRKY
ZmWRKY13.1	GRMZM2G071907	GRMZM2G071907_P03	WRKY

ZmWRKY13.2	GRMZM2G071907	GRMZM2G071907_P01	WRKY
ZmWRKY14	GRMZM2G024898	GRMZM2G024898_P01	WRKY
ZmWRKY15.1	GRMZM2G123387	GRMZM2G123387_P01	WRKY
ZmWRKY15.2	GRMZM2G123387	GRMZM2G123387_P02	WRKY
ZmWRKY16	GRMZM5G816457	GRMZM5G816457_P01	WRKY
ZmWRKY17	GRMZM2G102583	GRMZM2G102583_P02	WRKY
ZmWRKY18	GRMZM2G400559	GRMZM2G400559_P01	WRKY
ZmWRKY19	GRMZM2G099593	GRMZM2G099593_P01	WRKY
ZmWRKY20	GRMZM2G163418	GRMZM2G163418_P01	WRKY
ZmWRKY21	GRMZM2G057011	GRMZM2G057011_P01	WRKY
ZmWRKY22	GRMZM2G052671	GRMZM2G052671_P01	WRKY
ZmWRKY23	GRMZM2G130854	GRMZM2G130854_P01	WRKY
ZmWRKY24	GRMZM2G106560	GRMZM2G106560_P01	WRKY
ZmWRKY25.1	GRMZM5G871347	GRMZM5G871347_P01	WRKY
ZmWRKY25.2	GRMZM5G871347	GRMZM5G871347_P02	WRKY
ZmWRKY25.3	GRMZM5G871347	GRMZM5G871347_P03	WRKY
ZmWRKY26	GRMZM2G475984	GRMZM2G475984_P01	WRKY
ZmWRKY27	GRMZM2G176489	GRMZM2G176489_P01	WRKY
ZmWRKY28	GRMZM2G151444	GRMZM2G151444_P01	WRKY
ZmWRKY29	GRMZM2G327349	GRMZM2G327349_P01	WRKY
ZmWRKY30	GRMZM2G173680	GRMZM2G173680_P01	WRKY
ZmWRKY31	GRMZM2G076657	GRMZM2G076657_P01	WRKY
ZmWRKY32	AC165171.2_FG002	AC165171.2_FGP002	WRKY
ZmWRKY33	GRMZM2G148087	GRMZM2G148087_P01	WRKY
ZmWRKY34	GRMZM2G059562	GRMZM2G059562_P01	WRKY
ZmWRKY35	GRMZM2G158328	GRMZM2G158328_P01	WRKY
ZmWRKY36	GRMZM2G065290	GRMZM2G065290_P01	WRKY
ZmWRKY37	GRMZM2G382350	GRMZM2G382350_P01	WRKY
ZmWRKY38.1	GRMZM2G141299	GRMZM2G141299_P02	WRKY
ZmWRKY38.2	GRMZM2G141299	GRMZM2G141299_P01	WRKY
ZmWRKY39	GRMZM2G040298	GRMZM2G040298_P01	WRKY
ZmWRKY40	GRMZM2G101405	GRMZM2G101405_P01	WRKY
ZmWRKY41	GRMZM2G151763	GRMZM2G151763_P01	WRKY
ZmWRKY42	GRMZM2G408462	GRMZM2G408462_P01	WRKY
ZmWRKY43	AC198725.4_FG009	AC198725.4_FGP009	WRKY
ZmWRKY44	GRMZM2G105140	GRMZM2G105140_P01	WRKY
ZmWRKY45	GRMZM2G354384	GRMZM2G354384_P01	WRKY
ZmWRKY46	GRMZM2G054125	GRMZM2G054125_P01	WRKY
ZmWRKY47	GRMZM2G549512	GRMZM2G549512_P01	WRKY
ZmWRKY48	GRMZM2G148561	GRMZM2G148561_P01	WRKY
ZmWRKY49	AC205562.3_FG002	AC205562.3_FGP002	WRKY
ZmWRKY50	GRMZM2G377217	GRMZM2G377217_P01	WRKY
ZmWRKY51	GRMZM2G138683	GRMZM2G138683_P01	WRKY

ZmWRKY52	GRMZM2G063216	GRMZM2G063216_P01	WRKY
ZmWRKY53	GRMZM2G451035	GRMZM2G451035_P01	WRKY
ZmWRKY54	GRMZM2G461648	GRMZM2G461648_P01	WRKY
ZmWRKY55	GRMZM2G169564	GRMZM2G169564_P01	WRKY
ZmWRKY56	GRMZM2G027972	GRMZM2G027972_P01	WRKY
ZmWRKY57	GRMZM2G038158	GRMZM2G038158_P01	WRKY
ZmWRKY58	GRMZM2G147880	GRMZM2G147880_P01	WRKY
ZmWRKY59	GRMZM2G161411	GRMZM2G161411_P01	WRKY
ZmWRKY60	GRMZM5G880069	GRMZM5G880069_P02	WRKY
ZmWRKY61	GRMZM2G120320	GRMZM2G120320_P01	WRKY
ZmWRKY62	GRMZM2G048450	GRMZM2G048450_P01	WRKY
ZmWRKY63	GRMZM2G073272	GRMZM2G073272_P01	WRKY
ZmWRKY64	GRMZM5G823157	GRMZM5G823157_P01	WRKY
ZmWRKY65	GRMZM2G143765	GRMZM2G143765_P01	WRKY
ZmWRKY66	AC209050.3_FG003	AC209050.3_FGP003	WRKY
ZmWRKY67	GRMZM2G156529	GRMZM2G156529_P01	WRKY
ZmWRKY68.1	GRMZM2G163054	GRMZM2G163054_P04	WRKY
ZmWRKY68.2	GRMZM2G163054	GRMZM2G163054_P02	WRKY
ZmWRKY69	GRMZM2G127064	GRMZM2G127064_P01	WRKY
ZmWRKY70.1	GRMZM2G012724	GRMZM2G012724_P01	WRKY
ZmWRKY70.2	GRMZM2G012724	GRMZM2G012724_P03	WRKY
ZmWRKY70.3	GRMZM2G012724	GRMZM2G012724_P04	WRKY
ZmWRKY71	GRMZM2G169966	GRMZM2G169966_P01	WRKY
ZmWRKY72	GRMZM2G401521	GRMZM2G401521_P01	WRKY
ZmWRKY73	GRMZM5G863420	GRMZM5G863420_P01	WRKY
ZmWRKY74	GRMZM2G453571	GRMZM2G453571_P01	WRKY
ZmWRKY75.1	GRMZM2G366795	GRMZM2G366795_P01	WRKY
ZmWRKY75.2	GRMZM2G366795	GRMZM2G366795_P02	WRKY
ZmWRKY76	GRMZM2G018721	GRMZM2G018721_P01	WRKY
ZmWRKY77	GRMZM2G139815	GRMZM2G139815_P01	WRKY
ZmWRKY78	GRMZM2G025895	GRMZM2G025895_P01	WRKY
ZmWRKY79.1	GRMZM2G125653	GRMZM2G125653_P01	WRKY
ZmWRKY79.2	GRMZM2G125653	GRMZM2G125653_P02	WRKY
ZmWRKY80	GRMZM2G169149	GRMZM2G169149_P01	WRKY
ZmWRKY81.1	GRMZM2G151407	GRMZM2G151407_P01	WRKY
ZmWRKY81.2	GRMZM2G151407	GRMZM2G151407_P02	WRKY
ZmWRKY82.1	GRMZM2G398506	GRMZM2G398506_P01	WRKY
ZmWRKY82.2	GRMZM2G398506	GRMZM2G398506_P02	WRKY
ZmWRKY83	GRMZM2G381378	GRMZM2G381378_P01	WRKY
ZmWRKY84	GRMZM2G006497	GRMZM2G006497_P01	WRKY
ZmWRKY85	GRMZM2G516301	GRMZM2G516301_P01	WRKY
ZmWRKY86	GRMZM2G083350	GRMZM2G083350_P01	WRKY
ZmWRKY87	GRMZM2G448605	GRMZM2G448605_P01	WRKY

ZmWRKY88	GRMZM2G063880	GRMZM2G063880_P01	WRKY
ZmWRKY89	GRMZM2G414315	GRMZM2G414315_P01	WRKY
ZmWRKY90	GRMZM2G057116	GRMZM2G057116_P01	WRKY
ZmWRKY91	GRMZM2G061408	GRMZM2G061408_P01	WRKY
ZmWRKY92.1	GRMZM2G449681	GRMZM2G449681_P01	WRKY
ZmWRKY92.2	GRMZM2G449681	GRMZM2G449681_P02	WRKY
ZmWRKY93	GRMZM2G034421	GRMZM2G034421_P01	WRKY
ZmWRKY94	GRMZM2G432583	GRMZM2G432583_P01	WRKY
ZmWRKY95	GRMZM2G015433	GRMZM2G015433_P01	WRKY
ZmWRKY96	GRMZM2G137802	GRMZM2G137802_P01	WRKY
ZmWRKY97	GRMZM2G304573	GRMZM2G304573_P01	WRKY
ZmWRKY98	GRMZM2G149683	GRMZM2G149683_P01	WRKY
ZmWRKY99	GRMZM2G145554	GRMZM2G145554_P01	WRKY
ZmWRKY100	GRMZM2G411766	GRMZM2G411766_P01	WRKY
ZmWRKY101	GRMZM5G812272	GRMZM5G812272_P02	WRKY
ZmWRKY102	GRMZM2G045560	GRMZM2G045560_P01	WRKY
ZmWRKY103	GRMZM2G036703	GRMZM2G036703_P01	WRKY
ZmWRKY104	GRMZM2G029282	GRMZM2G029282_P01	WRKY
ZmWRKY105	GRMZM2G029292	GRMZM2G029292_P03	WRKY
ZmWRKY106	GRMZM2G013391	GRMZM2G013391_P01	WRKY
ZmWRKY107	GRMZM2G111354	GRMZM2G111354_P01	WRKY
ZmWRKY108	GRMZM2G003551	GRMZM2G003551_P01	WRKY
ZmWRKY109	GRMZM2G111711	GRMZM2G111711_P01	WRKY
ZmWRKY110.1	GRMZM2G171428	GRMZM2G171428_P01	WRKY
ZmWRKY110.2	GRMZM2G171428	GRMZM2G171428_P02	WRKY
ZmWRKY111	GRMZM2G060918	GRMZM2G060918_P01	WRKY
ZmWRKY112	GRMZM2G005207	GRMZM2G005207_P01	WRKY
ZmWRKY113	GRMZM2G441031	GRMZM2G441031_P01	WRKY
ZmWRKY114	GRMZM2G090594	GRMZM2G090594_P01	WRKY
ZmWRKY115	GRMZM2G004060	GRMZM2G004060_P01	WRKY
ZmWRKY116.1	GRMZM2G020254	GRMZM2G020254_P01	WRKY
ZmWRKY116.2	GRMZM2G020254	GRMZM2G020254_P02	WRKY
ZmWRKY116.3	GRMZM2G020254	GRMZM2G020254_P03	WRKY
ZmWRKY117	GRMZM2G031963	GRMZM2G031963_P01	WRKY
ZmWRKY118	AC208110.2_FG001	AC208110.2_FGP001	WRKY
ZmSIMK1	GRMZM2G127141	GRMZM2G127141_P01	MAPK
ZmMPK2	GRMZM2G123886	GRMZM2G123886_P01	MAPK
ZmMPK3	GRMZM2G017792	GRMZM2G017792_P01	MAPK
ZmMPK4	GRMZM2G053987	GRMZM2G053987_P02	MAPK
ZmMPK5	GRMZM2G020216	GRMZM2G020216_P01	MAPK
ZmMPK6	GRMZM2G089484	GRMZM2G089484_P01	MAPK
ZmMPK7	GRMZM2G002100	GRMZM2G002100_P01	MAPK
ZmMPK8	GRMZM2G048455	GRMZM2G048455_P01	MAPK

ZmMPK9	GRMZM2G062914	GRMZM2G062914_P01	MAPK
ZmMPK10	GRMZM2G034052	GRMZM2G034052_P01	MAPK
ZmMPK11	GRMZM2G163861	GRMZM2G163861_P01	MAPK
ZmMPK12	GRMZM2G131334	GRMZM2G131334_P02	MAPK
ZmMPK13	GRMZM2G122335	GRMZM2G122335_P01	MAPK
ZmMPK14	GRMZM2G007848	GRMZM2G007848_P01	MAPK
ZmMPK15	GRMZM2G135904	GRMZM2G135904_P02	MAPK
ZmMPK17	GRMZM2G374088	GRMZM2G374088_P01	MAPK
ZmMPK16	GRMZM2G306028	GRMZM2G306028_P02	MAPK
ZmMPK18	GRMZM2G375975	GRMZM2G375975_P01	MAPK
ZmMPK19	GRMZM2G062761	GRMZM2G062761_P01	MAPK
ZmRACR1	GRMZM2G063882	GRMZM2G063882_P01	ABA receptor
ZmRACR2	GRMZM2G048733	GRMZM2G048733_P02	ABA receptor
ZmRACR3	GRMZM2G405064	GRMZM2G405064_P01	ABA receptor
ZmRACR4	GRMZM2G165567	GRMZM2G165567_P02	ABA receptor
ZmRACR5	GRMZM2G133631	GRMZM2G133631_P01	ABA receptor
ZmRACR6	GRMZM2G144224	GRMZM2G144224_P01	ABA receptor
ZmRACR7	GRMZM2G057959	GRMZM2G057959_P01	ABA receptor
ZmRACR8	GRMZM2G047677	GRMZM2G047677_P01	ABA receptor
ZmRACR9	GRMZM2G169695	GRMZM2G169695_P01	ABA receptor
ZmRACR10	GRMZM2G141382	GRMZM2G141382_P01	ABA receptor
ZmRACR11	GRMZM2G134731	GRMZM2G134731_P01	ABA receptor
ZmRACR12	AC194914.3_FG002	AC194914.3_FGP002	ABA receptor
ZmRACR13	GRMZM2G154987	GRMZM2G154987_P01	ABA receptor
ZmPP6	GRMZM2G010855	GRMZM2G010855_P01	PP2CA
ZmPP31	GRMZM2G019819	GRMZM2G019819_P01	PP2CA
ZmPP39	GRMZM2G082487	GRMZM2G082487_P01	PP2CA
ZmPP53	GRMZM2G059453	GRMZM2G059453_P01	PP2CA
ZmPP56	GRMZM2G122228	GRMZM2G122228_P01	PP2CA
ZmPP57	GRMZM2G134628	GRMZM2G134628_P01	PP2CA
ZmPP107	GRMZM2G177386	GRMZM2G177386_P02	PP2CA
ZmPP108	GRMZM2G102255	GRMZM2G102255_P01	PP2CA
ZmPP112	GRMZM2G308615	GRMZM2G308615_P01	PP2CA
ZmPP121	GRMZM5G818101	GRMZM5G818101_P02	PP2CA
ZmPP123	GRMZM2G383807	GRMZM2G383807_P01	PP2CA
ZmPP127	GRMZM2G001243	GRMZM2G001243_P01	PP2CA
ZmPP130	GRMZM2G300125	GRMZM2G300125_P01	PP2CA
ZmPP132	GRMZM2G166297	GRMZM2G166297_P01	PP2CA
ZmPP134	GRMZM2G149132	GRMZM2G149132_P01	PP2CA
ZmCAX1	GRMZM5G809587	GRMZM5G809587_P03	Ca^{2+}/H^{+}antiporter
ZmNHX1	GRMZM2G027851	GRMZM2G027851_P01	Na^{+}/H^{+}antiporters
ZmNHX3	GRMZM2G063492	GRMZM2G063492_P01	Na^{+}/H^{+}antiporters
ZmNHX4	GRMZM2G037342	GRMZM2G037342_P02	Na^{+}/H^{+}antiporters
