# Supplementary material for: Maize protein phosphatase gene family: identification and molecular characterization
Source: BMC Genomics. 2014 Sep 9;15(1):773. doi: 10.1186/1471-2164-15-773 (PMC4169795; doi:10.1186/1471-2164-15-773)
Supplement: Supplementary file 17 — Additional file 17: Table S7: List of expression values of MAPK cascade-related genes and key genes of ABA biosynthesis in maize under salt stress. (PDF 74 KB) [file 12864_2014_6458_MOESM17_ESM.pdf]

Table S7. List of expression values of MAPK cascade-related genes and key genes of ABA biosynthesis in maize under salt stress.

Values in red and blue indicate the fold increase and decrease in expression in the salt-stressed tissue, respectively.

Name	CR	Name	PR	Nane	SR
ZmMPK3	0.822793	*ZmMPK10*	0.647817	*ZmMPK3*	0.708986
ZmMPK12	0.357354	*ZmMPK19*	0.561517	*ZmMPK12*	0.470683
ZmMPK6	0.194932	*ZmMPK12*	0.429276	*ZmMPK19*	0.457447
ZmMPK10	0.1521	*ZmMPK14*	0.168414	*ZmMPK10*	0.235054
ZmMPK19	0.045185	*ZmMPK6*	0.121254	*ZmMPK14*	0.206726
ZmMPK5	0.019545	*ZmMPK5*	0.063902	*ZmMPK5*	0.140035
ZmMPK14	-0.646976	*ZmMPK3*	-0.12557	*ZmMPK6*	0.116234
ZmMPK15	-0.043352	*ZmMPK15*	-0.00074	*ZmMPK15*	0.058608
ZmMKK3	0.295163	*ZmMKK6*	0.073743	*ZmMKK3*	0.309055
ZmMKK2	0.23363	*ZmMKK3*	0.072793	*ZmMEK1*	0.203611
ZmMEK1	0.061768	*ZmMKK2*	-0.49017	*ZmMKK6*	-0.35159
ZmMKK4	-0.100245	*ZmMKK4*	-0.22838	*ZmMKK4*	-0.29101
ZmMKK6	-0.068771	*ZmMEK1*	-0.06204	*ZmMKK2*	-0.23234
ZmRaf46	1.723257	*ZmRaf7*	0.665914	*ZmRaf46*	0.708986
ZmRaf49	0.522887	*ZmRaf9*	0.61962	*ZmRaf38*	0.688227
ZmRaf17	0.489666	*ZmMAPKKK23*	0.563037	*ZmRaf22*	0.615876
ZmZIK1	0.481284	*ZmRaf17*	0.45472	*ZmMAPKKK20*	0.509677
ZmRaf13	0.412917	*ZmMAPKKK15*	0.356586	*ZmRaf14*	0.468325
ZmMAPKKK24	0.357299	*ZmRaf44*	0.351311	*ZmRaf17*	0.364337
ZmRaf29	0.251636	*ZmRaf26*	0.304118	*ZmRaf49*	0.359836
ZmRaf42	0.225757	*ZmZIK1*	0.289472	*ZmRaf2*	0.317156
ZmRaf2	0.222063	*ZmRaf21*	0.256853	*ZmZIK9*	0.309055
ZmRaf6	0.21511	*ZmRaf38*	0.2022	*ZmRaf45*	0.270865
ZmRaf38	0.206936	*ZmRaf45*	0.196267	*ZmRaf34*	0.270848
ZmZIK8	0.156216	*ZmRaf4*	0.184819	*ZmRaf48*	0.250996
ZmRaf40	0.149522	*ZmMAPKKK24*	0.184298	*ZmRaf20*	0.235054
ZmRaf45	0.147065	*ZmRaf39*	0.180887	*ZmRaf40*	0.204681
ZmZIK9	0.144721	*ZmZIK4*	0.180555	*ZmMAPKKK24*	0.187526
ZmRaf39	0.144457	*ZmMAPKKK22*	0.16116	*ZmRaf6*	0.182238
ZmMAPKKK22	0.13976	*ZmRaf47*	0.149353	*ZmZIK8*	0.139748
ZmRaf4	0.089579	*ZmZIK8*	0.138994	*ZmMAPKKK6*	0.13803
ZmMAPKKK18	0.076894	*ZmRaf6*	0.137945	*ZmZIK2*	0.117451
ZmRaf21	0.045185	*ZmZIK2*	0.135086	*ZmRaf21*	0.10381
ZmMAPKKK8	-1.443101	*ZmRaf40*	0.128827	*ZmRaf13*	0.057993
ZmMAPKKK17	-0.902347	*ZmRaf13*	0.117398	*ZmMAPKKK22*	0.056682
ZmMAPKKK6	-0.774243	*ZmRaf33*	0.116382	*ZmMAPKKK18*	0.04417

ZmRaf9	-0.420478	*ZmRaf19*	0.113501	*ZmZIK5*	0.029736
ZmRaf44	-0.417989	*ZmRaf29*	0.111473	*ZmMAPKKK17*	0.028604
ZmRaf22	-0.350744	*ZmRaf20*	0.10382	*ZmRaf27*	0.028604
ZmMAPKKK13	-0.333327	*ZmZIK9*	0.09909	*ZmRaf39*	0.023301
ZmRaf18	-0.324572	*ZmRaf31*	0.081921	*ZmRaf30*	0.020168
ZmRaf30	-0.269089	*ZmRaf1*	0.03149	*ZmRaf44*	0.009014
ZmRaf1	-0.266017	*ZmRaf14*	0.028571	*ZmRaf33*	0.003729
ZmMAPKKK20	-0.234923	*ZmRaf48*	0.027705	*ZmMAPKKK13*	-
ZmRaf7	-0.232349	*ZmMAPKKK6*	-	*ZmMAPKKK8*	-0.53048
ZmZIK2	-0.222437	*ZmMAPKKK8*	-	*ZmMAPKKK23*	-0.44302
ZmRaf48	-0.15198	*ZmMAPKKK13*	-	*ZmZIK4*	-0.41591
ZmMAPKKK16	-0.150539	*ZmRaf46*	-	*ZmRaf4*	-0.39835
ZmZIK6	-0.14746	*ZmRaf22*	-0.93292	*ZmRaf42*	-0.37688
ZmZIK4	-0.145863	*ZmZIK6*	-0.54388	*ZmMAPKKK26*	-0.32475
ZmRaf14	-0.110733	*ZmMAPKKK17*	-0.47349	*ZmRaf47*	-0.27901
ZmMAPKKK15	-0.110093	*ZmRaf36*	-0.43696	*ZmRaf18*	-0.27591
ZmRaf31	-0.107585	*ZmRaf18*	-0.42769	*ZmRaf1*	-0.2752
ZmRaf20	-0.106818	*ZmMAPKKK20*	-0.38038	*ZmRaf7*	-0.26502
ZmRaf47	-0.099313	*ZmRaf30*	-0.20554	*ZmMAPKKK16*	-0.2111
ZmZIK5	-0.093262	*ZmMAPKKK18*	-0.20388	*ZmRaf26*	-0.1979
ZmRaf19	-0.086059	*ZmRaf27*	-0.17056	*ZmRaf9*	-0.16548
ZmMAPKKK26	-0.053923	*ZmRaf49*	-0.14272	*ZmRaf19*	-0.1349
ZmRaf34	-0.049244	*ZmMAPKKK16*	-0.13752	*ZmRaf29*	-0.12752
ZmRaf33	-0.027419	*ZmMAPKKK26*	-0.08037	*ZmMAPKKK15*	-0.11544
ZmRaf26	-0.025204	*ZmRaf2*	-0.06872	*ZmZIK1*	-0.02798
ZmMAPKKK23	-0.021929	*ZmRaf42*	-0.05845	*ZmZIK6*	-0.02798
ZmRaf36	-0.016215	*ZmRaf34*	-0.03906	*ZmRaf36*	-0.02798
ZmRaf27	-0.008586	*ZmZIK5*	-0.00084	*ZmRaf31*	-0.02798
ZmMAP4K5	0.765077	*ZmMAP4K2*	0.349206	*ZmMAP4K6*	0.637601
ZmMAP4K2	0.091728	*ZmMAP4K1*	0.063227	*ZmMAP4K2*	0.486593
ZmMIK	-0.112356	*ZmMAP4K5*	-0.13862	*ZmMAP4K1*	0.198905
ZmMAP4K3	-0.026965	*ZmMIK*	-0.12884	*ZmMIK*	0.0739
ZmMAP4K1	-0.009263	*ZmMAP4K3*	-0.02808	*ZmMAP4K3*	0.035214
ZmMAP4K6	-0.007282	*ZmMAP4K6*	-0.02283	*ZmMAP4K5*	-0.66025
VP14	2.17522	VP14	-	VP14	3.252038
ZmAO2	0.827522	ZmAO2	0.398593	ZmAO2	1.070303
ZmAO1	0.158123	ZmAO1	0.2668	ZmAO1	0.285491
