# Supplementary material for: Maize protein phosphatase gene family: identification and molecular characterization
Source: BMC Genomics. 2014 Sep 9;15(1):773. doi: 10.1186/1471-2164-15-773 (PMC4169795; doi:10.1186/1471-2164-15-773)
Supplement: Supplementary file 18 — Additional file 18: Table S8: List of FPKM values of ZmPPs in maize reproductive (cob) and vegetative tissue (leaf) under both drought and well-watered conditions. MCC and MCD stand for maize ovary, well watered and drought, respectively; MLC and MLD stand for maize basal leaf meristem, well watered and drought, respectively. Numbers 1 and 2 indicate the two biological replicates. The extent of differential expression is measured in terms of fold change and (-) indicates failure to calculate or undetected values. Values in red and blue indicate the fold increase and decrease in expression in the drought-stressed tissue, respectively. (PDF 220 KB) [file 12864_2014_6458_MOESM18_ESM.pdf]

Table S8. List of FPKM values of *ZmPPs* in maize reproductive (cob) and vegetative tissue (leaf) under both drought and well-watered conditions.

MCC and MCD stand for maize ovary, well watered and drought, respectively; MLC and MLD stand for maize basal leaf meristem, well watered and drought, respectively.

Numbers 1 and 2 indicate the two biological replicates.

The extent of differential expression is measured in terms of fold change and (-) indicates failure to calculate or undetected values.

Values in red and blue indicate the fold increase and decrease in expression in the drought-stressed tissue, respectively.

**Name**	**MLC-1**	**MLC-2**	**MLD-1**	**MLD-2**	**leaf**	**Name**	**MCC-1**	**MCC-2**	**MCD-1**	**MCD-2**	**cob**
ZmPP130	0.185674	0.066071	1.33133	1.90762	12.866	ZmPP56	1.04451	1.28581	24.9423	31.9753	24.4248
ZmPP154	1.59809	0.376698	5.761	9.98914	7.97561	ZmPP67	5.21057	5.2209	43.7203	71.5037	11.0458
ZmPP113	0.484378	0.196986	1.38925	3.95152	7.83835	ZmPP107	38.1802	36.5708	339.674	361.27	9.37705
ZmPP146	2.24921	0.515408	10.2968	10.6892	7.59092	ZmPP112	3.64398	4.16687	31.5676	37.0921	8.7903
ZmPP31	0.20963	0.111677	0.562482	1.0729	5.08978	ZmPP39	5.27084	7.3829	45.4388	54.5162	7.89925
ZmPP124	3.66464	3.72866	18.0225	18.5982	4.95323	ZmPP154	3.20284	5.04657	28.3206	36.5484	7.86347
ZmPP29	12.0032	7.26424	35.0496	32.4684	3.50425	ZmPP130	1.46837	1.72438	11.0961	12.7946	7.4828
ZmPP21	7.75276	10.1622	25.9208	24.897	2.83661	ZmPP6	30.6439	19.3653	166.999	156.819	6.47517
ZmPP6	13.2422	5.96945	21.988	27.9786	2.60085	ZmPP114	9.05156	6.6742	33.1996	32.6189	4.18539
ZmPP127	12.2224	11.8801	27.1083	24.6031	2.14548	ZmPP158	20.75	18.8583	72.6705	78.1879	3.80876
ZmPP90	5.35336	6.43224	11.443	11.6782	1.96182	ZmPP127	48.0534	56.6635	188.154	205.834	3.76241
ZmPP57	18.5235	14.0137	28.2524	33.7509	1.90561	ZmPP57	58.579	60.7115	213.385	220.639	3.63838
ZmPP67	4.97823	3.87003	8.73824	7.97337	1.88869	ZmPP82	9.19464	5.35317	22.591	29.4821	3.57945
ZmPP149	2.63842	3.26649	5.97417	4.75183	1.81645	ZmPP53	11.0058	13.8623	39.2616	44.3019	3.36027
ZmPP74	11.3371	18.6391	30.1931	24.2148	1.81504	ZmPP132	26.0031	34.0162	84.9169	98.3847	3.05404
ZmPP9	1.72638	1.41624	2.81897	2.45387	1.67785	ZmPP8	11.1297	5.09641	24.3144	23.8146	2.96615
ZmPP119	2.00781	1.28805	2.83876	2.52035	1.62601	ZmPP89	50.3389	44.629	119.561	152.385	2.86356
ZmPP129	1.60432	0.887178	1.72336	2.29755	1.61385	ZmPP27	10.5591	11.0851	28.757	32.5497	2.83248
ZmPP53	19.9122	12.6392	26.7656	25.6849	1.61131	ZmPP152	9.9546	6.04288	23.1463	22.1456	2.83119
ZmPP80	6.73822	6.83238	10.5571	10.8681	1.5788	ZmPP76	91.2057	77.4417	225.877	242.135	2.77509
ZmPP147	12.4116	16.1198	23.7318	21.1765	1.574	ZmPP102	22.5061	17.6575	70.2547	37.3971	2.68033
ZmPP66	4.98943	3.14712	6.31716	6.33883	1.55545	ZmPP147	4.03735	2.46956	9.05094	8.0589	2.62949
ZmPP107	21.688	22.6746	34.1335	34.0586	1.53715	ZmPP84	133.799	129.48	350.681	314.375	2.52605
ZmPP156	13.9405	8.38363	16.3703	16.983	1.49405	ZmPP77	2.54611	2.86422	6.16176	6.7516	2.3868
ZmPP151	29.9995	30.2764	45.3952	43.1828	1.46954	ZmPP86	36.7961	26.6074	74.953	76.3566	2.38646
ZmPP118	12.4979	10.1137	17.059	16.0399	1.4638	ZmPP70	24.9858	22.9534	51.6358	59.722	2.3229
ZmPP132	30.9497	19.4919	37.8076	32.7186	1.39818	ZmPP66	2.32257	2.77945	4.39515	6.9931	2.23211
ZmPP59	16.8321	14.6969	15.7944	26.2278	1.33281	ZmPP16	2.7846	2.6143	5.70469	6.11631	2.18952
ZmPP3	34.6636	29.4353	42.0113	42.4058	1.31698	ZmPP11	21.0873	19.269	39.5077	41.0968	1.99732
ZmPP85	148.536	151.026	195.212	194.284	1.30022	ZmPP50	18.648	15.3533	32.0618	35.4659	1.98603
ZmPP49	7.81392	5.96188	8.66562	8.95123	1.27883	ZmPP12	21.3986	24.09	44.2435	44.1075	1.94227
ZmPP121	5.34135	5.80499	6.35177	7.88247	1.27703	ZmPP119	9.55329	7.13517	15.3407	16.8754	1.93044
ZmPP103	24.191	28.1741	33.8413	32.7325	1.27134	ZmPP74	8.29224	10.5032	16.1432	19.4852	1.89559

ZmPP116	9.69173	11.7587	13.0926	13.9851	1.26234	ZmPP159	23.1697	21.8698	39.8886	42.1443	1.82135
ZmPP122	13.7251	10.217	14.9458	15.2366	1.26064	ZmPP103	17.6715	15.288	26.4096	31.4388	1.75514
ZmPP158	27.3912	29.5786	37.5611	33.5318	1.24791	ZmPP121	9.10774	11.0797	18.0611	15.5674	1.66581
ZmPP39	7.28209	3.93612	6.8885	7.07429	1.24465	ZmPP117	7.9963	8.77288	13.3379	14.257	1.64557
ZmPP8	20.4467	39.3878	34.0703	39.7544	1.23381	ZmPP125	13.2454	13.1151	20.3106	22.9016	1.63928
ZmPP143	16.6265	14.9732	18.7862	19.6428	1.21612	ZmPP85	33.648	22.3069	50.4755	40.3082	1.62244
ZmPP141	4.25445	3.4396	4.80061	4.39825	1.19558	ZmPP58	18.4888	15.2274	26.7394	27.8146	1.61804
ZmPP76	70.5727	69.0452	88.4138	77.148	1.18582	ZmPP5	152.889	162.416	251.413	253.521	1.60141
ZmPP12	10.3607	12.8873	13.9513	13.3005	1.17222	ZmPP30	18.2591	14.2942	26.1071	23.2174	1.51519
ZmPP27	11.3944	10.7615	13.0402	12.9301	1.17216	ZmPP36	32.1811	26.3726	45.0716	42.6853	1.49874
ZmPP123	4.61382	5.01435	5.42137	5.85181	1.17085	ZmPP18	33.9997	27.1508	46.9525	44.221	1.49097
ZmPP120	0.849872	0.678156	0.960236	0.828849	1.17085	ZmPP153	32.3494	41.6334	45.9235	63.9916	1.48568
ZmPP2	16.2443	15.017	18.3718	17.8721	1.15939	ZmPP1	47.704	28.7508	54.255	58.6306	1.4765
ZmPP54	50.1249	37.8985	47.5748	52.7962	1.14028	ZmPP131	16.2074	13.7505	20.8027	23.3582	1.4741
ZmPP11	17.2698	19.2814	15.7121	16.647	1.12955	ZmPP52	16.4845	13.6432	21.9472	19.8485	1.38728
ZmPP33	7.07584	7.44706	8.98425	7.08869	1.10673	ZmPP55	57.8138	37.7231	67.4245	61.6255	1.35079
ZmPP46	86.2351	80.2141	90.1065	93.5276	1.10324	ZmPP51	24.774	20.3823	32.3474	28.6036	1.34978
ZmPP86	20.7928	20.0617	22.042	22.2939	1.08521	ZmPP26	131.027	107.928	161.833	160.163	1.34752
ZmPP153	25.1572	23.6001	27.0062	25.6218	1.07939	ZmPP32	12.1652	13.687	18.8258	15.7912	1.33903
ZmPP28	8.18833	8.90074	9.48358	8.7541	1.06721	ZmPP139	19.1456	15.9555	22.8918	21.8025	1.2733
ZmPP93	70.6074	27.1429	54.3915	49.8267	1.06617	ZmPP105	9.85205	10.0315	10.8037	14.4375	1.26945
ZmPP38	21.0444	19.3032	20.3132	22.4182	1.05908	ZmPP149	1.03684	1.02955	1.32947	1.25263	1.24957
ZmPP42	14.864	15.9897	16.9183	15.4272	1.04835	ZmPP78	17.8435	16.8402	20.6351	21.9734	1.22849
ZmPP138	22.5884	19.7551	22.4607	20.1412	1.0061	ZmPP14	14.9371	13.3572	16.7259	16.7702	1.18385
ZmPP106	15.7094	16.3935	15.9277	16.284	1.00339	ZmPP138	33.0021	25.4637	36.8276	32.2419	1.18137
ZmPP40	0.331158	0.117346	0.073778	0	6.0791	ZmPP144	15.1872	13.5984	16.1179	17.7065	1.17505
ZmPP133	116.6	229.209	54.2873	50.5259	3.29929	ZmPP24	2.36699	1.78147	2.32481	2.52774	1.16972
ZmPP68	1.11747	0.397004	0.389779	0.294518	2.21318	ZmPP128	14.5558	13.6606	15.2649	17.4691	1.16011
ZmPP125	13.4543	13.0776	8.20332	7.28758	1.71274	ZmPP100	23.7016	23.6762	27.2509	26.4344	1.13313
ZmPP77	6.45944	5.1612	3.64185	3.23558	1.68968	ZmPP140	68.2834	64.9481	70.1462	79.5097	1.12328
ZmPP105	55.6624	94.3706	49.7978	45.3481	1.57687	ZmPP81	29.4897	27.2894	32.1501	31.5501	1.1219
ZmPP7	0.461365	0.462036	0.318559	0.279202	1.54477	ZmPP62	47.1975	51.166	56.48	53.7435	1.12057
ZmPP95	16.4874	9.02565	9.22345	7.32581	1.54164	ZmPP19	29.9932	26.1723	29.0573	33.827	1.11963
ZmPP24	6.96567	8.63377	5.33443	4.93724	1.51869	ZmPP22	45.0931	34.4947	42.2148	46.5219	1.11495
ZmPP101	19.1545	15.0024	11.5594	11.5565	1.47764	ZmPP73	12.0116	10.2779	11.7121	13.0759	1.11209
ZmPP96	73.7034	71.8415	53.0655	47.0989	1.45306	ZmPP44	10.0116	7.85127	10.2215	9.6331	1.1115
ZmPP137	8.36853	7.29775	5.40696	5.41981	1.44699	ZmPP104	47.1538	28.9169	45.6717	38.4602	1.10597
ZmPP55	57.3109	49.1493	41.1232	34.6892	1.40426	ZmPP33	5.38771	5.76108	6.63012	5.46192	1.08461
ZmPP159	37.4392	39.969	29.3275	26.8906	1.37693	ZmPP83	31.5839	34.7421	38.2951	32.3841	1.06563
ZmPP87	55.9537	53.485	43.5427	37.9824	1.34239	ZmPP80	9.96954	9.04872	10.0567	9.87198	1.04787
ZmPP117	7.22686	12.8119	8.27118	6.66828	1.34133	ZmPP42	15.3564	17.8587	16.7892	17.9745	1.04662
ZmPP35	5.25554	3.07415	2.72464	3.48644	1.3411	ZmPP21	9.3037	9.46444	8.91574	10.6738	1.04377
ZmPP134	14.217	12.0266	10.1268	9.54927	1.33378	ZmPP90	12.479	10.2509	11.5734	12.0774	1.04051
ZmPP50	19.6598	22.3562	16.3207	16.0971	1.29608	ZmPP64	8.2555	6.00063	7.15862	7.63307	1.03757

ZmPP79	30.4668	31.5017	24.3475	23.5499	1.29378	ZmPP108	14.8591	16.988	15.6455	16.774	1.01797
ZmPP145	163.038	166.309	130.26	130.151	1.26472	ZmPP23	29.7905	27.8377	29.0629	29.6003	1.01796
ZmPP10	7.45523	8.42871	6.76743	5.82466	1.26142	ZmPP141	16.1016	13.7913	14.914	15.0749	1.00321
ZmPP73	11.4903	8.45641	8.2788	7.60057	1.25614	ZmPP99	48.7079	55.2251	55.8019	48.4354	1.00293
ZmPP44	6.60234	6.27345	5.06418	5.31486	1.24056	ZmPP146	30.2943	26.0334	3.04059	2.54314	10.0878
ZmPP19	16.3468	20.3377	14.6321	15.0629	1.23538	ZmPP59	13.8876	17.5477	2.9221	2.33276	5.98214
ZmPP5	70.5041	83.5952	63.4697	61.4093	1.23399	ZmPP35	5.42582	4.37968	0.898318	1.0287	5.08843
ZmPP81	23.1525	27.052	21.5792	19.267	1.22911	ZmPP28	10.6021	12.4982	3.38174	3.89878	3.17289
ZmPP62	29.0449	36.8254	28.786	26.0936	1.20027	ZmPP87	10.4185	15.5331	3.83474	5.73153	2.71282
ZmPP65	90.8401	72.7938	68.9515	67.4221	1.19989	ZmPP29	38.373	31.4447	14.6423	12.1796	2.60301
ZmPP139	13.2522	12.3145	10.3855	11.0228	1.19424	ZmPP133	1.74203	0.850706	0.329314	0.71266	2.48829
ZmPP152	22.3027	24.3494	20.5095	18.559	1.19411	ZmPP156	9.94471	6.3643	3.61743	3.17095	2.40249
ZmPP155	100.941	114.559	90.2031	91.0936	1.18866	ZmPP110	12.4356	11.2484	5.04746	5.00348	2.3564
ZmPP144	21.9672	23.7418	20.7523	17.7033	1.18862	ZmPP145	55.6328	69.2476	24.6477	31.2308	2.23486
ZmPP140	82.3085	89.68	71.7232	73.3405	1.18561	ZmPP46	6.68555	8.99703	5.03838	2.12323	2.18981
ZmPP98	27.1719	23.8227	21.0347	22.0664	1.18314	ZmPP120	6.39303	5.02293	3.07505	2.20925	2.16035
ZmPP71	17.7559	13.8277	12.381	14.3298	1.18243	ZmPP9	7.35266	5.38827	3.03914	2.92015	2.13799
ZmPP18	15.7235	17.0493	13.7021	14.0364	1.18149	ZmPP68	3.62532	4.41198	2.05066	1.72709	2.12754
ZmPP70	42.0596	44.3133	38.5656	34.5762	1.1809	ZmPP72	38.5462	32.3006	17.3409	19.0816	1.94514
ZmPP83	36.3859	31.317	29.5255	28.0858	1.17517	ZmPP95	5.44812	4.45256	2.45946	3.18048	1.75546
ZmPP34	13.6408	12.8962	11.1403	11.4769	1.17331	ZmPP37	9.65837	7.64606	5.58963	4.52721	1.71046
ZmPP115	58.4366	38.2366	45.1109	37.283	1.17331	ZmPP118	24.2233	24.4197	13.4486	15.4172	1.68514
ZmPP52	17.2767	13.5477	14.6748	11.736	1.16711	ZmPP47	168.488	183.085	100.35	108.859	1.68049
ZmPP58	86.972	119.642	83.8663	93.2094	1.16681	ZmPP94	1.14893	0.923301	0.376262	0.901773	1.62142
ZmPP78	22.2092	20.7969	17.5028	19.3801	1.16602	ZmPP143	7.00821	8.08854	5.09695	4.62049	1.55357
ZmPP112	2.86505	0.9147	1.52314	1.73677	1.15946	ZmPP31	1.01396	2.30448	1.10731	1.05203	1.53678
ZmPP110	9.41879	8.66314	7.92931	7.69231	1.15749	ZmPP54	22.6887	23.9481	15.3277	15.3092	1.52224
ZmPP104	25.385	24.8747	21.9043	21.6233	1.15466	ZmPP10	5.88827	6.7994	4.08401	4.38576	1.49799
ZmPP1	73.4701	96.4074	73.7093	75.5407	1.13821	ZmPP48	26.086	19.74	16.4954	14.3452	1.4859
ZmPP108	9.12133	13.4001	9.70928	10.2021	1.13108	ZmPP122	31.2016	26.8275	21.0068	18.8682	1.45528
ZmPP69	2.22415	2.01515	1.87882	1.88163	1.12734	ZmPP109	14.648	12.0413	9.39185	9.0439	1.44769
ZmPP135	7.56716	6.81701	6.36205	6.45553	1.12222	ZmPP75	28.0376	30.122	19.8514	20.4468	1.44323
ZmPP100	23.3465	21.7561	21.0255	19.3686	1.11656	ZmPP101	20.7666	25.5066	16.1951	16.0416	1.43542
ZmPP75	23.0639	23.5941	19.4665	22.3424	1.11598	ZmPP135	16.268	16.2142	11.8223	11.311	1.40413
ZmPP45	23.9198	21.4927	19.9735	20.75	1.11514	ZmPP96	63.1203	46.9631	36.4465	42.1163	1.40122
ZmPP109	6.73948	7.62699	6.39697	6.55203	1.10947	ZmPP124	8.53421	6.4324	6.05305	4.99118	1.35515
ZmPP99	75.6677	56.4876	62.7545	56.3758	1.10933	ZmPP116	21.7695	22.5203	13.5557	19.2703	1.34923
ZmPP32	9.95496	9.27084	9.28434	8.06862	1.10793	ZmPP13	6.61811	7.79393	5.94663	5.06791	1.30846
ZmPP37	6.62915	7.10718	6.22466	6.25631	1.10058	ZmPP49	15.7871	13.8159	11.2344	11.8965	1.2798
ZmPP23	26.6848	24.7501	22.7013	24.1619	1.09755	ZmPP92	3.72201	4.85476	1.23605	1.3116	1.27383
ZmPP114	12.6079	14.6025	12.5447	12.3065	1.09493	ZmPP34	14.7981	13.5688	10.9697	11.592	1.2573
ZmPP136	38.6948	42.125	37.3907	36.9024	1.08785	ZmPP79	67.725	58.7948	48.5442	52.8332	1.24801
ZmPP14	30.7162	34.3841	28.664	32.2899	1.06803	ZmPP137	17.7565	17.5794	14.3021	14.4142	1.23052
ZmPP88	30.0892	29.6455	28.3706	27.6331	1.06662	ZmPP45	34.5749	29.6712	25.3792	27.241	1.22094

ZmPP64	2.64749	3.07377	2.64029	2.73406	1.06455	ZmPP123	6.20928	7.59663	4.85596	6.59948	1.20518
ZmPP131	20.8915	17.136	19.2322	16.5843	1.06173	ZmPP25	9.58167	10.8395	8.72324	8.39267	1.19311
ZmPP41	7.26561	7.52162	7.7893	6.18906	1.05787	ZmPP155	32.1221	26.1056	24.4357	24.6018	1.18741
ZmPP25	9.24159	8.83369	8.68741	8.4241	1.05632	ZmPP41	13.528	18.0055	10.5297	16.5774	1.16329
ZmPP89	63.2377	54.4462	63.9442	48.0936	1.05039	ZmPP38	8.25554	10.921	8.05281	8.46345	1.16107
ZmPP48	4.81959	3.54623	3.68704	4.27749	1.05038	ZmPP115	42.4696	43.9734	39.9785	35.8384	1.14015
ZmPP36	27.4638	29.6612	27.3134	27.2252	1.04742	ZmPP151	42.9379	45.735	38.969	39.0487	1.13657
ZmPP128	12.4945	10.1266	10.6101	10.99	1.04727	ZmPP3	27.2061	26.6465	23.8611	25.3199	1.09499
ZmPP97	0.590819	0.402196	0.46661	0.483317	1.04536	ZmPP17	19.9973	16.4668	17.8732	15.4746	1.09345
ZmPP47	112.683	67.4409	92.4163	80.2367	1.04327	ZmPP4	28.9875	28.2602	25.5453	27.193	1.08551
ZmPP16	8.97193	9.7205	8.69455	9.23408	1.0426	ZmPP129	9.44188	6.9871	8.1242	7.02719	1.08432
ZmPP22	17.5356	15.4169	16.9908	14.9159	1.03278	ZmPP106	21.1864	22.4172	18.9966	21.7125	1.0711
ZmPP72	14.4493	10.7897	12.7997	11.7949	1.0262	ZmPP69	9.87477	10.9432	9.98304	9.56157	1.06515
ZmPP30	26.8248	24.107	23.1794	26.4544	1.02615	ZmPP88	32.7136	40.6227	36.3927	32.8025	1.05985
ZmPP84	128.754	137.758	138.201	121.929	1.02453	ZmPP111	47.4697	41.101	37.6262	46.9569	1.04714
ZmPP51	14.9271	14.0861	15.3687	13.0117	1.0223	ZmPP98	39.117	40.6569	40.1608	37.1867	1.03137
ZmPP82	17.2773	14.9322	14.7487	16.8395	1.01967	ZmPP65	69.6937	68.7188	66.9736	68.5842	1.02106
ZmPP56	0	0	0	0	-	ZmPP134	12.1784	10.8482	9.97209	12.7846	1.01186
ZmPP4	0	0	0	0	-	ZmPP136	32.0371	32.32	34.8645	28.9263	1.00888
ZmPP92	0	0	0	0	-	ZmPP97	0	0	0	0	-
ZmPP26	128.794	118.324	0	0	-	ZmPP7	0	0	0	0	-
ZmPP111	0	0	18.0403	18.3423	-	ZmPP2	0	0	0	0	-
ZmPP94	0	0	0	0	-	ZmPP71	0	0	0	0	-
ZmPP102	0	0	0	0	-	ZmPP40	0	0	0	0	-
ZmPP13	5.25889	5.96888	0	0	-	ZmPP113	0	0	0	0	-
ZmPP17	12.2193	12.2176	0	0	-	ZmPP93	0	0	79.8298	69.0419	-
