# Supplementary material for: Maize protein phosphatase gene family: identification and molecular characterization
Source: BMC Genomics. 2014 Sep 9;15(1):773. doi: 10.1186/1471-2164-15-773 (PMC4169795; doi:10.1186/1471-2164-15-773)
Supplement: Supplementary file 19 — Additional file 19: Table S9: List of FPKM values of components involved in ABA biosynthesis and ABA-dependent pathway of response to drought in vegetative tissue (leaf) under both drought and well-watered conditions. MLC and MLD stand for maize basal leaf meristem, well watered and drought, respectively. Numbers 1 and 2 indicate the two biological replicates. The extent of differential expression is measured in terms of fold change and (-) indicates failure to calculate or undetected values. Values in red and blue indicate the fold increase and decrease in expression in the drought-stressed tissue, respectively. (PDF 265 KB) [file 12864_2014_6458_MOESM19_ESM.pdf]

Table S9. List of FPKM values of components involved in ABA biosynthesis and ABA-dependent pathway of response to drought in vegetative tissue (leaf) under both drought and well-watered conditions.under both drought and well-watered conditions.

MLC and MLD stand for maize basal leaf meristem, well watered and drought, respectively.

Numbers 1 and 2 indicate the two biological replicates.

The extent of differential expression is measured in terms of fold change and (-) indicates failure to calculate or undetected values.

Values in red and blue indicate the fold increase and decrease in expression in the drought-stressed tissue, respectively.

Name	MLC.1	MLC.2	MLD.1	MLD.2	leaf
ZmNCED5	0.62933	0.94671	4.61936	7.28051	7.55052562
ZmNCED3	0.16654	1.22934	0.72204	1.02595	1.25224661
VP14	1.79557	2.2555	3.16196	6.01443	2.26517685
ZmAO1	3.60535	3.50057	3.50726	4.49193	1.12570786
ZmABA3	49.8809	39.4595	40.0753	53.0794	1.04269401
ZmAO2	36.9973	55.9415	26.8218	31.9814	1.58050582
ZmZEP	47.2803	55.0909	39.9891	49.3702	1.14561327
ZmNCED2	0.45339	0.13586	0.18707	0.39581	1.01093356
ZmRACR3	6.4989	6.19568	7.64866	6.86369	1.14319261
ZmRACR7	2.06334	4.00065	0.97855	0.59528	3.85302931
ZmRACR6	19.5262	28.7328	12.5767	6.03131	2.59345303
ZmRACR11	14.2379	14.4443	6.43849	5.51184	2.40011782
ZmRACR13	2.3419	4.38102	2.20695	2.25284	1.50745214
ZmRACR1	105.047	126.242	87.539	73.3419	1.43764114
ZmRACR2	62.5026	55.5645	56.2156	47.6209	1.13704815
ZmRACR4	101.254	125.301	109.27	90.3924	1.13469036
ZmRACR5	37.8236	41.0704	39.7535	38.3082	1.01066208
ZmPP130	0.18567	0.06607	1.33133	1.90762	12.8659954
ZmPP154	1.59809	0.3767	5.761	9.98914	7.97561055
ZmPP31	0.20963	0.11168	0.56248	1.0729	5.08978018
ZmPP6	13.2422	5.96945	21.988	27.9786	2.60084896
ZmPP127	12.2224	11.8801	27.1083	24.6031	2.14547869
ZmPP57	18.5235	14.0137	28.2524	33.7509	1.90561265
ZmPP53	19.9122	12.6392	26.7656	25.6849	1.61131319
ZmPP107	21.688	22.6746	34.1335	34.0586	1.53715292
ZmPP132	30.9497	19.4919	37.8076	32.7186	1.39817532
ZmPP121	5.34135	5.80499	6.35177	7.88247	1.27703264
ZmPP39	7.28209	3.93612	6.8885	7.07429	1.24465401
ZmPP123	4.61382	5.01435	5.42137	5.85181	1.17085386
ZmPP134	14.217	12.0266	10.1268	9.54927	1.33378261
ZmPP112	2.86505	0.9147	1.52314	1.73677	1.15946453

ZmPP108	9.12133	13.4001	9.70928	10.2021	1.13108333
ZmSnRK2.1	18.337	9.38266	19.9432	28.3008	1.74042539
ZmSnRK2.11	60.9796	47.7941	76.5369	74.2249	1.38601335
ZmSnRK2.8	36.5374	30.3637	41.7699	44.9881	1.29680977
ZmSnRK2.5	55.4515	66.9464	69.9633	77.8876	1.20795291
ZmSnRK2.9	75.3773	63.5691	77.89	82.4513	1.15397952
ZmSnRK2.2	7.08055	7.60499	7.20634	8.82873	1.09189516
ZmSnRK2.6	34.5763	32.2517	34.982	35.8112	1.05933441
ZmSnRK2.7	22.5727	21.4951	24.0242	21.0826	1.02357731
ZmSnRK2.4	14.7863	14.4142	16.1686	13.2488	1.00742796
ZmSnRK2.12	18.2365	20.7825	13.2286	18.0797	1.24628293
ZmSnRK2.10	44.2821	45.75	40.9253	35.6434	1.17583425
ZmSnRK2.13	43.0578	40.8371	37.9779	37.8255	1.10674323
ZmSnRK2.14	65.5268	59.0061	61.6601	55.4941	1.0629828
ZmSnRK2.3	41.9692	39.0476	39.3765	40.6054	1.01293918
ZmMYB078	0.36451	0.23698	7.49355	10.3334	29.637736
ZmMYB139	0.46439	0.3479	1.86158	2.06539	4.83446717
ZmMYB132	7.80069	1.64123	20.6836	22.4455	4.56783154
ZmMYB007	0.5106	1.21128	3.08386	3.93265	4.07492177
ZmMYB144	6.20284	2.93188	12.7554	23.2718	3.94398515
ZmMYB088	0.74759	0.3357	2.45472	1.68332	3.81991024
ZmMYB123	1.48828	0.47326	1.69531	4.71928	3.27018891
ZmMYB070	4.23764	5.69844	19.3894	12.9287	3.25260062
ZmMYB043	4.2597	1.61696	8.33236	10.4575	3.19737062
ZmMYB085	6.14869	1.85597	11.5525	13.6693	3.15088961
ZmMYB048	0.34564	0.082	0.68843	0.55145	2.89935857
ZmMYB038	0.78626	0.27978	1.64432	1.41122	2.86625536
ZmMYB021	3.015	0.57694	4.10382	4.76696	2.46963823
ZmMYB092	0.56644	0.92719	1.84123	1.83704	2.46262976
ZmMYB030	3.33484	2.6988	7.58137	7.15701	2.44270125
ZmMYB018	0.29348	0.41772	0.5892	1.14068	2.43234735
ZmMYB074	0.5187	0.53007	1.11895	1.09316	2.10924226
ZmMYB027	9.76142	10.535	21.6554	20.4102	2.07256255
ZmMYB109	6.0272	5.48436	11.9609	11.8298	2.06667906
ZmMYB073	18.6937	13.0509	37.9208	23.8547	1.94601602
ZmMYB036	3.13208	2.75592	7.10485	3.56683	1.81244565
ZmMYB095	4.26759	3.44705	6.64936	7.09217	1.78122764
ZmMYB006	0.46231	0.31688	0.68272	0.69295	1.76549221
ZmMYB011	0.92071	0.48762	0.94616	1.36899	1.64389992
ZmMYB113	7.79692	4.86681	9.18829	11.3631	1.6228544
ZmMYB022	0.0976	0.24803	0.27922	0.24934	1.52925246

ZmMYB114	4.0571	1.35393	4.16005	4.02	1.51173621
ZmMYB041	15.953	8.49125	15.1612	20.8633	1.47374127
ZmMYB094	2.6967	0.45298	2.28797	2.31712	1.46208186
ZmMYB068	0.90367	0.18375	1.0285	0.55397	1.45525539
ZmMYB117	2.72546	0.60932	1.63705	3.15111	1.43582485
ZmMYB138	0.64155	0.47832	0.94922	0.60505	1.3879043
ZmMYB028	10.7383	5.91565	11.8495	11.1356	1.38015906
ZmMYB118	7.44262	8.59259	11.3254	9.8542	1.32081837
ZmMYB121	4.76735	2.22465	3.0469	6.16956	1.31814359
ZmMYB052	17.9112	10.6115	20.3097	15.9475	1.27116998
ZmMYB064	3.68302	0.48095	3.35958	1.80686	1.2407501
ZmMYB133	1.33065	1.04226	1.34742	1.32549	1.12642705
ZmMYB142	6.79843	5.82314	7.07472	6.95568	1.11162082
ZmMYB148	1.58115	1.0172	1.47462	1.39389	1.10397368
ZmMYB039	13.7325	9.84747	16.4193	8.64131	1.06279228
ZmMYB112	1.4216	1.67282	1.61539	1.65844	1.05797855
ZmMYB135	0.42447	0.03804	0.21875	0.26297	1.04155865
ZmMYB149	2.80876	1.47496	2.33477	2.10246	1.03583568
ZmMYB062	0.44478	0.46338	0.50181	0.43721	1.03398983
ZmMYB140	0.68791	0.68137	0.68479	0.73038	1.03350958
ZmMYB141	0.84256	0.78727	0.80292	0.8724	1.02791034
ZmMYB081	19.2582	21.3786	21.8708	19.0773	1.00766054
ZmMYB125	8.78834	7.06702	8.28961	7.5692	1.00021759
ZmMYB101	297.08	1177.27	229.797	160.549	3.77703371
ZmMYB015	31.8726	43.0598	10.7184	10.5171	3.52863837
ZmMYB042	43.4511	74.2362	18.2417	16.2906	3.40803538
ZmMYB071	5.14386	1.40586	0.71054	1.3945	3.11144238
ZmMYB026	4.41967	2.46079	1.26674	1.13236	2.86793381
ZmMYB008	5.797	5.30974	2.21016	1.769	2.79122729
ZmMYB090	6.18198	9.43112	2.95644	3.10261	2.5768231
ZmMYB076	38.3842	51.7332	22.8966	12.6884	2.53245469
ZmMYB104	18.9333	20.5158	8.45762	7.64152	2.45038555
ZmMYB122	3.9181	6.9636	2.7537	2.38041	2.11949101
ZmMYB111	10.5205	7.37358	6.2158	2.61316	2.02674834
ZmMYB143	12.3023	4.05625	4.0059	4.20127	1.99320229
ZmMYB075	1.40706	0.51684	0.56412	0.40453	1.9861848
ZmMYB024	5.29298	1.51077	1.72882	1.81018	1.92250636
ZmMYB091	38.5306	69.1644	35.3546	24.8864	1.78773593
ZmMYB016	2.79071	0.79852	1.05353	0.99204	1.75463122
ZmMYB107	0.32002	0.6051	0.44401	0.10736	1.67787277
ZmMYB066	3.83129	5.23522	2.69586	2.85309	1.63391452
ZmMYB065	2.47891	1.25297	0.96728	1.31797	1.63302707
ZmMYB150	2.58894	0.12913	0.94502	0.80851	1.55005244

ZmMYB100	4.80165	5.12041	3.24733	3.16485	1.54737702
ZmMYB084	2.35057	2.38366	1.80194	1.28418	1.53403951
ZmMYB017	1.63267	1.07798	1.07202	0.70859	1.5223171
ZmMYB098	12.5813	13.6882	9.37498	8.42583	1.47574745
ZmMYB103	12.5813	13.6882	9.37498	8.42583	1.47574745
ZmMYB029	13.856	15.5195	10.5416	9.70074	1.45119092
ZmMYB108	9.50043	5.04737	4.7308	5.89831	1.36867527
ZmMYB106	35.8632	33.6201	31.5895	22.0518	1.29533214
ZmMYB119	8.80026	13.0191	9.08303	7.94514	1.28136846
ZmMYB040	14.8308	4.74497	7.35195	8.66556	1.22214814
ZmMYB156	2.09666	0.93443	1.21365	1.28617	1.2125213
ZmMYB069	0.57741	1.56154	1.20065	0.56464	1.21166902
ZmMYB005	0.26938	0.76687	0.37558	0.49277	1.19335841
ZmMYB151	0.45099	0.944	0.65942	0.55168	1.15183697
ZmMYB126	3.09456	0.65205	1.43934	1.84292	1.14147143
ZmMYB093	5.29466	3.64507	3.55177	4.48149	1.11283962
ZmMYB049	3.41012	3.71646	3.2866	3.15478	1.10637472
ZmMYB086	0.47957	0.29254	0.52398	0.20353	1.06130775
ZmMYB023	0.48966	0.6934	0.64861	0.48435	1.04422863
ZmMYB010	19.8665	31.1161	28.1433	21.1307	1.03467549
ZmMYB110	24.4323	20.131	28.2073	15.2173	1.02622246
ZmMYB131	9.75473	9.25257	10.0046	8.61437	1.02085669
ZmMYB072	1.28874	0.0096	0.35479	0.93544	1.0062927
ZmMYC8	5.50526	3.68347	56.2334	83.0302	15.1559138
ZmMYC7	20.8406	19.8705	71.7191	95.4893	4.10719435
ZmMYC4	7.98938	6.56123	20.7688	26.5491	3.25195301
ZmMYC5	4.82167	1.67122	5.37637	5.50085	1.67525093
ZmMYC3	14.2999	16.2847	17.0407	18.8812	1.17450939
ZmMYC1	33.3698	13.837	2.87612	2.29736	9.12476708
ZmMYC2	182.915	58.8024	13.2296	19.9174	7.29228588
ZmMYC6	8.10959	7.63966	6.62082	5.97033	1.25081903
ZmWRKY109	0.46629	0.33185	3.53445	5.78835	11.6807014
ZmWRKY85	0.39591	0.4004	4.6765	4.488	11.5087523
ZmWRKY61	85.8389	90.7044	318.309	385.829	3.98847195
ZmWRKY94	16.8465	7.7075	43.9716	46.4398	3.68214548
ZmWRKY103	0.64725	0.69159	1.82744	2.30488	3.08648356
ZmWRKY112	112.37	23.7532	195.175	180.714	2.76138821
ZmWRKY79	95.1914	45.4389	158.734	187.019	2.45859534
ZmWRKY9	4.49523	6.59772	13.2492	9.37576	2.03958009
ZmWRKY33	2.02748	2.24921	4.04563	4.61688	2.0255174
ZmWRKY42	1.17895	1.00184	1.9346	2.4395	2.00574104

ZmWRKY12	3.11228	1.7073	4.26978	4.80174	1.8822221
ZmWRKY64	0.73463	0.532	1.0227	1.30181	1.8351897
ZmWRKY83	7.40744	5.95464	19.0804	5.01692	1.80341085
ZmWRKY80	35.452	14.1855	47.1057	39.2222	1.73916696
ZmWRKY101	2.21477	2.02372	3.79973	3.13307	1.63567686
ZmWRKY93	13.2381	5.37843	15.0938	15.198	1.62714534
ZmWRKY73	20.9058	18.9992	39.6238	25.2844	1.6265681
ZmWRKY113	46.2962	28.4932	56.2427	63.41	1.59986175
ZmWRKY24	2.08486	2.3135	3.43815	3.44791	1.56559718
ZmWRKY21	11.2783	10.2819	15.7284	17.3495	1.53421119
ZmWRKY77	14.7325	19.4428	26.0813	23.9489	1.46392863
ZmWRKY60	9.65812	12.3963	16.4462	15.2636	1.43779796
ZmWRKY8	4.25197	3.91233	6.20449	5.47764	1.43087956
ZmWRKY20	9.95266	8.85841	13.079	13.7814	1.42790389
ZmWRKY38	34.4158	33.651	55.9534	38.4693	1.38720639
ZmWRKY115	313.907	300.489	480.797	368.901	1.38298101
ZmWRKY15	1.64246	1.38682	2.07569	2.07669	1.37074816
ZmWRKY96	4.61178	5.89036	8.33827	5.49246	1.31694398
ZmWRKY19	0.34831	0.09916	0.1332	0.44839	1.29975439
ZmWRKY110	7.55117	5.13758	8.25837	8.17892	1.29542232
ZmWRKY98	0.8233	0.83264	0.89068	1.24439	1.28933172
ZmWRKY51	1.40336	0.45694	1.00844	1.27162	1.22563839
ZmWRKY34	8.12413	10.2146	11.1165	11.3301	1.2239997
ZmWRKY59	8.20072	4.84478	8.4655	7.29404	1.20804415
ZmWRKY57	3.47086	3.06005	4.29641	3.59097	1.2077
ZmWRKY111	3.71656	1.73035	3.29874	3.18028	1.18948541
ZmWRKY2	0.75777	0.40912	0.6745	0.69865	1.17674885
ZmWRKY25	5.20535	5.74467	7.57851	5.08298	1.15629834
ZmWRKY17	13.235	14.7469	15.6131	16.4192	1.14475071
ZmWRKY68	46.1683	67.3684	71.4477	52.3077	1.0900035
ZmWRKY82	22.7641	22.8555	23.8221	25.1898	1.07436058
ZmWRKY54	0.40376	0.22988	0.2702	0.39983	1.05744019
ZmWRKY1	8.04162	4.89421	6.82335	6.50719	1.03051292
ZmWRKY100	1.07575	1.34952	0.3312	0.14445	5.09893996
ZmWRKY29	2.64074	2.64856	1.27192	1.29687	2.05906283
ZmWRKY39	16.8827	18.7772	9.68426	8.32029	1.9806049
ZmWRKY41	2.39989	2.6687	1.43399	1.13875	1.97011358
ZmWRKY72	1.01843	0.65397	0.66959	0.24485	1.82888416
ZmWRKY116	0.456	0.55894	0.51997	0.04859	1.78509392
ZmWRKY13	23.1891	28.1663	15.9621	13.9323	1.71789365
ZmWRKY10	1.47513	1.37888	0.9472	0.71949	1.71238716
ZmWRKY119	31.2341	31.4516	21.4001	15.4381	1.70164937
ZmWRKY7	3.23664	2.59477	2.28405	1.18313	1.68188845

ZmWRKY65	7.85328	8.40159	5.27591	4.44391	1.6723427
ZmWRKY31	8.00229	8.26221	5.46083	4.60956	1.61508144
ZmWRKY63	0.3566	0.57102	0.72446	0.70626	1.54235121
ZmWRKY46	3.95376	6.12915	3.34955	3.25627	1.52636766
ZmWRKY76	2.22397	2.55346	1.71294	1.44508	1.51279283
ZmWRKY90	40.3453	90.5299	48.5456	39.3594	1.48882544
ZmWRKY58	7.61986	7.03409	5.11582	4.77782	1.4811485
ZmWRKY71	0.67353	0.72116	0.70607	0.27675	1.41907871
ZmWRKY4	2.95223	3.44592	2.44346	2.10415	1.40692584
ZmWRKY62	1.4945	1.68927	1.20817	1.06644	1.39969929
ZmWRKY56	1.96876	3.06814	1.84749	1.76483	1.39436705
ZmWRKY70	33.5666	59.7265	36.8117	31.3749	1.36820284
ZmWRKY22	31.8059	29.2978	24.5894	20.5337	1.35415563
ZmWRKY48	9.86635	9.32924	6.49948	7.71613	1.35031771
ZmWRKY6	15.2129	18.0396	12.5246	12.5505	1.32611635
ZmWRKY53	1.40512	1.11111	1.24383	0.72149	1.28031763
ZmWRKY99	1.12577	1.14311	0.95713	0.83023	1.2694086
ZmWRKY114	14.7954	10.3799	9.02771	11.5787	1.22172178
ZmWRKY74	0.49501	0.91182	0.52893	0.63437	1.20935052
ZmWRKY106	17.4794	19.6753	17.2616	13.8647	1.19367544
ZmWRKY92	37.1598	56.0039	43.9462	34.1269	1.19328809
ZmWRKY102	11.0135	9.71195	10.6711	6.72007	1.19172258
ZmWRKY30	9.08967	6.83654	6.82953	6.68617	1.17834888
ZmWRKY52	10.1831	12.9756	12.4054	14.0459	1.14217551
ZmWRKY23	10.1431	10.6467	8.688	9.6748	1.13216939
ZmWRKY81	13.5669	13.4099	11.3913	12.6114	1.12390689
ZmWRKY88	4.57697	1.87766	3.19908	2.55372	1.12199798
ZmWRKY78	73.6462	112.338	94.9595	71.6976	1.11596926
ZmWRKY91	73.6502	112.355	94.9921	71.7208	1.1157217
ZmWRKY44	0.24231	0.47162	0.44101	0.20897	1.09838288
ZmWRKY27	1.45203	2.15172	1.88262	1.4379	1.08529688
ZmWRKY28	2.44161	2.51228	2.1374	2.47611	1.07377897
ZmWRKY47	12.2781	10.3947	10.4926	10.7131	1.06918423
ZmWRKY50	3.55365	2.56515	3.10404	2.661	1.06136297
ZmWRKY26	3.13925	4.97089	6.08765	1.58715	1.0567233
ZmbZIP84	0.43231	0.30767	5.86895	7.96276	18.6919041
ZmbZIP6	1.16223	0.34625	3.82436	3.47951	4.84188999
ZmbZIP100	0.43542	0.08274	0.75418	0.89369	3.18021707
ZmbZIP69	0.9398	0	1.56203	1.24216	2.98381571
ZmbZIP106	1.82414	0.23588	3.69536	2.30279	2.91170069
ZmbZIP41	1.7	0.32504	3.65077	1.89702	2.73959797
ZmbZIP43	1.84878	1.11175	3.25008	4.00592	2.45091251

ZmbZIP35	17.5375	17.9373	41.7772	43.9754	2.41728213
ZmbZIP37	2.29609	2.38092	5.24958	5.75879	2.35371958
ZmbZIP36	9.49545	9.98699	23.2663	21.9625	2.3215162
ZmbZIP124	1.08669	0.79833	2.3268	1.97867	2.2840411
ZmbZIP42	0.77268	0.51925	1.49679	1.42244	2.25958837
ZmbZIP32	2.39642	2.08707	4.79319	4.29676	2.02742729
ZmbZIP1	0.74331	0.62172	1.57161	0.95907	1.85393519
ZmbZIP56	1.00032	0.04593	0.77125	1.08645	1.77557563
ZmbZIP13	13.8822	13.1816	24.0237	23.4852	1.75544085
ZmbZIP92	2.2754	3.03724	4.64177	4.09859	1.64520088
ZmbZIP53	3.4118	2.95725	5.38441	4.86115	1.60864807
ZmbZIP19	1.07438	0.34089	1.14409	1.09085	1.57915823
ZmbZIP83	16.2687	15.8841	24.6327	25.583	1.56178311
ZmbZIP70	6.29082	4.21744	7.04737	8.48336	1.47795449
ZmbZIP95	0.83304	0.17566	0.79641	0.64161	1.42562167
ZmbZIP14	9.95715	8.17074	12.9311	12.2427	1.38867789
ZmbZIP55	11.8584	9.98093	15.5832	14.5622	1.38032623
ZmbZIP46	6.09383	5.50396	8.14348	7.77714	1.37272877
ZmbZIP5	6.77865	5.67765	8.45877	7.85342	1.3095534
ZmbZIP20	18.8282	16.4287	22.7506	22.5262	1.28419685
ZmbZIP47	3.03593	2.21255	2.86339	3.56343	1.22451072
ZmbZIP21	8.36574	6.57641	7.96724	9.74301	1.18525446
ZmbZIP28	19.733	19.7826	23.4429	23.0921	1.17763617
ZmbZIP113	3.55836	0.62465	2.78898	2.12437	1.17459592
ZmbZIP102	7.73061	5.62325	6.78456	8.86284	1.17175109
ZmbZIP73	29.4954	26.2532	34.8015	30.1815	1.16564362
ZmbZIP119	15.2035	11.5236	15.1044	14.881	1.12190997
ZmbZIP16	11.8918	13.5759	15.2348	13.1562	1.11478461
ZmbZIP17	11.8918	13.5759	15.2348	13.1562	1.11478461
ZmbZIP2	20.8042	19.9512	22.9859	21.4847	1.09115847
ZmbZIP86	4.78169	4.76054	5.16326	5.21395	1.08750365
ZmbZIP65	37.3511	44.6348	44.6348	43.2089	1.07144887
ZmbZIP107	8.47749	7.66336	8.14744	8.64044	1.04008649
ZmbZIP90	7.51725	6.8572	7.33668	7.58561	1.03811207
ZmbZIP48	1.53654	1.22689	1.43316	1.41025	1.02894229
ZmbZIP91	0.80617	0.61602	0.80462	0.64789	1.02131715
ZmbZIP9	59.231	184.83	49.2631	13.1412	3.91096447
ZmbZIP44	26.6806	20.8194	6.71486	7.28813	3.39213268
ZmbZIP22	54.0905	49.6488	18.6616	20.8049	2.62854066
ZmbZIP7	24.6749	29.7253	13.0836	7.66196	2.62225749
ZmbZIP87	150.611	193.08	69.8106	66.8718	2.51452272
ZmbZIP121	2.59467	1.88698	1.06482	0.85994	2.3284238
ZmbZIP82	9.6966	12.7359	5.8152	4.77157	2.11891823

ZmbZIP88	7.01374	7.67412	3.78603	3.49636	2.01690104
ZmbZIP108	8.59122	4.40667	3.77378	2.87419	1.95516677
ZmbZIP105	2.15133	3.26567	1.53992	1.29549	1.91048208
ZmbZIP8	138.946	114.575	70.794	71.9543	1.77600013
ZmbZIP81	11.0213	9.46859	7.23068	4.73756	1.71202198
ZmbZIP67	35.4644	10.1259	14.6604	12.5617	1.67475323
ZmbZIP45	30.3006	31.0442	18.6359	17.9948	1.67468271
ZmbZIP31	38.3861	28.6641	24.2478	16.3045	1.65342533
ZmbZIP50	1.51139	2.939	1.34195	1.35977	1.64724324
ZmbZIP97	32.3217	15.5259	14.6085	14.4568	1.64621043
ZmbZIP94	1.21971	1.33063	0.84093	0.72703	1.62653073
ZmbZIP59	16.9394	23.6404	14.3127	11.4901	1.57268979
ZmbZIP38	3.62642	5.33409	3.2242	2.50636	1.56363601
ZmbZIP112	86.7683	121.103	79.2577	54.1783	1.55783522
ZmbZIP40	2.15771	2.81595	1.95675	1.24598	1.55294389
ZmbZIP125	26.962	19.7805	16.1713	14.8581	1.50639394
ZmbZIP111	5.67477	5.21965	3.86856	3.38097	1.50277604
ZmbZIP117	1.24823	0.91373	0.88649	0.56507	1.48940553
ZmbZIP122	0.44203	0.22471	0.39241	0.06253	1.46553202
ZmbZIP68	3.60198	4.98967	2.92401	3.18935	1.40538918
ZmbZIP23	55.6491	56.6068	47.2566	35.1114	1.36285815
ZmbZIP103	4.3928	4.68866	3.66771	3.04432	1.35301243
ZmbZIP79	13.3826	13.1928	10.2487	9.5421	1.34281585
ZmbZIP33	19.8583	24.4089	17.8746	15.819	1.31381627
ZmbZIP64	26.08	27.5307	22.3149	20.0661	1.26497015
ZmbZIP34	16.7417	15.237	12.9298	13.219	1.22295096
ZmbZIP18	9.5335	6.31775	6.03106	6.93857	1.22218213
ZmbZIP71	0.33719	0.21883	0.2798	0.18354	1.20001683
ZmbZIP98	7.73521	6.77845	7.0267	5.11116	1.19573467
ZmbZIP39	27.4974	28.0702	21.73	24.9819	1.18958124
ZmbZIP49	1.71442	2.72182	1.32384	2.416	1.18621117
ZmbZIP74	62.1063	72.3442	62.9911	51.3309	1.17606847
ZmbZIP4	35.2404	40.3305	35.0426	29.2922	1.17465042
ZmbZIP96	9.74165	9.23455	8.07512	8.14767	1.16972481
ZmbZIP12	0.62382	0.79435	0.38644	0.84402	1.15255221
ZmbZIP114	27.6806	32.5734	28.2177	24.9062	1.13421643
ZmbZIP99	12.1473	13.7542	10.9748	11.8844	1.13308865
ZmbZIP76	17.1975	16.359	14.9068	14.9882	1.12247868
ZmbZIP26	10.9654	10.7342	9.38225	10.0233	1.11821618
ZmbZIP120	19.0813	19.4953	19.6432	15.5046	1.09755376
ZmbZIP93	63.676	64.5058	65.4251	53.4145	1.07861184
ZmbZIP110	89.5636	78.8987	83.1657	74.7613	1.06670994
ZmbZIP58	18.325	21.1005	19.1721	18.0905	1.0580448

ZmbZIP63	3.79831	4.30785	4.03213	3.70083	1.04826095
ZmbZIP123	2.79496	2.00902	2.54147	2.05935	1.04415735
ZmbZIP101	1.03145	1.06617	1.01199	1.02646	1.02902696
ZmbZIP60	17.3119	20.3538	17.8195	19.0778	1.02082537
ZmbZIP62	28.127	31.8442	0	0	-
