# Supplementary material for: Maize protein phosphatase gene family: identification and molecular characterization
Source: BMC Genomics. 2014 Sep 9;15(1):773. doi: 10.1186/1471-2164-15-773 (PMC4169795; doi:10.1186/1471-2164-15-773)
Supplement: Supplementary file 20 — Additional file 20: Table S10: List of putative components involved in ABA biosynthesis and ABA-dependent pathway of response to drought in maize. (PDF 270 KB) [file 12864_2014_6458_MOESM20_ESM.pdf]

Table S10. List of putative components involved in ABA biosynthesis and ABA-dependent pathway of response to drought in maize.

Name	Gene ID	Protein ID	Class
ZmZEP1	GRMZM2G127139	GRMZM2G127139_P01	ZEP
ZmZEP2	GRMZM2G127139	GRMZM2G127139_P02	ZEP
VP14	GRMZM2G014392	GRMZM2G014392_P01	NCED
ZmNCED3	GRMZM5G838285	GRMZM5G838285_P01	NCED
ZmNCED5	GRMZM2G417954	GRMZM2G417954_P01	NCED
ZmABA3	GRMZM2G081571	GRMZM2G081571_P04	ABA3
ZmAO1	GRMZM2G141535	GRMZM2G141535_P01	AAO
ZmAO2	GRMZM2G141473	GRMZM2G141473_P01	AAO
ZmRACR1	GRMZM2G063882	GRMZM2G063882_P01	ABA receptor
ZmRACR2	GRMZM2G048733	GRMZM2G048733_P02	ABA receptor
ZmRACR3	GRMZM2G405064	GRMZM2G405064_P01	ABA receptor
ZmRACR4	GRMZM2G165567	GRMZM2G165567_P02	ABA receptor
ZmRACR5	GRMZM2G133631	GRMZM2G133631_P01	ABA receptor
ZmRACR6	GRMZM2G144224	GRMZM2G144224_P01	ABA receptor
ZmRACR7	GRMZM2G057959	GRMZM2G057959_P01	ABA receptor
ZmRACR8	GRMZM2G047677	GRMZM2G047677_P01	ABA receptor
ZmRACR9	GRMZM2G169695	GRMZM2G169695_P01	ABA receptor
ZmRACR10	GRMZM2G141382	GRMZM2G141382_P01	ABA receptor
ZmRACR11	GRMZM2G134731	GRMZM2G134731_P01	ABA receptor
ZmRACR12	AC194914.3_FG002	AC194914.3_FGP002	ABA receptor
ZmRACR13	GRMZM2G154987	GRMZM2G154987_P01	ABA receptor
ZmPP6	GRMZM2G010855	GRMZM2G010855_P01	PP2C
ZmPP31	GRMZM2G019819	GRMZM2G019819_P01	PP2C
ZmPP39	GRMZM2G082487	GRMZM2G082487_P01	PP2C
ZmPP53	GRMZM2G059453	GRMZM2G059453_P01	PP2C
ZmPP56	GRMZM2G122228	GRMZM2G122228_P01	PP2C
ZmPP57	GRMZM2G134628	GRMZM2G134628_P01	PP2C
ZmPP107	GRMZM2G177386	GRMZM2G177386_P02	PP2C
ZmPP108	GRMZM2G102255	GRMZM2G102255_P01	PP2C
ZmPP112	GRMZM2G308615	GRMZM2G308615_P01	PP2C
ZmPP121	GRMZM5G818101	GRMZM5G818101_P02	PP2C
ZmPP123	GRMZM2G383807	GRMZM2G383807_P01	PP2C
ZmPP127	GRMZM2G001243	GRMZM2G001243_P01	PP2C
ZmPP130	GRMZM2G300125	GRMZM2G300125_P01	PP2C
ZmPP132	GRMZM2G166297	GRMZM2G166297_P01	PP2C
ZmPP134	GRMZM2G149132	GRMZM2G149132_P01	PP2C
ZmPP154	GRMZM2G159811	GRMZM2G159811_P01	PP2C
ZmPP8	AC208201.3_FG002	AC208201.3_FGP002	PP2C
ZmPP43	GRMZM2G342197	GRMZM2G342197_P01	PP2C
ZmPP60	GRMZM2G407605	GRMZM2G407605_P01	PP2C

ZmPP61	GRMZM2G407623	GRMZM2G407623_P01	PP2C
ZmPP97	GRMZM2G000603	GRMZM2G000603_P01	PP2C
ZmPP147	GRMZM2G465287	GRMZM2G465287_P01	PP2C
ZmPP7	GRMZM2G112925	GRMZM2G112925_P01	PP2C
ZmPP24	GRMZM2G093776	GRMZM2G093776_P01	PP2C
ZmPP30	GRMZM2G016749	GRMZM2G016749_P01	PP2C
ZmPP77	GRMZM2G144109	GRMZM2G144109_P01	PP2C
ZmPP126	GRMZM2G141859	GRMZM2G141859_P01	PP2C
ZmPP148	GRMZM2G103247	GRMZM2G103247_P01	PP2C
ZmPP155	GRMZM2G443509	GRMZM2G443509_P01	PP2C
ZmPP2	AC155624.2_FG006	AC155624.2_FGP006	PP2C
ZmPP3	GRMZM5G833774	GRMZM5G833774_P01	PP2C
ZmPP4	GRMZM5G891266	GRMZM5G891266_P01	PP2C
ZmPP21	GRMZM2G050512	GRMZM2G050512_P01	PP2C
ZmPP25	GRMZM2G156543	GRMZM2G156543_P01	PP2C
ZmPP28	GRMZM2G479665	GRMZM2G479665_P01	PP2C
ZmPP80	GRMZM2G377904	GRMZM2G377904_P01	PP2C
ZmPP83	GRMZM2G069970	GRMZM2G069970_P01	PP2C
ZmPP92	GRMZM2G057907	GRMZM2G057907_P01	PP2C
ZmPP99	GRMZM2G151254	GRMZM2G151254_P01	PP2C
ZmPP144	GRMZM2G108309	GRMZM2G108309_P01	PP2C
ZmPP149	GRMZM2G130943	GRMZM2G130943_P02	PP2C
ZmPP151	GRMZM2G010017	GRMZM2G010017_P01	PP2C
ZmPP12	GRMZM2G170299	GRMZM2G170299_P01	PP2C
ZmPP35	GRMZM2G153675	GRMZM2G153675_P01	PP2C
ZmPP41	GRMZM2G021610	GRMZM2G021610_P01	PP2C
ZmPP63	GRMZM2G120246	GRMZM2G120246_P01	PP2C
ZmPP69	GRMZM2G071196	GRMZM2G071196_P01	PP2C
ZmPP70	GRMZM2G067910	GRMZM2G067910_P01	PP2C
ZmPP71	GRMZM2G053722	GRMZM2G053722_P01	PP2C
ZmPP74	GRMZM2G040452	GRMZM2G040452_P03	PP2C
ZmPP87	GRMZM2G119623	GRMZM2G119623_P01	PP2C
ZmPP89	GRMZM2G102560	GRMZM2G102560_P02	PP2C
ZmPP105	GRMZM2G069713	GRMZM2G069713_P01	PP2C
ZmPP116	GRMZM2G107565	GRMZM2G107565_P01	PP2C
ZmPP117	GRMZM2G161544	GRMZM2G161544_P01	PP2C
ZmPP128	GRMZM2G136765	GRMZM2G136765_P01	PP2C
ZmPP27	GRMZM2G111232	GRMZM2G111232_P01	PP2C
ZmPP40	GRMZM2G311187	GRMZM2G311187_P01	PP2C
ZmPP115	GRMZM2G010298	GRMZM2G010298_P01	PP2C
ZmPP152	GRMZM2G074489	GRMZM2G074489_P02	PP2C
ZmPP158	GRMZM2G155991	GRMZM2G155991_P01	PP2C
ZmPP159	GRMZM2G180471	GRMZM2G180471_P01	PP2C

ZmPP26	GRMZM2G056572	GRMZM2G056572_P02	PP2C
ZmPP72	GRMZM2G073788	GRMZM2G073788_P01	PP2C
ZmPP76	GRMZM2G166035	GRMZM2G166035_P01	PP2C
ZmPP84	GRMZM5G829894	GRMZM5G829894_P01	PP2C
ZmPP86	GRMZM2G442404	GRMZM2G442404_P01	PP2C
ZmPP109	GRMZM2G412937	GRMZM2G412937_P01	PP2C
ZmPP122	GRMZM2G158818	GRMZM2G158818_P01	PP2C
ZmPP129	GRMZM2G040642	GRMZM2G040642_P01	PP2C
ZmPP10	GRMZM2G057768	GRMZM2G057768_P02	PP2C
ZmPP66	GRMZM2G108147	GRMZM2G108147_P02	PP2C
ZmPP95	GRMZM2G180430	GRMZM2G180430_P01	PP2C
ZmPP111	GRMZM2G126832	GRMZM2G126832_P02	PP2C
ZmPP137	GRMZM2G158734	GRMZM2G158734_P01	PP2C
ZmPP143	GRMZM2G009163	GRMZM2G009163_P01	PP2C
ZmPP153	GRMZM2G060798	GRMZM2G060798_P01	PP2C
ZmPP9	GRMZM2G077960	GRMZM2G077960_P01	PP2C
ZmPP29	GRMZM2G015610	GRMZM2G015610_P01	PP2C
ZmPP42	GRMZM5G836628	GRMZM5G836628_P02	PP2C
ZmPP49	GRMZM2G173734	GRMZM2G173734_P01	PP2C
ZmPP64	GRMZM2G081359	GRMZM2G081359_P03	PP2C
ZmPP90	GRMZM2G052699	GRMZM2G052699_P01	PP2C
ZmPP113	GRMZM2G135444	GRMZM2G135444_P01	PP2C
ZmPP118	GRMZM2G113016	GRMZM2G113016_P01	PP2C
ZmPP141	GRMZM2G150213	GRMZM2G150213_P01	PP2C
ZmPP146	GRMZM2G006429	GRMZM2G006429_P01	PP2C
ZmPP156	GRMZM2G044382	GRMZM2G044382_P01	PP2C
ZmPP85	GRMZM2G360455	GRMZM2G360455_P02	PP2C
ZmPP100	GRMZM2G019812	GRMZM2G019812_P01	PP2C
ZmPP114	GRMZM2G006416	GRMZM2G006416_P02	PP2C
ZmPP138	GRMZM2G047376	GRMZM2G047376_P01	PP2C
ZmPP139	GRMZM2G104076	GRMZM2G104076_P02	PP2C
ZmPP157	GRMZM2G109496	GRMZM2G109496_P05	PP2C
ZmPP23	GRMZM2G134227	GRMZM2G134227_P01	PP2C
ZmPP46	GRMZM2G003096	GRMZM2G003096_P01	PP2C
ZmPP78	AC210013.4_FG011	AC210013.4_FGP011	PP2C
ZmPP82	GRMZM2G071087	GRMZM2G071087_P01	PP2C
ZmPP91	GRMZM2G159904	GRMZM2G159904_P01	PP2C
ZmPP142	AC217887.3_FG001	AC217887.3_FGP001	PP2C
ZmPP44	GRMZM2G053713	GRMZM2G053713_P01	PP2C
ZmPP37	GRMZM2G150608	GRMZM2G150608_P01	PP2C
ZmPP58	GRMZM2G372297	GRMZM2G372297_P01	PP2C
ZmPP110	GRMZM2G042627	GRMZM2G042627_P01	PP2C
ZmSnRK2.1	GRMZM2G180916	GRMZM2G180916_P04	SnRK2

ZmSnRK2.2	GRMZM2G081915	GRMZM2G081915_P02	SnRK2
ZmSnRK2.3	GRMZM2G138861	GRMZM2G138861_P04	SnRK2
ZmSnRK2.4	GRMZM2G155593	GRMZM2G155593_P01	SnRK2
ZmSnRK2.5	GRMZM2G110922	GRMZM2G110922_P01	SnRK2
ZmSnRK2.6	GRMZM2G130018	GRMZM2G130018_P01	SnRK2
ZmSnRK2.7	GRMZM2G171435	GRMZM2G171435_P01	SnRK2
ZmSnRK2.8	GRMZM2G066867	GRMZM2G066867_P01	SnRK2
ZmSnRK2.9	GRMZM2G000278	GRMZM2G000278_P03	SnRK2
ZmSnRK2.10	GRMZM2G063961	GRMZM2G063961_P01	SnRK2
ZmSnRK2.11	GRMZM2G056732	GRMZM2G056732_P02	SnRK2
ZmSnRK2.12	GRMZM2G334791	GRMZM2G334791_P03	SnRK2
ZmSnRK2.13	GRMZM2G035809	GRMZM2G035809_P01	SnRK2
ZmSnRK2.14	GRMZM2G110908	GRMZM2G110908_P01	SnRK2
ZmWRKY1	GRMZM2G030272	GRMZM2G030272_P01	WRKY
ZmWRKY2	GRMZM2G425430	GRMZM2G425430_P01	WRKY
ZmWRKY3	GRMZM2G383594	GRMZM2G383594_P01	WRKY
ZmWRKY4	GRMZM2G130374	GRMZM2G130374_P01	WRKY
ZmWRKY5	GRMZM2G324999	GRMZM2G324999_P01	WRKY
ZmWRKY6	GRMZM2G070211	GRMZM2G070211_P01	WRKY
ZmWRKY7	GRMZM2G149219	GRMZM2G149219_P01	WRKY
ZmWRKY8	GRMZM2G018487	GRMZM2G018487_P01	WRKY
ZmWRKY9.1	GRMZM2G143204	GRMZM2G143204_P01	WRKY
ZmWRKY9.2	GRMZM2G143204	GRMZM2G143204_P02	WRKY
ZmWRKY10	GRMZM2G008029	GRMZM2G008029_P01	WRKY
ZmWRKY11	GRMZM2G164082	GRMZM2G164082_P01	WRKY
ZmWRKY12	GRMZM2G083717	GRMZM2G083717_P01	WRKY
ZmWRKY13.1	GRMZM2G071907	GRMZM2G071907_P03	WRKY
ZmWRKY13.2	GRMZM2G071907	GRMZM2G071907_P01	WRKY
ZmWRKY14	GRMZM2G024898	GRMZM2G024898_P01	WRKY
ZmWRKY15.1	GRMZM2G123387	GRMZM2G123387_P01	WRKY
ZmWRKY15.2	GRMZM2G123387	GRMZM2G123387_P02	WRKY
ZmWRKY16	GRMZM5G816457	GRMZM5G816457_P01	WRKY
ZmWRKY17	GRMZM2G102583	GRMZM2G102583_P02	WRKY
ZmWRKY18	GRMZM2G400559	GRMZM2G400559_P01	WRKY
ZmWRKY19	GRMZM2G099593	GRMZM2G099593_P01	WRKY
ZmWRKY20	GRMZM2G163418	GRMZM2G163418_P01	WRKY
ZmWRKY21	GRMZM2G057011	GRMZM2G057011_P01	WRKY
ZmWRKY22	GRMZM2G052671	GRMZM2G052671_P01	WRKY
ZmWRKY23	GRMZM2G130854	GRMZM2G130854_P01	WRKY
ZmWRKY24	GRMZM2G106560	GRMZM2G106560_P01	WRKY
ZmWRKY25.1	GRMZM5G871347	GRMZM5G871347_P01	WRKY
ZmWRKY25.2	GRMZM5G871347	GRMZM5G871347_P02	WRKY
ZmWRKY25.3	GRMZM5G871347	GRMZM5G871347_P03	WRKY

ZmWRKY26	GRMZM2G475984	GRMZM2G475984_P01	WRKY
ZmWRKY27	GRMZM2G176489	GRMZM2G176489_P01	WRKY
ZmWRKY28	GRMZM2G151444	GRMZM2G151444_P01	WRKY
ZmWRKY29	GRMZM2G327349	GRMZM2G327349_P01	WRKY
ZmWRKY30	GRMZM2G173680	GRMZM2G173680_P01	WRKY
ZmWRKY31	GRMZM2G076657	GRMZM2G076657_P01	WRKY
ZmWRKY32	AC165171.2_FG002	AC165171.2_FGP002	WRKY
ZmWRKY33	GRMZM2G148087	GRMZM2G148087_P01	WRKY
ZmWRKY34	GRMZM2G059562	GRMZM2G059562_P01	WRKY
ZmWRKY35	GRMZM2G158328	GRMZM2G158328_P01	WRKY
ZmWRKY36	GRMZM2G065290	GRMZM2G065290_P01	WRKY
ZmWRKY37	GRMZM2G382350	GRMZM2G382350_P01	WRKY
ZmWRKY38.1	GRMZM2G141299	GRMZM2G141299_P02	WRKY
ZmWRKY38.2	GRMZM2G141299	GRMZM2G141299_P01	WRKY
ZmWRKY39	GRMZM2G040298	GRMZM2G040298_P01	WRKY
ZmWRKY40	GRMZM2G101405	GRMZM2G101405_P01	WRKY
ZmWRKY41	GRMZM2G151763	GRMZM2G151763_P01	WRKY
ZmWRKY42	GRMZM2G408462	GRMZM2G408462_P01	WRKY
ZmWRKY43	AC198725.4_FG009	AC198725.4_FGP009	WRKY
ZmWRKY44	GRMZM2G105140	GRMZM2G105140_P01	WRKY
ZmWRKY45	GRMZM2G354384	GRMZM2G354384_P01	WRKY
ZmWRKY46	GRMZM2G054125	GRMZM2G054125_P01	WRKY
ZmWRKY47	GRMZM2G549512	GRMZM2G549512_P01	WRKY
ZmWRKY48	GRMZM2G148561	GRMZM2G148561_P01	WRKY
ZmWRKY49	AC205562.3_FG002	AC205562.3_FGP002	WRKY
ZmWRKY50	GRMZM2G377217	GRMZM2G377217_P01	WRKY
ZmWRKY51	GRMZM2G138683	GRMZM2G138683_P01	WRKY
ZmWRKY52	GRMZM2G063216	GRMZM2G063216_P01	WRKY
ZmWRKY53	GRMZM2G451035	GRMZM2G451035_P01	WRKY
ZmWRKY54	GRMZM2G461648	GRMZM2G461648_P01	WRKY
ZmWRKY55	GRMZM2G169564	GRMZM2G169564_P01	WRKY
ZmWRKY56	GRMZM2G027972	GRMZM2G027972_P01	WRKY
ZmWRKY57	GRMZM2G038158	GRMZM2G038158_P01	WRKY
ZmWRKY58	GRMZM2G147880	GRMZM2G147880_P01	WRKY
ZmWRKY59	GRMZM2G161411	GRMZM2G161411_P01	WRKY
ZmWRKY60	GRMZM5G880069	GRMZM5G880069_P02	WRKY
ZmWRKY61	GRMZM2G120320	GRMZM2G120320_P01	WRKY
ZmWRKY62	GRMZM2G048450	GRMZM2G048450_P01	WRKY
ZmWRKY63	GRMZM2G073272	GRMZM2G073272_P01	WRKY
ZmWRKY64	GRMZM5G823157	GRMZM5G823157_P01	WRKY
ZmWRKY65	GRMZM2G143765	GRMZM2G143765_P01	WRKY
ZmWRKY66	AC209050.3_FG003	AC209050.3_FGP003	WRKY
ZmWRKY67	GRMZM2G156529	GRMZM2G156529_P01	WRKY

ZmWRKY68.1	GRMZM2G163054	GRMZM2G163054_P04	WRKY
ZmWRKY68.2	GRMZM2G163054	GRMZM2G163054_P02	WRKY
ZmWRKY69	GRMZM2G127064	GRMZM2G127064_P01	WRKY
ZmWRKY70.1	GRMZM2G012724	GRMZM2G012724_P01	WRKY
ZmWRKY70.2	GRMZM2G012724	GRMZM2G012724_P03	WRKY
ZmWRKY70.3	GRMZM2G012724	GRMZM2G012724_P04	WRKY
ZmWRKY71	GRMZM2G169966	GRMZM2G169966_P01	WRKY
ZmWRKY72	GRMZM2G401521	GRMZM2G401521_P01	WRKY
ZmWRKY73	GRMZM5G863420	GRMZM5G863420_P01	WRKY
ZmWRKY74	GRMZM2G453571	GRMZM2G453571_P01	WRKY
ZmWRKY75.1	GRMZM2G366795	GRMZM2G366795_P01	WRKY
ZmWRKY75.2	GRMZM2G366795	GRMZM2G366795_P02	WRKY
ZmWRKY76	GRMZM2G018721	GRMZM2G018721_P01	WRKY
ZmWRKY77	GRMZM2G139815	GRMZM2G139815_P01	WRKY
ZmWRKY78	GRMZM2G025895	GRMZM2G025895_P01	WRKY
ZmWRKY79.1	GRMZM2G125653	GRMZM2G125653_P01	WRKY
ZmWRKY79.2	GRMZM2G125653	GRMZM2G125653_P02	WRKY
ZmWRKY80	GRMZM2G169149	GRMZM2G169149_P01	WRKY
ZmWRKY81.1	GRMZM2G151407	GRMZM2G151407_P01	WRKY
ZmWRKY81.2	GRMZM2G151407	GRMZM2G151407_P02	WRKY
ZmWRKY82.1	GRMZM2G398506	GRMZM2G398506_P01	WRKY
ZmWRKY82.2	GRMZM2G398506	GRMZM2G398506_P02	WRKY
ZmWRKY83	GRMZM2G381378	GRMZM2G381378_P01	WRKY
ZmWRKY84	GRMZM2G006497	GRMZM2G006497_P01	WRKY
ZmWRKY85	GRMZM2G516301	GRMZM2G516301_P01	WRKY
ZmWRKY86	GRMZM2G083350	GRMZM2G083350_P01	WRKY
ZmWRKY87	GRMZM2G448605	GRMZM2G448605_P01	WRKY
ZmWRKY88	GRMZM2G063880	GRMZM2G063880_P01	WRKY
ZmWRKY89	GRMZM2G414315	GRMZM2G414315_P01	WRKY
ZmWRKY90	GRMZM2G057116	GRMZM2G057116_P01	WRKY
ZmWRKY91	GRMZM2G061408	GRMZM2G061408_P01	WRKY
ZmWRKY92.1	GRMZM2G449681	GRMZM2G449681_P01	WRKY
ZmWRKY92.2	GRMZM2G449681	GRMZM2G449681_P02	WRKY
ZmWRKY93	GRMZM2G034421	GRMZM2G034421_P01	WRKY
ZmWRKY94	GRMZM2G432583	GRMZM2G432583_P01	WRKY
ZmWRKY95	GRMZM2G015433	GRMZM2G015433_P01	WRKY
ZmWRKY96	GRMZM2G137802	GRMZM2G137802_P01	WRKY
ZmWRKY97	GRMZM2G304573	GRMZM2G304573_P01	WRKY
ZmWRKY98	GRMZM2G149683	GRMZM2G149683_P01	WRKY
ZmWRKY99	GRMZM2G145554	GRMZM2G145554_P01	WRKY
ZmWRKY100	GRMZM2G411766	GRMZM2G411766_P01	WRKY
ZmWRKY101	GRMZM5G812272	GRMZM5G812272_P02	WRKY
ZmWRKY102	GRMZM2G045560	GRMZM2G045560_P01	WRKY

ZmWRKY103	GRMZM2G036703	GRMZM2G036703_P01	WRKY
ZmWRKY104	GRMZM2G029282	GRMZM2G029282_P01	WRKY
ZmWRKY105	GRMZM2G029292	GRMZM2G029292_P03	WRKY
ZmWRKY106	GRMZM2G013391	GRMZM2G013391_P01	WRKY
ZmWRKY107	GRMZM2G111354	GRMZM2G111354_P01	WRKY
ZmWRKY108	GRMZM2G003551	GRMZM2G003551_P01	WRKY
ZmWRKY109	GRMZM2G111711	GRMZM2G111711_P01	WRKY
ZmWRKY110.1	GRMZM2G171428	GRMZM2G171428_P01	WRKY
ZmWRKY110.2	GRMZM2G171428	GRMZM2G171428_P02	WRKY
ZmWRKY111	GRMZM2G060918	GRMZM2G060918_P01	WRKY
ZmWRKY112	GRMZM2G005207	GRMZM2G005207_P01	WRKY
ZmWRKY113	GRMZM2G441031	GRMZM2G441031_P01	WRKY
ZmWRKY114	GRMZM2G090594	GRMZM2G090594_P01	WRKY
ZmWRKY115	GRMZM2G004060	GRMZM2G004060_P01	WRKY
ZmWRKY116.1	GRMZM2G020254	GRMZM2G020254_P01	WRKY
ZmWRKY116.2	GRMZM2G020254	GRMZM2G020254_P02	WRKY
ZmWRKY116.3	GRMZM2G020254	GRMZM2G020254_P03	WRKY
ZmWRKY117	GRMZM2G031963	GRMZM2G031963_P01	WRKY
ZmWRKY118	AC208110.2_FG001	AC208110.2_FGP001	WRKY
ZmbZIP1	GRMZM2G428184	GRMZM2G428184_P01	bZIP
ZmbZIP2	GRMZM2G157177	GRMZM2G157177_P01	bZIP
ZmbZIP3	GRMZM2G038015	GRMZM2G038015_P01	bZIP
ZmbZIP4	GRMZM2G174284	GRMZM2G174284_P01	bZIP
ZmbZIP5	GRMZM2G062391	GRMZM2G062391_P01	bZIP
ZmbZIP6	GRMZM2G353553	GRMZM2G353553_P01	bZIP
ZmbZIP7	GRMZM2G093020	GRMZM2G093020_P02	bZIP
ZmbZIP8	GRMZM2G479885	GRMZM2G479885_P01	bZIP
ZmbZIP9.1	GRMZM2G073427	GRMZM2G073427_P04	bZIP
ZmbZIP9.2	GRMZM2G073427	GRMZM2G073427_P02	bZIP
ZmbZIP9.3	GRMZM2G073427	GRMZM2G073427_P03	bZIP
ZmbZIP9.4	GRMZM2G073427	GRMZM2G073427_P07	bZIP
ZmbZIP10	GRMZM2G370026	GRMZM2G370026_P01	bZIP
ZmbZIP11	AC186606.4_FG003	AC186606.4_FGP003	bZIP
ZmbZIP12	GRMZM2G332294	GRMZM2G332294_P01	bZIP
ZmbZIP13	GRMZM2G175280	GRMZM2G175280_P01	bZIP
ZmbZIP14	GRMZM2G478417	GRMZM2G478417_P01	bZIP
ZmbZIP15	GRMZM2G177046	GRMZM2G177046_P01	bZIP
ZmbZIP16.1	GRMZM2G019446	GRMZM2G019446_P02	bZIP
ZmbZIP16.2	GRMZM2G019446	GRMZM2G019446_P01	bZIP
ZmbZIP17	GRMZM2G016150	GRMZM2G016150_P01	bZIP
ZmbZIP18.1	GRMZM2G131961	GRMZM2G131961_P01	bZIP
ZmbZIP18.2	GRMZM2G131961	GRMZM2G131961_P03	bZIP
ZmbZIP18.3	GRMZM2G131961	GRMZM2G131961_P05	bZIP

ZmbZIP19.1	GRMZM2G112483	GRMZM2G112483_P01	bZIP
ZmbZIP19.2	GRMZM2G112483	GRMZM2G112483_P02	bZIP
ZmbZIP20.1	GRMZM5G821024	GRMZM5G821024_P01	bZIP
ZmbZIP20.2	GRMZM5G821024	GRMZM5G821024_P02	bZIP
ZmbZIP21.1	GRMZM2G171370	GRMZM2G171370_P03	bZIP
ZmbZIP21.2	GRMZM2G171370	GRMZM2G171370_P01	bZIP
ZmbZIP22	AC203957.3_FG004	AC203957.3_FGP004	bZIP
ZmbZIP23	GRMZM2G002075	GRMZM2G002075_P01	bZIP
ZmbZIP24	GRMZM2G137532	GRMZM2G137532_P01	bZIP
ZmbZIP25	GRMZM2G125934	GRMZM2G125934_P01	bZIP
ZmbZIP26	GRMZM2G180847	GRMZM2G180847_P01	bZIP
ZmbZIP27	GRMZM2G037910	GRMZM2G037910_P01	bZIP
ZmbZIP28	GRMZM2G060109	GRMZM2G060109_P01	bZIP
ZmbZIP29	GRMZM2G166566	GRMZM2G166566_P01	bZIP
ZmbZIP30	AC233853.1_FG002	AC233853.1_FGP002	bZIP
ZmbZIP31	GRMZM2G039828	GRMZM2G039828_P01	bZIP
ZmbZIP32	GRMZM2G160136	GRMZM2G160136_P02	bZIP
ZmbZIP33	GRMZM2G074373	GRMZM2G074373_P01	bZIP
ZmbZIP34	GRMZM2G160902	GRMZM2G160902_P01	bZIP
ZmbZIP35	GRMZM2G024851	GRMZM2G024851_P01	bZIP
ZmbZIP36.1	GRMZM2G146020	GRMZM2G146020_P01	bZIP
ZmbZIP36.2	GRMZM2G146020	GRMZM2G146020_P02	bZIP
ZmbZIP37.1	GRMZM5G858197	GRMZM5G858197_P02	bZIP
ZmbZIP37.2	GRMZM5G858197	GRMZM5G858197_P03	bZIP
ZmbZIP38.1	GRMZM2G060216	GRMZM2G060216_P01	bZIP
ZmbZIP38.2	GRMZM2G060216	GRMZM2G060216_P02	bZIP
ZmbZIP39	GRMZM2G159134	GRMZM2G159134_P02	bZIP
ZmbZIP40.1	GRMZM2G019907	GRMZM2G019907_P02	bZIP
ZmbZIP40.2	GRMZM2G019907	GRMZM2G019907_P03	bZIP
ZmbZIP41	GRMZM2G027976	GRMZM2G027976_P01	bZIP
ZmbZIP42	GRMZM5G848942	GRMZM5G848942_P01	bZIP
ZmbZIP43	GRMZM2G117851	GRMZM2G117851_P01	bZIP
ZmbZIP44	GRMZM2G368491	GRMZM2G368491_P01	bZIP
ZmbZIP45	GRMZM2G120167	GRMZM2G120167_P01	bZIP
ZmbZIP46.1	GRMZM2G157722	GRMZM2G157722_P02	bZIP
ZmbZIP46.2	GRMZM2G157722	GRMZM2G157722_P01	bZIP
ZmbZIP47	GRMZM2G118870	GRMZM2G118870_P01	bZIP
ZmbZIP48	GRMZM2G380897	GRMZM2G380897_P01	bZIP
ZmbZIP49	GRMZM2G122846	GRMZM2G122846_P01	bZIP
ZmbZIP50	GRMZM2G030877	GRMZM2G030877_P01	bZIP
ZmbZIP51	GRMZM2G011119	GRMZM2G011119_P01	bZIP
ZmbZIP52	AC190609.3_FG001	AC190609.3_FGP001	bZIP
ZmbZIP53	GRMZM2G149150	GRMZM2G149150_P01	bZIP

ZmbZIP54	GRMZM2G144480	GRMZM2G144480_P01	bZIP
ZmbZIP55	GRMZM2G079365	GRMZM2G079365_P01	bZIP
ZmbZIP56	GRMZM2G052102	GRMZM2G052102_P01	bZIP
ZmbZIP57	GRMZM2G336766	GRMZM2G336766_P01	bZIP
ZmbZIP58	GRMZM2G019106	GRMZM2G019106_P01	bZIP
ZmbZIP59	GRMZM2G361611	GRMZM2G361611_P01	bZIP
ZmbZIP60.1	GRMZM2G007063	GRMZM2G007063_P01	bZIP
ZmbZIP60.2	GRMZM2G007063	GRMZM2G007063_P04	bZIP
ZmbZIP61	GRMZM2G386273	GRMZM2G386273_P01	bZIP
ZmbZIP62.1	GRMZM2G000171	GRMZM2G000171_P02	bZIP
ZmbZIP62.2	GRMZM2G000171	GRMZM2G000171_P01	bZIP
ZmbZIP63	GRMZM2G153144	GRMZM2G153144_P01	bZIP
ZmbZIP64	GRMZM2G444748	GRMZM2G444748_P01	bZIP
ZmbZIP65.1	GRMZM2G098904	GRMZM2G098904_P01	bZIP
ZmbZIP65.2	GRMZM2G098904	GRMZM2G098904_P02	bZIP
ZmbZIP66	GRMZM2G080111	GRMZM2G080111_P01	bZIP
ZmbZIP67	GRMZM2G137046	GRMZM2G137046_P01	bZIP
ZmbZIP68	GRMZM2G029979	GRMZM2G029979_P01	bZIP
ZmbZIP69	GRMZM2G158313	GRMZM2G158313_P01	bZIP
ZmbZIP70	GRMZM2G092609	GRMZM2G092609_P01	bZIP
ZmbZIP71	GRMZM2G111504	GRMZM2G111504_P01	bZIP
ZmbZIP72	GRMZM2G020799	GRMZM2G020799_P01	bZIP
ZmbZIP73	GRMZM2G073892	GRMZM2G073892_P01	bZIP
ZmbZIP74	GRMZM2G448607	GRMZM2G448607_P01	bZIP
ZmbZIP75	GRMZM2G402862	GRMZM2G402862_P01	bZIP
ZmbZIP76	GRMZM2G055413	GRMZM2G055413_P01	bZIP
ZmbZIP77.1	GRMZM2G133331	GRMZM2G133331_P01	bZIP
ZmbZIP77.2	GRMZM2G133331	GRMZM2G133331_P02	bZIP
ZmbZIP77.3	GRMZM2G133331	GRMZM2G133331_P03	bZIP
ZmbZIP78	GRMZM2G396632	GRMZM2G396632_P01	bZIP
ZmbZIP79.1	GRMZM2G045236	GRMZM2G045236_P01	bZIP
ZmbZIP79.2	GRMZM2G045236	GRMZM2G045236_P02	bZIP
ZmbZIP80.1	GRMZM2G000842	GRMZM2G000842_P01	bZIP
ZmbZIP80.2	GRMZM2G000842	GRMZM2G000842_P02	bZIP
ZmbZIP80.3	GRMZM2G000842	GRMZM2G000842_P03	bZIP
ZmbZIP81.1	GRMZM2G129247	GRMZM2G129247_P02	bZIP
ZmbZIP81.2	GRMZM2G129247	GRMZM2G129247_P03	bZIP
ZmbZIP82	GRMZM2G175870	GRMZM2G175870_P01	bZIP
ZmbZIP83.1	GRMZM2G011932	GRMZM2G011932_P01	bZIP
ZmbZIP83.2	GRMZM2G011932	GRMZM2G011932_P04	bZIP
ZmbZIP84	GRMZM2G438652	GRMZM2G438652_P01	bZIP
ZmbZIP85.1	GRMZM2G015534	GRMZM2G015534_P02	bZIP
ZmbZIP85.2	GRMZM2G015534	GRMZM2G015534_P01	bZIP

ZmbZIP85.3	GRMZM2G015534	GRMZM2G015534_P03	bZIP
ZmbZIP86.1	GRMZM2G095078	GRMZM2G095078_P01	bZIP
ZmbZIP86.2	GRMZM2G095078	GRMZM2G095078_P02	bZIP
ZmbZIP87	GRMZM2G092137	GRMZM2G092137_P01	bZIP
ZmbZIP88	GRMZM2G358796	GRMZM2G358796_P01	bZIP
ZmbZIP89.1	GRMZM2G006578	GRMZM2G006578_P01	bZIP
ZmbZIP89.2	GRMZM2G006578	GRMZM2G006578_P02	bZIP
ZmbZIP89.3	GRMZM2G006578	GRMZM2G006578_P03	bZIP
ZmbZIP90	AC200057.4_FG007	AC200057.4_FGP007	bZIP
ZmbZIP91	GRMZM2G043600	GRMZM2G043600_P01	bZIP
ZmbZIP92	GRMZM2G149040	GRMZM2G149040_P01	bZIP
ZmbZIP93.1	GRMZM2G361847	GRMZM2G361847_P04	bZIP
ZmbZIP93.2	GRMZM2G361847	GRMZM2G361847_P01	bZIP
ZmbZIP93.3	GRMZM2G361847	GRMZM2G361847_P02	bZIP
ZmbZIP93.4	GRMZM2G361847	GRMZM2G361847_P07	bZIP
ZmbZIP94	GRMZM2G077124	GRMZM2G077124_P03	bZIP
ZmbZIP95	GRMZM2G067921	GRMZM2G067921_P01	bZIP
ZmbZIP96.1	GRMZM2G030280	GRMZM2G030280_P01	bZIP
ZmbZIP96.2	GRMZM2G030280	GRMZM2G030280_P02	bZIP
ZmbZIP97.1	GRMZM2G171912	GRMZM2G171912_P01	bZIP
ZmbZIP97.2	GRMZM2G171912	GRMZM2G171912_P02	bZIP
ZmbZIP98	GRMZM2G132868	GRMZM2G132868_P01	bZIP
ZmbZIP99	GRMZM2G161009	GRMZM2G161009_P01	bZIP
ZmbZIP100.1	GRMZM2G080731	GRMZM2G080731_P01	bZIP
ZmbZIP100.2	GRMZM2G080731	GRMZM2G080731_P02	bZIP
ZmbZIP100.3	GRMZM2G080731	GRMZM2G080731_P03	bZIP
ZmbZIP101.1	GRMZM2G033230	GRMZM2G033230_P01	bZIP
ZmbZIP101.2	GRMZM2G033230	GRMZM2G033230_P02	bZIP
ZmbZIP102	GRMZM2G438293	GRMZM2G438293_P01	bZIP
ZmbZIP103	AC232238.2_FG004	AC232238.2_FGP004	bZIP
ZmbZIP104	GRMZM2G168079	GRMZM2G168079_P01	bZIP
ZmbZIP105	GRMZM2G125243	GRMZM2G125243_P01	bZIP
ZmbZIP106.1	GRMZM2G170079	GRMZM2G170079_P02	bZIP
ZmbZIP106.2	GRMZM2G170079	GRMZM2G170079_P01	bZIP
ZmbZIP107	GRMZM2G033413	GRMZM2G033413_P01	bZIP
ZmbZIP108	GRMZM2G140355	GRMZM2G140355_P01	bZIP
ZmbZIP109	GRMZM2G407631	GRMZM2G407631_P01	bZIP
ZmbZIP110.1	GRMZM2G025812	GRMZM2G025812_P01	bZIP
ZmbZIP110.2	GRMZM2G025812	GRMZM2G025812_P02	bZIP
ZmbZIP111	GRMZM2G060290	GRMZM2G060290_P01	bZIP
ZmbZIP112.1	GRMZM2G103647	GRMZM2G103647_P03	bZIP
ZmbZIP112.2	GRMZM2G103647	GRMZM2G103647_P01	bZIP
ZmbZIP113	GRMZM2G151295	GRMZM2G151295_P02	bZIP

ZmbZIP114.1	GRMZM2G056099	GRMZM2G056099_P01	bZIP
ZmbZIP114.2	GRMZM2G056099	GRMZM2G056099_P02	bZIP
ZmbZIP115	GRMZM2G066734	GRMZM2G066734_P01	bZIP
ZmbZIP116	GRMZM2G473274	GRMZM2G473274_P01	bZIP
ZmbZIP117	GRMZM2G366264	GRMZM2G366264_P01	bZIP
ZmbZIP118	GRMZM2G365754	GRMZM2G365754_P01	bZIP
ZmbZIP119.1	GRMZM2G136266	GRMZM2G136266_P01	bZIP
ZmbZIP119.2	GRMZM2G136266	GRMZM2G136266_P02	bZIP
ZmbZIP120	GRMZM2G088140	GRMZM2G088140_P02	bZIP
ZmbZIP121	GRMZM2G094352	GRMZM2G094352_P01	bZIP
ZmbZIP122	GRMZM5G884349	GRMZM5G884349_P01	bZIP
ZmbZIP123.1	GRMZM2G425920	GRMZM2G425920_P01	bZIP
ZmbZIP123.2	GRMZM2G425920	GRMZM2G425920_P02	bZIP
ZmbZIP124	GRMZM2G358701	GRMZM2G358701_P01	bZIP
ZmbZIP125.1	GRMZM2G445575	GRMZM2G445575_P01	bZIP
ZmbZIP125.2	GRMZM2G445575	GRMZM2G445575_P02	bZIP
ZmbZIP125.3	GRMZM2G445575	GRMZM2G445575_P03	bZIP
ZmMYB001	AC217264.3_FG005	AC217264.3_FGP005	MYB
ZmMYB002	GRMZM2G024468	GRMZM2G024468_P01	MYB
ZmMYB003	GRMZM2G037650	GRMZM2G037650_P01	MYB
ZmMYB004	GRMZM2G046443	GRMZM2G046443_P01	MYB
ZmMYB005	GRMZM2G054111	GRMZM2G054111_P01	MYB
ZmMYB006	GRMZM2G057027	GRMZM2G057027_P02	MYB
ZmMYB007	GRMZM2G070849	GRMZM2G070849_P01	MYB
ZmMYB008	GRMZM2G077147	GRMZM2G077147_P01	MYB
ZmMYB009	GRMZM2G079123	GRMZM2G079123_P01	MYB
ZmMYB010	GRMZM2G084583	GRMZM2G084583_P01	MYB
ZmMYB011	GRMZM2G084799	GRMZM2G084799_P01	MYB
ZmMYB012	GRMZM2G106558	GRMZM2G106558_P02	MYB
ZmMYB013	GRMZM2G110135	GRMZM2G110135_P01	MYB
ZmMYB014	GRMZM2G121570	GRMZM2G121570_P01	MYB
ZmMYB015	GRMZM2G130149	GRMZM2G130149_P01	MYB
ZmMYB016	GRMZM2G131937	GRMZM2G131937_P01	MYB
ZmMYB017	GRMZM2G143046	GRMZM2G143046_P01	MYB
ZmMYB018	GRMZM2G147346	GRMZM2G147346_P01	MYB
ZmMYB019	GRMZM2G147698	GRMZM2G147698_P01	MYB
ZmMYB020	GRMZM2G308034	GRMZM2G308034_P01	MYB
ZmMYB021	GRMZM2G403620	GRMZM2G403620_P01	MYB
ZmMYB022	GRMZM2G428555	GRMZM2G428555_P01	MYB
ZmMYB023	GRMZM5G870592	GRMZM5G870592_P01	MYB
ZmMYB024	AC165178.2_FG004	AC165178.2_FGP004	MYB
ZmMYB025	GRMZM2G032655	GRMZM2G032655_P01	MYB
ZmMYB026	GRMZM2G038722	GRMZM2G038722_P01	MYB

ZmMYB027	GRMZM2G048295	GRMZM2G048295_P01	MYB
ZmMYB028	GRMZM2G050305	GRMZM2G050305_P01	MYB
ZmMYB029	GRMZM2G064630	GRMZM2G064630_P01	MYB
ZmMYB030	GRMZM2G087955	GRMZM2G087955_P01	MYB
ZmMYB031	GRMZM2G090837	GRMZM2G090837_P01	MYB
ZmMYB032	GRMZM2G105137	GRMZM2G105137_P01	MYB
ZmMYB033	GRMZM2G115859	GRMZM2G115859_P01	MYB
ZmMYB034	GRMZM2G123202	GRMZM2G123202_P01	MYB
ZmMYB035	GRMZM2G124715	GRMZM2G124715_P01	MYB
ZmMYB036	GRMZM2G139284	GRMZM2G139284_P01	MYB
ZmMYB037	GRMZM2G166337	GRMZM2G166337_P01	MYB
ZmMYB038	GRMZM2G176327	GRMZM2G176327_P01	MYB
ZmMYB039	GRMZM2G001875	GRMZM2G001875_P01	MYB
ZmMYB040	GRMZM2G017520	GRMZM2G017520_P01	MYB
ZmMYB041	GRMZM2G041415	GRMZM2G041415_P01	MYB
ZmMYB042	GRMZM2G047626	GRMZM2G047626_P01	MYB
ZmMYB043	GRMZM2G051256	GRMZM2G051256_P01	MYB
ZmMYB044	GRMZM2G052377	GRMZM2G052377_P01	MYB
ZmMYB045	GRMZM2G064744	GRMZM2G064744_P01	MYB
ZmMYB046	GRMZM2G083239	GRMZM2G083239_P01	MYB
ZmMYB047	GRMZM2G088783	GRMZM2G088783_P01	MYB
ZmMYB048	GRMZM2G111731	GRMZM2G111731_P01	MYB
ZmMYB049	GRMZM2G139688	GRMZM2G139688_P01	MYB
ZmMYB050	GRMZM2G143328	GRMZM2G143328_P01	MYB
ZmMYB051	GRMZM2G158700	GRMZM2G158700_P01	MYB
ZmMYB052	GRMZM2G160838	GRMZM2G160838_P01	MYB
ZmMYB053	GRMZM2G160840	GRMZM2G160840_P01	MYB
ZmMYB054	GRMZM2G162709	GRMZM2G162709_P01	MYB
ZmMYB055	GRMZM2G167829	GRMZM2G167829_P01	MYB
ZmMYB056	GRMZM2G369799	GRMZM2G369799_P01	MYB
ZmMYB057	GRMZM2G460869	GRMZM2G460869_P01	MYB
ZmMYB058	GRMZM2G470307	GRMZM2G470307_P01	MYB
ZmMYB059	GRMZM5G803355	GRMZM5G803355_P01	MYB
ZmMYB060	GRMZM2G011422	GRMZM2G011422_P01	MYB
ZmMYB061	GRMZM2G015021	GRMZM2G015021_P01	MYB
ZmMYB062	GRMZM2G017268	GRMZM2G017268_P01	MYB
ZmMYB063	GRMZM2G043792	GRMZM2G043792_P01	MYB
ZmMYB064	GRMZM2G055158	GRMZM2G055158_P01	MYB
ZmMYB065	GRMZM2G089244	GRMZM2G089244_P01	MYB
ZmMYB066	GRMZM2G108959	GRMZM2G108959_P01	MYB
ZmMYB067	GRMZM2G111045	GRMZM2G111045_P01	MYB
ZmMYB068	GRMZM2G111117	GRMZM2G111117_P01	MYB
ZmMYB069	GRMZM2G127857	GRMZM2G127857_P01	MYB

ZmMYB070	GRMZM2G131442	GRMZM2G131442_P01	MYB
ZmMYB071	GRMZM2G138427	GRMZM2G138427_P01	MYB
ZmMYB072	GRMZM2G162434	GRMZM2G162434_P01	MYB
ZmMYB073	GRMZM2G419239	GRMZM2G419239_P01	MYB
ZmMYB074	GRMZM2G496770	GRMZM2G496770_P01	MYB
ZmMYB075	GRMZM5G833253	GRMZM5G833253_P01	MYB
ZmMYB076	GRMZM2G048136	GRMZM2G048136_P01	MYB
ZmMYB077	GRMZM2G001223	GRMZM2G001223_P03	MYB
ZmMYB078	GRMZM2G027697	GRMZM2G027697_P01	MYB
ZmMYB079	GRMZM2G040924	GRMZM2G040924_P01	MYB
ZmMYB080	GRMZM2G070523	GRMZM2G070523_P01	MYB
ZmMYB081	GRMZM2G073836	GRMZM2G073836_P01	MYB
ZmMYB082	GRMZM2G088189	GRMZM2G088189_P01	MYB
ZmMYB083	GRMZM2G095904	GRMZM2G095904_P01	MYB
ZmMYB084	GRMZM2G104789	GRMZM2G104789_P01	MYB
ZmMYB085	GRMZM2G145444	GRMZM2G145444_P01	MYB
ZmMYB086	GRMZM2G159547	GRMZM2G159547_P01	MYB
ZmMYB087	GRMZM2G161512	GRMZM2G161512_P01	MYB
ZmMYB088	GRMZM2G170049	GRMZM2G170049_P01	MYB
ZmMYB089	GRMZM2G302549	GRMZM2G302549_P01	MYB
ZmMYB090	GRMZM2G455869	GRMZM2G455869_P01	MYB
ZmMYB091	GRMZM2G013581	GRMZM2G013581_P01	MYB
ZmMYB092	GRMZM2G048910	GRMZM2G048910_P01	MYB
ZmMYB093	GRMZM2G069325	GRMZM2G069325_P02	MYB
ZmMYB094	GRMZM2G077789	GRMZM2G077789_P01	MYB
ZmMYB095	GRMZM2G078820	GRMZM2G078820_P01	MYB
ZmMYB096	GRMZM2G093647	GRMZM2G093647_P01	MYB
ZmMYB097	GRMZM2G093660	GRMZM2G093660_P01	MYB
ZmMYB098	GRMZM2G093789	GRMZM2G093789_P01	MYB
ZmMYB099	GRMZM2G102790	GRMZM2G102790_P01	MYB
ZmMYB100	GRMZM2G175232	GRMZM2G175232_P01	MYB
ZmMYB101	GRMZM2G305856	GRMZM2G305856_P01	MYB
ZmMYB102	GRMZM2G343068	GRMZM2G343068_P01	MYB
ZmMYB103	GRMZM2G423833	GRMZM2G423833_P01	MYB
ZmMYB104	GRMZM2G701063	GRMZM2G701063_P01	MYB
ZmMYB105	AC213884.3_FG002	AC213884.3_FGP002	MYB
ZmMYB106	GRMZM2G000818	GRMZM2G000818_P01	MYB
ZmMYB107	GRMZM2G031323	GRMZM2G031323_P01	MYB
ZmMYB108	GRMZM2G045748	GRMZM2G045748_P01	MYB
ZmMYB109	GRMZM2G050550	GRMZM2G050550_P01	MYB
ZmMYB110	GRMZM2G056407	GRMZM2G056407_P01	MYB
ZmMYB111	GRMZM2G104551	GRMZM2G104551_P01	MYB
ZmMYB112	GRMZM2G117244	GRMZM2G117244_P01	MYB

ZmMYB113	GRMZM2G126566	GRMZM2G126566_P01	MYB
ZmMYB114	GRMZM2G150841	GRMZM2G150841_P01	MYB
ZmMYB115	GRMZM2G169356	GRMZM2G169356_P01	MYB
ZmMYB116	GRMZM2G172327	GRMZM2G172327_P01	MYB
ZmMYB117	GRMZM2G003406	GRMZM2G003406_P01	MYB
ZmMYB118	GRMZM2G006352	GRMZM2G006352_P01	MYB
ZmMYB119	GRMZM2G028054	GRMZM2G028054_P01	MYB
ZmMYB120	GRMZM2G047600	GRMZM2G047600_P01	MYB
ZmMYB121	GRMZM2G051528	GRMZM2G051528_P01	MYB
ZmMYB122	GRMZM2G096358	GRMZM2G096358_P01	MYB
ZmMYB123	GRMZM2G119693	GRMZM2G119693_P01	MYB
ZmMYB124	GRMZM2G151205	GRMZM2G151205_P01	MYB
ZmMYB125	GRMZM2G169316	GRMZM2G169316_P01	MYB
ZmMYB126	GRMZM2G171781	GRMZM2G171781_P01	MYB
ZmMYB127	GRMZM2G312419	GRMZM2G312419_P01	MYB
ZmMYB128	GRMZM2G322490	GRMZM2G322490_P01	MYB
ZmMYB129	GRMZM2G330475	GRMZM2G330475_P01	MYB
ZmMYB130	GRMZM2G395672	GRMZM2G395672_P01	MYB
ZmMYB131	GRMZM2G405094	GRMZM2G405094_P01	MYB
ZmMYB132	GRMZM2G431156	GRMZM2G431156_P01	MYB
ZmMYB133	GRMZM2G004090	GRMZM2G004090_P01	MYB
ZmMYB134	GRMZM2G005066	GRMZM2G005066_P01	MYB
ZmMYB135	GRMZM2G022686	GRMZM2G022686_P01	MYB
ZmMYB136	GRMZM2G044824	GRMZM2G044824_P01	MYB
ZmMYB137	GRMZM2G089686	GRMZM2G089686_P01	MYB
ZmMYB138	GRMZM2G098179	GRMZM2G098179_P01	MYB
ZmMYB139	GRMZM2G134279	GRMZM2G134279_P01	MYB
ZmMYB140	GRMZM2G167088	GRMZM2G167088_P01	MYB
ZmMYB141	GRMZM2G416652	GRMZM2G416652_P01	MYB
ZmMYB142	GRMZM5G803308	GRMZM5G803308_P01	MYB
ZmMYB143	AC197146.3_FG002	AC197146.3_FGP002	MYB
ZmMYB144	AC206901.3_FG005	AC206901.3_FGP005	MYB
ZmMYB145	GRMZM2G001824	GRMZM2G001824_P01	MYB
ZmMYB146	GRMZM2G052606	GRMZM2G052606_P01	MYB
ZmMYB147	GRMZM2G081557	GRMZM2G081557_P01	MYB
ZmMYB148	GRMZM2G097636	GRMZM2G097636_P01	MYB
ZmMYB149	GRMZM2G097638	GRMZM2G097638_P01	MYB
ZmMYB150	GRMZM2G127490	GRMZM2G127490_P01	MYB
ZmMYB151	GRMZM2G150680	GRMZM2G150680_P01	MYB
ZmMYB152	GRMZM2G172487	GRMZM2G172487_P01	MYB
ZmMYB153	GRMZM2G172575	GRMZM2G172575_P01	MYB
ZmMYB154	GRMZM2G173633	GRMZM2G173633_P01	MYB
ZmMYB155	GRMZM2G311059	GRMZM2G311059_P01	MYB

ZmMYB156	GRMZM2G325907	GRMZM2G325907_P01	MYB
ZmMYB157	GRMZM2G425427	GRMZM2G425427_P01	MYB
ZmMYC1	GRMZM2G057413	GRMZM2G057413_P01	MYC
ZmMYC2	AC193786.3_FG005	AC193786.3_FGP005	MYC
ZmMYC3	GRMZM2G089501	GRMZM2G089501_P01	MYC
ZmMYC4	GRMZM2G009478	GRMZM2G009478_P05	MYC
ZmMYC5	GRMZM2G317450	GRMZM2G317450_P03	MYC
ZmMYC6	GRMZM2G114444	GRMZM2G114444_P02	MYC
ZmMYC7	GRMZM2G001930	GRMZM2G001930_P01	MYC
ZmMYC8	GRMZM2G049229	GRMZM2G049229_P01	MYC
