# Supplementary material for: Maize protein phosphatase gene family: identification and molecular characterization
Source: BMC Genomics. 2014 Sep 9;15(1):773. doi: 10.1186/1471-2164-15-773 (PMC4169795; doi:10.1186/1471-2164-15-773)
Supplement: Supplementary file 21 — Additional file 21: Table S11: List of expression values of ZmPP genes under cold stress. ETH-DH7 log(c), ETH-DH7 log(k), ETH-DL3 log(c), ETH-DL3 log(k) means intensity (AU) of fluorescence of labeled aaRNA from cold-treatment (c) or control (k) hybridizing to the probe, shown as log2 in the respective maize line. DH7 log(c/k), DL3 log(c/k) means log2 of ratio of expression in cold-treated vs. control of the probe in the respective maize line, respectively. Values in red and blue indicate the fold increase and decrease in expression in the drought-stressed tissue, respectively. (PDF 121 KB) [file 12864_2014_6458_MOESM21_ESM.pdf]

Table S11. List of expression values of ZmPP genes under cold stress.

DH7 log(c), DH7 log(k), DL3 log(c), DL3 log(k) means intensity (AU) of fluorescence of labeled aaRNA from cold-treatment (c) or control (k) hybridizing to the probe, shown as log2 in the respective maize line.

DH7 log(c/k), DL3 log(c/k) means log2 of ratio of expression in cold-treated vs. control of the probe in the respective maize line,respectively.

Values in red and blue indicate the fold increase and decrease in expression in the drought-stressed tissue, respectively.

Name	DH7 log(c)	DH7 log(k)	DL3 log(c)	DL3 log(c)	DH7 log(c/k)	DL3 log(c/k)
ZmPP92	12.2755891	8.598624721	12.21228293	8.837911579	3.676964381	3.374371
ZmPP112	11.54955362	8.265338552	11.1310569	8.489927601	3.284215069	2.641129
ZmPP6	10.07984605	7.28284839	9.424434785	7.617382441	2.796997663	1.807052
ZmPP127	12.35940949	10.18063729	12.21841043	10.1860328	2.178772196	2.032378
ZmPP154	9.896163431	7.723406847	8.610214666	7.710736723	2.172756584	0.899478
ZmPP77	6.980064984	4.886418438	6.725156172	5.201198739	2.093646546	1.523957
ZmPP66	10.84811506	8.835777353	11.23824522	8.847655697	2.012337709	2.39059
ZmPP155	9.383089494	7.527788394	9.409408919	8.47421486	1.8553011	0.935194
ZmPP149	10.41260733	8.619329697	10.41208824	8.590938065	1.793277635	1.82115
ZmPP116	11.15854185	9.466536325	10.61700151	9.420995312	1.692005524	1.196006
ZmPP82	13.07127009	11.41009969	12.93326538	11.29560818	1.661170398	1.637657
ZmPP24	11.08830144	9.499679419	10.80005566	9.320061817	1.588622017	1.479994
ZmPP29	11.90575413	10.40051102	11.93719061	10.2565259	1.505243108	1.680665
ZmPP27	13.26748158	11.80536894	13.38159698	11.72946917	1.462112638	1.652128
ZmPP80	12.522802	11.11657237	11.74050475	11.39852087	1.40622963	0.341984
ZmPP132	12.3186864	11.07213976	12.17359906	10.98792645	1.246546645	1.185673
ZmPP56	4.342196656	3.104836955	3.913025071	4.188681318	1.237359701	-0.27566
ZmPP28	12.28309221	11.0563846	11.66313249	10.98727403	1.226707608	0.675858
ZmPP76	11.27052205	10.0887268	11.55889605	10.25796511	1.181795251	1.300931
ZmPP159	12.6819751	11.52062234	12.62682101	11.79398156	1.161352757	0.832839
ZmPP156	10.99669878	9.936068793	10.89779218	9.758725256	1.060629984	1.139067
ZmPP135	11.12474438	10.14597803	11.34371983	10.6788961	0.978766349	0.664824
ZmPP139	5.334634983	4.362378575	4.701168463	4.76037798	0.972256408	-0.05921
ZmPP124	7.860842031	6.89499714	8.714069363	8.011643134	0.965844891	0.702426
ZmPP13	12.32920953	11.3838856	12.58838915	11.59916572	0.945323927	0.989223
ZmPP95	13.68410911	12.76682723	13.05638895	12.74074138	0.91728188	0.315648
ZmPP129	6.817685094	5.930875664	6.579063854	6.448932054	0.88680943	0.130132
ZmPP57	13.12064011	12.30173349	12.39563661	12.07020646	0.818906617	0.32543
ZmPP115	14.54815253	13.78120358	14.14932684	13.57728373	0.76694895	0.572043
ZmPP89	13.68136519	12.9145811	13.51241469	12.94277237	0.766784094	0.569642
ZmPP4	12.4979262	11.75525578	12.62497969	11.92012166	0.742670423	0.704858
ZmPP10	11.63720875	10.9081907	12.19914967	10.97235589	0.729018055	1.226794
ZmPP53	13.29529187	12.56913221	13.19052982	12.45982944	0.726159658	0.7307
ZmPP147	10.18551135	9.478730255	11.00175874	9.86031133	0.706781092	1.141447
ZmPP54	12.0073442	11.34572398	12.9665977	11.40058413	0.661620218	1.566014
ZmPP146	5.000527939	4.349701973	4.945741656	3.6245451	0.650825967	1.321197
ZmPP105	9.528059634	8.894928097	9.076896617	8.404963984	0.633131537	0.671933
ZmPP143	11.3862509	10.80026657	11.79229712	10.94269874	0.585984323	0.849598
ZmPP153	11.5830344	11.02959379	11.60089165	11.07489182	0.553440613	0.526
ZmPP144	11.23249265	10.70410717	10.98328808	11.10507877	0.528385478	-0.12179
ZmPP9	5.733209593	5.302130598	5.340739588	5.612986888	0.431078994	-0.27225
ZmPP145	14.85310581	14.43844464	14.73862919	14.63118704	0.414661168	0.107442
ZmPP98	12.49745449	12.10996056	12.47360192	12.24526636	0.387493928	0.228336
ZmPP128	11.95642001	11.60804673	11.54632935	11.59902057	0.348373281	-0.05269
ZmPP44	10.45270446	10.10558801	10.25026393	9.916670274	0.347116448	0.333594

ZmPP70	9.758673125	9.415697332	9.778183185	9.571448028	0.342975793	0.206735
ZmPP47	11.57857454	11.24535466	10.9884402	11.37116394	0.333219878	-0.38272
ZmPP90	10.33833083	10.02930996	10.57959313	10.08923108	0.309020866	0.490362
ZmPP85	12.4304873	12.15609027	12.22109413	12.53444497	0.274397038	-0.31335
ZmPP106	11.89520274	11.6354045	11.89309844	11.72286099	0.259798232	0.170237
ZmPP104	11.40940774	11.15006799	11.71256572	11.69860855	0.259339756	0.013957
ZmPP111	8.827907778	8.582086321	9.283204092	9.135676809	0.245821458	0.147527
ZmPP72	9.052378292	8.834129203	8.964701106	9.051506399	0.218249089	-0.08681
ZmPP41	8.795852779	8.592134847	7.869495671	7.622040037	0.203717932	0.247456
ZmPP84	14.53613877	14.38874864	14.75931945	14.29319314	0.147390133	0.466126
ZmPP121	8.803380791	8.679977983	8.754870179	8.374756048	0.123402808	0.380114
ZmPP26	12.59527027	12.48276331	12.45083416	12.62104893	0.112506965	-0.17021
ZmPP134	11.53810576	11.43359653	11.08074709	11.38406728	0.104509229	-0.30332
ZmPP65	7.843044935	7.754314124	7.75712142	7.802142855	0.088730811	-0.04502
ZmPP122	8.353554798	8.281980769	7.634827196	8.440722238	0.071574028	-0.8059
ZmPP102	10.02733753	10.00133547	10.04603802	10.24541398	0.02600206	-0.19938
ZmPP52	10.63141674	10.61815302	11.40187819	11.59974371	0.013263724	-0.19787
ZmPP69	5.077133624	7.282318234	5.538140102	7.372384707	-2.20518461	-1.83424
ZmPP101	12.49975932	14.2514791	12.88271088	13.79631736	-1.75171978	-0.91361
ZmPP87	9.573942564	11.17696113	9.71291012	10.98352452	-1.60301857	-1.27061
ZmPP118	7.932189158	9.294393076	9.39631727	10.58208216	-1.36220392	-1.18576
ZmPP137	10.51288511	11.77908807	10.38835156	11.42468527	-1.26620296	-1.03633
ZmPP33	9.849331705	10.90241773	10.15551994	11.19747396	-1.05308602	-1.04195
ZmPP36	10.5219866	11.56721042	10.46274943	11.93737022	-1.04522382	-1.47462
ZmPP58	14.51929284	15.55102517	14.63094204	15.25302329	-1.03173233	-0.62208
ZmPP16	11.6978626	12.69355374	10.93144032	12.06386002	-0.99569114	-1.13242
ZmPP67	8.574070758	9.53596328	8.272909077	9.161106887	-0.96189252	-0.8882
ZmPP11	5.919962293	6.790958995	6.351684829	7.30283055	-0.8709967	-0.95115
ZmPP18	6.21815879	7.059952344	7.525866108	6.928311092	-0.84179355	0.597555
ZmPP68	4.492661468	5.258593634	5.454760398	5.421476167	-0.76593217	0.033284
ZmPP34	10.21174518	10.91991307	9.473212728	10.32163902	-0.70816789	-0.84843
ZmPP131	11.65519905	12.33595773	11.55991226	12.28205263	-0.68075868	-0.72214
ZmPP126	7.102282082	7.753509574	7.340247912	7.017268766	-0.65122749	0.322979
ZmPP119	7.429439521	8.030547491	7.747943391	8.679577931	-0.60110797	-0.93163
ZmPP125	10.32511887	10.92122712	10.29805543	10.66911358	-0.59610825	-0.37106
ZmPP62	11.87225071	12.4635103	12.2331553	12.38908369	-0.59125958	-0.15593
ZmPP93	7.970683395	8.555648074	8.346805335	8.921558197	-0.58496468	-0.57475
ZmPP51	9.969956144	10.52048361	10.25367302	10.68825889	-0.55052747	-0.43459
ZmPP23	8.777454733	9.315626121	8.995376177	9.604484637	-0.53817139	-0.60911
ZmPP5	12.05197729	12.56189886	12.15279687	12.57742698	-0.50992157	-0.42463
ZmPP48	3.234968533	3.735295915	3.212038718	3.413069835	-0.50032738	-0.20103
ZmPP55	12.62268777	13.11872034	12.68521424	13.11742765	-0.49603257	-0.43221
ZmPP38	10.47790849	10.95955893	10.38913253	10.60857839	-0.48165044	-0.21945
ZmPP81	6.974378438	7.447969098	7.686191942	7.361093855	-0.47359066	0.325098
ZmPP64	6.007273698	6.475702227	6.705510087	6.308860655	-0.46842853	0.396649
ZmPP22	12.49339409	12.93528377	12.85284843	12.96069038	-0.44188968	-0.10784
ZmPP50	12.30368325	12.73889247	12.45554666	12.82942761	-0.43520922	-0.37388
ZmPP114	6.87038672	7.290692087	7.062932153	7.158375707	-0.42030537	-0.09544
ZmPP59	10.82573235	11.24038684	10.82375089	11.23008326	-0.41465448	-0.40633
ZmPP110	8.613977428	9.009360308	8.622403909	8.823970023	-0.39538288	-0.20157
ZmPP49	8.006241763	8.373195023	8.079665754	8.53356746	-0.36695326	-0.4539
ZmPP17	10.23882323	10.58563349	10.34373678	10.43103484	-0.34681026	-0.0873
ZmPP78	13.23025572	13.57067095	13.15054009	13.26598324	-0.34041523	-0.11544

ZmPP109	10.68394312	11.02351954	10.41844639	11.02427095	-0.33957642	-0.60582
ZmPP25	7.089615128	7.415725769	6.698597392	6.472788379	-0.32611064	0.225809
ZmPP157	4.129437666	4.455188119	4.744162066	4.732877149	-0.32575045	0.011285
ZmPP14	11.93304484	12.25252474	12.37330312	12.41089397	-0.3194799	-0.03759
ZmPP20	10.1836122	10.49355381	11.08250021	11.50827546	-0.30994161	-0.42578
ZmPP117	10.70992361	10.96436273	10.89236554	10.85740645	-0.25443912	0.034959
ZmPP71	9.332451457	9.579512643	9.635133031	9.35282287	-0.24706119	0.28231
ZmPP46	13.30265007	13.54318464	13.37996539	13.56451658	-0.24053457	-0.18455
ZmPP83	11.75396982	11.95401285	11.71917909	12.11983824	-0.20004303	-0.40066
ZmPP12	10.52157602	10.72137928	10.2901046	10.44639843	-0.19980326	-0.15629
ZmPP75	10.92287398	11.1117101	10.87859418	11.18825841	-0.18883612	-0.30966
ZmPP1	11.58843178	11.7663909	11.82654245	11.68191821	-0.17795913	0.144624
ZmPP140	13.5810591	13.70339399	13.75399734	13.79006624	-0.12233489	-0.03607
ZmPP100	11.49131071	11.56189806	11.75303982	11.66097809	-0.07058735	0.092062
ZmPP61	4.015074936	4.080932188	5.210206929	5.037459281	-0.06585725	0.172748
ZmPP45	10.72859672	10.78414145	10.69703114	10.74813235	-0.05554473	-0.0511
ZmPP150	14.07249713	14.11808268	14.1082653	14.25062156	-0.04558556	-0.14236
ZmPP136	11.84644124	11.89063653	11.88100167	11.92463723	-0.04419529	-0.04364
ZmPP130	4.459682464	4.468789664	4.402339837	5.267951368	-0.0091072	-0.86561
ZmPP15	6.075264989	6.0783458	5.757956162	5.438878048	-0.00308081	0.319078
