# Supplementary material for: Maize protein phosphatase gene family: identification and molecular characterization
Source: BMC Genomics. 2014 Sep 9;15(1):773. doi: 10.1186/1471-2164-15-773 (PMC4169795; doi:10.1186/1471-2164-15-773)
Supplement: Supplementary file 22 — Additional file 22: Table S12: List of expression values of MAPK cascades genes in cold stress. ETH-DH7 log(c), ETH-DH7 log(k), ETH-DL3 log(c), ETH-DL3 log(k) means intensity (AU) of fluorescence of labeled aaRNA from cold-treatment (c) or control (k) hybridizing to the probe, shown as log2 in the respective maize line. ETH-DH7 log(c/k), ETH-DL3 log(c/k) means log2 of ratio of expression in cold-treated vs. control of the probe in the respective maize line, respectively. (PDF 84 KB) [file 12864_2014_6458_MOESM22_ESM.pdf]

Table S12. List of expression values of MAPK cascades genes in cold stress.

DH7 log(c), DH7 log(k), DL3 log(c), DL3 log(k) means intensity (AU) of fluorescence of labeled aaRNA from cold-treatment (c) or control (k) hybridizing to the probe, shown as log2 in the respective maize line.

DH7 log(c/k), DL3 log(c/k) means log2 of ratio of expression in cold-treated vs. control of the probe in the respective maize line, respectively.

Values in red and blue indicate the fold increase and decrease in expression in the drought-stressed tissue, respectively.

Name	DH7 log(c)	DH7 log(k)	DL3 log(c)	DL3 log(c)	DH7 log(c/k)	DL3 log(c/k)
ZmMPK12	9.254116583	7.200538964	8.20086412	7.333360334	2.053577619	0.867503787
ZmMPK8	13.22432994	11.41630225	13.11966704	11.50011744	1.808027689	1.619549601
ZmMPK6	15.14855681	13.48505243	15.06134986	13.27489091	1.663504377	1.786458955
ZmMPK11	15.31213933	13.96328979	14.30758897	13.48709859	1.348849546	0.820490376
ZmMPK15	10.53304883	9.745627793	10.80796001	9.83340504	0.787421036	0.974554965
ZmSIMK1	13.13658422	12.6242119	12.65871102	12.7195762	0.512372319	-0.060865177
ZmMPK18	4.756354241	4.311253469	4.056122838	4.429762786	0.445100772	-0.373639948
ZmMPK7	6.683178896	6.468164744	6.976859148	6.648353878	0.215014152	0.328505269
ZmMPK17	5.192545189	4.99732629	6.322607684	7.289694171	0.195218898	-0.967086487
ZmMPK14	10.26658238	10.15206192	9.017493217	10.32444081	0.114520462	-1.306947597
ZmMPK5	12.85316178	12.75161444	13.07026569	12.91017476	0.101547339	0.160090937
ZmMPK13	12.36386923	13.24702517	11.47730413	12.7159848	-0.883155944	-1.238680673
ZmMPK19	8.487688796	9.030434262	7.564654052	8.248587891	-0.542745467	-0.683933839
ZmMPK9	14.21169279	14.21449407	13.94709853	14.42509794	-0.002801284	-0.477999414
ZmMKK4	9.777898743	8.127095532	10.07403	8.46065406	1.650803211	1.613375939
ZmMKK2	13.76736701	12.15013463	13.45736558	12.60051611	1.61723238	0.856849477
ZmMEK1	12.893139	12.2177944	12.98147008	12.53279652	0.675344602	0.448673556
ZmMKK3	12.1085362	11.94255781	12.24325035	11.67407897	0.165978392	0.569171386
ZmMAPKKK1	8.189048157	6.181811273	7.281574545	6.471011025	2.007236885	0.81056352
ZmMAPKKK17	12.41205315	10.45336959	12.6508795	10.4684761	1.958683554	2.182403405
ZmZIK9	9.735825897	7.832775883	10.12909004	7.733996513	1.903050014	2.395093527
ZmRaf43	8.914847884	7.364287245	9.49896839	7.143936482	1.550560639	2.355031908
ZmRaf17	9.893726452	8.510778546	9.948100239	9.324363095	1.382947906	0.623737145
ZmRaf24	5.117494528	3.817492973	5.399006486	4.741912681	1.300001555	0.657093805
ZmRaf21	14.71892051	13.5437152	14.56757306	13.49674125	1.17520531	1.070831813
ZmMAPKKK14	10.34847224	9.208019176	10.47601244	9.700474473	1.140453066	0.775537972
ZmRaf30	13.10940745	11.97485869	13.34146547	12.48621839	1.134548752	0.855247073
ZmMAPKKK15	8.532986403	7.405960399	8.407826647	7.769676551	1.127026004	0.638150096
ZmRaf36	12.93593327	11.86453755	13.27170465	12.11582454	1.071395725	1.155880108
ZmRaf33	11.43664635	10.6636381	11.55813477	10.74647079	0.773008251	0.811663983
ZmMAPKKK12	13.02316267	12.37400542	13.34745165	12.09574693	0.649157243	1.25170472
ZmRaf41	11.33842266	10.76511517	11.14448343	10.97057874	0.573307485	0.173904693
ZmMAPKKK20	13.00805577	12.45398296	13.39232267	12.63840526	0.55407281	0.753917401
ZmRaf10	11.56493173	11.08143532	11.10515527	10.87224434	0.48349641	0.232910932
ZmRaf34	12.76782754	12.28815883	12.98659957	12.45851406	0.479668705	0.528085508
ZmMAPKKK9	4.9261356	4.47370648	4.306421197	5.178268389	0.45242912	-0.871847192
ZmRaf46	5.685994702	5.294430364	5.461370454	5.486057814	0.391564338	-0.02468736
ZmRaf22	7.167631948	6.797945569	7.7060638	7.014447016	0.369686379	0.691616783
ZmRaf13	9.313627673	8.969110755	9.553672013	9.156736227	0.344516918	0.396935786
ZmMAPKKK22	5.717093852	5.39765161	6.260692066	5.609134973	0.319442241	0.651557093
ZmMAPKKK4	5.692196525	5.381332141	5.051736969	4.805321031	0.310864384	0.246415937
ZmZIK5	6.798246672	6.598506146	6.385781281	6.559547568	0.199740526	-0.173766286
ZmRaf27	7.980814485	7.813456789	8.463429828	7.644492232	0.167357697	0.818937596
ZmRaf3	5.634369727	5.619876665	6.084586514	6.262405428	0.014493062	-0.177818914

ZmRaf48	5.307389694	5.304565809	5.330829948	4.250487882	0.002823885	1.080342066
ZmRaf8	4.675533846	6.686077069	5.020401769	4.72959565	-2.010543223	0.290806119
ZmZIK1	10.09385552	11.90580262	10.11753305	11.35247381	-1.811947105	-1.234940767
ZmZIK4	5.616491563	7.086842371	6.051311611	6.598966122	-1.470350808	-0.547654511
ZmMAPKKK26	5.27517407	6.610889577	6.974709664	6.755000023	-1.335715507	0.219709641
ZmRaf42	5.433701907	6.723931409	5.906491003	6.921752348	-1.290229502	-1.015261346
ZmRaf25	12.02212757	13.17998342	12.03284736	12.84867499	-1.157855853	-0.815827627
ZmMAPKKK24	8.603284666	9.515872955	8.48110041	8.93587197	-0.912588289	-0.45477156
ZmMAPKKK19	3.122488733	3.994537715	4.054041929	4.428658957	-0.872048981	-0.374617028
ZmRaf44	12.05309393	12.85911873	12.00965786	12.84286281	-0.806024803	-0.833204957
ZmRaf5	11.39152877	12.15146194	11.05349106	11.57888824	-0.759933176	-0.525397173
ZmRaf18	5.89235873	6.582025782	6.771543652	7.526038503	-0.689667052	-0.754494851
ZmRaf9	10.98029623	11.6699405	10.87310034	11.26539626	-0.689644269	-0.392295916
ZmZIK2	7.474823641	8.042468285	7.877490428	7.659317647	-0.567644644	0.218172781
ZmZIK6	6.935373239	7.478075967	7.753648509	7.020270619	-0.542702728	0.73337789
ZmRaf15	9.709932613	10.11948442	9.924222955	10.09145835	-0.409551807	-0.16723539
ZmRaf38	6.531919069	6.905286665	7.075857156	7.762922111	-0.373367596	-0.687064955
ZmRaf35	6.617802348	6.978270192	6.322781806	6.594760653	-0.360467845	-0.271978847
ZmMAPKKK18	4.253833586	4.504590288	5.049186055	3.613424905	-0.250756702	1.43576115
ZmRaf11	13.10141887	13.25022488	13.25289585	13.2594104	-0.148806013	-0.006514552
ZmZIK3	7.730162696	7.835001822	7.547250841	8.044467974	-0.104839126	-0.497217132
ZmRaf49	4.886999838	4.974329999	3.852374191	4.102692639	-0.087330161	-0.250318449
ZmRaf26	11.62864962	11.68901412	11.55233054	11.71508905	-0.060364498	-0.162758518
ZmRaf39	10.62829996	10.67119362	10.06093834	9.874994743	-0.042893652	0.1859436
ZmRaf14	8.922694947	8.935517068	9.109013834	9.064372486	-0.012822121	0.044641348
ZmMAP4K1	7.17023491	6.683130257	7.358496603	6.884089897	0.487104652	0.474406706
ZmMAP4K2	7.17023491	6.683130257	7.358496603	6.884089897	0.487104652	0.474406706
ZmMAP4K4	3.47329336	4.493932595	3.564888674	5.198293706	-1.020639236	-1.633405032
ZmMIK	8.78436926	9.587351631	9.276597284	9.539646825	-0.802982371	-0.263049541
ZmMAP4K3	7.420463901	8.049236824	7.749727889	8.521599719	-0.628772924	-0.77187183
ZmMAP4K8	2.707298071	3.106683345	2.796541081	2.497869603	-0.399385274	0.298671479
ZmMAP4K5	11.42759805	11.73388569	11.84504307	11.72480653	-0.306287636	0.120236547
ZmMAP4K7	5.732740809	5.752620162	5.878489116	6.57958658	-0.019879354	-0.701097464
