# Supplementary material for: Maize protein phosphatase gene family: identification and molecular characterization
Source: BMC Genomics. 2014 Sep 9;15(1):773. doi: 10.1186/1471-2164-15-773 (PMC4169795; doi:10.1186/1471-2164-15-773)
Supplement: Supplementary file 23 — Additional file 23: Table S13: List of promoters in ZmPP genes. (PDF 49 KB) [file 12864_2014_6458_MOESM23_ESM.pdf]

Table S13. List of promoters in ZmPP genes.

Gene	*Cis*-regulatory elements
**PP2C**	
ZmPP6	A-box,ABRE,CE3,G-box,MBS
ZmPP31	A-box,ABRE,CCAAT-box,G-box
ZmPP39	A-box,ABRE,CE3,G-box
ZmPP53	A-box,ABRE,CE3,G-box,MBS
ZmPP56	A-box,ABRE,CCAAT-box,CE3,G-box
ZmPP57	A-box,G-box,MBS,MRE,W box
ZmPP107	ABRE,CCAAT-box,G-Box,MBS,W box
ZmPP108	A-box,C-repeat/DRE,G-box,MBS
ZmPP112	A-box,ABRE,CCAAT-box,CE3,G-box,MRE
ZmPP121	A-box,ABRE,CE3,G-box,W box
ZmPP123	A-box,ABRE,C-repeat/DRE,CCAAT-box,G-box,MRE
ZmPP127	A-box,ABRE,G-box
ZmPP130	A-box,ABRE,G-box,MRE
ZmPP132	A-box,ABRE,CE3,G-box
ZmPP134	A-box,ABRE,G-box
ZmPP154	A-box,ABRE,CCAAT-box,G-box
ZmPP8	A-box,ABRE,CCAAT-box,G-box,MBS
ZmPP43	A-box,CCAAT-box,MBS,W box
ZmPP60	A-box,ABRE,G-box,MBS,W box
ZmPP61	ABRE,G-box,MBS,W box
ZmPP97	A-box,ABRE,G-box,MBS,MRE,W box
ZmPP147	ABRE,CCAAT-box,G-box
ZmPP7	G-box
ZmPP24	A-box,ABRE,CCAAT-box,G-box,GCC box,MBS
ZmPP30	A-box,MBS,W box
ZmPP77	CCAAT-box,G-box,MBS
ZmPP126	ABRE,G-box,MBS,W box
ZmPP148	ABRE,G-box
ZmPP155	G-box,MBS,W box
ZmPP2	ABRE,G-box,MRE
ZmPP3	A-box,ABRE,G-box
ZmPP4	A-Box,MBS
ZmPP21	A-box,ABRE,G-box,MBS
ZmPP25	ABRE,CCAAT-box,G-box,GCC box
ZmPP28	A-box,ABRE,CE3,G-box,MBS,W box
ZmPP80	ABRE,G-box,MBS,MRE
ZmPP83	CCAAT-box,G-box,MBS
ZmPP92	CCAAT-box,MBS
ZmPP99	A-box,G-box,MBS,W box

ZmPP144	ABRE,G-box
ZmPP149	CCAAT-box,G-box,MRE
ZmPP151	A-box,CCAAT-box,G-box,MBS,W box
ZmPP12	G-box,MBS
ZmPP35	A-box,ABRE,CCAAT-box,G-Box
ZmPP41	A-box,ABRE,CCAAT-box,G-box,MBS,W box
ZmPP63	G-box,W box
ZmPP69	ABRE,G-box
ZmPP70	A-box,CCAAT-box,G-box,MRE
ZmPP71	
ZmPP74	A-box,G-box,MRE,W box
ZmPP87	ABRE,CCAAT-box,G-box,MBS,W box
ZmPP89	ABRE,G-box
ZmPP105	A-box,ABRE,CCAAT-box,G-box,MBS
ZmPP116	ABRE,CCAAT-box,G-box,GCC box,MBS,W box
ZmPP117	G-Box,MBS,MRE,W box
ZmPP128	ABRE,G-box,MBS,MRE
ZmPP27	g-box,W box
ZmPP40	A-box,ABRE,CCAAT-box,G-box,MBS
ZmPP115	ABRE,G-box
ZmPP152	GCC box,MRE
ZmPP158	G-box,MBS
ZmPP159	G-box,MBS,W box
ZmPP26	A-box,ABRE,G-box,MBS,
ZmPP72	A-box,ABRE,CCAAT-box,CE1,G-box
ZmPP76	A-box,ABRE,CCAAT-box,G-box
ZmPP84	A-box,CCAAT-box,G-box
ZmPP86	ABRE,G-box
ZmPP109	A-box,ABRE,CCAAT-box,G-box,MBS
ZmPP122	C-box,G-box,MBS,W box
ZmPP129	G-box
ZmPP10	A-box,DRE,MBS
ZmPP66	ABRE,G-box,MBS,W box
ZmPP95	ABRE,CCAAT-box,G-box,MBS,W box
ZmPP111	CCAAT-box,G-box,MBS
ZmPP137	A-box,ABRE,G-box,MBS,W box
ZmPP143	ABRE,G-box
ZmPP153	A-box,ABRE,G-box,MBS
ZmPP9	G-box
ZmPP29	ABRE,CCAAT-box,G-Box,MBS,MRE
ZmPP42	ABRE,G-box,MBS
ZmPP49	CCAAT-box,MRE
ZmPP64	MBS,W box

ZmPP90	A-box,CE3,G-box,MBS,W box
ZmPP113	A-box,ABRE,CE3,G-box,GCC box,MBS,W box
ZmPP118	CCAAT-box,G-box,MRE
ZmPP141	A-box,G-box
ZmPP146	A-box,G-box,MBS,W box
ZmPP156	ABRE,G-box,MBS
ZmPP85	ABRE,G-box
ZmPP100	ABRE,G-box,MBS
ZmPP114	ABRE,CCAAT-box,G-box
ZmPP138	ABRE,G-box
ZmPP139	ABRE,G-box
ZmPP157	ABRE,CCAAT-box,G-box,MBS,MRE,W box
ZmPP23	ABRE,G-Box,MBS
ZmPP46	CCAAT-box,G-box
ZmPP78	ABRE,G-box
ZmPP82	A-box,ABRE,G-box,MBS,MRE
ZmPP91	A-box,ABRE,G-box
ZmPP142	ABRE,G-box,MBS
ZmPP44	G-box,GCC box,MBS
ZmPP37	CCAAT-box,G-box,W box
ZmPP58	CCAAT-box,G-box,GCC-box,MBS
ZmPP110	CCAAT-box
**PTP**	
ZmPP1	A-box,ABRE,CCAAT-box,G-box,W box
ZmPP16	A-box,ABRE,CCAAT-box,G-box,MBS,W box
ZmPP18	A-box,ABRE,G-box,MBS
ZmPP20	A-box,ABRE,CCAAT-box,G-box,MBS
ZmPP33	ABRE,G-box,MBS
ZmPP34	A-box,ABRE,G-box,MBS,W box
ZmPP38	ABRE,G-box,MBS
ZmPP45	ABRE,CCAAT-box,G-box
ZmPP48	A-box,CCAAT-box,MBS
ZmPP52	G-box
ZmPP54	A-box,G-box,MBS
ZmPP59	ABRE,CCAAT-box,G-box,GCC box
ZmPP62	A-box,ABRE,CCAAT-box,G-box,MBS
ZmPP67	A-box,G-box,MBS,W box
ZmPP68	ABRE,DRE,G-box,MBS,MRE
ZmPP93	MBS
ZmPP94	ABRE,C-repeat/DRE,G-box,W box
ZmPP101	A-box,ABRE,CCAAT-box,G-box
ZmPP102	ABRE,G-box,MBS

ZmPP106	A-box,ABRE,G-box
ZmPP120	A-box,ABRE,G-box,MBS
ZmPP125	
ZmPP131	A-box,ABRE,G-box,MBS,W box
ZmPP133	ABRE,G-box
ZmPP140	ABRE,G-box
ZmPP51	MBS
ZmPP47	G-box,NBS
ZmPP14	G-Box,MBS
ZmPP145	G-box,MBS
**PP2A**	
ZmPP5	ABRE,G-box,MBS,W box
ZmPP11	A-box,G-box
ZmPP13	
ZmPP15	G-box,MBS
ZmPP17	ABRE,CCAAT-box,G-Box
ZmPP19	A-box,ABRE,G-box,MBS
ZmPP22	CCAAT-box,G-box,MBS,MRE
ZmPP32	ABRE,CCAAT-box,G-box,MBS
ZmPP36	ABRE,G-box
ZmPP50	A-box,G-box
ZmPP55	ABRE,G-box,MBS
ZmPP65	G-box,MBS
ZmPP73	A-box,ABRE,CCAAT-box,G-box,MBS,MRE,W box
ZmPP75	ABRE,CCAAT-box,G-box,MBS
ZmPP79	A-box,ABRE,CE3,G-box,MBS,MRE
ZmPP81	G-box,MBS,MRE
ZmPP88	A-box,CCAAT-box,MBS
ZmPP96	A-box,ABRE,G-box,MBS
ZmPP98	ABRE,CCAAT-box,G-box,MBS
ZmPP103	A-box,CCAAT-box,MBS
ZmPP104	MBS,MRE,W box
ZmPP119	G-box,MBS
ZmPP124	ABRE,CCAAT-box,G-box,MBS,W box
ZmPP135	C-box,G-box
ZmPP136	G-box,MRE
ZmPP150	G-box,MBS
