# Supplementary material for: Maize protein phosphatase gene family: identification and molecular characterization
Source: BMC Genomics. 2014 Sep 9;15(1):773. doi: 10.1186/1471-2164-15-773 (PMC4169795; doi:10.1186/1471-2164-15-773)
Supplement: Supplementary file 24 — Additional file 24: Table S14: List of predicted miRNA-regulated ZmPP genes. (PDF 144 KB) [file 12864_2014_6458_MOESM24_ESM.pdf]

Table S14. List of predicted miRNA-regulated ZmPP genes.

<table>
<tr><th>miRNA</th><th>Target Gene</th><th>Name</th><th>Score</th><th>Mismatches</th><th>Alignment</th></tr>
<tr><td rowspan="2">zma-miR529-5p</td><td>GRMZM2G074489</td><td>*ZmPP152*</td><td>88</td><td>2.5</td><td>3' UCCGACAUGAGAGAGAGAAGA 5'
||||||| ||:|:|:||||||
5' AGGCUGUUCUUUUUUUCUUCU 3'</td></tr>
<tr><td>GRMZM2G151087</td><td>*ZmPP47*</td><td>78</td><td>4</td><td>3' UCCGACAUGAGAGAGAGAAGA 5'
: |||||:||||||: :||||
5' GUGCUGUGCUCUCUUGUUUCU 3'</td></tr>
<tr><td rowspan="2">zma-miR159k-3p</td><td>GRMZM2G038195</td><td>*ZmPP75*</td><td>86</td><td>3</td><td>3' GUCUCGAGGGAAGUUAGGUUU 5'
|||| ||:||||||||:|||
5' CAGAACUUCCUUCAAUUCAAC 3'</td></tr>
<tr><td>GRMZM2G133464</td><td>*ZmPP88*</td><td>83</td><td>3.5</td><td>3' GUCUCGAGGGAAGUUAGGUUU 5'
|||| ||:||||||||:|:|
5' CAGAACUUCCUUCAAUUCGAC 3'</td></tr>
<tr><td>zma-miR169m-5p</td><td>GRMZM2G010855</td><td>*ZmPP6*</td><td>77</td><td>4</td><td>3' AUCCGUUCGGUAAGAACCGAU 5'
|:| | ||||:|||||||:|:
5' UGGACUAGCCGUUCUUGGUUG 3'</td></tr>
<tr><td>zma-miR166j-5p</td><td>GRMZM2G136765</td><td>*ZmPP128*</td><td>82</td><td>4</td><td>3' GGA-ACUUGGUC-UGUUUGUUUGG 5'
||| ||||::|| ||||||||||::
5' CCUCUGAAUUAGUACAAACAAAUU 3'</td></tr>
<tr><td>zma-miR399d-3p</td><td>GRMZM2G127374</td><td>*ZmPP16*</td><td>83</td><td>3</td><td>3' GUCCCGUCGAGAGGAAACCGU 5'
|| ||:||||:|||||||| |
5' CAUGGUAGCUUUCCUUUGGAA 3'</td></tr>
<tr><td>zma-miR169a-3p</td><td>GRMZM2G161544</td><td>*ZmPP117*</td><td>75</td><td>4</td><td>3' ACAUCGGUUCUUGUUGAACGG 5'
||||||::||| |:||||:|
5' AGUAGCCGGGAA-AGCUUGUC 3'</td></tr>
<tr><td>zma-miR399i-3p</td><td>GRMZM2G127374</td><td>*ZmPP16*</td><td>80</td><td>3.5</td><td>3' GUCCCGUUGAGAGGAAACCGU 5'
|| ||:|:||:|||||||| |
5' CAUGGUAGCUUUCCUUUGGAA 3'</td></tr>
<tr><td>zma-miR399e-3p</td><td>GRMZM2G127374</td><td>*ZmPP16*</td><td>80</td><td>3.5</td><td>3' GUCCCGUUGAGAGGAAACCGU 5'
|| ||:|:||:|||||||| |
5' CAUGGUAGCUUUCCUUUGGAA 3'</td></tr>
<tr><td rowspan="2">zma-miR159f-3p</td><td>GRMZM2G038195</td><td>*ZmPP75*</td><td>86</td><td>3</td><td>3' GUCUCGAGGGAAGUUAGGUUU 5'
|||| ||:||||||||:|||
5' CAGAACUUCCUUCAAUUCAAC 3'</td></tr>
<tr><td>GRMZM2G133464</td><td>*ZmPP88*</td><td>83</td><td>3.5</td><td>3' GUCUCGAGGGAAGUUAGGUUU 5'
|||| ||:||||||||:|:|
5' CAGAACUUCCUUCAAUUCGAC 3'</td></tr>
<tr><td>zma-miR164f-3p</td><td>GRMZM2G159811</td><td>*ZmPP154*</td><td>84</td><td>3</td><td>3' CAACCUCUUCCUCGCGUGCAC 5'
| ||||:||||||||:||||
5' GGCGGAGGAGGAGCGCGCGUG 3'</td></tr>
<tr><td>zma-miR169n-5p</td><td>GRMZM2G010855</td><td>*ZmPP6*</td><td>77</td><td>4</td><td>3' AUCCGUUCGGUAAGAACCGAU 5'
|:| | ||||:|||||||:|:
5' UGGACUAGCCGUUCUUGGUUG 3'</td></tr>
<tr><td>zma-miR171f-3p</td><td>GRMZM2G109496</td><td>*ZmPP157*</td><td>75</td><td>4</td><td>3' ACACUAUAACCGUGCCGAGUU 5'
||| ||| |||: |||:||||
5' UGUCAUACUGGUUCGGUUCAA 3'</td></tr>
<tr><td>zma-miR167e-3p</td><td>GRMZM2G420926</td><td>*ZmPP150*</td><td>77</td><td>4</td><td>3' CUACUUUGACGUGUCGUACUAG 5'
||||:||| |:||:||| |||:
5' GAUGGAAC-GUACGGCAAGAUU 3'</td></tr>
<tr><td>zma-miR169n-3p</td><td>GRMZM2G161544</td><td>*ZmPP117*</td><td>77</td><td>3.5</td><td>3' GAAUCGGUUCUUCCGGACGG 5'
||||||:::||| ||:||:|
5' CUUAGCUGGGAAAGCUUGUC 3'</td></tr>
<tr><td>zma-miR160f-5p</td><td>GRMZM2G108309</td><td>*ZmPP144*</td><td>77</td><td>4</td><td>3' GCCGUAUGUC-CCUCGGUCCGU 5'
|||:||::: |||||||||||</td></tr>
</table>

<table>
<tr><td></td><td></td><td></td><td></td><td></td><td>5' CGGUAUGUGAAGGAGCCAGGCA 3'</td></tr>
<tr><td>zma-miR169o-3p</td><td>GRMZM5G818101</td><td>ZmPP121</td><td>77</td><td>3.5</td><td>3' CGAUCGGUUCUUCUGGACGG 5'
|||:|||:||:||::| |||
5' GCUGGCCGAGGAGGUCGGCC 3'</td></tr>
<tr><td>zma-miR169r-5p</td><td>GRMZM2G001243</td><td>ZmPP127</td><td>77</td><td>3.5</td><td>3' GGCCGUUCAGUAGGAACCGAC 5'
|||||::|| ||::||||:||
5' CCGGCGGGU-AUUUUUGGUUG 3'</td></tr>
<tr><td>zma-miR164h-5p</td><td>GRMZM2G319357</td><td>ZmPP14</td><td>78</td><td>3.5</td><td>3' GUGU-GCACGGGACGAAGAGGU 5'
|||| | |||||| |||||||:
5' CACAGCCUGCCCUCCUUCUCCG 3'</td></tr>
<tr><td rowspan="4">zma-miR159e-5p</td><td>GRMZM2G021610</td><td>ZmPP41</td><td>75</td><td>4</td><td>3' CUUGUCUACGACGUCCUCGAC 5'
| :|||: ||||||||||| |
5' GCGCAGGAGCUGCAGGAGCCG 3'</td></tr>
<tr><td>GRMZM2G161544</td><td>ZmPP117</td><td>78</td><td>3.5</td><td>3' CUUG-UCUACGACGUCCUCG-AC 5'
||:| || |||||||||||| ||
5' GAGCCAGCUGCUGCAGGAGCCUG 3'</td></tr>
<tr><td>GRMZM2G028700</td><td>ZmPP11</td><td>75</td><td>4</td><td>3' CUUGUCUACG-ACGUCCUCGAC 5'
|:||| ||| ||||||||:||
5' GGACACAUGGAUGCAGGAGUUG 3'</td></tr>
<tr><td>GRMZM2G009593</td><td>ZmPP50</td><td>75</td><td>4</td><td>3' CUUGUCUACG-ACGUCCUCGAC 5'
|:||| ||| ||||||||:||
5' GGACACAUGGAUGCAGGAGUUG 3'</td></tr>
<tr><td>zma-miR2118f</td><td>GRMZM2G119079</td><td>ZmPP140</td><td>86</td><td>3</td><td>3' AUCCGUACCUUCCGUAACCCUU 5'
||||:||||:|| ||||||||
5' GAGGCGUGGAGGG-AUUGGGAA 3'</td></tr>
<tr><td>zma-miR393c-3p</td><td>GRMZM2G003640</td><td>ZmPP20</td><td>80</td><td>4</td><td>3' UAAGGUUUCCCUAACGUGACUG 5'
| ||| :|||||| |||||||:
5' AGUCCCGAGGGAUAGCACUGAU 3'</td></tr>
<tr><td>zma-miR169b-3p</td><td>GRMZM2G161544</td><td>ZmPP117</td><td>75</td><td>4</td><td>3' ACAUCGGUUCUUGUUGAACGG 5'
||||||::||| |:||||:|
5' AGUAGCCGGGAA-AGCUUGUC 3'</td></tr>
<tr><td>zma-miR169q-5p</td><td>GRMZM2G010855</td><td>ZmPP6</td><td>77</td><td>4</td><td>3' AUCCGUUCGGUAAGAACCGAU 5'
|:| | ||||:|||||||:|:
5' UGGACUAGCCGUUCUUGGUUG 3'</td></tr>
<tr><td rowspan="2">zma-miR159a-3p</td><td>GRMZM2G038195</td><td>ZmPP75</td><td>86</td><td>3</td><td>3' GUCUCGAGGGAAGUUAGGUUU 5'
|||| ||:||||||||:|||
5' CAGAACUUCCUUCAAUUCAAC 3'</td></tr>
<tr><td>GRMZM2G133464</td><td>ZmPP88</td><td>83</td><td>3.5</td><td>3' GUCUCGAGGGAAGUUAGGUUU 5'
|||| ||:||||||||:|:|
5' CAGAACUUCCUUCAAUUCGAC 3'</td></tr>
<tr><td rowspan="3">zma-miR396a-5p</td><td>GRMZM2G112240</td><td>ZmPP65</td><td>78</td><td>3.5</td><td>3' GUC-AAGUU-C-UUUCGACACCUU 5'
||| ||||: | ||||||||||||
5' CAGAUUCAGUGUAAAGCUGUGGAA 3'</td></tr>
<tr><td>GRMZM2G338631</td><td>ZmPP73</td><td>78</td><td>3.5</td><td>3' GUC-AAGUU-C-UUUCGACACCUU 5'
||| ||||: | ||||||||||||
5' CAGAUUCAGUGUAAAGCUGUGGAA 3'</td></tr>
<tr><td>GRMZM2G390076</td><td>ZmPP96</td><td>78</td><td>3.5</td><td>3' GUC-AAGUU-C-UUUCGACACCUU 5'
||| ||||: | ||||||||||||
5' CAGAUUCAGUGUAAAGCUGUGGAA 3'</td></tr>
<tr><td>zma-miR2118a</td><td>GRMZM2G081359</td><td>ZmPP64</td><td>80</td><td>4</td><td>3' AUCCUUACUCUCCGUAGUCCUU 5'
|||::||||||||| |:||:|
5' AAGGGGUGAGAGGCA-CGGGGA 3'</td></tr>
<tr><td>zma-miR156j-5p</td><td>GRMZM2G158734</td><td>ZmPP137</td><td>81</td><td>4</td><td>3' ACACGAGAGAGAGAAGACAGU 5'
||||||||:||||||| :|
5' ACUGCUCUCUUUCUUCUGGUA 3'</td></tr>
</table>

<table>
<tr><td rowspan="4">zma-miR164a-3p</td><td>GRMZM2G019819</td><td>ZmPP31</td><td>75</td><td>3.5</td><td>3' CUACCUCUUCCUCUUGUGCAC 5'
|| ||||:||||| :|:||||
5' GA-GGAGGAGGAGCGCGCGUG 3'</td></tr>
<tr><td>GRMZM2G082487</td><td>ZmPP39</td><td>75</td><td>4</td><td>3' CUACCUCUUCCUCUUGUG-CAC 5'
|| ||||| ||||:||:| |||
5' GAGGGAGAUGGAGGACGCCGUG 3'</td></tr>
<tr><td>GRMZM5G818101</td><td>ZmPP121</td><td>78</td><td>4</td><td>3' CUACCUCUUCCUCUUGUGCAC 5'
|| ||||| ||||:||:|||
5' GAGGGAGAUGGAGGACGCGUU 3'</td></tr>
<tr><td>GRMZM2G159811</td><td>ZmPP154</td><td>77</td><td>4</td><td>3' CUACCUCUUCCUCUUGUGCAC 5'
|: ||||:||||| :|:||||
5' GGCGGAGGAGGAGCGCGCGUG 3'</td></tr>
<tr><td rowspan="2">zma-miR159b-3p</td><td>GRMZM2G038195</td><td>ZmPP75</td><td>86</td><td>3</td><td>3' GUCUCGAGGGAAGUUAGGUUU 5'
|||| ||:||||||||:|||
5' CAGAACUUCCUUCAAUUCAAC 3'</td></tr>
<tr><td>GRMZM2G133464</td><td>ZmPP88</td><td>83</td><td>3.5</td><td>3' GUCUCGAGGGAAGUUAGGUUU 5'
|||| ||:||||||||:|:|
5' CAGAACUUCCUUCAAUUCGAC 3'</td></tr>
<tr><td>zma-miR171d-5p</td><td>GRMZM2G360455</td><td>ZmPP85</td><td>75</td><td>4</td><td>3' AGACUCACUCGGCUCGGUUGU 5'
||||| | ||:|:|||||||
5' UCUGACUUUGCUGGGCCAACA 3'</td></tr>
<tr><td>zma-miR827-3p</td><td>GRMZM2G003096</td><td>ZmPP46</td><td>75</td><td>4</td><td>3' ACAAACGACUACCAGUAGAUU 5'
||| | :||:|| ||||||||
5' UGUCUAUUGGUGCUCAUCUAA 3'</td></tr>
<tr><td>zma-miR169c-5p</td><td>GRMZM2G001243</td><td>ZmPP127</td><td>77</td><td>3.5</td><td>3' GGCCGUUCAGUAGGAACCGAC 5'
|||||::|| ||::||||:||
5' CCGGCGGGU-AUUUUUGGUUG 3'</td></tr>
<tr><td>zma-miR164e-5p</td><td>GRMZM2G067910</td><td>ZmPP70</td><td>76</td><td>3.5</td><td>3' GAGUGCACAGGACGAAGAGGU 5'
||| ||| ||:|||||||||
5' CUC-CGUUUCUUGCUUCUCCU 3'</td></tr>
<tr><td rowspan="5">zma-miR399b-3p</td><td>AC155624.2_FG006</td><td>ZmPP2</td><td>75</td><td>3.5</td><td>3' GUCCUGUCGAGAGGAAACCGU 5'
||||||:|: ||||| |||:|
5' CAGGACGGU-CUCCUCUGGUA 3'</td></tr>
<tr><td>GRMZM5G891266</td><td>ZmPP4</td><td>75</td><td>3.5</td><td>3' GUCCUGUCGAGAGGAAACCGU 5'
||||||:|: ||||| |||:|
5' CAGGACGGU-CUCCUCUGGUA 3'</td></tr>
<tr><td>GRMZM5G833774</td><td>ZmPP3</td><td>75</td><td>3.5</td><td>3' GUCCUGUCGAGAGGAAACCGU 5'
||||||:|: ||||| |||:|
5' CAGGACGGU-CUCCUCUGGUA 3'</td></tr>
<tr><td>GRMZM2G010017</td><td>ZmPP151</td><td>78</td><td>3</td><td>3' GUCCUGUCGAGAGGAAACCGU 5'
||||||:|| ||||| |||:|
5' CAGGACGGC-CUCCUCUGGUA 3'</td></tr>
<tr><td>GRMZM2G127374</td><td>ZmPP16</td><td>80</td><td>3.5</td><td>3' GUCCUGUCGAGAGGAAACCGU 5'
|| |::||||:|||||||| |
5' CAUGGUAGCUUUCCUUUGGAA 3'</td></tr>
<tr><td>zma-miR166m-3p</td><td>GRMZM2G319357</td><td>ZmPP14</td><td>75</td><td>4</td><td>3' CUCCU-UACUUCGGACCAGGCU 5'
||||| | ||:|||| |||||:
5' GAGGACAGGAGGCCUCGUCCGG 3'</td></tr>
<tr><td>zma-miR171e-5p</td><td>GRMZM2G360455</td><td>ZmPP85</td><td>75</td><td>4</td><td>3' AGACUCACUCGGCUCGGUUGU 5'
||||| | ||:|:|||||||
5' UCUGACUUUGCUGGGCCAACA 3'</td></tr>
<tr><td rowspan="2">zma-miR159j-3p</td><td>GRMZM2G038195</td><td>ZmPP75</td><td>86</td><td>3</td><td>3' GUCUCGAGGGAAGUUAGGUUU 5'
|||| ||:||||||||:|||
5' CAGAACUUCCUUCAAUUCAAC 3'</td></tr>
<tr><td>GRMZM2G133464</td><td>ZmPP88</td><td>83</td><td>3.5</td><td>3' GUCUCGAGGGAAGUUAGGUUU 5'</td></tr>
</table>

<table>
<tr><td></td><td></td><td></td><td></td><td></td><td>|||| ||:||||||||:|:|
5' CAGAACUUCCUUCAAUUCGAC 3'</td></tr>
<tr><td rowspan="2">zma-miR399j-3p</td><td>GRMZM2G127374</td><td>ZmPP16</td><td>80</td><td>3.5</td><td>3' GUCCCGUUGAGAGGAAACCGU 5'
|| ||:|:||:|||||||| |
5' CAUGGUAGCUUUCCUUUGGAA 3'</td></tr>
<tr><td>GRMZM2G112240</td><td>ZmPP65</td><td>78</td><td>3.5</td><td>3' GUC-AAGUU-C-UUUCGACACCUU 5'
||| ||||: | ||||||||||||
5' CAGAUUCAGUGUAAAGCUGUGGAA 3'</td></tr>
<tr><td rowspan="2">zma-miR396b-5p</td><td>GRMZM2G338631</td><td>ZmPP73</td><td>78</td><td>3.5</td><td>3' GUC-AAGUU-C-UUUCGACACCUU 5'
||| ||||: | ||||||||||||
5' CAGAUUCAGUGUAAAGCUGUGGAA 3'</td></tr>
<tr><td>GRMZM2G390076</td><td>ZmPP96</td><td>78</td><td>3.5</td><td>3' GUC-AAGUU-C-UUUCGACACCUU 5'
||| ||||: | ||||||||||||
5' CAGAUUCAGUGUAAAGCUGUGGAA 3'</td></tr>
<tr><td>zma-miR166l-3p</td><td>GRMZM2G319357</td><td>ZmPP14</td><td>75</td><td>4</td><td>3' CUCCU-UACUUCGGACCAGGCU 5'
||||| | ||:|||| |||||:
5' GAGGACAGGAGGCCUCGUCCGG 3'</td></tr>
<tr><td>zma-miR393a-3p</td><td>GRMZM2G003640</td><td>ZmPP20</td><td>83</td><td>3.5</td><td>3' UAAGGUUUCCCUAACGUGACUA 5'
| ||| :|||||| ||||||||
5' AGUCCCGAGGGAUAGCACUGAU 3'</td></tr>
<tr><td>zma-miR164d-3p</td><td>GRMZM2G031094</td><td>ZmPP68</td><td>75</td><td>4</td><td>3' UACCUCUUCCUCUGGUGCAC 5'
||| :|||||||:||::||
5' AUGAGGAAGGAGGCCGUGUA 3'</td></tr>
</table>
